# Supplementary material for: Bacteriophage-related epigenetic natural and non-natural pyrimidine nucleotides and their influence on transcription with T7 RNA polymerase
Source: Commun Chem. 2024 Nov 9;7:256. doi: 10.1038/s42004-024-01354-5 (PMC11550810; doi:10.1038/s42004-024-01354-5)
Supplement: Supplementary file 2 — Supplementary Information [file 42004_2024_1354_MOESM2_ESM.pdf]

Supplementary Information

Bacteriophage-related epigenetic natural and non-natural pyrimidine nucleotides and their influence on transcription with T7 RNA polymerase

Table of contents

1. Experimental section – organic chemistry part 4
  1.1. General remarks – synthetic part 4
  1.2. Synthetic schemes 5
  1.3. Synthesis of nucleosides 7
  1.4. Synthesis of nucleoside triphosphates 21
2. Experimental section - biochemistry part 33
  2.1. Scheme of used nucleoside triphosphates 33
  2.2. General remarks – biochemistry 33
  2.3. General annealing procedure 35
  2.4. Initial testing of prepared nucleoside triphosphates 35
    2.4.1. Preparation of 19DNA and 19DNA_U^X by PEX 35
    2.4.2. Preparation of 31DNA and 31DNA_U^X by PEX 37
    2.4.3. Preparation of 98DNA and 98DNA_U^X by PCR 39
    2.4.4. TFA deprotection on 98DNA_U^{tfa} to prepare 98DNA_U^{am} 40
  2.5. Preparation of DNA by PEX 41
    2.5.1. Preparation of 37DNA, 37DNA_U^X and 37DNA_C^X by PEX 41
    2.5.2. Preparation of 87DNA and 87DNA_U^X by PEX 43
    2.5.3. Preparation of 87DNA and 87DNA_C^X by PEX 45
  2.6. Preparation of single stranded DNA 47
    2.6.1. Preparation of 37ON/37ON_U^X and 87ON/87ON_U^X by Lambda exonuclease digestion of 37DNA/37DNA_U^X and 87DNA/87DNA_U^X 47
  2.7. Preparation of DNA templates used for transcriptions 48
    2.7.1. General notes for ligation reactions 48
    2.7.2. Preparation of 107DNA_S/107DNA_S_U^X/107DNA_S_C^X by a ligation reaction between 70DNA and 37DNA/37DNA_U^X/37DNA_C^X 49
    2.7.3. Preparation of 107DNA_A/107DNA_A_U^X/107DNA_A_C^X by a ligation reaction between 20DNA and 87DNA/87DNA_U^X/87DNA_C^X 51
    2.7.4. Preparation of 107DNA_P and 107DNA_P_U^X by PEX 53
    2.7.5. Preparation of 107DNA_F and 107DNA_F_U^X by PCR 55
  2.8. Multiple round transcription experiments 56
    2.8.1. Quantification of templates used in *in vitro* transcriptions 56

2.8.2. Transcription reaction using 107DNA_S and 107DNA_S_U^X/107DNA_S_C^X containing sense-modified promoter........ 56

2.8.3. Transcription reaction using 107DNA_A and 107DNA_A_U^X/107DNA_A_C^X containing antisense-modified promoter........ 58

2.8.4. Transcription reaction using 107DNA_P and 107DNA_P_U^X containing fully modified promoter........ 59

2.8.5. Transcription reaction using 107DNA_F and fully modified 107DNA_F_U^X.... 60

2.9. Preparation of samples from RNA for next generation sequencing........ 62

2.9.1. Reverse transcription of 70RNA_F from *in vitro* transcription reaction to prepare 108RT……………........ 62

2.9.2. Preparation of DNA for next generation sequencing by PCR........ 63

3. LC-MS spectra........ 65

3.1. LC-MS spectra of 19DNA and 19DNA_U^X........ 65

3.2. LC-MS spectra of 31DNA and 31DNA_U^X........ 77

3.3. LC-MS spectra of 37DNA/37DNA_U^X/37DNA_C^X........ 89

3.4. LC-MS spectra of 87DNA/87DNA_U^X/87DNA_C^X........ 105

3.5. LC-MS spectra of 98DNA_PCR/98DNA_U^{tfa} and 98DNA_U^{am}........ 120

4. Copies of IR spectra........ 126

5. Next generation sequencing........ 132

5.1. NGS results........ 133

6. Supplementary references........ 136

1. **Experimental section – organic chemistry part**

1.1. General remarks – synthetic part

Reagents and solvents were purchased from commercial suppliers (Fluorochem, Sigma–Aldrich, Acros Organics and Alfa Aesar), and were used without further purification unless stated otherwise. Phosphoryl chloride ($POCl_3$) and trimethyl phosphate [$PO(OMe)_3$] were distilled prior to use. Dried solvents were purchased from Acros Organics. Unless stated otherwise, all reactions were performed in heatgun-dried glassware under argon atmosphere, using standard septa techniques. The reactions were monitored by thin-layer chromatography (TLC) using silica gel 60 F254 plates (Merck) and visualized by UV (254 nm) or with Advion Expression Compact Mass Spectrometer connected with Plate Express® TLC Plate Reader using electrospray ionization. Column chromatography was performed using silica gel (40–63 μm, Fluorochem) either by flash or by HPFC chromatography system (FLC) Teledyne ISCO CombiFlash Rf 200 or 300. Reverse phase (RP) and diol-modified columns for FLC were purchased from Teledyne ISCO. Purifications of nucleoside triphosphates and some nucleosides were performed using HPLC (Waters modular HPLC system), using columns Phenomenex Kinetex EVO C18 (Kinetex® 5 µm EVO C18 100 Å, AXIA Packed LC Column 250 x 21.2 mm) or Waters X-Bridge Shield RP18 (XBridge BEH Shield RP18 OBD Prep Column, 130Å, 5 µm, 19 x 150 mm). Buffer A (0.1 M TEAB in H_2O) and buffer B (0.1 M TEAB in 50% MeOH) were used for purification of nucleoside triphosphates on RP-HPLC columns. For ion-exchange separation, Sepharose column (Sepharose DEAE Fast Flow, lab-packed, 12.5 x 2.3 mm) was used. NMR spectra were measured on Bruker AVANCE 400 III HD (^{1}H at 401.0 MHz and ^{13}C at 100.8 MHz), Bruker AVANCE 500 III HD (^{1}H at 500.0 MHz, ^{13}C at 125.7 MHz, ^{31}P at 202.4 MHz, and ^{19}F at 470.7 MHz), Bruker AVANCE 600 III HD (^{1}H at 600.1 MHz and ^{13}C at 150.9 MHz) in CD_3OD, $CDCl_3$ or D_2O solutions at 25 °C. Chemical shifts (in ppm, δ scale) were referenced to the residual solvent signal in ^{1}H spectra (δ (CHD_2OD) = 3.31 ppm, δ ($CHCl_3$) = 7.26 ppm) or to the solvent signal in ^{13}C spectra (δ (CD_3OD) = 49.0 ppm, δ ($CDCl_3$) = 77.16 ppm. NMR spectra measured in D_2O were referenced to the signal of *t*-BuOH (10% v/v solution in D_2O, 1 drop) as the internal standard (1.24 ppm in ^{1}H, 32.43 ppm in 13C). ^{31}P NMR spectra were referenced to H_3PO_4 signal (0 ppm) as the external standard in 1 mm coaxial capillary. ^{19}F NMR spectra were referenced to C_6F_6 signal (-163 ppm) as the external standard in 1 mm coaxial capillary. ^{1}H and ^{13}C NMR spectra measured

in D_2O on Bruker Avance 400 HD were referenced using the default Topspin reference frequency. Coupling constants (*J*) are given in Hz. The complete assignment of ^{1}H and ^{13}C signals was performed by an analysis of the correlated homonuclear H,H-COSY, heteronuclear H,C-HSQC and H,C-HMBC spectra. High resolution mass spectra were measured on LTQ Orbitrap XL spectrometer (Thermo Fisher Scientific). Infrared spectra were measured on Bruker ALPHA FTIR ATR spectrometer and analyzed by OPUS 6.5. Compounds dT^{N3}, dU^{I}TP, **dU^{hm}TP**, **dU^{et}TP**, **dU^{She}TP**, **dU^{ac}TP**, **dC^{hm}TP**, **dC^{et}TP**, **dC^{She}TP** and **dC^{ac}TP** were prepared according to published procedures.[1,2,3]

1.2. Synthetic schemes

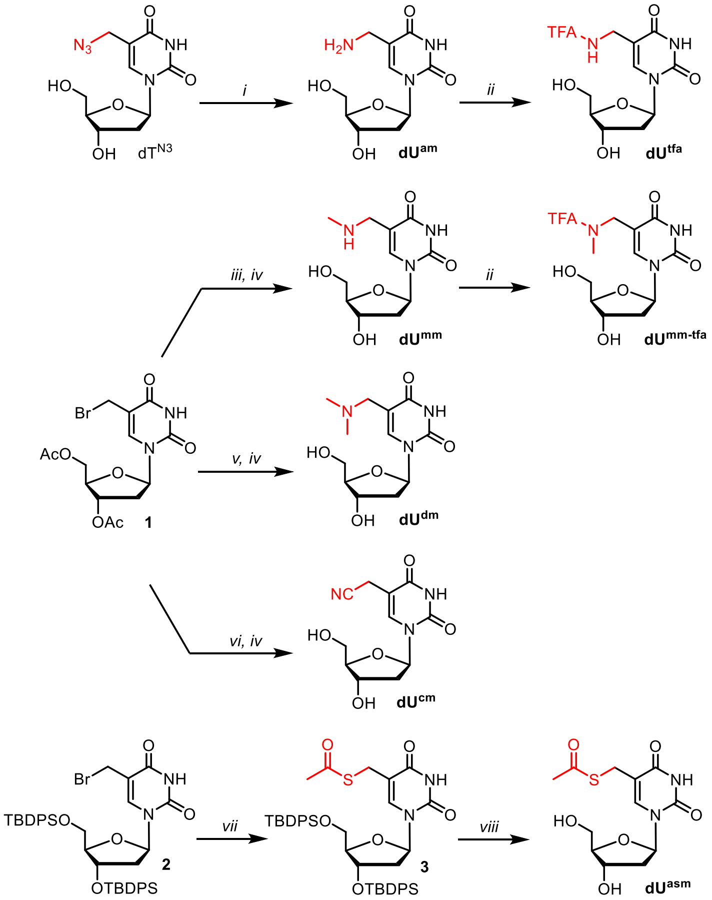

Scheme S1. Synthetic overview of a set of modified nucleosides. Conditions: *i*) H_2, EtOH, H_2O, 23 °C, 1.5 h; *ii*) TFAEt, Et_3N, MeOH, 23 °C, 16 h; *iii*) $MeNH_2$, Et_3N, MeCN, 0 °C, 15 min; *iv*) NH_4OH, MeOH, 23 °C, 2 h; *v*) Me_2NH, Et_3N, MeCN, –20 °C, 1.5 h; *vi*) KCN, DMF, 50 °C, 16 h; *vii*) AcSK, Et_3N, DMF, 75 °C, 1.5 h; *viii*) TBAF, AcOH, THF, 23 °C, 4 h.

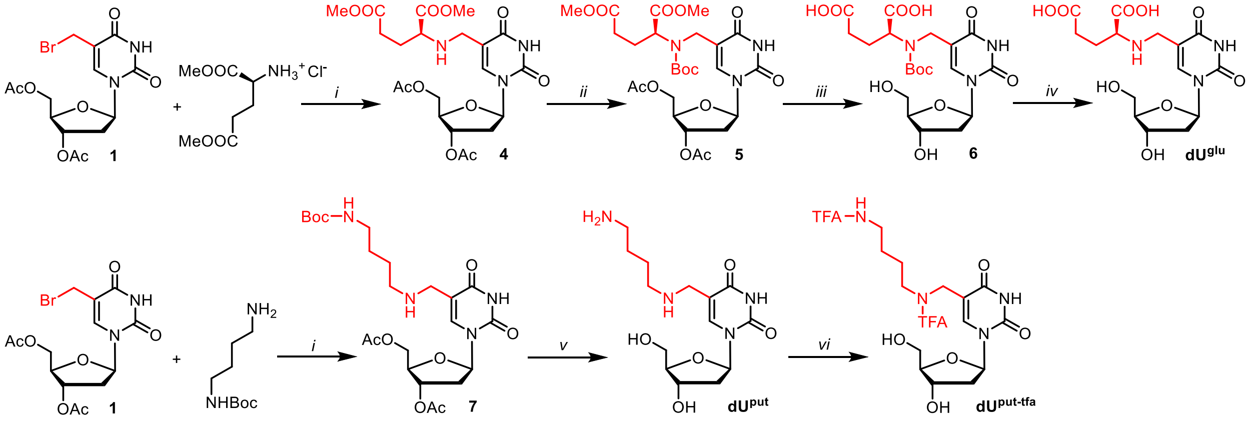

Scheme S2. Synthetic overview of a set of modified nucleosides. Conditions: *i*) Et_3N, DCM, 23 °C, 3 h; *ii*) Boc_2O, MeOH, 23 °C, 48 h; *iii*) K_2CO_3, H_2O, MeOH, 23 °C, 24 h; *iv*) TFA, DCM, 23 °C, 35 min; *v*) AcCl, MeOH, 23 °C; *vi*) TFAEt, Et_3N, MeOH, 23 °C, 16 h.

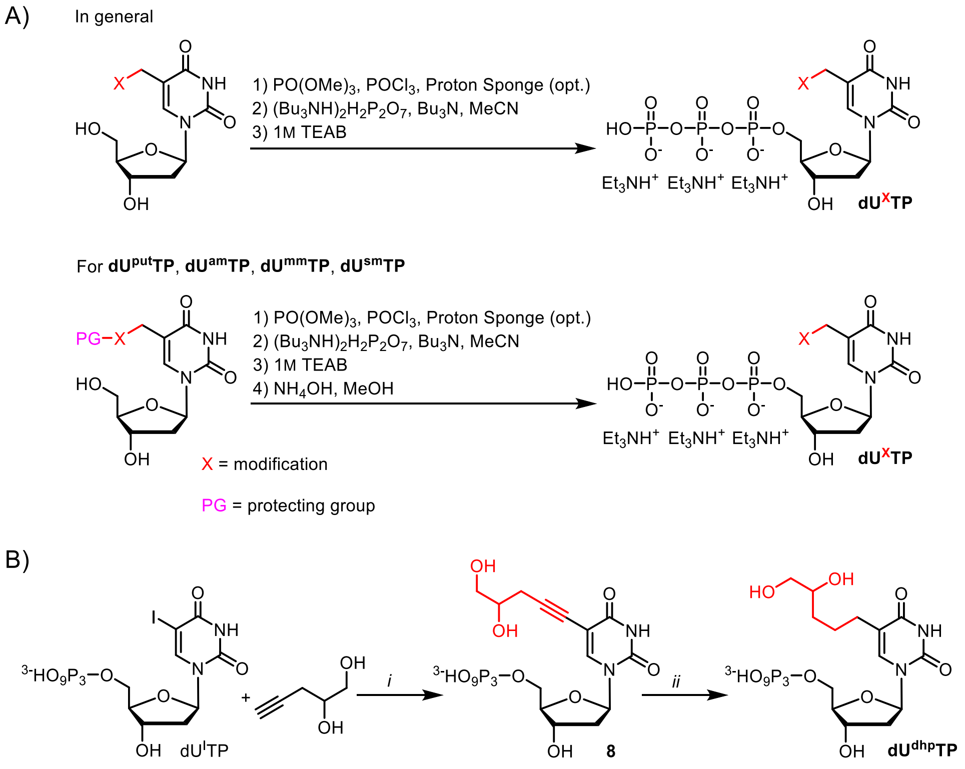

Scheme S3. Preparation of nucleoside triphosphates. A) Triphosphorylation procedures. B) Preparation of **dU^dhp^TP**. Conditions: *i*) $Pd(OAc)_2$, CuI, TPPTS, Et_3N, 80 °C, 1 h; *ii*) H_2, H_2O, MeOH, 23 °C, 24 h.

1.3. Synthesis of nucleosides

5-Bromomethyl-3′,5′-di-*O*-acetyl-2′-deoxyuridine[4] (1)

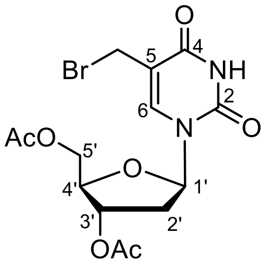

The compound was synthesized according to published procedure.[4] After the reaction was finished and hot-filtered, the solution was either kept under argon atmosphere at –20 °C for up to 1 week or evaporated and used immediately in the next step. The estimated yield (based on TLC) was 70%.

5-Aminomethyl-2′-deoxyuridine formate[5] (dU^{am})

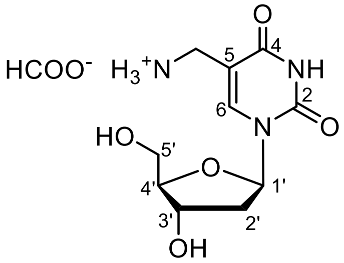

5-Azidomethyl-2’-deoxyuridine[1] (150 mg, 0.53 mmol) was dissolved in H_2O (3 mL) and EtOH (2 mL). The mixture was briefly evacuated and filled with Ar a total of three times. 10% Pd/C (56.4 mg, 0.053 mmol of Pd) was added, followed by an H_2 balloon. The reaction was stirred in H_2 atmosphere for 1.5 hours at 23 °C, followed by filtration and evaporation of the filtrate. The crude product was purified using HPLC (0 to 20% MeOH in H_2O containing 0.1% formic acid, Kinetex EVO C18). Pure product **dU^{am}** (112 mg, 70%) was acquired in a salt form with formic acid as a white hydroscopic solid.

^{1}H NMR (401.0 MHz, D_2O): 2.34 (dt, 1H, J_{gem} = 14.2, $J_{2'b,1'}$ = $J_{2'b,3'}$ = 6.6, H-2′b); 2.42 (ddd, 1H, J_{gem} = 14.2, $J_{2'a,1'}$ = 6.6, $J_{2'a,3'}$ = 4.2, H-2′a); 3.75 (dd, 1H, J_{gem} = 12.6, $J_{5'b,4'}$ = 4.9, H-5′b); 3.84 (dd, 1H, J_{gem} = 12.6, $J_{5'a,4'}$ = 3.4, H-5′a); 3.91, 3.95 (2 × dd, 2 × 1H, J_{gem} = 14.0, $J_{CH2,6}$ = 0.7, CH_2N);

4.04 (ddd, 1H, $J_{4',5'}$ = 4.9, 3.4, $J_{4',3'}$ = 4.2, H-3′); 4.44 (dt, 1H, $J_{3',2'}$ = 6.6, 4.2, $J_{3',4'}$ = 4.2, H-3′); 6.25 (t, 1H, $J_{1',2'}$ = 6.5, H-1′); 8.06 (s, 1H, H-6); 8.43 (s, 1H, HCOO).

^{13}C NMR (100.8 MHz, D_2O): 36.98 (CH_2N); 39.64 (CH_2-2′); 61.71 (CH_2-5′); 70.99 (CH-3′); 86.45 (CH-1′); 87.53 (CH-4′); 107.12 (C-5); 142.90 (CH-6); 151.97 (C-2); 165.25 (C-4); 171.68 (HCOO).

IR (ATR): ν = 3485, 3212, 2812, 2392, 1703, 1671, 1617, 1568, 1472, 1435, 1383, 1334, 1274, 1068, 762, 728, 575 cm^{-1}.

HRMS (ESI$^+$): *m/z* calcd for $C_{10}H_{15}O_5N_3Na$ [M + Na$^+$] 280.09039; found: 280.09020.

5-(*N*-Trifluoroacetyl)-aminomethyl-2′-deoxyuridine (dU^{tfa})

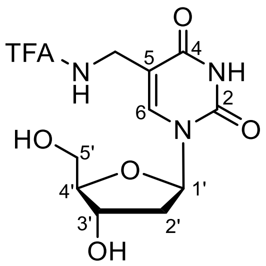

dU^{am} (29 mg, 0.113 mmol) was suspended in MeOH (1.5 mL) and Et_3N (39 µL, 0.57 mmol) together with ethyltrifluoroacetate (65 µL, 0.283 mmol) were added. The reaction was stirred at 23 °C for 16 hours, after which the mixture was evaporated. The residue was purified by column chromatography (20% MeOH in DCM). The product (34 mg, 85%) was obtained as a white solid.

^{1}H NMR (401.0 MHz, CD_3OD): 2.21 (ddd, 1H, J_{gem} = 13.7, $J_{2'b,1'}$ = 7.0, $J_{2'b,3'}$ = 6.0, H-2′b); 2.30 (ddd, 1H, J_{gem} = 13.7, $J_{2'a,1'}$ = 6.2, $J_{2'a,3'}$ = 3.5, H-2′a); 3.74 (dd, 1H, J_{gem} = 12.1, $J_{5'b,4'}$ = 3.9, H-5′b); 3.79 (dd, 1H, J_{gem} = 12.1, $J_{5'a,4'}$ = 3.4, H-5′a); 3.93 (ddd, 1H, $J_{4',5'}$ = 3.9, 3.4, $J_{4',3'}$ = 3.5, H-4′); 4.15 (s, 2H, CH_2N); 4.40 (dt, 1H, $J_{3',2'}$ = 6.0, 3.5, $J_{3',4'}$ = 3.5, H-3′); 6.28 (dd, 1H, $J_{1',2'}$ = 7.0, 6.2, H-1′); 8.05 (s, 1H, H-6).

^{13}C NMR (100.8 MHz, CD_3OD): 37.51 (CH_2N); 41.35 (CH_2-2′); 62.91 (CH_2-5′); 72.30 (CH-3′); 86.62 (CH-1′); 89.05 (CH-4′); 110.49 (C-5); 117.39 (q, $J_{C,F}$ = 286.7, **C**F_3CO); 141.20 (CH-6); 152.08 (C-2); 159.01 (q, $J_{C,F}$ = 37.1, CF_3**C**O); 164.97 (C-4).

IR (ATR): ν = 3461, 3321, 2956, 2566, 2459, 2393, 1725, 1688, 1651, 1470, 1181, 1152, 1098, 1048, 939, 755, 540 cm^{-1}.

HRMS (ESI$^+$): *m/z* calcd for $C_{12}H_{14}O_6N_3F_3Na$ [M + Na$^+$] 376.07269; found: 376.07282.

5-(*N*-Methyl)-aminomethyl-2′-deoxyuridine[6] (dU^{mm})

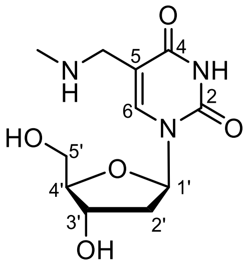

The compound was synthesized according to published procedure.[6] The crude bromo-compound **1** (20 mL, ~ 3.0 mmol) was dissolved in MeCN (20 mL). The mixture was cooled to 0 °C and acetonitrile saturated with $MeNH_2$ (24 mL) was added dropwise over 6 min. The mixture was stirred at 0 °C for 15 minutes and then evaporated. The solid residue was dissolved in MeOH (20 mL) and aqueous ammonia (20 mL), and stirred for 2 hours at 23 °C. The mixture was evaporated and separated by RP-FLC (10 to 100% MeOH in H_2O, C18). Fractions containing product were combined, evaporated and re-purified using FLC (20% to 80% H_2O in MeCN, diol column, hilic mode). Pure product (200 mg, 25% after 2 steps) was obtained as a colourless hydroscopic solid.

^{1}H NMR (401.0 MHz, D_2O): 2.36 (dt, 1H, J_{gem} = 14.2, $J_{2'b,1'}$ = $J_{2'b,3'}$ = 6.6, H-2′b); 2.44 (ddd, 1H, J_{gem} = 14.2, $J_{2'a,1'}$ = 6.6, $J_{2'a,3'}$ = 4.2, H-2′a); 2.71 (s, 3H, CH_3N); 3.76 (dd, 1H, J_{gem} = 12.6, $J_{5'b,4'}$ = 4.9, H-5′b); 3.85 (dd, 1H, J_{gem} = 12.6, $J_{5'a,4'}$ = 3.3, H-5′a); 3.98 (s, 2H, CH_2N); 4.05 (ddd, 1H, $J_{4',5'}$ = 4.9, 3.3, $J_{4',3'}$ = 4.2, H-4′); 4.45 (dt, 1H, $J_{3',2'}$ = 6.6, 4.2, $J_{3',4'}$ = 4.2, H-3′); 6.25 (t, 1H, $J_{1',2'}$ = 6.6, H-1′); 8.12 (s, 1H, H-6).
^{13}C NMR (100.8 MHz, D_2O): 32.85 (CH_3N); 39.75 (CH_2-2′); 45.82 (CH_2N); 61.68 (CH_2-5′); 70.95 (CH-3′); 86.57 (CH-1′); 87.58 (CH-4′); 105.42 (C-5); 143.97 (CH-6); 151.91 (C-2); 165.24 (C-4).
IR (ATR): ν = 2823, 1684, 1479, 1428, 1282, 1202, 1134, 1092, 1055, 918 cm^{-1}.

HRMS (ESI$^+$): *m/z* calcd for $C_{11}H_{18}O_5N_3$ [M + H$^+$] 272.12410; found: 272.12401.

5-(*N*-Trifluoroacetyl)-(*N*-methyl)-aminomethyl-2′-deoxyuridine[6] (dU$^{mm\text{-}tfa}$)

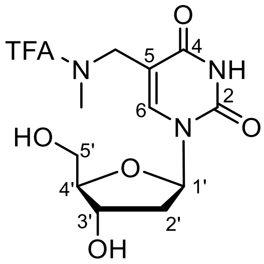

The compound was synthesized according to published procedure.[6] **dU^{mm}** (100 mg, 0.369 mmol) was suspended in MeOH (2.5 mL) and Et_3N (256 µL, 1.84 mmol) together with ethyltrifluoroacetate (440 µL, 3.69 mmol) were added. The reaction was stirred at 23 °C for 16 hours, after which the mixture was evaporated. The residue was dissolved in minimal amount of MeOH and purified by FLC (0 to 10% MeOH in DCM). The pure product **dU$^{mm\text{-}tfa}$** (90 mg, 67%) was obtained as a white powder.

NMR of the major rotamer:

^{1}H NMR (401.0 MHz, CD_3OD): 2.21 (ddd, 1H, J_{gem} = 13.7, $J_{2'b,1'}$ = 7.2, $J_{2'b,3'}$ = 6.2, H-2′b); 2.31 (ddd, 1H, J_{gem} = 13.7, $J_{2'a,1'}$ = 6.2, $J_{2'a,3'}$ = 3.6, H-2′a); 3.24 (q, 3H, $J_{H,F}$ = 1.6, CH_3N); 3.74 (dd, 1H, J_{gem} = 12.0, $J_{5'b,4'}$ = 3.9, H-5′b); 3.79 (dd, 1H, J_{gem} = 12.1, $J_{5'a,4'}$ = 3.5, H-5′a); 3.94 (ddd, 1H, $J_{4',5'}$ = 3.9, 3.5, $J_{4',3'}$ = 3.6, H-4′); 4.26, 4.32 (2 × d, 2 × 1H, J_{gem} = 14.5, CH_2N); 4.39 (dt, 1H, $J_{3',2'}$ = 6.2, 3.6, $J_{3',4'}$ = 3.6, H-3′); 6.27 (dd, 1H, $J_{1',2'}$ = 7.2, 6.2, H-1′); 8.08 (s, 1H, H-6).

^{13}C NMR (100.8 MHz, CD_3OD): 36.36 (q, $J_{C,F}$ = 3.8, CH_3N); 41.39 (CH_2-2′); 47.15 (CH_2N); 62.96 (CH_2-5′); 72.34 (CH-3′); 86.73 (CH-1′); 89.12 (CH-4′); 109.56 (C-5); 117.87 (q, $J_{C,F}$ = 287.0, **C**F_3CO); 142.57 (CH-6); 152.01 (C-2); 158.43 (q, $J_{C,F}$ = 35.6, CF_3**C**O); 165.31 (C-4).

IR (ATR): ν = 2954, 1736, 1691, 1455, 1411, 1367, 1326, 1227, 1199, 1158, 1100, 1043, 1023, 947, 918, 861, 811, 780, 732, 648, 605, 559, 529 cm^{-1}.

HRMS (ESI$^+$): *m/z* calcd for $C_{13}H_{17}F_3N_3O_6$ [M + H$^+$] 368.10640; found: 368.10649.

5-(*N,N*-Dimethyl)-aminomethyl-2′-deoxyuridine[7] formate (dU^{dm})

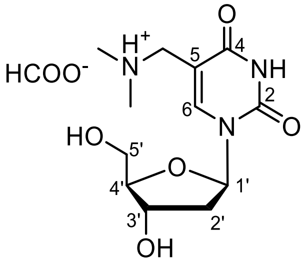

The crude bromo-compound **1** (20 mL, ~ 3.0 mmol) was dissolved in MeCN (20 mL). The mixture was cooled to -20 °C and acetonitrile saturated with Me_2NH (2.5 mL) was added dropwise over 5 min. The mixture was stirred at -20 °C for 1 hour and then another portion of acetonitrile saturated with Me_2NH (2.5 mL) was added and the reaction was stirred for additional 30 min at -20 °C. The reaction was evaporated, the residue dissolved in MeOH (20 mL) and aqueous ammonia (10 mL) and stirred for 2 hours at 23 °C. The reaction was again evaporated and separated by FLC (10% to 80% H_2O in MeCN, diol column, hilic mode). Fractions containing product were combined, evaporated and re-purified using FLC (10% H_2O in MeCN with 2% Et_3N, hilic mode). The crude product was finally purified by HPLC (100% H_2O containing 0.1 % formic acid, Kinetex EVO C18) providing pure compound **dU^{dm}** (298 mg, 30% after 2 steps) in a salt form with formic acid as a white hydroscopic solid.

^{1}H NMR (401.0 MHz, D_2O): 2.35 (dt, 1H, J_{gem} = 14.2, $J_{2'b,1'}$ = $J_{2'b,3'}$ = 6.5, H-2′b); 2.45 (ddd, 1H, J_{gem} = 14.2, $J_{2'a,1'}$ = 6.5, $J_{2'a,3'}$ = 4.3, H-2′a); 2.86 (s, 6H, $(CH_3)_2N$); 3.75 (dd, 1H, J_{gem} = 12.6, $J_{5'b,4'}$ = 4.7, H-5′b); 3.85 (dd, 1H, J_{gem} = 12.6, $J_{5'a,4'}$ = 3.3, H-5′a); 4.00 – 4.13 (m, 3H, H-4′, CH_2N); 4.45 (dt, 1H, $J_{3',2'}$ = 6.5, 4.3, $J_{3',4'}$ = 4.3, H-3′); 6.24 (t, 1H, $J_{1',2'}$ = 6.5, H-1′); 8.18 (s, 1H, H-6); 8.43 (s, 1H, HCOO).

^{13}C NMR (100.8 MHz, D_2O): 39.86 (CH_2-2′); 42.94 ($(CH_3N)_2$); 54.48 (CH_2N); 61.55 (CH_2-5′); 70.85 (CH-3′); 86.73 (CH-1′); 87.64 (CH-4′); 104.38 (C-5); 145.06 (CH-6); 151.82 (C-2); 165.26 (C-4); 171.62 (HCOO).

IR (ATR): ν = 3019, 2945, 2819, 1684, 1581, 1478, 1382, 1350, 1283, 1093, 1056, 942, 763 cm^{-1}.
HRMS (ESI$^+$): *m/z* calcd for $C_{12}H_{20}O_5N_3$ [M + H$^+$] 286.13975; found: 286.13954.

5-Cyanomethyl-2′-deoxyuridine[4] (dU^{cm})

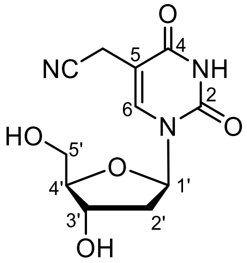

The crude bromo-compound **1** (20 mL, ~ 3.0 mmol) was dissolved in DMF (20 mL). Powdered KCN (780 mg, 12.0 mmol) was added and the reaction was stirred at 50 °C for 16 hours. DMF was evaporated and the residue coevaporated with toluene (2 × 100 mL). After FLC (0 to 10% *i*PrOH in $CHCl_3$), the partially pure protected product was dissolved in MeOH (5 mL) and aqueous ammonia (5 mL). The mixture was stirred at 23 °C for 2 hours, evaporated and separated by HPLC (5 to 20% MeOH in H_2O, Kinetex EVO C18). The pure product **dU^{cm}** (91 mg, 11% after 2 steps) was obtained as a white powder.

^{1}H NMR (401.0 MHz, D_2O): 2.35 (dt, 1H, J_{gem} = 14.2, $J_{2'b,1'}$ = $J_{2'b,3'}$ = 6.6, H-2′b); 2.42 (ddd, 1H, J_{gem} = 14.2, $J_{2'a,1'}$ = 6.6, $J_{2'a,3'}$ = 4.3, H-2′b); 3.56 (d, 2H, $J_{CH2,6}$ = 0.9, CH_2CN); 3.76 (dd, 1H, J_{gem} = 12.5, $J_{5'b,4'}$ = 4.8, H-5′b); 3.85 (dd, 1H, J_{gem} = 12.5, $J_{5'a,4'}$ = 3.4, H-5′a); 4.04 (ddd, 1H, $J_{4',5'}$ = 4.8, 3.4, $J_{4',3'}$ = 4.3, H-4′); 4.46 (dt, 1H, $J_{3',2'}$ = 6.6, 4.3, $J_{3',4'}$ = 4.3, H-3′); 6.28 (t, 1H, $J_{1',2'}$ = 6.6, H-1′); 7.95 (t, 1H, $J_{6,CH2}$ = 0.9, H-6).

^{13}C NMR (100.8 MHz, D_2O): 116.19 (**C**H_2CN); 39.64 (CH_2-2′); 61.68 (CH_2-5′); 71.01 (CH-3′); 86.30 (CH-1′); 87.43 (CH-4′); 105.94 (C-5), 118.87 (CN); 140.45 (CH-6); 152.57 (C-2); 165.63 (C-4).

IR (ATR): ν = 3437, 3190, 3062, 3011, 2913, 2256, 2026, 1703, 1682, 1471, 1278, 1203, 1106, 1085, 1054, 819, 751, 589, 545 cm^{-1}.

HRMS (ESI$^+$): *m/z* calcd for $C_{11}H_{13}O_5N_3Na$ [M + Na$^+$] 290.07474; found: 290.07467.

5-Bromomethyl-3′,5′-bis-*O*-(*tert*-butyldiphenylsilyl)-2′-deoxyuridine[3] (2)

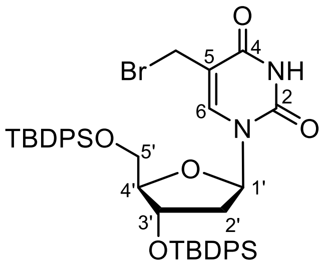

The compound was synthesized according to published procedure.[3] After the reaction was finished, the reaction was hot-filtered, evaporated and used immediately in the next step. The estimated yield (based on TLC) was 70%.

5-(Acetylthio)methyl-3′,5′-bis-*O*-(*tert*-butyldiphenylsilyl)-2′-deoxyuridine (3)

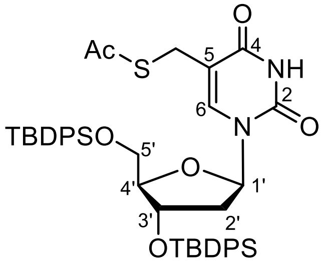

The crude TBDPS-protected bromo-compound **2** (20 mL, ~ 3.0 mmol) was dissolved in DMF (25 mL). The mixture was heated to 75 °C and potassium thioacetate (1.83 g, 16 mmol) was added in one portion. The reaction was stirred at 23 °C for 1.5 hour, followed by evaporation. The residue was suspended in H_2O (50 mL) and DCM (200 mL). The organic layer was washed with H_2O (3 × 50 mL) and then evaporated. The residue was separated by FLC (0 to 60% EtOAc in cHex), followed by another FLC (0 to 5% MeOH in DCM). The crude product **3** (1.54 g) was obtained as a light yellow foam and was used in the next step without further purification.

HRMS (ESI^+): *m/z* calcd for $C_{44}H_{53}N_2O_6SSi_2$ [M + H^+] 793.31574; found: 793.31604

5-(Acetylthio)methyl-2′-deoxyuridine[8] (dU^{asm})

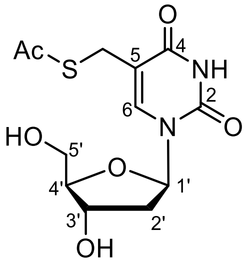

Crude starting compound **3** (1.0 g, 1.26 mmol) was dissolved in THF (10 mL), cooled to 0 °C and to this, AcOH (360 µL, 6.3 mmol) and 1 M TBAF in THF (3.03 mL, 3.03 mmol) were added. After 10 min, the reaction was warmed to 23 °C and stirred for 4 hours. The mixture was then evaporated, dissolved in minimal amount of DCM and purified by FLC (0 to 10% MeOH in DCM) and then RP-FLC (10 to 100% MeOH in H_2O, C18). The pure product **dU^{asm}** (285 mg, 46% after 2 steps) was obtained as a white powder.

^{1}H NMR (401.0 MHz, D_2O): 2.33 (dt, 1H, J_{gem} = 14.2, $J_{2'b,1'}$ = $J_{2'b,3'}$ = 6.6, H-2′b); 2.36 (s, 3H, CH_3); 2.40 (ddd, 1H, J_{gem} = 14.2, $J_{2'a,1'}$ = 6.6, $J_{2'a,3'}$ = 4.4, H-2′a); 3.76 – 3.89 (m, 4H, H-5′, CH_2S); 4.04 (ddd, 1H, $J_{4',5'}$ = 4.9, 3.7, $J_{4',3'}$ = 4.4, H-4′); 4.48 (dt, 1H, $J_{3',2'}$ = 6.6, 4.4, $J_{3',4'}$ = 4.4, H-3′); 6.28 (t, 1H, $J_{1',2'}$ = 6.6, H-1′); 7.96 (s, 1H, H-6).

^{13}C NMR (100.8 MHz, D_2O): 26.27 (CH_2S); 30.56 (CH_3); 39.52 (CH_2-2′); 61.87 (CH_2-5′); 71.13 (CH-3′); 86.03 (CH-1′); 87.42 (CH-4′); 111.67 (C-5); 140.49 (CH-6); 152.13 (C-2); 165.73 (C-4); 201.21 (**C**OCH_3).

IR (ATR): ν = 3399, 3244, 3110, 3056, 2947, 1722, 1687, 1645, 1475, 1277, 1099, 1055, 1031, 953, 754, 619 cm^{-1}.

HRMS (ESI$^+$): *m/z* calcd for $C_{12}H_{16}O_6N_2SNa$ [M + Na$^+$] 339.06213; found: 339.06220.

5-{*N*-[1*R*,3-Bis(methoxycarbonyl)prop-1-yl]-aminomethyl}-3′,5′-di-*O*-acetyl-2′-deoxyuridine (4)

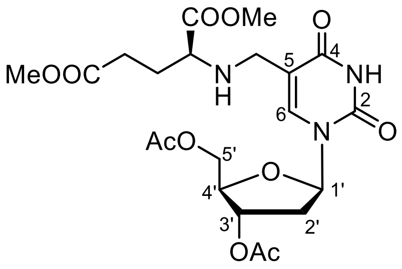

The crude bromo-compound **1** (20 mL, ~ 3.0 mmol) was dissolved in DCM (15 mL). Dimethylglutamate hydrochloride (1.27 g, 6 mmol) was added, followed by dropwise addtition of Et_3N (1.7 mL, 12 mmol) over 1 min. The reaction was stirred at 23 °C for 3 hours and then evaporated. Purification with RP-FLC (10 to 100% MeOH in H_2O), followed by FLC (0 to 10% MeOH in EtOAc) afforded product **4** (640 mg, 43%) as a white powder.

^{1}H NMR (401.0 MHz, $CDCl_3$): 1.86 – 2.07 (m, 2H, H-3-Glu); 2.09, 2.11 (2 × s, 2 × 3H, CH_3CO); 2.26 (ddd, 1H, J_{gem} = 14.6, $J_{2'b,1'}$ = 8.7, $J_{2'b,3'}$ = 6.6, H-2′b); 2.35 – 2.50 (m, 3H, H-2′a, H-4-Glu); 3.33 (dd, 1H, $J_{2,3}$ = 7.3, 6.3, H-2-Glu); 3.43, 3.57 (2 × d, 2 × 1H, J_{gem} = 14.3, CH_2N); 3.63 (s, 3H, CH_3OOC-5-Glu); 3.70 (s, 3H, CH_3OOC-1-Glu); 4.23 (ddd, 1H, $J_{4',5'}$ = 4.9, 3.5, $J_{4',3'}$ = 2.3, H-4′); 4.30 (dd, 1H, J_{gem} = 12.0, $J_{5'b,4'}$ = 3.5, H-5′b); 4.41 (dd, 1H, J_{gem} = 12.0, $J_{5'a,4'}$ = 4.9, H-5′a); 5.20 (dt, 1H, $J_{3',2'}$ = 6.6, 2.5, $J_{3',4'}$ = 2.5, H-3′); 6.28 (dd, 1H, $J_{1',2'}$ = 8.7, 5.6, H-1′); 7.55 (s, 1H, H-6).

^{13}C NMR (100.8 MHz, $CDCl_3$): 20.94, 21.00 (**C**H_3CO); 27.83 (CH_2-3-Glu); 30.30 (CH_2-4-Glu); 37.46 (CH_2-2′); 44.32 (CH_2N); 51.78 (**C**H_3OOC-5-Glu); 52.27 (**C**H_3OOC-1-Glu); 60.07 (CH-2-Glu); 63.92 (CH_2-5′); 74.41 (CH-3′); 82.51 (CH-4′); 85.38 (CH-1′); 112.50 (C-5); 137.19 (CH-6); 150.21 (C-2); 163.19 (C-4); 170.48, 170.51 (CH_3**C**O); 173.38 (CH_3OO**C**-5-Glu); 174.32 (CH_3OO**C**-1-Glu).

HRMS (ESI$^+$): *m/z* calcd for $C_{21}H_{30}N_3O_{11}$ [M + H$^+$] 500.18749; found: 500.18762.

5-{*N*-(*Tert*-butoxycarbonyl)-*N*-[1*R*,3-bis(methoxycarbonyl)prop-1-yl]-aminomethyl}-3′,5′-di-*O*-acetyl-2′-deoxyuridine (5)

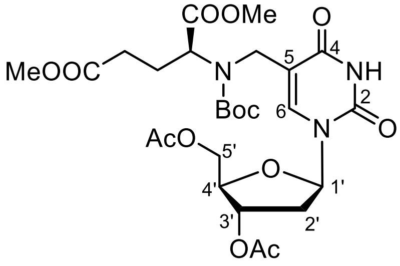

Starting material **4** (750 mg, 1.5 mmol) was dissolved in MeOH (7.5 mL) and Boc_2O (390.5 mg, 1.79 mmol) was added in one portion. The reaction was stirred at 23 °C for 2 days. After evaporation, the mixture was purified by FLC (0 to 40% MeOH in EtOAc), affording the product **5** (718 mg, 80%) as a white powder.

NMR of the major rotamer:

^{1}H NMR (401.0 MHz, CD_3OD): 1.40 (s, 9H, $(CH_3)_3C$); 2.10, 2.17 (2 × s, 2 × 3H, CH_3CO); 2.21 (m, 1H, H-3b-Glu); 2.27 – 2.49 (m, 5H, H-2′, H-3a,4-Glu); 3.66, 3.68 (2 × s, 2 × 3H, CH_3O); 4.08, 4.20 (2 × d, 2 × 1H, J_{gem} = 14.9, CH_2N); 4.21 – 4.41 (m, 4H, H-2-Glu, H-4′,5′); 5.28 (bm, 1H, H-3′); 6.30 (bm, 1H, H-1′); 7.90 (s, 1H, H-6).
^{13}C NMR (100.8 MHz, CD_3OD): 20.79, 20.91 (**C**H_3CO); 26.94 (CH_2-3-Glu); 28.54 ((**C**$H_3)_3C$); 32.49 (CH_2-4-Glu); 37.86 (CH_2-2′); 46.09 (CH_2N); 52.21, 52.75 (CH_3O); 62.07 (CH-2-Glu); 65.16 (CH_2-5′); 75.79 (CH-3′); 82.30 ($(CH_3)_3$**C**); 83.47 (CH-4′); 86.12 (CH-1′); 112.60 (C-5); 140.86 (CH-6); 151.99 (C-2); 156.63 (OCON); 165.57 (C-4); 172.05, 172.50 (CH_3CO); 173.34 (C-1-Glu); 175.15 (C-5-Glu).
HRMS (ESI$^+$): *m/z* calcd for $C_{26}H_{38}N_3O_{13}$ [M + H$^+$] 600.23991; found: 600.24001.

5-[*N*-(*Tert*-butoxycarbonyl)-*N*-(1*R*,3-dicarboxyprop-1-yl)-aminomethyl]-2′-deoxyuridine (6)

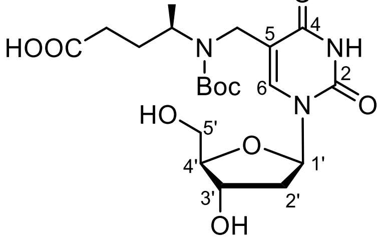

Starting compound **5** (400 mg, 0.67 mmol) was dissolved in mixture of water (5 mL) and MeOH (2.5 mL) and treated with K_2CO_3 (553 mg, 4.0 mmol). The mixture was stirred for 24 hours at 23 °C. Then, the reaction was evaporated, redissolved in H_2O (5 mL) and stirred for another 24 hours. The mixture was treated with diluted formic acid until pH = 5, evaporated and separated using RP-FLC (10 to 100% MeOH in H_2O, C18). The product **6** (160 mg, 49%) was acquired as a white amorphous solid. The separation also purifies partially deprotected starting material, which was subjected to another round of deprotection.

NMR of the major rotamer:

^{1}H NMR (401.0 MHz, CD_3OD): 1.45 (s, 9H, $(CH_3)_3C$); 2.05 – 2.41 (m, 6H, H-2′, H-3,4-Glu); 3.71 (bdd, 1H, J_{gem} = 12.0, $J_{5'b,4'}$ = 4.5, H-5′b); 3.77 (dd, 1H, J_{gem} = 12.0, $J_{5'a,4'}$ = 3.9, H-5′a); 3.91 (ddd, 1H, $J_{4',5'}$ = 4.5, 3.9, $J_{4',3'}$ = 3.3, H-4′); 4.09 (bd, 1H, J_{gem} = 15.7, CH$_a$**H$_b$**N); 4.20 (bm, 1H, H-2-Glu); 4.23 (bd, 1H, J_{gem} = 15.7, C**H$_a$**H$_b$N); 4.37 (bm, 1H, H-3′); 6.30 (t, 1H, $J_{1',2'}$ = 6.8, H-1′); 8.00 (s, 1H, H-6).

^{13}C NMR (100.8 MHz, CD_3OD): 27.20 (CH_2-3-Glu); 28.60 ((**C**H_3)$_3$C); 32.10 (CH_2-4-Glu); 40.90 (CH_2-2′); 45.44 (CH_2N); 62.07 (CH-2-Glu); 63.20 (CH_2-5′); 72.34 (CH-3′); 82.37 ((CH_3)$_3$**C**); 86.21 (CH-1′); 88.89 (CH-4′); 112.02 (C-5); 140.82 (CH-6); 152.19 (C-2); 157.23 (OCON); 165.54 (C-4); 175.16 (C-1-Glu); 177.10 (C-5-Glu).

IR (ATR): ν = 2978, 1672, 1458, 1410, 1368, 1273, 1160, 1093, 1053, 858, 775, 603, 561 cm^{-1}.

HRMS (ESI$^+$): *m/z* calcd for $C_{20}H_{30}N_3O_{11}$ [M + H$^+$] 488.18749; found: 488.18764.

5-[*N*-(1*R*,3-Dicarboxyprop-1-yl)-aminomethyl]-2′-deoxyuridine (dU^{glu})

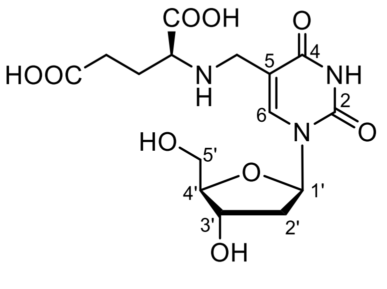

Starting material **6** (160 mg, 0.33 mmol) was suspended in DCM (36 mL) and TFA (4 mL) was added slowly. After the addition of TFA, the compound dissolved in the mixture. The reaction was stirred for 35 min at 23 °C, after which it was combined with toluene (100 mL) and the whole

mixture was evaporated. Residual solids were dissolved in water (10 mL) and aqueous ammonia (1 mL) was added. The mixture was evaporated and the crude product was purified on HPLC (0 to 60% of MeOH in H_2O containing 0.1% formic acid, Waters X-Bridge Shield RP18). The purified product **dU^{glu}** (100 mg, 79%) was acquired as a colourless amorphous solid.

^{1}H NMR (401.0 MHz, CD_3OD): 2.08 – 2.23 (m, 2H, H-3-Glu); 2.28 (dt, 1H, J_{gem} = 13.8, $J_{2'b,1'}$ = $J_{2'b,3'}$ = 6.4, H-2′b); 2.38 (ddd, 1H, J_{gem} = 13.8, $J_{2'a,1'}$ = 6.4, $J_{2'a,3'}$ = 3.9, H-2′a); 2.50 – 2.62 (m, 2H, H-4-Glu); 3.72 (dd, 1H, $J_{2,3}$ = 7.0, 5.6, H-2-Glu); 3.77 (dd, 1H, J_{gem} = 12.3, $J_{5'b,4'}$ = 4.1, H-5′b); 3.85 (dd, 1H, J_{gem} = 12.3, $J_{5'a,4'}$ = 3.2, H-5′a); 3.99 (ddd, 1H, $J_{4',5'}$ = 4.1, 3.2, $J_{4',3'}$ = 3.9, H-4′); 3.99, 4.03 (2 × d, 2 × 1H, J_{gem} = 13.6, CH_2N); 4.43 (dt, 1H, $J_{3',2'}$ = 6.4, 3.9, $J_{3',4'}$ = 3.9, H-3′); 6.24 (t, 1H, $J_{1',2'}$ = 6.4, H-1′); 8.21 (s, 1H, H-6).
^{13}C NMR (100.8 MHz, CD_3OD): 26.10 (CH_2-3-Glu); 30.97 (CH_2-4-Glu); 41.21 (CH_2-2′); 44.51 (CH_2N); 61.79 (CH-2-Glu); 62.37 (CH_2-5′); 71.69 (CH-3′); 86.99 (CH-1′); 88.75 (CH-4′); 105.71 (C-5); 144.22 (CH-6); 151.91 (C-2); 165.26 (C-4); 172.41 (C-1-Glu); 176.96 (C-5-Glu).
IR (ATR): ν 1738, 1709, 1667, 1602, 1472, 1392, 1301, 1186, 1176, 1131, 1058, 1017, 759 cm^{-1}.
HRMS (ESI$^+$): *m/z* calcd for $C_{15}H_{22}O_9N_3$ [M + H$^+$] 388.13506; found: 388.13527.

5-{*N*-[4-(*Tert*-butoxycarbamido)-but-1-yl]-aminomethyl}-3′,5′-di-*O*-acetyl-2′-deoxyuridine (7)

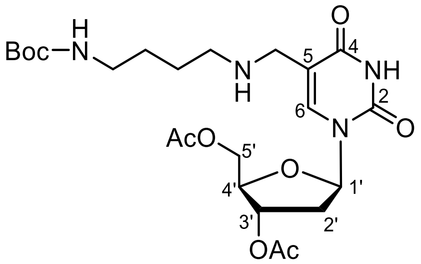

The crude bromo-compound **1** (10 mL, ~ 1.5 mmol) was dissolved in DCM (7.5 mL) and treated with Et_3N (0.83 mL, 6 mmol) and *N*-Boc-putrescine (0.58 mL, 3 mmol). The reaction was stirred at 23 °C and monitored by TLC using ninhydrin and anisaldehyde stains. After 3 hours, the reaction was evaporated and separated by FLC (0 to 30% DCM in MeOH). Purification provided the product **7** (450 mg, 58%) as a slightly yellow foam.

^1H NMR (401.0 MHz, CD_3OD): 1.42 (s, 9H, $(CH_3)_3C$); 1.44 – 1.59 (m, 4H, $NHCH_2C\mathbf{H_2}C\mathbf{H_2}CH_2NH_2$); 2.10, 2.11 (2 × s, 2 × 3H, CH_3CO); 2.39 (ddd, 1H, J_{gem} = 14.4, $J_{2'b,1'}$ = 8.4, $J_{2'b,3'}$ = 6.4, H-2′b); 2.45 (ddd, 1H, J_{gem} = 14.4, $J_{2'a,1'}$ = 6.1, $J_{2'a,3'}$ = 2.6, H-2′a); 2.58 – 2.63 (m, 2H, $NHC\mathbf{H_2}CH_2CH_2CH_2NHBoc$); 3.02 – 3.07 ($NHCH_2CH_2CH_2C\mathbf{H_2}NHBoc$); 3.47, 3.51 (2 × dd, 2 × 1H, J_{gem} = 13.8, $J_{CH2,6}$ = 0.8, CH_2N); 4.26 (ddd, 1H, $J_{4',5'}$ = 5.2, 3.8, $J_{4',3'}$ = 2.6, H-4′); 4.29 (dd, 1H, J_{gem} = 11.7, $J_{5'b,4'}$ = 3.8, H-5′b); 4.41 (dd, 1H, J_{gem} = 11.7, $J_{5'a,4'}$ = 5.2, H-5′a); 5.26 (dt, 1H, $J_{3',2'}$ = 6.4, 2.6, $J_{3',4'}$ = 2.6, H-3′); 6.25 (dd, 1H, $J_{1',2'}$ = 8.4, 6.1, H-1′); 7.66 (s, 1H, H-6).
^{13}C NMR (100.8 MHz, CD_3OD): 20.80, 20.86 ($\mathbf{C}H_3CO$); 27.51, 28.64 ($NHCH_2\mathbf{C}H_2\mathbf{C}H_2CH_2NHBoc$); 28.79 ($(\mathbf{C}H_3)_3C$); 37.72 ($CH_2$-2′); 41.07 ($NHCH_2CH_2CH_2\mathbf{C}H_2NHBoc$); 46.53 ($CH_2N$); 49.41 ($NH\mathbf{C}H_2CH_2CH_2CH_2NH_2$); 65.04 ($CH_2$-5′); 75.78 (CH-3′); 79.84 ($(CH_3)_3\mathbf{C}$); 83.65 (CH-4′); 86.76 (CH-1′); 112.96 (C-5); 139.39 (CH-6); 152.14 (C-2); 158.52 (OCONH); 165.76 (C-4); 172.11, 172.34 ($CH_3\mathbf{C}O$).

IR (ATR): ν = 3365, 2933, 1740, 1681, 1519, 1455, 1365, 1226, 1196, 1167, 1100, 1022, 864, 780, 760, 603, 572, 552 cm^{-1}.

HRMS (ESI^+): *m/z* calcd for $C_{23}H_{37}O_9N_4$ $[M + H^+]$ 513.25551; found: 513.25527.

5-[*N*-(4-Aminobut-1-yl)-aminomethyl]-2′-deoxyuridine[6] (dU^{put})

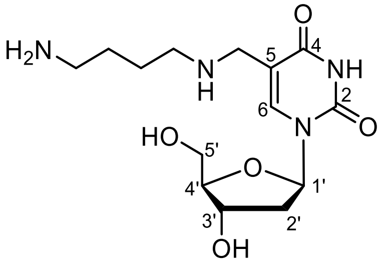

Compound **7** (305 mg, 0.595 mmol) was dissolved in MeOH (6 mL) and cooled to 0 °C. AcCl (233 µL, 3.27 mmol) was then added dropwise over 1 min. The reaction was taken out of the cooling bath, stirred at 23 °C and monitored by TLC. After 3 hours, more AcCl (100 µL, 1.4 mmol) was added, followed by stirring for 2 hours. Finally, the last portion of AcCl (100 µL, 1.4 mmol) was added, the reaction stirred for 1 hour, evaporated and further dried on high vacuum. The crude product was purified by HPLC (0 to 60% MeOH in H_2O containing

0.1% TFA, 15 mL/min, Waters X-Bridge Shield RP18). Lyophilization of the appropriate fractions gave the product **dU^put^** (126 mg, 53%) as an off-white amorphous solid.

^{1}H NMR (401.0 MHz, D_2O): 1.65 – 1.80 (m, 4H, $NHCH_2C\mathbf{H_2}C\mathbf{H_2}CH_2NH_2$); 2.31 (dt, 1H, J_{gem} = 14.2, $J_{2'b,1'}$ = $J_{2'b,3'}$ = 6.6, H-2′b); 2.40 (ddd, 1H, J_{gem} = 14.2, $J_{2'a,1'}$ = 6.6, $J_{2'a,3'}$ = 4.2, H-2′a); 2.96 – 3.02 (m, 2H, $NHCH_2CH_2CH_2C\mathbf{H_2}NH_2$); 3.05 – 3.11 ($NHC\mathbf{H_2}CH_2CH_2CH_2NH_2$); 3.72 (dd, 1H, J_{gem} = 12.6, $J_{5'b,4'}$ = 4.9, H-5′b); 3.81 (dd, 1H, J_{gem} = 12.6, $J_{5'a,4'}$ = 3.3, H-5′a); 3.95, 3.99 (2 × d, 2 × 1H, J_{gem} = 13,8, CH_2N); 4.02 (ddd, 1H, $J_{4',5'}$ = 4.9, 3.4, $J_{4',3'}$ = 4.2, H-4′); 4.41 (dt, 1H, $J_{3',2'}$ = 6.6, 4.2, $J_{3',4'}$ = 4.2, H-3′); 6.21 (t, 1H, $J_{1',2'}$ = 6.6, H-1′); 8.09 (s, 1H, H-6).

^{13}C NMR (100.8 MHz, D_2O): 23.23 ($NHCH_2\mathbf{C}H_2CH_2CH_2NH_2$); 24.58 ($NHCH_2CH_2\mathbf{C}H_2CH_2NH_2$); 39.43 ($NHCH_2CH_2CH_2\mathbf{C}H_2NH_2$); 39.70 ($CH_2$-2′); 44.28 ($CH_2N$); 47.06 ($NH\mathbf{C}H_2CH_2CH_2CH_2NH_2$); 61.68 ($CH_2$-5′); 70.95 (CH-3′); 86.55 (CH-1′); 87.56 (CH-4′); 105.45 (C-5); 144.06 (CH-6); 151.83 (C-2); 165.19 (C-4).

IR (ATR): ν = 2953, 1688, 1477, 1284, 1201, 1092, 1055 cm^{-1}.

HRMS (ESI^{+}): *m/z* calcd for $C_{14}H_{25}O_5N_4$ [M + H^{+}] 329.18195; found: 329.18203.

5-[*N*, *N′*-Bis(trifluoroacetyl)-*N*-(4-aminobut-1-yl)-aminomethyl]-2′-deoxyuridine[6] (dU^put-tfa^)

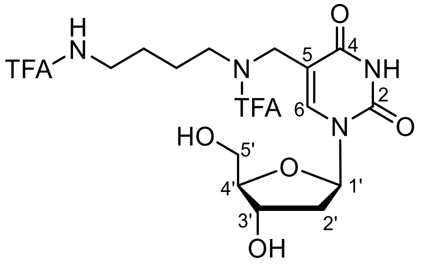

The compound was synthesized according to published procedure.[6] **dU^put^** (129 mg, 0.32 mmol) was suspended in MeOH (2.1 mL) and ethyl-trifluoroacetate (457 mg, 3.2 mmol) was added, followed by Et_3N (163 mg, 1.6 mmol). The reaction was stirred at 23 °C for 2 days and monitored by TLC using ninhydrin stain. The mixture was then evaporated and further dried on high vacuum. Purification by FLC (0 to 100% MeOH in DCM) provided the product **dU^put-tfa^** (106 mg, 63%) as a yellow amorphous solid.

NMR of the major rotamer:

^{1}H NMR (401.0 MHz, CD_3OD): 1.49 – 1.78 (m, 4H, $NCH_2C\mathbf{H_2}C\mathbf{H_2}CH_2NH$); 2.20 (ddd, 1H, J_{gem} = 13.6, $J_{2'b,1'}$ = 7.3, $J_{2'b,3'}$ = 6.1, H-2′b); 2.31 (ddd, 1H, J_{gem} = 14.6, $J_{2'a,1'}$ = 6.2, $J_{2'a,3'}$ = 3.5, H-2′a); 3.27 – 3.34 (m, 2H, $NCH_2CH_2CH_2C\mathbf{H_2}NH$); 3.56 – 3.72 ($NC\mathbf{H_2}CH_2CH_2CH_2NH$); 3.74 (dd, 1H, J_{gem} = 12.1, $J_{5'b,4'}$ = 4.1, H-5′b); 3.78 (dd, 1H, J_{gem} = 12.1, $J_{5'a,4'}$ = 3.5, H-5′a); 3.94 (dt, 1H, $J_{4',5'}$ = 4.1, 3.5, $J_{4',3'}$ = 3.5, H-4′); 4.27, 4.31 (2 × d, 2 × 1H, J_{gem} = 14.7, CH_2N); 4.40 (dt, 1H, $J_{3',2'}$ = 6.1, 3.5, $J_{3',4'}$ = 3.5, H-3′); 6.22 (t, 1H, $J_{1',2'}$ = 7.3, 6.2, H-1′); 8.10 (s, 1H, H-6).

^{13}C NMR (100.8 MHz, CD_3OD): 26.84, 26.86 ($NCH_2\mathbf{C}H_2\mathbf{C}H_2CH_2NH$); 40.20 ($NCH_2CH_2CH_2\mathbf{C}H_2NH$); 41.37 ($CH_2$-2′); 44.13 ($N\mathbf{C}H_2CH_2CH_2CH_2NH$); 48.90 (q. $J_{C,F}$ = 3.0, CH_2N); 63.03 (CH_2-5′); 72.41 (CH-3′); 86.71 (CH-1′); 89.13 (CH-4′); 109.69 (C-5); 117.54 (q, $J_{C,F}$ = 286.7, $\mathbf{C}F_3CO$); 117.90 (q, $J_{C,F}$ = 287.1, $\mathbf{C}F_3CO$); 142.75 (CH-6); 151.91 (C-2); 158.40 (q, $J_{C,F}$ = 35.7, $CF_3\mathbf{C}O$); 159.02 (q, $J_{C,F}$ = 36.8, $CF_3\mathbf{C}O$); 165.38 (C-4).

HRMS (ESI$^+$): *m/z* calcd for $C_{18}H_{23}O_7N_4F_6$ [M + H$^+$] 521.14654; found: 521.14654.

1.4. Synthesis of nucleoside triphosphates

5-(4,5-Dihydroxypent-1-yn-1-yl)-2′-deoxyuridine-5′-*O*-triphosphate, triethylammonium salt (8)

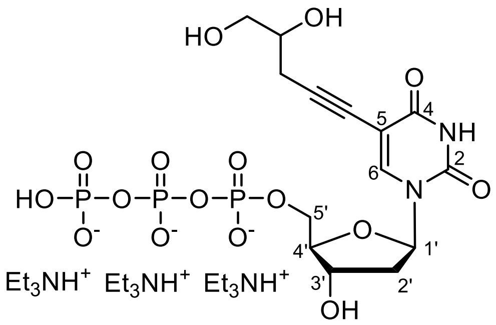

5-iodo-dUTP in triethylammonium salt form (43.2 mg, 48 µmol), $Pd(OAc)_2$ (0.5 mg, 2.4 µmol), TPPTS (6.8 mg, 12 µmol) and CuI (0.9 mg, 4.8 µmol) were put into a flask filled with Ar and suspended in a deggased 2:1 mixture of H_2O and MeCN (0.8 mL). Then, pent-4-yn-1,2-diol[9] (9.6 mg, 96 µmol) and Et_3N (54 µL, 385 µmol) were added and the reaction mixture was stirred at 80 °C for 1 hour. The reaction was diluted with H_2O and evaporated. The residue was suspended in a 1:1 mixture of H_2O and MeOH (2 mL) and filtered through a small C18 silica plug. The filtered

solution was evaporated, residue dissolved in buffer A and injected into HPLC (5 to 30% buffer B in buffer A, Waters X-Bridge Shield RP18). This crude intermediate **8** (25.6 mg) was used in the next step without further purification.

5-(4,5-Dihydroxypent-1-yl)-2′-deoxyuridine-5′-*O*-triphosphate, triethylammonium salt (dU^{dhp}TP)

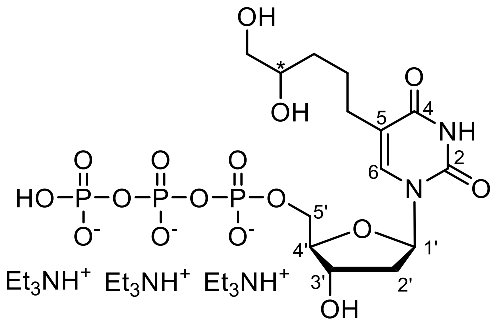

Crude compound **8** (25.6 mg, 29 µmol) was dissolved in MeOH (5 mL) with 5 drops of H_2O. 10% Pd/C (7.7 mg, 7.5 µmol of Pd) was added, followed by attaching a H_2 balloon via septum. The reaction was stirred under H_2 atmosphere at 23 °C for 24 hours. The reaction was diluted with H_2O, filtered through a celite plug, evaporated and redissolved in buffer A. The solution was then injected into HPLC (0 to 60% of buffer B in buffer A, Waters X-Bridge Shield RP18). The pure product **dU^{dhp}TP** (19.6 mg, 46% after 2 steps) was obtained as an amorphous solid.

~ 1:1 mixture of epimers

^{1}H NMR (500.0 MHz, CD_3OD): 1.31 (t, 54H, J_{vic} = 7.3, C**H**$_3$CH$_2$N); 1.33 – 1.45 (m, 2H, H-3″b); 1.49 – 1.66 (m, 4H, H-2″b,3″a); 1.68 – 1.76 (m, 2H, H-2″a); 2.19 – 2.24 (m, 2H, H-2′b); 2.27 – 2.33 (m, 2H, H-2′a); 2.34 – 2.46 (m, 4H, H-1″); 3.18 (q, 36H, J_{vic} = 7.3, CH$_3$C**H**$_2$N); 3.40 – 3.49 (m, 4H, H-5″); 3.66 – 3.73 (m, 2H, H-4″); 4.01 – 4.04 (m, 2H, H-4′); 4.18 – 4.23 (m, 2H, H-5′b); 4.32 – 4.38 (m, 2H, H-5′a); 4.63 – 4.68 (m, 2H, H-3′); 6.32, 6.33 (2 × dd, 2 × 1H, $J_{1',2'}$ = 7.5, 3.2, H-1′); 7.80, 7.81 (2 × t, 2 × 1H, ^4J = 1.0, H-6).
^{13}C NMR (125.7 MHz, CD_3OD): 9.15 (**C**H$_3$CH$_2$N); 25.43, 25.46 (CH$_2$-2″); 27.51, 27.59 (CH$_2$-1″); 33.76, 33.84 (CH$_2$-3″); 40.68, 40.70 (CH$_2$-2′); 47.34 (CH$_3$**C**H$_2$N); 66.72, 66.79 (2 × d, $J_{C,P}$ = 5.9, CH$_2$-5′); 67.49, 67.56 (CH$_2$-5″); 72.04, 72.11 (CH-3′); 72.50, 72.55 (CH-4″); 85.99 (CH-1′); 87.63, 87.67 (2 × d, $J_{C,P}$ = 9.2, CH-4′); 115.99, 116.05 (C-5); 138.31, 138.35 (CH-6); 152.35 (C-2); 166.10 (C-4).

$^{31}P\{^1H\}$ NMR (202.4 MHz, CD_3OD): –22.34 (t, J = 21.4, P_β); –10.11, –10.03 (2 × d, J = 21.4, P_α); –8.93 (d, J = 21.4, P_γ).

HRMS (ESI⁻): *m/z* calcd for $C_{14}H_{24}O_{16}N_2P_3$ [M – H⁺] 569.03442; found: 569.03430.

5-{*N*-[1*R*,3-Bis(methoxycarbonyl)prop-1-yl]-aminomethyl}-2′-deoxyuridine-5′-*O*-triphosphate, triethylammonium salt (dU^glu^TP)

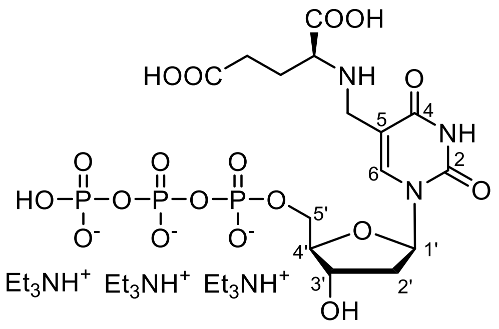

dU^glu^ (25 mg, 0.065 mmol) was dried on high vacuum for 16 hours and then suspended in $PO(OMe)_3$ (0.37 mL), stirred at 23 °C for 10 min and then cooled to 0 °C. $POCl_3$ (7 µL, 0.077 mmol) was added dropwise over 1 min and the reaction was stirred at 0 °C for 5 hours. Then, Bu_3N (61 µL, 0.26 mmol) and chilled 0.5 M solution of $(Bu_3N)_2H_2P_2O_7$ in MeCN (0.65 mL, 0.32 mmol) were added and the reaction was stirred at 0 °C for 30 min and then 15 min at 23 °C. The reaction was finished by adding 1 M TEAB (1 mL) and stirred for 15 min. The mixture was diluted with H_2O, evaporated, diluted again with H_2O (5 mL) and lyophilized for 16 hours. The lyophilizate was dissolved in buffer A and injected into HPLC (0 to 60% of buffer B in buffer A, Waters X-Bridge Shield RP18). The appropriate fractions were collected and evaporated. The crude product was repurified by ion-exchange HPLC (0 to 100% of 800 mM TEAB in H_2O, Sepharose DEAE Fast Flow). The pure triphosphate **dU^glu^TP** (4.0 mg, 7%) was obtained as a hydroscopic amorphous solid.

^1H NMR (500.0 MHz, D_2O): 1.27 (t, 18H, J_{vic} = 7.3, C**H₃**CH₂N); 2.08 – 2.25 (m, 2H, H-3-Glu); 2.36 – 2.46 (m, 2H, H-2′); 2.46 – 2.58 (m, 2H, H-4-Glu); 3.19 (q, 12H, J_{vic} = 7.3, CH₃C**H₂**N); 3.71 (t, 1H, $J_{2,3}$ = 6.1, H-2-Glu); 4.08 (s, 2H, CH₂N); 4.19 – 4.29 (nm, 3H, H-4′,5′); 4.66 (m, 1H, H-3′); 6.28 (t, 1H, $J_{1',2'}$ = 6.5, H-1′); 8.32 (s, 1H, H-6).

^{13}C NMR (125.7 MHz, D_2O): 11.07 (**C**H_3CH_2N); 28.15 (CH_2-3-Glu); 33.45 (CH_2-4-Glu); 42.30 (CH_2-2′); 45.57 (CH_2N); 49.51 (CH_3**C**H_2N); 63.73 (CH-2-Glu); 68.02 (d, $J_{C,P}$ = 5.7, CH_2-5′); 73.40 (CH-3′); 88.72 (d, $J_{C,P}$ = 9.2, CH-4′); 88.85 (CH-1′); 107.85 (C-5); 146.50 (CH-6); 154.18 (C-2); 167.57 (C-4); 175.81 (C-1-Glu); 180.58 (C-5-Glu).

^{31}P NMR (202.4 MHz, D_2O): –22.56 (t, J = 19.5, P_β); –10.75 (d, J = 19.5, P_α); –10.23 (d, J = 19.5, P_γ).

HRMS (ESI$^-$): *m/z* calcd for $C_{15}H_{23}O_{18}N_3P_3$ [M – H^+] 626.01949; found: 626.01907.

5-[*N*-(4-Aminobut-1-yl)-aminomethyl]-2′-deoxyuridine-5′-*O*-triphosphate, triethylammonium salt (dU^{put}TP)

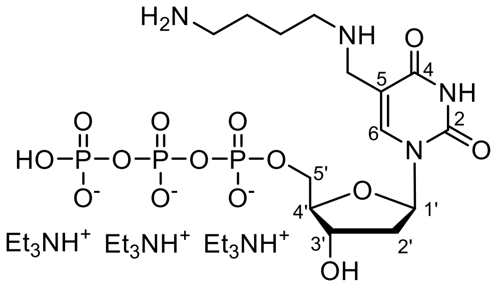

Starting material **dU$^{put-tfa}$** (45 mg, 0.086 mmol) and Proton Sponge (18.5 mg, 0.086 mmol) were dried together on high vacuum for 16 hours. The mixture was suspended in $PO(OMe)_3$ (0.5 mL), stirred at 23 °C for 10 min and then cooled to 0 °C. $POCl_3$ (9 µL, 0.104 mmol) was added dropwise over 1 min and the reaction was stirred at 0 °C for 4.5 hours. Then, Bu_3N (82 µL, 0.35 mmol) and chilled 0.5 M solution of $(Bu_3N)_2H_2P_2O_7$ in MeCN (0.87 mL, 0.42 mmol) were added and the reaction was stirred at 0 °C for 30 min and then 15 min at 23 °C. The reaction was finished by adding 1 M TEAB (1 mL) and stirred for 15 min. The mixture was diluted with H_2O, evaporated, diluted again with H_2O (5 mL) and lyophilized for 16 hours. The lyophilizate was dissolved in buffer A and injected into HPLC (0 to 60% of buffer B in buffer A, Waters X-Bridge Shield RP18). The appropriate fractions were collected and evaporated. Half of this crude TFA-protected product was dissolved in 50 mM TEAB in H_2O (2.5 mL) and aqueous ammonia (2.5 mL) was added. The reaction was stirred at 23 °C for 2 hours, evaporated, dissolved in buffer A and injected into HPLC (0 to 30% buffer B in buffer A, Waters X-Bridge Shield RP18).

The appropriate fractions were combined, evaporated and lyophilized. The pure triphosphate **dU^put^TP** (9.6 mg, 26%, counted from half of the protected triphosphate used) was obtained as a hydroscopic amorphous solid.

^{1}H NMR (500.0 MHz, CD_3OD): 1.31 (t, 27H, J_{vic} = 7.3, C**H3**CH_2N); 1.80 – 1.88 (m, 2H, H-5″); 1.88 – 1.94 (m, 2H, H-4″); 2.34 (ddd, 1H, J_{gem} = 13.6, $J_{2'b,1'}$ = 6.3, $J_{2'b,3'}$ = 5.3, H-2′b); 2.39 (ddd, 1H, J_{gem} = 13.6, $J_{2'a,3'}$ = 6.1, $J_{2'a,1'}$ = 5.5, H-2′a); 2.95 – 3.01 (m, 2H, H-6″); 3.04 – 3.08 (m, 2H, H-3″); 3.20 (q, 18H, J_{vic} = 7.3, CH_3C**H2**N); 3.98, 4.01 (2 × d, 2 × 1H, J_{gem} = 13.2, H-1″); 4.02 (m, 1H, H-4′); 4.23 (ddd, 1H, J_{gem} = 11.5, $J_{H,P}$ = 5.5, $J_{5'b,4'}$ = 1.9, H-5′b); 4.30 (ddd, 1H, J_{gem} = 11.5, $J_{5'a,4'}$ = 3.7, $J_{H,P}$ = 2.3, H-5′a); 4.65 (ddd, 1H, $J_{3',2'}$ = 6.1, 5.3, $J_{3',4'}$ = 4.0, H-3′); 6.23 (dd, 1H, $J_{1',2'}$ = 6.3, 5.5, H-1′); 8.61 (s, 1H, H-6).
^{13}C NMR (125.7 MHz, CD_3OD): 9.21 (**C**H_3CH_2N); 23.89 (CH_2-4″); 25.19 (CH_2-5″); 39.61 (CH_2-6″); 41.67 (CH_2-2′); 43.84 (CH_2-1″); 47.44 (CH_2-3″); 47.81 (CH_3**C**H_2N); 65.86 (d, $J_{C,P}$ = 5.6, CH_2-5′); 71.05 (CH-3′); 87.30 (CH-1′); 87.81 (d, $J_{C,P}$ = 9.2, CH-4′); 105.74 (C-5); 145.19 (CH-6); 151.97 (C-2); 165.40 (C-4).
^{31}P{^{1}H} NMR (202.4 MHz, CD_3OD): –21.33 (bdd, J = 19.7, 18.8, P_β); –9.45 (d, J = 19.7, P_α); –8.92 (bd, J = 18.8, P_γ).
HRMS (ESI$^-$): *m/z* calcd for $C_{14}H_{26}O_{14}N_4P_3$ [M – H^+] 567.06638; found: 567.06589.

5-Aminomethyl-2′-deoxyuridine-5′-*O*-triphosphate, triethylammonium salt (dU^am^TP)

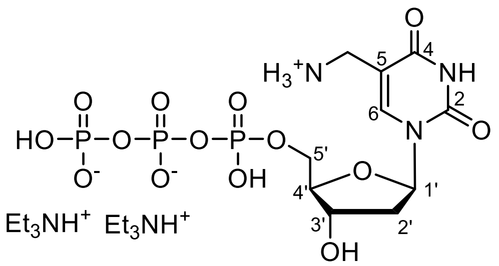

Starting material **dU^tfa^** (30 mg, 0.085 mmol) and Proton Sponge (21.8 mg, 0.10 mmol) were dried on high vacuum for 16 hours and then suspended in $PO(OMe)_3$ (0.28 mL), stirred at 23 °C for 10 min and then cooled to 0 °C. $POCl_3$ (9 µL, 0.10 mmol) was added dropwise over 1 min and the reaction was stirred at 0 °C for 5 hours. Then, Bu_3N (101 µL, 0.43 mmol) and chilled 0.5 M solution of $(Bu_3N)_2H_2P_2O_7$ in MeCN (0.68 mL, 0.34 mmol) were added and the reaction

was stirred at 0 °C for 30 min and then 15 min at 23 °C. The reaction was finished by adding 1 M TEAB (2 mL) and stirred for 15 min. The mixture was diluted with H_2O, evaporated, diluted again with H_2O (5 mL) and lyophilized for 16 hours. The residue was dissolved in 50 mM TEAB in H_2O (2.5 mL) and aqueous ammonia (2.5 mL) was added. The reaction was stirred at 23 °C for 40 min, evaporated, dissolved in H_2O (22 mL) and separated on ion-exchange HPLC (0 to 100% of 800 mM TEAB in H_2O, Sepharose DEAE Fast Flow). The appropriate fraction were collected, evaporated, dissolved in buffer A and injected into HPLC (0 to 40% buffer B in buffer A, Kinetex EVO C18). This separation was done twice. The pure triphosphate **dU^{am}TP** (3.8 mg, 7%) was obtained as a hydroscopic amorphous solid.

^{1}H NMR (500.0 MHz, D_2O): 1.28 (t, 18H, J_{vic} = 7.3, C**H$_3$**CH$_2$N); 2.42 (dt, 1H, J_{gem} = 14.2, $J_{2'b,1'}$ = $J_{2'b,3'}$ = 6.4, H-2′b); 2.45 (ddd, 1H, J_{gem} = 14.2, $J_{2'a,1'}$ = 6.4, $J_{2'a,3'}$ = 4.7, H-2′b); 3.20 (q, 12H, J_{vic} = 7.3, CH$_3$C**H$_2$**N); 4.00 (s, 2H, CH$_2$N); 4.18 – 4.30 (m, 3H, H-4′,5′); 4.70 (m, 1H, H-3′); 6.33 (t, 1H, $J_{1',2'}$ = 6.4, H-1′); 8.35 (s, 1H, H-6).

^{13}C NMR (125.7 MHz, D_2O): 11.06 (**C**H$_3$CH$_2$N); 38.42 (CH$_2$N); 42.26 (CH$_2$-2′); 49.51 (CH$_3$**C**H$_2$N); 67.78 (d, $J_{C,P}$ = 5.5, CH$_2$-5′); 73.28 (CH-3′); 88.66 (CH-1′); 88.74 (d, $J_{C,P}$ = 9.5, CH-4′); 109.45 (C-5); 145.56 (CH-6); 154.31 (C-2); 167.54 (C-4).

^{31}P NMR (202.4 MHz, D_2O): –22.07 (t, J = 19.9, P$_\beta$); –10.58 (d, J = 19.9, P$_\alpha$); –9.08 (bd, J = 19.9, P$_\gamma$).

HRMS (ESI$^-$): *m/z* calcd for $C_{10}H_{17}O_{14}N_3P_3$ [M – H$^+$] 495.99234; found: 495.99278.

5-*N*-(Methyl)-aminomethyl-2′-deoxyuridine-5′-*O*-triphosphate, triethylammonium salt (dU^{mm}TP)

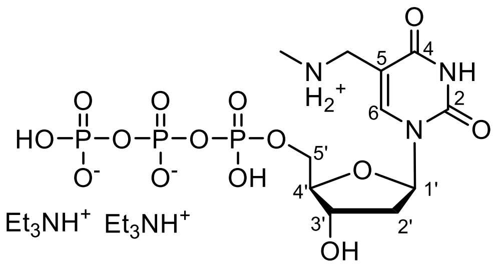

Starting material **dU$^{mm\text{-}tfa}$** (30 mg, 0.082 mmol) and Proton Sponge (21.0 mg, 0.10 mmol) were dried on high vacuum for 16 hours and then suspended in PO(OMe)$_3$ (0.3 mL), stirred at 23 °C for

10 min and then cooled to 0 °C. $POCl_3$ (9 µL, 0.10 mmol) was added dropwise over 1 min and the reaction was stirred at 0 °C for 5 hours. Then, Bu_3N (97 µL, 0.41 mmol) and chilled 0.5 M solution of $(Bu_3N)_2H_2P_2O_7$ in MeCN (0.65 mL, 0.33 mmol) were added and the reaction was stirred at 0 °C for 30 min and then 15 min at 23 °C. The reaction was finished by adding 1 M TEAB (2 mL) and stirred for 15 min. The mixture was diluted with H_2O, evaporated, diluted again with H_2O (5 mL) and lyophilized for 16 hours. The residue was dissolved in 50 mM TEAB in H_2O (2.5 mL) and aqueous ammonia (2.5 mL) was added. The reaction was stirred at 23 °C for 40 min, evaporated, dissolved in H_2O (22 mL) and separated on ion-exchange HPLC (0 to 100% of 800 mM TEAB in H_2O, Sepharose DEAE Fast Flow). The appropriate fraction were collected, evaporated, dissolved in buffer A and injected into HPLC (0 to 40% buffer B in buffer A, Kinetex EVO C18). The pure product **dU$^{\mathbf{mm}}$TP** (12.2 mg, 21%) was obtained as a hydroscopic amorphous solid.

^{1}H NMR (500.0 MHz, D_2O): 1.28 (t, 18H, J_{vic} = 7.3, C**H$_3$**CH$_2$N); 2.42 (dt, 1H, J_{gem} = 14.1, $J_{2'b,1'}$ = $J_{2'b,3'}$ = 6.1, H-2′b); 2.46 (ddd, 1H, J_{gem} = 14.1, $J_{2'a,1'}$ = 6.4, $J_{2'a,3'}$ = 4.8, H-2′b); 2.72 (s, 3H, CH_3N); 3.20 (q, 12H, J_{vic} = 7.3, CH$_3$C**H$_2$**N); 4.00, 4.04 (2 × d, 2 × 1H, J_{gem} = 13.5, CH$_2$N); 4.20 – 4.24 (m, 2H, H-4′,5′b); 4.28 (ddd, 1H, J_{gem} = 12.0, $J_{H,P}$ = 3.5, $J_{5'a,4'}$ = 2.8, H-5′a); 4.70 (m, 1H, H-3′); 6.32 (dd, 1H, $J_{1',2'}$ = 6.4, 6.1, H-1′); 8.41 (s, 1H, H-6).
^{13}C NMR (125.7 MHz, D_2O): 11.06 (**C**H$_3$CH$_2$N); 35.12 (CH$_3$N); 42.29 (CH$_2$-2′); 46.88 (CH$_2$N); 49.51 (CH$_3$**C**H$_2$N); 67.69 (d, $J_{C,P}$ = 5.6, CH$_2$-5′); 73.09 (CH-3′); 88.69 (d, $J_{C,P}$ = 9.3, CH-4′); 88.73 (CH-1′); 107.96 (C-5); 146.65 (CH-6); 154.25 (C-2); 167.69 (C-4).
^{31}P NMR (202.4 MHz, D_2O): –21.82 (bt, J = 19.5, P_β); –10.30 (d, J = 19.5, P_α); –8.92 (bs, P_γ).
HRMS (ESI$^-$): *m/z* calcd for $C_{11}H_{19}O_{14}N_3P_3$ [M – H$^+$] 510.00799; found: 510.00828.

5-(*N,N*-Dimethyl)-aminomethyl-2′-deoxyuridine-5′-*O*-triphosphate, triethylammonium salt (dU^{dm}TP)

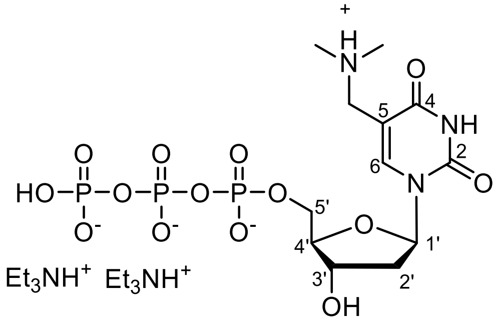

dU^{dm} (30 mg, 0.091 mmol) was dried on high vacuum for 16 hours and then suspended in $PO(OMe)_3$ (0.30 mL), stirred at 23 °C for 10 min and then cooled to 0 °C. $POCl_3$ (10 µL, 0.11 mmol) was added dropwise over 1 min and the reaction was stirred at 0 °C for 4 hours. Then, Bu_3N (108 µL, 0.36 mmol) and chilled 0.5 M solution of $(Bu_3N)_2H_2P_2O_7$ in MeCN (0.72 mL, 0.36 mmol) were added and the reaction was stirred at 0 °C for 30 min and then 15 min at 23 °C. The reaction was finished by adding 1 M TEAB (2 mL) and stirred for 15 min. The mixture was diluted with H_2O, evaporated, diluted again with H_2O (5 mL) and lyophilized for 16 hours. The lyophilizate was dissolved in H_2O (15 mL) and separated on ion-exchange HPLC (0 to 100% of 800 mM TEAB in H_2O, Sepharose DEAE Fast Flow). The appropriate fraction were collected, evaporated, dissolved in buffer A and injected into HPLC (0 to 30% of buffer B in buffer A, Kinetex EVO C18). The pure triphosphate **dU^{dm}TP** (15.9 mg, 24%) was obtained as a hydroscopic amorphous solid.

^{1}H NMR (500.0 MHz, D_2O): 1.28 (t, 27H, J_{vic} = 7.3, C**H$_3$**CH$_2$N); 2.37 (ddd, 1H, J_{gem} = 14,1, $J_{2'b,1'}$ = 7.0, $J_{2'b,3'}$ = 5.8, H-2′b); 2.41 (ddd, 1H, J_{gem} = 14,1, $J_{2'a,1'}$ = 6.5, $J_{2'a,3'}$ = 4.2, H-2′b); 3.20 (q, 18H, J_{vic} = 7.3, CH$_3$C**H$_2$**N); 3.62, 3.66 (2 × dd, 2 × 1H, *J*gem = 17.7, $J_{CH2,6}$ = 0.9, CH$_2$N); 4.19 – 4.27 (nm, 3H, H-4′,5′); 4.67 (m, 1H, H-3′); 6.32 (dd, 1H, $J_{1',2'}$ = 7.0, 6.5, H-1′); 8.08 (t, 1H, $J_{6,CH2}$ = H-6).

^{13}C NMR (125.7 MHz, D_2O): 11.06 (**C**H$_3$CH$_2$N); 18.48 (CH$_2$N); 41.89 (CH$_2$-2′); 49.50 (CH$_3$**C**H$_2$N); 68.07 (d, $J_{C,P}$ = 5.7, CH$_2$-5′); 73.41 (CH-3′); 88.35 (CH-1′); 88.57 (d, $J_{C,P}$ = 9.2, CH-4′); 108.17 (C-5); 121.33 (CN); 143.27 (CH-6); 154.29 (C-2); 167.31 (C-4).

^{31}P NMR (202.4 MHz, D_2O): –21.91 (t, *J* = 19.8, P_β); –10.12 (d, *J* = 19.8, P_α); –9.02 (bs, P_γ).

HRMS (ESI$^-$): *m/z* calcd for $C_{12}H_{21}O_{14}N_3P_3$ [M – H$^+$] 524.02418; found: 524.02443.

5-Cyanomethyl-2′-deoxyuridine-5′-*O*-triphosphate, triethylammonium salt (dU^{cm}TP)

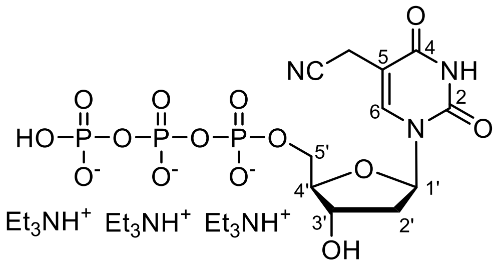

dU^{cm} (40 mg, 0.150 mmol) was dried on high vacuum for 16 hours and then suspended in $PO(OMe)_3$ (0.5 mL), stirred at 23 °C for 10 min and then cooled to 0 °C. $POCl_3$ (16 µL, 0.18 mmol) was added dropwise over 1 min and the reaction was stirred at 0 °C for 4 hours. Then, Bu_3N (178 µL, 0.75 mmol) and chilled 0.5 M solution of $(Bu_3N)_2H_2P_2O_7$ in MeCN (1.2 mL, 0.6 mmol) were added and the reaction was stirred at 0 °C for 30 min and then 15 min at 23 °C. The reaction was finished by adding 1 M TEAB (2 mL) and stirred for 15 min. The mixture was diluted with H_2O, evaporated, diluted again with H_2O (5 mL) and lyophilized for 16 hours. The lyophilizate was dissolved in H_2O (22 mL) and separated on ion-exchange HPLC (0 to 100% of 800 mM TEAB in H_2O, Sepharose DEAE Fast Flow). The appropriate fraction were collected, evaporated, dissolved in buffer A and injected into HPLC (0 to 30% of buffer B in buffer A, Kinetex EVO C18). The pure triphosphate **dU^{cm}TP** (36.9 mg, 30%) was obtained as a hydroscopic amorphous solid.

^{1}H NMR (500.0 MHz, D_2O): 1.28 (t, 27H, J_{vic} = 7.3, C**H$_3$**CH_2N); 2.37 (ddd, 1H, J_{gem} = 14,1, $J_{2'b,1'}$ = 7.0, $J_{2'b,3'}$ = 5.8, H-2′b); 2.41 (ddd, 1H, J_{gem} = 14,1, $J_{2'a,1'}$ = 6.5, $J_{2'a,3'}$ = 4.2, H-2′b); 3.20 (q, 18H, J_{vic} = 7.3, CH_3C**H$_2$**N); 3.62, 3.66 (2 × dd, 2 × 1H, *J*gem = 17.7, $J_{CH2,6}$ = 0.9, CH_2N); 4.19 – 4.27 (nm, 3H, H-4′,5′); 4.67 (m, 1H, H-3′); 6.32 (dd, 1H, $J_{1',2'}$ = 7.0, 6.5, H-1′); 8.08 (t, 1H, $J_{6,CH2}$ = H-6).

^{13}C NMR (125.7 MHz, D_2O): 11.06 (**C**H_3CH_2N); 18.48 (CH_2N); 41.89 (CH_2-2′); 49.50 (CH_3**C**H_2N); 68.07 (d, $J_{C,P}$ = 5.7, CH_2-5′); 73.41 (CH-3′); 88.35 (CH-1′); 88.57 (d, $J_{C,P}$ = 9.2, CH-4′); 108.17 (C-5); 121.33 (CN); 143.27 (CH-6); 154.29 (C-2); 167.31 (C-4).

^{31}P NMR (202.4 MHz, D_2O): –22.50 (t, *J* = 20.2, $P_β$); –10.88 (d, *J* = 20.2, $P_α$); –9.61 (bs, $P_γ$). HRMS (ESI$^–$): *m/z* calcd for $C_{11}H_{15}O_{14}N_3P_3$ [M – H$^+$] 505.97723; found: 505.97747.

5-*N*-(Trifluoroacetyl)-aminomethyl-2′-deoxyuridine-5′-*O*-triphosphate, triethylammonium salt (dU^{tfa}TP)

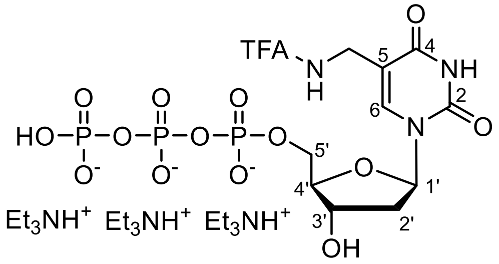

Starting material **dU^{tfa}** (24 mg, 0.068 mmol) and Proton Sponge (30.0 mg, 0.14 mmol) were dried on high vacuum for 16 hours and then suspended in $PO(OMe)_3$ (0.3 mL), stirred at 23 °C for 10 min and then cooled to 0 °C. $POCl_3$ (8 µL, 0.09 mmol) was added dropwise over 1 min and the reaction was stirred at 0 °C for 5 hours. Then, Bu_3N (80 µL, 0.34 mmol) and chilled 0.5 M solution of $(Bu_3N)_2H_2P_2O_7$ in MeCN (0.54 mL, 0.27 mmol) were added and the reaction was stirred at 0 °C for 30 min and then 15 min at 23 °C. The reaction was finished by adding 1 M TEAB (2 mL) and stirred for 15 min. The mixture was diluted with H_2O, evaporated, diluted again with H_2O (5 mL) and lyophilized for 16 hours. The residue was dissolved in H_2O (20 mL) and separated on ion-exchange HPLC (0 to 100% of 800 mM TEAB in H_2O, Sepharose DEAE Fast Flow). The appropriate fraction were collected, evaporated, dissolved in buffer A and injected into HPLC (0 to 40% buffer B in buffer A, Kinetex EVO C18). Another HPLC (0 to 20% buffer B in buffer A, Kinetex EVO C18) was performed, the fractions containing pure desired triphosphate were combined and evaporated. The pure triphosphate **dU^{tfa}TP** (2.1 mg, 3.4%) was obtained as a hydroscopic amorphous solid.

^1H NMR (600.1 MHz, D_2O): 1.28 (t, 27H, J_{vic} = 7.3, C**H$_3$**CH$_2$N); 2.40 (dd, 2H, $J_{2′,1′}$ = 6.7, $J_{2′,3′}$ = 5.1, H-2′); 3.20 (t, 18H, J_{vic} = 7.3, CH$_3$C**H$_2$**N); 4.19 – 4.25 (m, 3H, H-4′,5′); 4.26, 4.31 (2 × d, 2 × 1H, J_{gem} = 14.7, CH$_2$N); 4.67 (m, 1H, H-3′); 6.30 (t, 1H, $J_{1′,2′}$ = 6.7, H-1′); 8.04 (s, 1H, H-6).

^{13}C NMR (150.9 MHz, D_2O): 11.06 (**C**H_3CH_2N); 39.05 (CH_2N); 41.78 (CH_2-2′); 49.51 (CH_3**C**H_2N); 68.08 (d, $J_{C,P}$ = 5.7, CH_2-5′); 73.40 (CH-3′); 88.40 (CH-1′); 88.55 (d, $J_{C,P}$ = 9.1, CH-4′); 112.64 (C-5); 118.66 (q, $J_{C,F}$ = 286.1, **C**F_3CO); 143.46 (CH-6); 154.37 (C-2); 161.34 (q, $J_{C,F}$ = 37.4, CF_3**C**O); 167.50 (C-4).

^{19}F NMR (470.7 MHz, D_2O): –72.02.

^{31}P NMR (202.4 MHz, D_2O): –22.25 (bm, P_β); –10.75 (d, J = 19.5, P_α); –9.35 (bm, P_γ).

HRMS (ESI$^-$): *m/z* calcd for $C_{12}H_{16}O_{15}N_3F_3P_3$ [M – H^+] 591.97518; found: 591.97538.

5-(Acetylthio)methyl-2′-deoxyuridine-5′-*O*-triphosphate, triethylammonium salt (dU^{asm}TP) and 5-mercaptomethyl-2′-deoxyuridine-5′-*O*-triphosphate, triethylammonium salt (dU^{sm}TP)

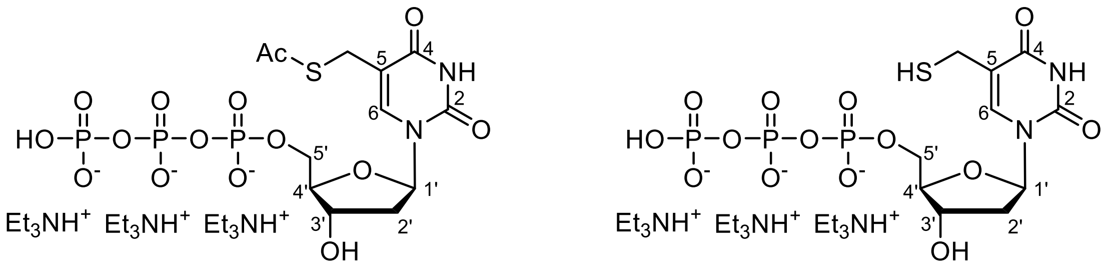

dU^{asm} (50 mg, 0.158 mmol) and Proton Sponge (50.8 mg, 0.24 mmol) were dried on high vacuum for 16 hours and then suspended in $PO(OMe)_3$ (0.5 mL), stirred at 23 °C for 10 min and then cooled to 0 °C. $POCl_3$ (18 µL, 0.19 mmol) was added dropwise over 1 min and the reaction was stirred at 0 °C for 4 hours. Then, Bu_3N (185 µL, 0.79 mmol) and chilled 0.5 M solution of $(Bu_3N)_2H_2P_2O_7$ in MeCN (1.25 mL, 0.63 mmol) were added and the reaction was stirred at 0 °C for 30 min and then 15 min at 23 °C. The reaction was finished by adding 1 M TEAB (2 mL) and stirred for 15 min. The mixture was diluted with H_2O, evaporated, diluted again with H_2O (5 mL) and lyophilized for 16 hours. The residue was dissolved in H_2O (22 mL) and separated on ion-exchange HPLC (0 to 100% of 800 mM TEAB in H_2O, Sepharose DEAE Fast Flow). The appropriate fraction were collected and evaporated.

For **dU^{sm}TP**, half of the crude product was dissolved in 50 mM TEAB in H_2O (2.5 mL) and aqueous ammonia (2.5 mL) was added. The reaction was stirred at 23 °C for 40 min, evaporated, dissolved

in buffer A and injected into HPLC (0 to 30% of buffer B in buffer A, Kinetex EVO C18). The pure triphosphate **dU^{sm}TP** (13.7 mg, 21%, calculated from half of the starting material) was obtained as a hydroscopic amorphous solid.

For **dU^{asm}TP**, the other half was repurified by HPLC (0 to 30% of buffer B in buffer A, Kinetex EVO C18). The pure triphosphate **dU^{asm}TP** (15.4 mg, 23%, calculated from half of the starting material) was obtained as a hydroscopic amorphous solid.

dU^{sm}TP:

^{1}H NMR (500.0 MHz, D_2O): 1.27 (t, 27H, J_{vic} = 7.3, C**H$_3$**CH$_2$N); 2.36 (ddd, 1H, J_{gem} = 14.0, $J_{2'b,1'}$ = 6.5, $J_{2'b,3'}$ = 4.0, H-2′b); 2.40 (ddd, 1H, J_{gem} = 14.0, $J_{2'a,1'}$ = 7.2, $J_{2'a,3'}$ = 5.8, H-2′b); 3.20 (q, 18H, J_{vic} = 7.3, CH$_3$C**H$_2$**N); 3.53 (s, 2H, CH$_2$S); 4.18 – 4.27 (m, 3H, H-4′,5′b); 4.68 (m, 1H, H-3′); 6.33 (dd, 1H, $J_{1',2'}$ = 7.2, 6.5, H-1′); 7.97 (s, 1H, H-6).
^{13}C NMR (125.7 MHz, D_2O): 11.07 (**C**H$_3$CH$_2$N); 23.04 (CH$_2$S); 41.65 (CH$_2$-2′); 49.49 (CH$_3$**C**H$_2$N); 68.17 (d, $J_{C,P}$ = 5.9, CH$_2$-5′); 73.56 (CH-3′); 88.10 (CH-1′); 88.52 (d, $J_{C,P}$ = 9.1, CH-4′); 118.16 (C-5); 141.06 (CH-6); 154.38 (C-2); 167.75 (C-4).
^{31}P NMR (202.4 MHz, D_2O): –22.49 (t, J = 20.2, P_β); –10.95 (d, J = 20.2, P_α); –9.21 (bd, J = 20.2, P_γ).
HRMS (ESI$^-$): *m/z* calcd for $C_{10}H_{16}O_{14}N_2P_3S$ [M – H$^+$] 512.95406; found: 512.95395.

dU^{asm}TP:

^{1}H NMR (500.0 MHz, D_2O): 1.27 (t, 27H, J_{vic} = 7.3, C**H$_3$**CH$_2$N); 2.35 (s, 3H, CH$_3$CO); 2.35 – 2.40 (m, 2H, H-2′); 3.19 (q, 18H, J_{vic} = 7.3, CH$_3$C**H$_2$**N); 3.87, 3.90 (2 × d, 2 × 1H, J_{gem} = 14.1, CH$_2$S); 4.18 – 4.26 (m, 3H, H-4′,5′b); 4.65 (td, 1H, $J_{3',2'}$ = 5.0, 2.9, $J_{3',4'}$ = 5.0, H-3′); 6.29 (t, 1H, $J_{1',2'}$ = 6.8, H-1′); 7.99 (s, 1H, H-6).
^{13}C NMR (125.7 MHz, D_2O): 11.07 (**C**H$_3$CH$_2$N); 28.64 (CH$_2$S); 32.69 (**C**H$_3$CO); 41.45 (CH$_2$-2′); 49.50 (CH$_3$**C**H$_2$N); 68.29 (d, $J_{C,P}$ = 5.9, CH$_2$-5′); 73.63 (CH-3′); 88.27 (CH-1′); 88.42 (d, $J_{C,P}$ = 8.8, CH-4′); 113.54 (C-5); 142.81 (CH-6); 154.25 (C-2); 167.72 (C-4); 203.28 (CH$_3$**C**O).
^{31}P NMR (202.4 MHz, D_2O): –22.63 (t, J = 20.0, P_β); –10.95 (d, J = 20.0, P_α); –10.17 (d, J = 20.0, P_γ).

HRMS (ESI$^-$): *m/z* calcd for $C_{12}H_{18}O_{15}N_2P_3S$ [M – H$^+$] 554.96462; found: 554.96448.

2. Experimental section - biochemistry part

2.1. Scheme of used nucleoside triphosphates

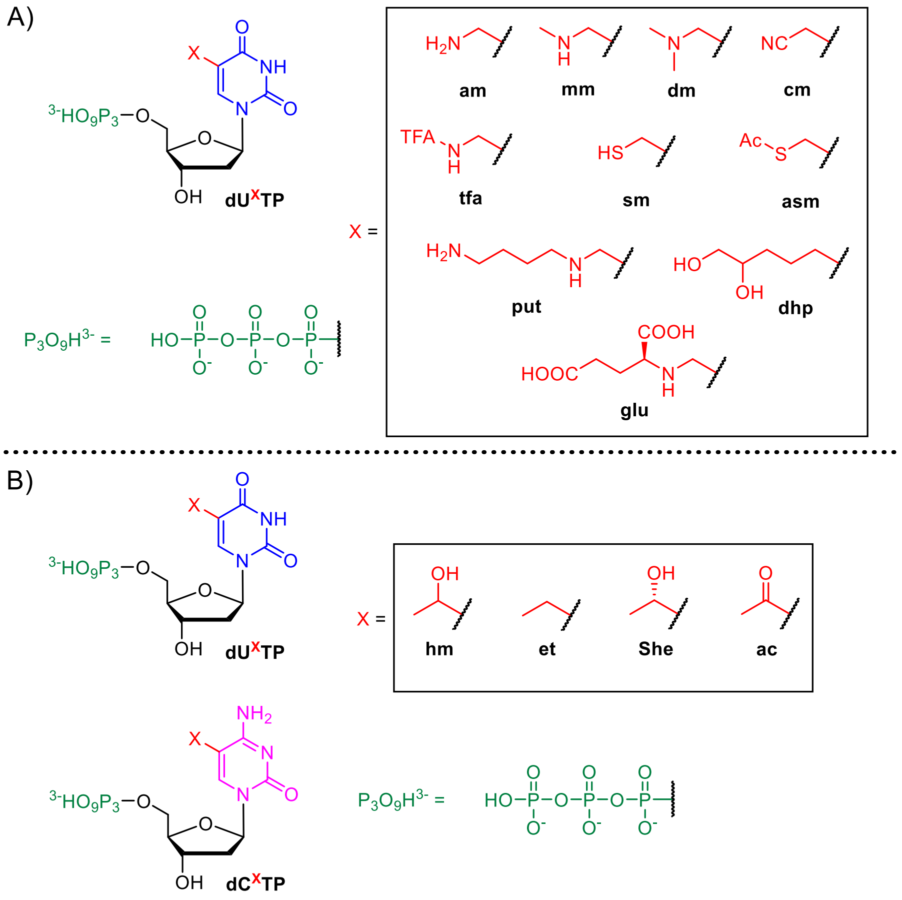

Figure S1. Nucleoside triphosphates used in this study. A) Nucleoside triphosphates prepared in this study. B) Nucleoside triphosphates prepared according to already published protocols in previous studies.[2,3]

2.2. General remarks – biochemistry

Synthetic oligonucleotides (non-modified, 5'-6-FAM-labelled with 6-carboxyfluorescein, 5'-phosphorylated, ribonucleotide-modified, 5'-6-FAM-labelled 2'-methoxyribonucleotide-modified and 5'-Cy5-labelled) were purchased either from Eurofins Genomics, Biomers, or Generi Biotech. Natural 2′-deoxynucleoside triphosphates (dATP, dCTP, dGTP, dTTP) were purchased from New England Biolabs (NEB). KOD XL DNA polymerase was purchased from Merck, Vent(exo$^-$) DNA polymerase, HotStart Q5 DNA polymerase, T4 DNA ligase, Lambda

exonuclease and DNase I from NEB, M-MLV reverse transcriptase from Thermo Fisher Scientific. All chemicals were purchased from commercial suppliers and were of analytical, molecular biology (BioUltra) or LC-MS grade. Milli-Q water was used for buffers, UPLC-grade water was used in reaction mixtures, DEPC-treated water was used in transcription reactions and reverse transcription. SYBR Gold (10 000X concentrate in DMSO) was purchased from Thermo Fisher Scientific. All PEX products, PCR products and final DNA templates were purified on columns (MinElute PCR Purification Kit and QIAquick Nucleotide Removal Kit from QIAGEN; E.Z.N.A. Gel Extraction Kit from Omega Bio-Tek) or on Agencourt AMPure XP magnetic particles (Beckman Coulter Life Science - GE Healthcare). RNA was purified by Monarch RNA Cleanup kit by NEB. Column purifications were done according to the manufacturers' manuals, unless specified otherwise. Samples after reactions were analyzed by 12.5% denaturing polyacrylamide gel (PAGE, acrylamide/bisacrylamide 19:1, Roth) under denaturing conditions (1 h, 50 °C, 7M urea, 1X TBE buffer) or by 12.5% native PAGE (acrylamide/bisacrylamide 19:1, Roth) under native conditions (3 h, 23 °C, 1X TBE buffer). PAGE stop solution used contains: 95% [v/v] formamide, 0.5 mM EDTA, 0.025% [w/v] bromophenol blue, 0.025% [w/v] xylene cyanol FF, 0.025% [w/v] SDS. 6X Native PAGE loading dye contains: 40% [w/v] saccharose, 0.02% [w/v] orange G, 0.02% [w/v] bromophenol blue in Milli-Q water. PCR reactions were analyzed on agarose gel (SERVA, 8 V/cm, 1 hod, 0.5X TBE buffer), using 6X SDS containing loading dye (NEB). PAGE and agarose gels were scanned by fluorescence imaging using Typhoon FLA 9500 or Amersham Typhoon Gel Scanner (Cytiva). Purification of DNA from gel was done using EDVOTEK TruBlu™ 2 Blue/White Transilluminator. UV-Vis spectra (concentration of products) were measured at 23 °C on NanoDrop1000 (ThermoFisher Scientific) or Nanophotometer N60 (Implen). LC-MS measurements of DNA were measured on Agilent 1920 Infinity II BIO system with MSD XT mass spectrometer equipped with ESI ion source, using Phenomenex Biozen 1.7μm Oligo 50 x 2.1 mm column together with buffer A (300 mM HFIP + 15 mM TEA in H_2O) and buffer B (300 mM HFIP + 15 mM TEA in MeOH). Aquired spectra were deconvoluted using UniDec program[10] with a deconvolution resolution of ± 0.5 Da. Next generation sequencing (NGS) was done on Illumina NovaSeq with an output of 2 millions of 2 x 150 bp paired-end reads per sample (Novogene). Raw, paired-end data were pre-processed by trimming adaptors, merging, primer cliping and length-filtering using standard bioinformatics tools and bash commands. Then, for each sample, position-wise nucleotide frequencies were computed with in-house scripts.

2.3. General annealing procedure

The annealing mixture (100 µL) contained both complementary oligonucleotides (each 10 µM) in annealing buffer consisting of 10 mM Tris, 50 mM NaCl, 1 mM EDTA, pH = 7.8. The mixture was then subjected to following thermal cycler program: 95 °C for 5 min, followed by cooling from 95 °C to 23 °C over 50 min.

Table S1. List of oligonucleotides used for annealing and their dsDNA products

Name of oligonucleotide	Name of annealed product	Sequence (5‘→ 3‘)$^{a, b, c}$	Length (nt)
**Prim^{20ON}-FAM**	**20DNA**	F-GACATCATGAGAGACATCGC	20
**Oligo^{20ON}-P**		P-GCGATGTCTCTCATGATGTC	20
**Prim^{70ON}-FAM**	**70DNA**	F-[mG][mC][mU]CGACCAGGATGGGCACCACCCCGGTGAACAGCT CCTCGCCCTTGCTCACCATGGTGGCGGCTCTC*CC*	70
**Oligo^{70ON}-P**		P-*GG*GAGAGCCGCCACCATGGTGAGCAAGGGCGAGGAGCTGTTC ACCGGGGTGGTGCCCATCCTGGTCGAGC	70

^{a} F = 5′- 6-FAM-labelled; ^{b} P = 5′- phosphorylated; ^{c} [mN] 2’-OMe

2.4. Initial testing of prepared nucleoside triphosphates

2.4.1. Preparation of 19DNA and 19DNA_U^{X} by PEX

Table S2. Used oligonucleotides and prepared dsDNA

Name	Sequence (5‘→ 3‘)$^{b, c, d}$	Length (nt)
**Prim^{15ON}-FAM**	F-CATGGGCGGCATGGG	15
**Temp^{19ON_T}**	CCCACCCATGCCGCCCATG	19
**19DNA**	F-CATGGGCGGCATGGGTGGG	19
**19DNA_U$^{X\,a}$**	F-CATGGGCGGCATGGG**U***GGG	19

^{a} set of modified **dU^{X}** used; ^{b} F = 5′- 6-FAM-labelled; ^{c} * position of modified nucleotide; ^{d} primer sequences in templates underlined; DNA – double stranded

The reaction mixture (20 µL) contained KOD XL DNA polymerase (0.125 U), natural dNTPs (dATP, dGTP, dCTP, 160 µM each), primer **Prim^{15ON}-FAM** (3 µM), template **Temp^{19ON_T}** (3 µM), appropriate **dU^{X}TP** (for natural DNA dTTP, 160 µM) and KOD XL polymerase reaction

buffer (2 μL). The reactions were incubated for 30 min at 60 °C, then 1 μL of each sample was pipetted into a mixture of 10 μL PAGE stop solution and 9 μL of H_2O, followed by denaturing at 95 °C for 5 min. Samples were analyzed on a 12.5% denaturing PAGE gel and visualized using fluorescence imaging (Figure S2). The reactions were then purified using QIAquick nucleotide removal kit (eluted in 30 μL of H_2O). The products (**19DNA**, **19DNA_U^{X}** and **19DNA_C^{X}**) were further analyzed by LC-MS (Table S3, Figures S23 – S46).

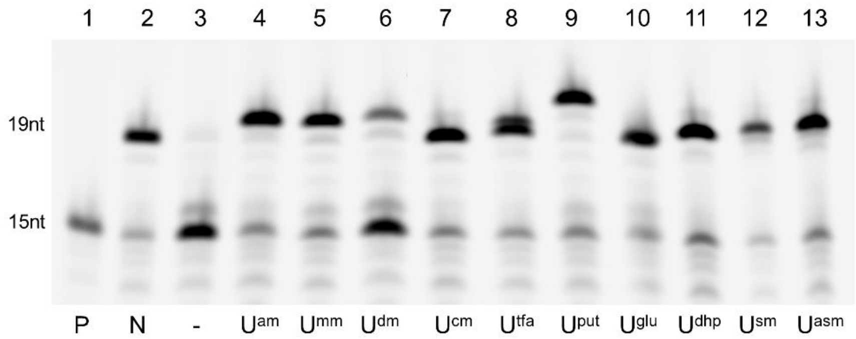

Figure S2. dPAGE analysis of PEX products using KOD XL DNA polymerase, template **Temp^{19ON}-^{T}** and primer **Prim^{15ON}-FAM**. Lane 1 (P): primer **Prim^{15ON}-FAM**; lane 2 (N): product with all natural dNTPs; lane 3 (-): product in absence of dTTP and **dU^{X}TP**; lanes 4-13: products in presence of **dU^{X}TP** and remaining natural dNTPs. In lane 8 (U^{tfa}), upper band of TFA-deprotected modification is visible (see LC-MS measurements, Table S3).

Table S3. Summary of LC-MS measurements of 19DNA/19DNA_U^{X}

Name	Mw (modified strand) calculated [Da]	Mw (modified strand) found [Da]	Δ [Da]	Figure number
**19DNA**	6502.9	6502.0	0.9	23, 24
**19DNA_U^{am}**	6517.9	6517.0	0.9	25, 26
**19DNA_U^{mm}**	6531.9	6531.5	0.4	27, 28
**19DNA_U^{dm}**	6546.0	6545.5	0.5	29, 30
**19DNA_U^{cm}**	6527.9	6527.5	0.4	31, 32
**19DNA_U^{tfa}**	6613.9	6612.0, 6517.0[a]	1.9, –	33 – 36
**19DNA_U^{put}**	6589.0	6588.5	0.5	37, 38
**19DNA_U^{glu}**	6647.9	6647.5	0.4	39, 40
**19DNA_U^{dhp}**	6591.0	6590.5	0.5	41, 42
**19DNA_U^{sm}**	6534.9	6533.5	1.4	43, 44
**19DNA_U^{asm}**	6576.9	6576.0	0.9	45, 46

[a] Mw assigned to modified strand lacking TFA protecting group

2.4.2. Preparation of 31DNA and 31DNA_U^X by PEX

Table S4. Used oligonucleotides and prepared dsDNA

Name	Sequence (5'→ 3')[b, c, d]	Length (nt)
**Prim^{15ON}-FAM**	F-CATGGGCGGCATGGG	15
**Temp^{31ON}**	CTAGCATGAGCTCAGTCCCATGCCGCCCATG	31
**31DNA**	F-CATGGGCGGCATGGGACTGAGCTCATGCTAG	31
**31DNA_U^X** [a]	F-CATGGGCGGCATGGGAC**U***GAGC**U***CA**U***GC**U***AG	31

[a] set of modified **dU^X** used; [b] F = 5′- 6-FAM-labelled; [c] * position of modified nucleotide; [d] primer sequences in templates underlined; DNA – double stranded

The reaction mixture (50 μL) contained KOD XL DNA polymerase (1.5 U), natural dNTPs (dATP, dGTP, dCTP, 200 μM each), primer **Prim^{15ON}-FAM** (3 μM), template **Temp^{31ON}** (3 μM), appropriate **dU^{X}TP** (for natural DNA dTTP, 200 μM) and KOD XL polymerase reaction buffer (5 μL). The reactions were incubated for 40 min at 55 °C, followed by 20 min at 72 °C. 1μL of each sample was then pipetted into a mixture of 10 μL PAGE stop solution and 9 μL of H_2O, followed by denaturing at 95 °C for 5 min. Samples were analyzed on a 12.5% denaturing PAGE gel and visualized using fluorescence imaging (Figure S3). The reactions were then purified using QIAquick nucleotide removal kit (eluted in 30 μL of H_2O). The products (**31DNA** and **31DNA_U^X**) were further analyzed by LC-MS (Table S5, Figures S47 – S70).

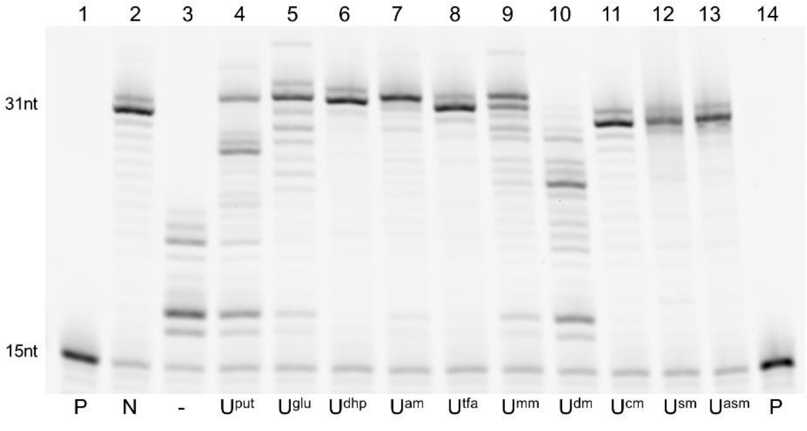

Figure S3. dPAGE analysis of PEX products using KOD XL DNA polymerase, template **Temp^{31ON}** and primer **Prim^{15ON}-FAM**. Lanes 1 and 14 (P): primer **Prim^{15ON}-FAM**; lane 2 (N): product with all natural dNTPs; lane 3 (-): product in absence of dTTP and **dU^{X}TP**; lanes 4-13: products in presence of **dU^{X}TP** and remaining natural dNTPs.

Table S5. Summary of LC-MS measurements of 31DNA/31DNA_U^X

Name	Mw (modified strand) calculated [Da]	Mw (modified strand) found [Da]	Δ [Da]	Figure number
**31DNA**	10154.3	10152.0	2.3	47, 48
**31DNA_U^{am}**	10214.4	10212.0	2.4	49, 50
**31DNA_U^{mm}**	10270.5	10268.0, 9939.0[a]	2.5, –	51, 52
**31DNA_U^{dm}**	10326.6	not detected	–	53, 54
**31DNA_U^{cm}**	10254.3	10252.0	2.3	55, 56
**31DNA_U^{tfa}**	10598.4	10596.5, 10500.5[b]	1.9, –	57 – 60
**31DNA_U^{put}**	10498.9	not detected	–	61, 62
**31DNA_U^{glu}**	10734.8	10733.0	1.8	63, 64
**31DNA_U^{dhp}**	10506.7	10504.0	2.7	65, 66
**31DNA_U^{sm}**	10282.5	not detected	–	67, 68
**31DNA_U^{asm}**	10450.7	10448.5, 10407.5[c]	2.2, –	69, 70

[a] Mw assigned to modified strand lacking dG; [b] Mw assigned to modified strand lacking one TFA protecting group; [c] Mw assigned to modified strand lacking one Ac protecting group

2.4.3. Preparation of 98DNA and 98DNA_U^{X} by PCR

Table S6. Used primers and prepared dsDNA products

Name	Strand	Sequence (5‘→ 3‘) [b, c, d, e]	Length (nt)
**Prim^{20ON}-FAM**		F-GACATCATGAGAGACATCGC	20
**Prim^{25ON}-Cy5**		Cy5-CAAGGACAAAATACCTGTATTCCTT	25
**98DNA**	sense	GACATCATGAGAGACATCGCCTCTGGGCTAATAGGA CTACTTCTAATCTGTAAGAGCAGATCCCTGGACAGGCA AGGAATACAGGTATTTTGTCCTTG	98
	antisense	CAAGGACAAAATACCTGTATTCCTTGCCTGTCCAGGG ATCTGCTCTTACAGATTAGAAGTAGTCCTATTAGCCCAG AGGCGATGTCTCTCATGATGTC	
**98DNA_PCR**	sense	F-GACATCATGAGAGACATCGCCTCTGGGCTAATAGGA CTACTTCTAATCTGTAAGAGCAGATCCCTGGACAGGCA AGGAATACAGGTATTTTGTCCTTG	98
	antisense	Cy5-CAAGGACAAAATACCTGTATTCCTTGCCTGTCCAGGG ATCTGCTCTTACAGATTAGAAGTAGTCCTATTAGCCCAG AGGCGATGTCTCTCATGATGTC	
**98DNA_U^{X}** [a]	sense	F-GACATCATGAGAGACATCGCCU*CU*GGGCU*AAU*AGGA CU*ACU*U*CU*AAU*CU*GU*AAGAGCAGAU*CCCU*GGACA GGCAAGGAAU*ACAGGU*AU*U*U*U*GU*CCU*U*G	98
	antisense	Cy5-CAAGGACAAAATACCTGTATTCCTTGCCU*GU*CCAGGG AU*CU*GCU*CU*U*ACAGAU*U*AGAAGU*AGU*CCU*AU*U* AGCCCAGAGGCGAU*GU*CU*CU*CAU*GAU*GU*C	

[a] set of modified **dU^{X}** used; [b] F = 5′- 6-FAM-labelled; [c] Cy5 = 5′- Cy5-labelled; [d] * position of modified nucleotide; [e] primer sequences in templates underlined; DNA – double stranded

The PCR mixture (20 μL) contained primers (**Prim^{20ON}-FAM** and **Prim^{25ON}-Cy5**, each 1 μM), mixture of three natural dNTPs (dATP, dGTP, dCTP; 200 μM) and **dU^{X}TP** (400 μM; for natural DNA dTTP, 200 μM), additional $MgSO_4$ (**dU^{put}TP**, **dU^{glu}TP**, **dU^{dhp}TP**, **dU^{am}TP**, **dU^{tfa}TP**, **dU^{mm}TP** and **dU^{dm}TP**; extra 5 mM), additional dithiothreitol (**dU^{sm}TP** and **dU^{asm}TP**; extra 25 mM), template **98DNA** (5 nM), KOD XL buffer (2 μL) and KOD XL DNA polymerase (0.5 U for natural DNA, 1 U for **dU^{dhp}TP** and **dU^{cm}TP**; 2 U for the remaining modifications). All reaction mixtures were run under following cycling conditions: preheating at 95 °C for 3 min, followed by 25 cycles of denaturation at 95 °C for 30 sec, annealing at 54 °C for 30 sec and extension at 72 °C for 1.25 min, finished by final extension at 72 °C for 5 min. 1μL of each sample

was pipetted into a mixture of 10 µL PAGE stop solution and 9 µL of H_2O, followed by denaturation at 95 °C for 5 min. Samples were analyzed on a 12.5% denaturing PAGE gel and visualized using fluorescence imaging (Figure S4). The reactions that provided full length product were then purified using QIAquick PCR purification kit (eluted in 30 µL of H_2O). Products **98DNA_PCR** and **98DNA_U^{tfa}** were further analyzed by LC-MS (section 2.4.4., Table S7, Figures S133 – S140) and **98DNA_U^{tfa}** was used for deprotection experiment (section 2.4.4.).

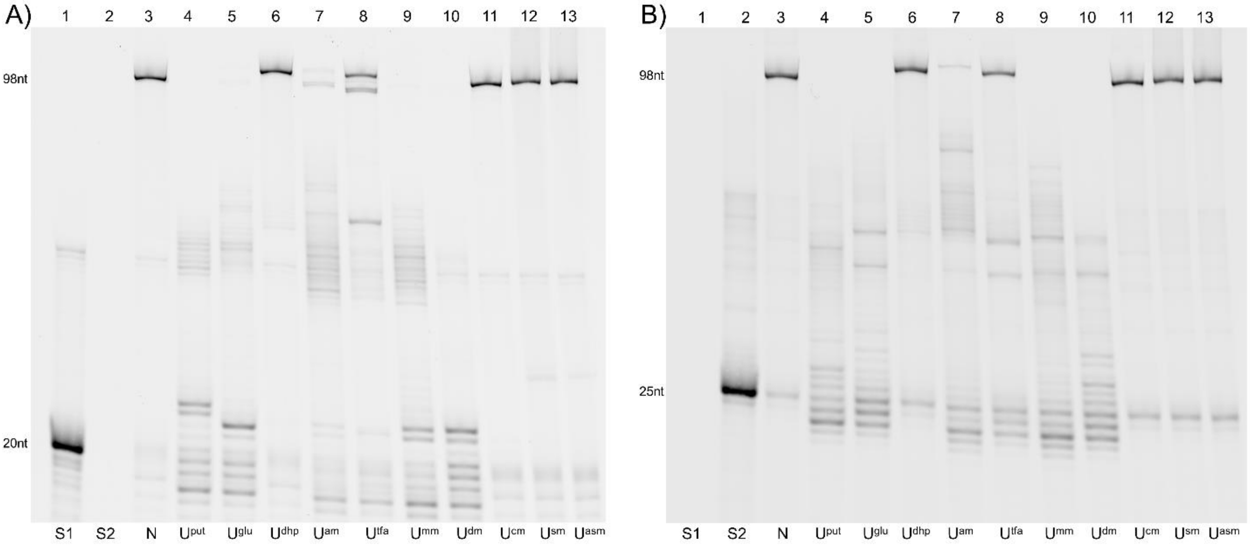

Figure S4. dPAGE analysis of PCR products using template **98DNA** amplified by KOD XL DNA polymerase, in 6-FAM (A) and Cy5 (B) scans. Both A) and B): Lane 1 (S1): **Prim^{20ON}-FAM**; lane 2 (S2): **Prim^{25ON}-Cy5**; lane 3 (N): all natural dNTPs; lanes 4-13: appropriate **dU^{X}TP** with remaining natural dNTPs.

2.4.4. TFA deprotection on 98DNA_U^{tfa} to prepare 98DNA_U^{am}

The reaction mixture (20 µL) contained **98DNA_U^{tfa}** (40.5 ng/µL, 19 µL) and NaOH (1 M, 1 µL). The mixture was incubated at 23 °C for 1 hour, followed by addition of HCl (100 mM, 10 µL) and Tris-HCl buffer (1 M, pH = 8.0, 1 µL). The mixture was then analyzed by LC-MS (Table S7, Figures S141 – S144) and compared to original **98DNA_U^{tfa}**.

Table S7. Summary of LC-MS measurements of 98DNA_PCR, 98DNA_ U^{tfa} and 98DNA_U^{am} prepared by TFA deprotection on 98DNA_U^{tfa}

Name	strand	Mw calculated [Da]	Mw found [Da]	Δ [Da]	Figure number
**98DNA_PCR**	sense	30823.7	30820.5	3.2	133, 134
	antisense	30672.6	30668.5	4.1	135, 136
**98DNA_U^{tfa}**	sense	33266.2	not detected	–	137, 138
	antisense	33004.1	not detected	–	139, 140
**98DNA_U^{am}**	sense	31154.0	31151.5	2.5	141, 142
	antisense	30987.9	30984.0	3.9	143, 144

2.5. Preparation of DNA by PEX

2.5.1. Preparation of 37DNA, 37DNA_U^{X} and 37DNA_C^{X} by PEX

Table S8. Used oligonucleotides and prepared dsDNA

Name	Sequence (5‘→ 3‘) [c, d, e, f, g]	Length (nt)
**Prim^{20ON}-FAM**	F-GACATCATGAGAGACATCGC	20
**Temp^{37ON}-P**	P-*TATAGTGAGTCGTATTA*GCGATGTCTCTCATGATGTC	37
**37DNA**	F-GACATCATGAGAGACATCGC*TAATACGACTCACTATA*	37
**37DNA_U^{X} [a]**	F-GACATCATGAGAGACATCGC***U*****AA*****U****ACGAC***U****CAC***U****A***U****A*	37
**37DNA_C^{X} [b]**	F-GACATCATGAGAGACATCGC*TAATA****C****GA****C****T****C****A****C****TATA*	37

[a] set of modified **dU^{X}** used; [b] set of modified **dC^{X}** used; [c] F = 5′- 6-FAM-labelled; [d] P = 5′- phosphorylated; [e] * position of modified nucleotide; [f] primer sequences in templates underlined; [g] T7 promoter sequence in italic; DNA – double stranded

The reaction mixture (100 μL) contained primer **Prim^{20ON}-FAM** (2.5 μM), template **Temp^{37ON}-P** (2.5 μM), natural dNTPs (dATP, dGTP and either dCTP or dTTP, 200 μM each), appropriate **dU^{X}TP** or **dC^{X}TP** (for natural DNA either dTTP or dCTP, 200 μM; 300 μM for **dU^{ac}TP**; 400 μM for **dU^{glu}TP**, **dU^{am}TP**, **dU^{mm}TP**), KOD XL polymerase reaction buffer (10 μL) and KOD XL DNA polymerase (5 U; 7.5 U for **dU^{glu}TP**, **dU^{am}TP**, **dU^{mm}TP**). The reactions were then subjected to the following thermal cycler program: 95 °C for 5 min, followed by 55 °C for 10 min and then 72 °C for 5 min. The reactions were purified using QIAquick nucleotide removal kit (eluted in 30 μL of 5 mM Tris-Cl buffer, pH = 8.5). 1μL of each sample was pipetted into a mixture of 10 μL PAGE stop solution and 9 μL of H_2O, followed by

denaturation at 95 °C for 5 min. Samples were analyzed on a 12.5% denaturing PAGE gel and visualized using fluorescence imaging (Figure S5). The products (**37DNA**, **37DNA_U^{X}** and **37DNA_C^{X}**) were further analyzed by LC-MS (Table S9, Figures S71 – S102) and used for: 1) Preparation of **107DNA_S**, **107DNA_S_U^{X}** and **107DNA_S_C^{X}** by ligation (section 2.7.2.)

2) Preparation of **37ON** and **37ON_U^{X}** by Lambda exonuclease digestion (section 2.6.1.)

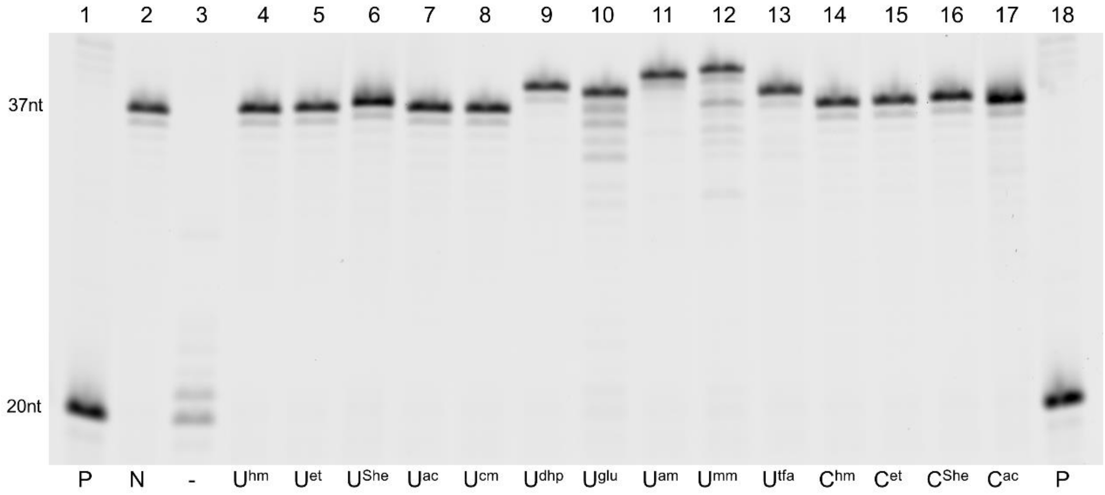

Figure S5. dPAGE analysis of PEX products using KOD XL DNA polymerase, template **Temp^{37ON}-P** and primer **Prim^{20ON}-FAM**. Lanes 1 and 18 (P): **Prim^{20ON}-FAM**; lane 2 (N): product with all natural dNTPs; lane 3 (-): product in absence of dTTP and **dT^{X}TP**; lanes 4-17: products in presence of either **dU^{X}TP** or **dC^{X}TP** and the remaining natural dNTPs.

Table S9. Summary of LC-MS measurements of 37DNA/37DNA_U^{X}/37DNA_C^{X}

Name	Mw (modified strand) calculated [Da]	Mw (modified strand) found [Da]	Δ [Da]	Figure number
**37DNA**	11871.5	11870.5	1.0	71, 72
**37DNA_U**^{hm}	11951.5	11950.5	1.0	73, 74
**37DNA_U**^{et}	11941.7	11941.0	0.7	75, 76
**37DNA_U**^{She}	12021.7	12020.5	1.2	77, 78
**37DNA_U**^{ac}	12011.6	12011.0	0.6	79, 80
**37DNA_U**^{cm}	11996.6	11995.5	1.1	81, 82
**37DNA_U**^{dhp}	12312.1	12311.0	1.1	83, 84
**37DNA_U**^{glu}	12597.1	12596.5	0.6	85, 86
**37DNA_U**^{am}	11946.6	11945.5	1.1	87, 88
**37DNA_U**^{mm}	12016.7	12016.5	0.2	89, 90
**37DNA_U**^{tfa}	12426.6	12426.0, 12330.0[a]	0.6, –	91 – 94
**37DNA_C**^{hm}	11991.6	11991.5	0.1	95, 96
**37DNA_C**^{et}	11983.7	11983.0	0.7	97, 98
**37DNA_C**^{She}	12047.7	12046.5	1.2	99, 100
**37DNA_C**^{ac}	12039.6	12039.0	0.6	101, 102

[a] Mw assigned to modified strand lacking one TFA protecting group

2.5.2. Preparation of 87DNA and 87DNA_U^{X} by PEX

Table S10. Used oligonucleotides and prepared dsDNA

Name	Sequence (5‘→ 3‘) [b, c, d, e, f, g]	Length (nt)
**Prim**^{70ON}**-FAM**	F-[mG][mC][mU]CGACCAGGATGGGCACCACCCCGGTGAACAGCTCCTCGCCCT TGCTCACCATGGTGGCGGCTCTC*CC*	70
**Temp**^{87ON}**-P**	P-*TAATACGACTCACTATAGG*GAGAGCCGCCACCATGGTGAGCAAGGGCGAGGAG CTGTTCACCGGGGTGGTGCCCATCCTGGTCGAGC	87
**87DNA**	F-[mG][mC][mU]CGACCAGGATGGGCACCACCCCGGTGAACAGCTCCTCGCCCT TGCTCACCATGGTGGCGGCTCTC*CCTATAGTGAGTCGTATTA*	87
**87DNA_U**^{X} [a]	F-[mG][mC][mU]CGACCAGGATGGGCACCACCCCGGTGAACAGCTCCTCGCCCT TGCTCACCATGGTGGCGGCTCTCCCU*AU*AGU*GAGU*CGU*AU*U*A	87

[a] set of modified **dU**^{X} used; [b] F = 5′- 6-FAM-labelled; [c] P = 5′- phosphorylated; [d] * position of modified nucleotide; [e] primer sequences in templates underlined; [f] T7 promoter sequence in italic; [g] [mN] 2’-OMe; DNA – double stranded

The reaction mixture (30 µL) contained primer **Prim**^{70ON}**-FAM** (2.5 µM), template **Temp**^{87ON}**-P** (2.5 µM), natural dNTPs (dATP, dGTP and dCTP, 200 µM each), appropriate **dU**^{X}**TP**

(for natural DNA dTTP, 200 µM; 300 µM for **dU^ac^TP**; 400 µM for **dU^{glu}TP**, **dU^{am}TP**, **dU^{mm}TP**), betaine (only for **dU^{glu}TP**, **dU^{am}TP**, **dU^{mm}TP**, 1 M**)**, KOD XL polymerase reaction buffer (3 µL) and KOD XL DNA polymerase (1.5 U; 3.75 U for **dU^{glu}TP**, **dU^{am}TP**, **dU^{mm}TP**). The reactions were then subjected to the following thermal cycler program: 95 °C for 5 min, followed by 55 °C for 10 min and then 72 °C for 5 min. The reactions were purified using MinElute PCR purification kit (eluted in 15 µL of 5 mM Tris-Cl buffer, pH = 8.5). 1µL of each sample was pipetted into a mixture of 10µL PAGE stop solution and 9 µL of H_2O, followed by denaturation at 95 °C for 5 min. Samples were separated with a 12.5% denaturing PAGE gel and visualized using fluorescence imaging (Figure S6). The products (**87DNA** and **87DNA_U^{X}**) were further analyzed by LC-MS (section 2.5.3., Table S12, Figures S103 – S124) and used for:
1) Preparation of **107DNA_A** and **107DNA_A_U^{X}** by ligation (section 2.7.3.)

2) Preparation of **87ON** and **87ON_U^{X}** by Lambda exonuclease digestion (section 2.6.1.)

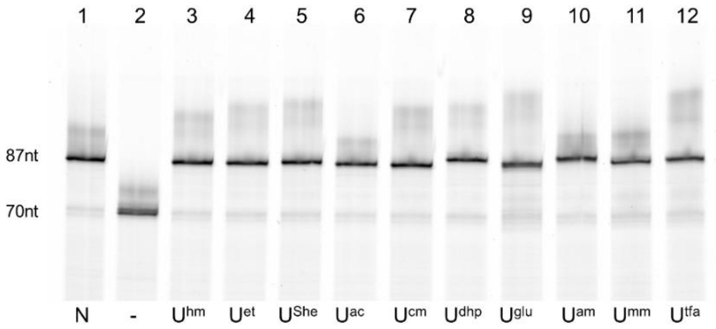

Figure S6. dPAGE analysis of PEX products using KOD XL DNA polymerase, template **Temp^{87ON}-P** and primer **Prim^{70ON}-FAM**. Lane 1 (N): product with all natural dNTPs; lane 2 (-): product in absence of dTTP and **dU^{X}TP**; lanes 3-12: products in presence of appropriate **dU^{X}TP** and the remaining natural dNTPs.

2.5.3. Preparation of 87DNA and 87DNA_C^{X} by PEX

Table S11. Used oligonucleotides and prepared dsDNA

Name	Sequence (5'→ 3')[b, c, d, e, f, g]	Length (nt)
**Prim^{68ON}-FAM**	F-[mG][mC][mU]CGACCAGGATGGGCACCACCCCGGTGAACAGCTCCTCGCCCT TGCTCACCATGGTGGCGGCTCTC	68
**Temp^{87ON}-P**	P-*TAATACGACTCACTATAGG*GAGAGCCGCCACCATGGTGAGCAAGGGCGAGGAG CTGTTCACCGGGGTGGTGCCCATCCTGGTCGAGC	87
**87DNA**	F-[mG][mC][mU]CGACCAGGATGGGCACCACCCCGGTGAACAGCTCCTCGCCCT TGCTCACCATGGTGGCGGCTCTC*CCTATAGTGAGTCGTATTA*	87
**87DNA_C^{X}**[a]	F-[mG][mC][mU]CGACCAGGATGGGCACCACCCCGGTGAACAGCTCCTCGCCCT TGCTCACCATGGTGGCGGCTCTC**C*C*** TATAGTGAGT**C*** GTATTA	87

[a] set of modified **dC^{X}** used; [b] F = 5′- 6-FAM-labelled; [c] P = 5′- phosphorylated; [d] * position of modified nucleotide; [e] primer sequences in templates underlined; [f] T7 promoter sequence in italic; [g] [mN] 2'-OMe; DNA – double stranded

The reaction mixture (30 µL) contained primer **Prim^{68ON}-FAM** (2.5 µM), template **Temp^{87ON}-P** (2.5 µM), natural dNTPs (dATP, dGTP and dTTP, 200 µM each), appropriate **dC^{X}TP** (for natural DNA dCTP, 200 µM), KOD XL polymerase reaction buffer (3 µL) and KOD XL DNA polymerase (1.5 U). The reactions were then subjected to the following thermal cycler program: 95 °C for 5 min, followed by 55 °C for 10 min and then 72 °C for 5 min. The reactions were purified using MinElute PCR purification kit (eluted in 15 µL of 5 mM Tris-Cl buffer, pH = 8.5). 1µL of each sample was pipetted into a mixture of 10µL PAGE stop solution and 9 µL of H_2O, followed by denaturation at 95 °C for 5 min. Samples were analyzed on a 12.5% denaturing PAGE gel and visualized using fluorescence imaging (Figure S7). The products (**87DNA_C^{X}**) were further analyzed by LC-MS (Table S12, Figures S125 – S132) and used for preparation of **107DNA_A** and **107DNA_A_C^{X}** by ligation (section 2.7.3.).

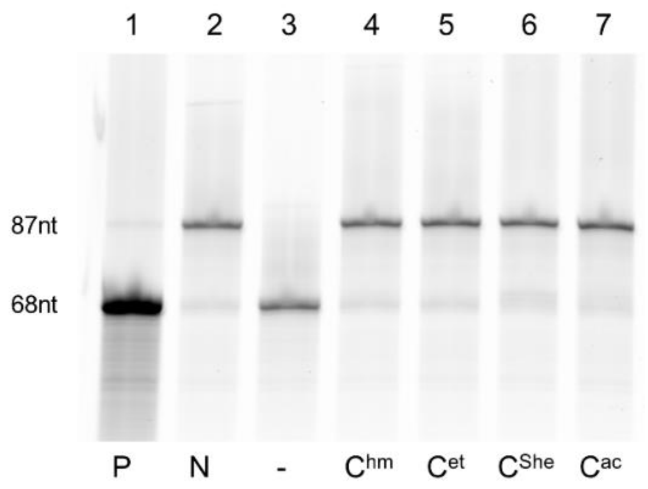

Figure S7. dPAGE analysis of PEX products using KOD XL DNA polymerase, template **Temp^{87ON}-P** and primer **Prim^{68ON}-FAM**. Lane 1 (P): **Prim^{68ON}-FAM**; lane 2 (N): product with all natural dNTPs; lane 3 (-): product in absence of dCTP and **dC^{X}TP**; lanes 4-7: products in presence of appropriate **dC^{X}TP** and the remaining natural dNTPs.

Table S12. Summary of LC-MS measurements of 87DNA/87DNA_U^{X}/87DNA_C^{X}

Name	Mw (modified strand) calculated [Da]	Mw (modified strand) found [Da]	Δ [Da]	Figure number
**87DNA**	27276.6	27267.5	9.1	103, 104
**87DNA_U^{hm}**	27388.6	27379.0	9.6	105, 106
**87DNA_U^{et}**	27374.8	27366.0	8.8	107, 108
**87DNA_U^{She}**	27486.8	27478.0	8.8	109, 110
**87DNA_U^{ac}**	27472.7	27463.5	9.2	111, 112
**87DNA_U^{cm}**	27451.7	27441.5	10.2	113, 114
**87DNA_U^{dhp}**	27893.4	27884.0	9.4	115, 116
**87DNA_U^{glu}**	28292.4	27540.0^{a}	–	117, 118
**87DNA_U^{am}**	27381.7	27369.5	12.2	119, 120
**87DNA_U^{mm}**	27479.9	26837.5^{b}	–	121, 122
**87DNA_U^{tfa}**	28053.8	28045.5	8.3	123, 124
**87DNA_C^{hm}**	27366.7	27364.0	2.7	125, 126
**87DNA_C^{et}**	27360.8	27357.5	3.3	127, 128
**87DNA_C^{She}**	27408.8	27405.5	3.3	129, 130
**87DNA_C^{ac}**	27402.7	27401.0	1.7	131, 132

^{a} Mw assigned to modified strand lacking **dU^{glu}** and dA; ^{b} Mw assigned to modified strand lacking **dU^{mm}** and dA

2.6. Preparation of single stranded DNA

2.6.1. Preparation of 37ON/37ON_U^{X} and 87ON/87ON_U^{X} by Lambda exonuclease digestion of 37DNA/37DNA_U^{X} and 87DNA/87DNA_U^{X}

Table S13. Used dsDNA and prepared ssDNA products

Name	Strand	Sequence (5‘→ 3‘) [b, c. d, e]	Length (nt)
**37DNA**		F-GACATCATGAGAGACATCGC*TAATACGACTCACTATA*	37
**87DNA**		F-[mG][mC][mU]CGACCAGGATGGGCACCACCCCGGTGAACAG<br>CTCCTCGCCCTTGCTCACCATGGTGGCGGCTCTC*CCTATAGT*<br>*GAGTCGTATTA*	87
**37DNA_U^{X}** [a]		F-GACATCATGAGAGACATCGCU*AAU*ACGACU*CACU*AU*A	37
**87DNA_U^{X}** [a]		F-[mG][mC][mU]CGACCAGGATGGGCACCACCCCGGTGAACAG<br>CTCCTCGCCCTTGCTCACCATGGTGGCGGCTCTCCCU*AU*A<br>GU*GAGU*CGU*AU*U*A	87
**37ON**	sense	F-GACATCATGAGAGACATCGC*TAATACGACTCACTATA*	37
**87ON**	antisense	F-[mG][mC][mU]CGACCAGGATGGGCACCACCCCGGTGAACAG<br>CTCCTCGCCCTTGCTCACCATGGTGGCGGCTCTC*CCTATAGT*<br>*GAGTCGTATTA*	87
**37ON_U^{X}** [a]	sense	F-GACATCATGAGAGACATCGCU*AAU*ACGACU*CACU*AU*A	37
**87ON_U^{X}** [a]	antisense	F-[mG][mC][mU]CGACCAGGATGGGCACCACCCCGGTGAACAG<br>CTCCTCGCCCTTGCTCACCATGGTGGCGGCTCTCCCU*AU*A<br>GU*GAGU*CGU*AU*U*A	87

[a] set of modified **dU^{X}** used; [b] F = 5′- 6-FAM-labelled; [c] * position of modified nucleotide; [d] T7 promoter sequence in italic; [e] [mN] 2’-OMe; ON – single stranded; DNA – double stranded

Reaction mixture (30 µL) contained Lambda exonuclease buffer (3 µL), dsDNA with one strand 5’-phosphorylated and the complementary strand 5’-FAM-labelled (either **37DNA**, **37DNA_U^{X}**, **87DNA** or **87DNA_U^{X}**, 1.2 µg) and Lambda exonuclease (1.8 U). The reaction mixture was incubated at 37 °C for 30 min and then purified by QIAquick nucleotide removal kit (eluted in 30 µL of 5 mM Tris-Cl buffer, pH = 8.5). After purification, 1 µL of each sample was pipetted into a mixture of 2 µL 6X native PAGE loading dye and 7 µL of H_2O. Samples were analyzed on a 12.5% native PAGE gel and visualized using fluorescence imaging (Figure S8). The products (**37ON**, **87ON**, **37ON_U^{X}** and **87ON_U^{X}**) were used for preparation of **107DNA_P** and **107DNA_P_U^{X}** by PEX (section 2.7.4.).

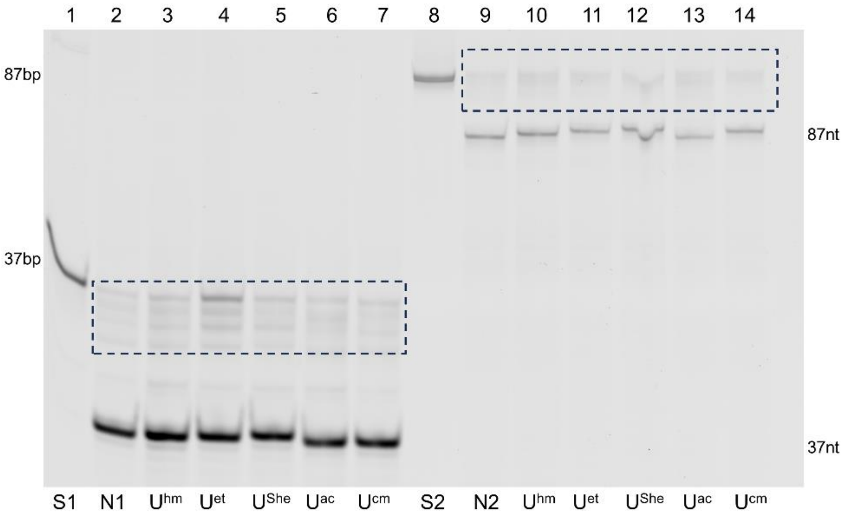

Figure S8. Native PAGE analysis of products after Lambda exonuclease treatment of **37DNA/37DNA_U^{X}** or **87DNA/87DNA_U^{X}**. Lane 1 (S1): **37DNA**; lane 2 (N1): **37ON**; lanes 3-7: **37ON_U^{X}**; lane 8 (S2): **87DNA**; lane 9 (N2): **87ON**; lanes 10-14: **87ON_U^{X}**. Traces of residual dsDNA (not fully digested) highlighted.

2.7. Preparation of DNA templates used for transcriptions

2.7.1. General notes for ligation reactions

Ligation reactions were done using T4 DNA ligase, together with in-house prepared 2X Quick ligation buffer (132 mM Tris, pH = 7.6, 20 mM $MgCl_2$, 2 mM DTT, 2 mM rATP, 15% PEG 6000). After the reaction, samples were first pre-purified by QIAquick PCR purification kit (eluted in 30 μL of 5 mM Tris-Cl buffer, pH = 8.5). 6X loading dye (10 μL) was then added to the pre-purified samples and each sample was loaded onto 3% agarose gel (SERVA agarose for PCR, molecular biology grade) and run at 5 V/cm for 2 h. DNA was visualized by blue light transilluminator and the DNA of the desired length was cut out of the gel and purified on Qiagen MinElute columns, but using buffers and protocol from E.Z.N.A. Gel Extraction Kit (eluted in 15 μL of 5 mM Tris-Cl buffer, pH = 8.5). The products were then analyzed on denaturing PAGE to confirm the length of the products as well as the presence of only partially ligated DNA. This partially ligated DNA is visible as a residual FAM signal of the used dsDNA for the ligation.

2.7.2. Preparation of 107DNA_S/107DNA_S_U^{X}/107DNA_S_C^{X} by a ligation reaction between 70DNA and 37DNA/37DNA_U^{X}/37DNA_C^{X}

Table S14. Used dsDNA and prepared ligated dsDNA products

Name	Strand	Sequence (5'→ 3') [c, d, e, f]	Length (nt)
**37DNA**		F-GACATCATGAGAGACATCGC_TAATACGACTCACTATA_	37
**37DNA_U^{X}** [a]		F-GACATCATGAGAGACATCGC_U*AAU*ACGACU*CACU*AU*A_	37
**37DNA_C^{X}** [b]		F-GACATCATGAGAGACATCGC_TAATAC*GAC*TC*AC*TATA_	37
**70DNA**		F-[mG][mC][mU]CGACCAGGATGGGCACCACCCCGGTGAACAG<br>CTCCTCGCCCTTGCTCACCATGGTGGCGGCTCTC_CC_	70
**107DNA_S**	sense	F-GACATCATGAGAGACATCGC_TAATACGACTCACTATAGG_GAG<br>AGCCGCCACCATGGTGAGCAAGGGCGAGGAGCTGTTCACC<br>GGGGTGGTGCCCATCCTGGTCGAGC	107
	antisense	F-[mG][mC][mU]CGACCAGGATGGGCACCACCCCGGTGAACAG<br>CTCCTCGCCCTTGCTCACCATGGTGGCGGCTCTC_CCTATAGT_<br>_GAGTCGTATTA_GCGATGTCTCTCATGATGTC	
**107DNA_S_U^{X}** [a]	sense	F-GACATCATGAGAGACATCGC_U*AAU*ACGACU*CACU*AU*AG_<br>_G_GAGAGCCGCCACCATGGTGAGCAAGGGCGAGGAGCTGTT<br>CACCGGGGTGGTGCCCATCCTGGTCGAGC	107
	antisense	F-[mG][mC][mU]CGACCAGGATGGGCACCACCCCGGTGAACAG<br>CTCCTCGCCCTTGCTCACCATGGTGGCGGCTCTC_CCTATAGT_<br>_GAGTCGTATTA_GCGATGTCTCTCATGATGTC	
**107DNA_S_C^{X}** [b]	sense	F-GACATCATGAGAGACATCGC_TAATAC*GAC*TC*AC*TATAGG_G<br>AGAGCCGCCACCATGGTGAGCAAGGGCGAGGAGCTGTTCAC<br>CGGGGTGGTGCCCATCCTGGTCGAGC	107
	antisense	F-[mG][mC][mU]CGACCAGGATGGGCACCACCCCGGTGAACAG<br>CTCCTCGCCCTTGCTCACCATGGTGGCGGCTCTC_CCTATAGT_<br>_GAGTCGTATTA_GCGATGTCTCTCATGATGTC	

[a] set of modified **dU^{X}** used; [b] set of modified **dC^{X}** used; [c] F = 5′- 6-FAM-labelled; [d] * position of modified nucleotide; [e] T7 promoter sequence in italic; [f] [mN] 2'-OMe; DNA – double stranded

The reaction mixture (50 µL) contained **70DNA** (0.8 µM), either **37DNA**, **37DNA_U^{X}** or **37DNA_C^{X}** (0.8 µM), T4 DNA ligase (3200 U) and Quick ligation buffer (25 µL). The reaction was incubated at 23 °C for 30 min, followed by purification procedure (see general notes, section 2.7.1.). After purification, 1µL of each sample was pipetted into a mixture of 5 µL PAGE stop solution and 4 µL of H_2O, followed by denaturing at 95 °C for 5 min. The samples were analyzed on a 12.5% dPAGE gel and visualized using fluorescence imaging (Figure S9). The products

(**107DNA_S**, **107DNA_S_U^X** and **107DNA_S_C^X**) were used for transcription study (section 2.8.2.).

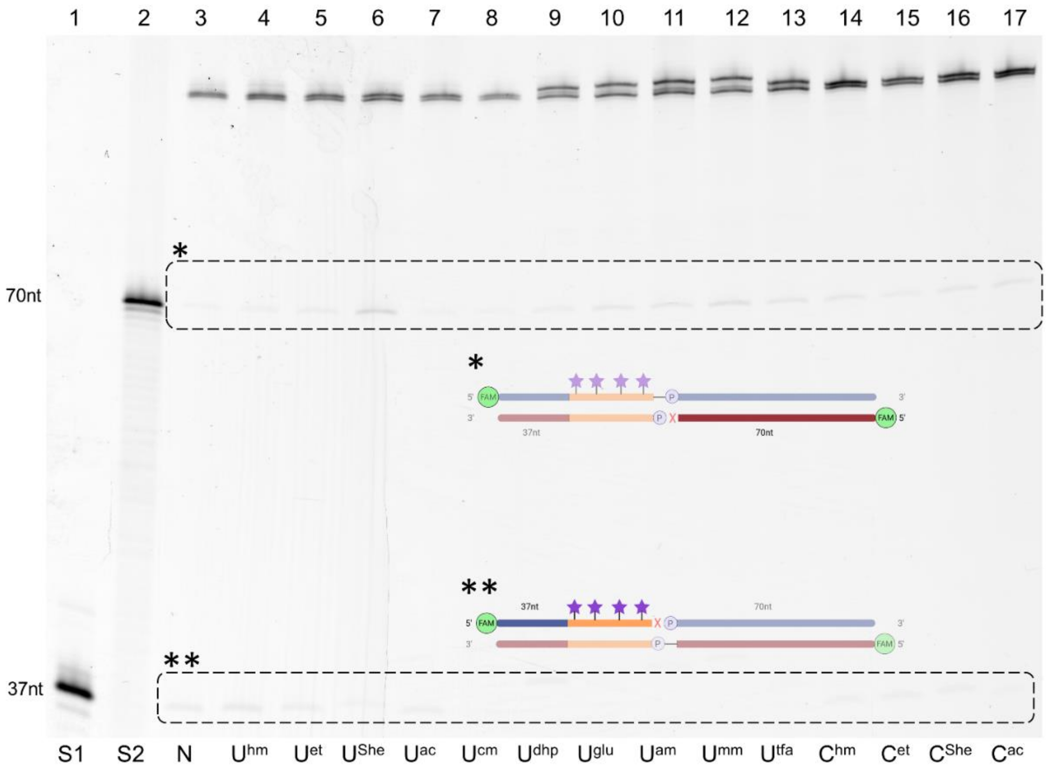

Figure S9. dPAGE analysis of ligation products using T4 DNA ligase, **70DNA** and either **37DNA**, **37DNA_U^X** or **37DNA_C^X**. Lane 1 (S1): **37DNA**; lane 2 (S2): **70DNA**; lane 3 (N): **107DNA_S**; lane 4-13: **107DNA_S_U^X**; lanes 14-17: **107DNA_S_C^X**. In all cases (lanes 3-17), negligible amount of FAM-signal from 37-mer and 70-mer is visible, suggesting presence of partially ligated template (details depicted in Figure and described in general notes).

2.7.3. Preparation of 107DNA_A/107DNA_A_U^{X}/107DNA_A_C^{X} by a ligation reaction between 20DNA and 87DNA/87DNA_U^{X}/87DNA_C^{X}

Table S15. Used dsDNA and prepared ligated dsDNA products

Name	Strand	Sequence (5'→ 3') [c, d, e, f]	Length (nt)
**20DNA**		F-GACATCATGAGAGACATCGC	20
**87DNA**		F-[mG][mC][mU]CGACCAGGATGGGCACCACCCCGGTGAACAG<br>CTCCTCGCCCTTGCTCACCATGGTGGCGGCTCTCCCTATAGT<br>GAGTCGTATTA	87
**87DNA_U^{X}** [a]		F-[mG][mC][mU]CGACCAGGATGGGCACCACCCCGGTGAACAG<br>CTCCTCGCCCTTGCTCACCATGGTGGCGGCTCTCCCU*AU*A<br>GU*GAGU*CGU*AU*U*A	87
**87DNA_C^{X}** [b]		F-[mG][mC][mU]CGACCAGGATGGGCACCACCCCGGTGAACAG<br>CTCCTCGCCCTTGCTCACCATGGTGGCGGCTCTCC*C*TATA<br>GTGAGTC*GTATTA	87
**107DNA_A**	sense	F-GACATCATGAGAGACATCGCTAATACGACTCACTATAGGGAG<br>AGCCGCCACCATGGTGAGCAAGGGCGAGGAGCTGTTCACC<br>GGGGTGGTGCCCATCCTGGTCGAGC	107
	antisense	F-[mG][mC][mU]CGACCAGGATGGGCACCACCCCGGTGAACAG<br>CTCCTCGCCCTTGCTCACCATGGTGGCGGCTCTCCCTATAGT<br>GAGTCGTATTAGCGATGTCTCTCATGATGTC	
**107DNA_A_U^{X}** [a]	sense	F-GACATCATGAGAGACATCGCTAATACGACTCACTATAGGGAG<br>AGCCGCCACCATGGTGAGCAAGGGCGAGGAGCTGTTCACC<br>GGGGTGGTGCCCATCCTGGTCGAGC	107
	antisense	F-[mG][mC][mU]CGACCAGGATGGGCACCACCCCGGTGAACAG<br>CTCCTCGCCCTTGCTCACCATGGTGGCGGCTCTCCCU*AU*A<br>GU*GAGU*CGU*AU*U*AGCGATGTCTCTCATGATGTC	
**107DNA_A_C^{X}** [b]	sense	F-GACATCATGAGAGACATCGCTAATACGACTCACTATAGGGAG<br>AGCCGCCACCATGGTGAGCAAGGGCGAGGAGCTGTTCACC<br>GGGGTGGTGCCCATCCTGGTCGAGC	107
	antisense	F-[mG][mC][mU]CGACCAGGATGGGCACCACCCCGGTGAACAG<br>CTCCTCGCCCTTGCTCACCATGGTGGCGGCTCTCC*C*TATA<br>GTGAGTC*GTATTAGCGATGTCTCTCATGATGTC	

[a] set of modified **dU^{X}** used; [b] set of modified **dC^{X}** used; [c] F = 5′- 6-FAM-labelled; [d] * position of modified nucleotide; [e] T7 promoter sequence in italic; [f] [mN] 2'-OMe; DNA – double stranded

The reaction mixture (50 µL) contained **20DNA** (0.8 µM), either **87DNA**, **87DNA _U^{X}** or **87DNA _C^{X}** (0.8 µM), T4 DNA ligase (3200 U) and Quick ligation buffer (25 µL). The reaction was incubated at 23 °C for 30 min, followed by purification procedure (see general notes, section

2.7.1.). After purification, 1 µL of each sample was pipetted into a mixture of 5 µL PAGE stop solution and 4 µL of H_2O, followed by denaturing at 95 °C for 5 min. Samples were analyzed on a 12.5% denaturing PAGE gel and visualized using fluorescence imaging (Figure S10). The products (**107DNA_A**, **107DNA_A_U^X** and **107DNA_A_C^X**) were used for transcription study (section 2.8.3.).

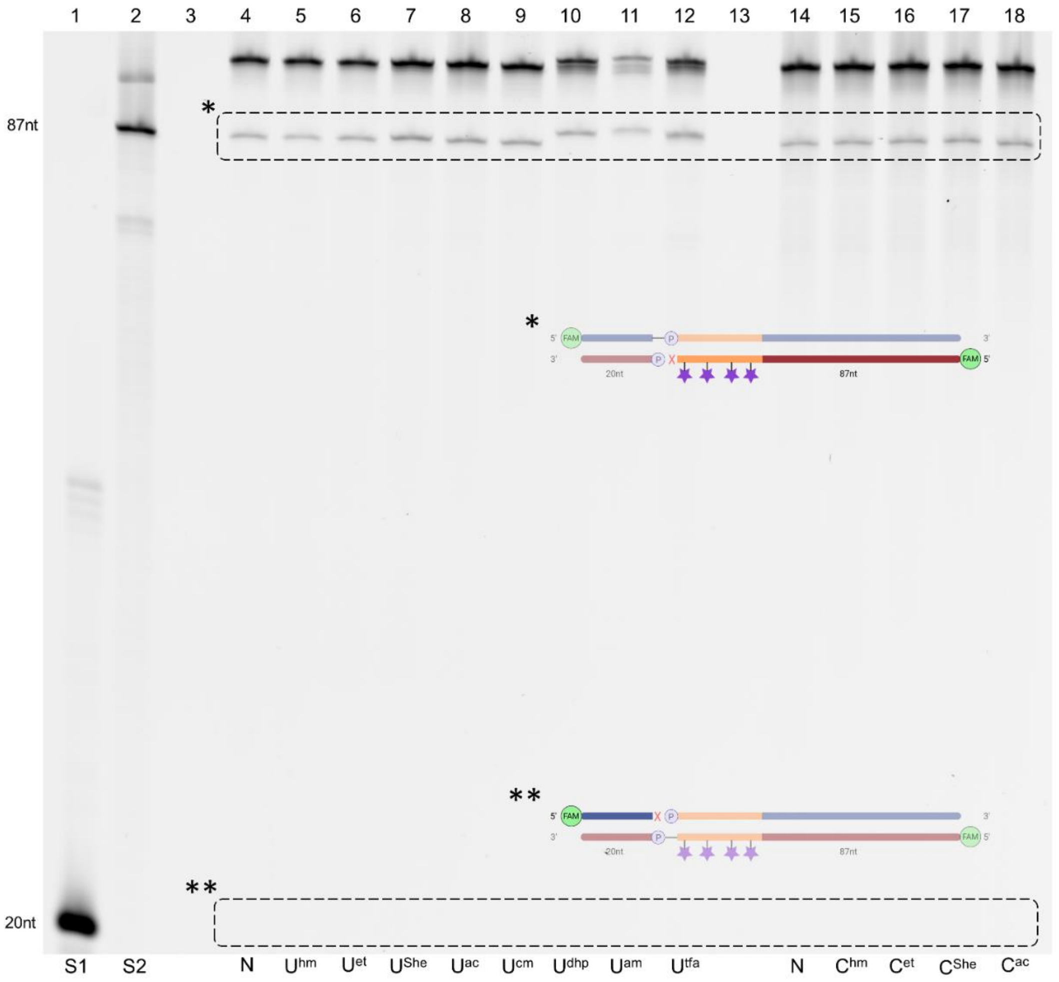

Figure S10. dPAGE analysis of ligation products using T4 DNA ligase, **20DNA** and either **87DNA**, **87DNA_U^X** or **87DNA_C^X**. Lane 1 (S1): **Prim^{20ON}-FAM** ; lane 2 (S2): **87DNA**; lanes 3 and 13: blank space; lanes 4 and 14 (N): **107DNA_A**; lanes 5-12: **107DNA_A_U^X**; lanes 15-18: **107DNA_A_C^X**. In all cases (lanes 4-12, 14-18), small amount of FAM-signal from 87-mer is visible, suggesting presence of partially ligated template (details depicted in Figure and described in general notes).

2.7.4. Preparation of 107DNA_P and 107DNA_P_U^{X} by PEX

Table S16. Used oligonucleotides and prepared dsDNA

Name	Strand	Sequence (5‘→ 3‘) [b, c, d, e, f]	Length (nt)
**37ON**	sense	F-GACATCATGAGAGACATCGC*TAATACGACTCACTATA*	37
**87ON**	antisense	F-[mG][mC][mU]CGACCAGGATGGGCACCACCCCGGTGAACAG CTCCTCGCCCTTGCTCACCATGGTGGCGGCTCTC*CCTATAGT GAGTCGTATTA*	87
**37ON_U^{X}** [a]	sense	F-GACATCATGAGAGACATCGCU*AAU*ACGACU*CACU*AU*A	37
**87ON_U^{X}** [a]	antisense	F-[mG][mC][mU]CGACCAGGATGGGCACCACCCCGGTGAACAG CTCCTCGCCCTTGCTCACCATGGTGGCGGCTCTCCCU*AU*A GU*GAGU*CGU*AU*U*A	87
**107DNA_P**	sense	F-GACATCATGAGAGACATCGC*TAATACGACTCACTATAGG*GAG AGCCGCCACCATGGTGAGCAAGGGCGAGGAGCTGTTCACC GGGGTGGTGCCCATCCTGGTCGAGC	107
	antisense	F-[mG][mC][mU]CGACCAGGATGGGCACCACCCCGGTGAACAG CTCCTCGCCCTTGCTCACCATGGTGGCGGCTCTC*CCTATAGT GAGTCGTATTA*GCGATGTCTCTCATGATGTC	
**107DNA_P_U^{X}** [a]	sense	F-GACATCATGAGAGACATCGCU*AAU*ACGACU*CACU*AU*AG GGAGAGCCGCCACCATGGTGAGCAAGGGCGAGGAGCTGTT CACCGGGGTGGTGCCCATCCTGGTCGAGC	107
	antisense	F-[mG][mC][mU]CGACCAGGATGGGCACCACCCCGGTGAACAG CTCCTCGCCCTTGCTCACCATGGTGGCGGCTCTCCCU*AU*A GU*GAGU*CGU*AU*U*AGCGATGTCTCTCATGATGTC	

[a] set of modified **dU^{X}** used; [b] F = 5′- 6-FAM-labelled; [c] * position of modified nucleotide; [d] T7 promoter sequence in italic; [e] complementary sequence (17 nt) underlined; [f] [mN] 2’-OMe; ON – single stranded; DNA – double stranded

Primer extension reaction (30 μL) contained natural dNTPs (200 μM), **37ON** or **37ON_U^{X}** (0.25 μM), corresponding **87ON** or **87ON_U^{X}** (0.167 μM), Thermopol buffer (3 μL) and Vent (exo^{-}) DNA polymerase (2 U). The reactions were incubated at 40 °C for 30 min, followed by 60 °C for 2 min. The reactions were purified by AMPure XP beads (eluted in 20 μL of H_2O). 1μL of each sample was pipetted into a mixture of 5 μL PAGE stop solution and 4 μL of H_2O, followed by denaturing at 95 °C for 5 min. Samples were analyzed on a 12.5% denaturing PAGE gel and visualized using fluorescence imaging (Figure S11). The products (**107DNA_P** and **107DNA_P_U^{X}**) were used for transcription study (section 2.8.4.).

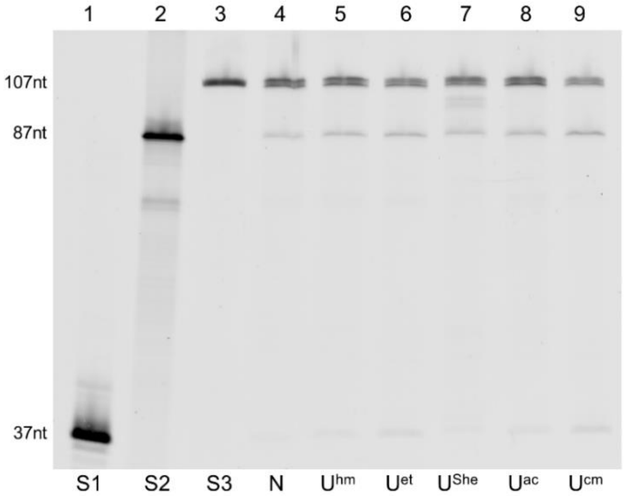

Figure S11. dPAGE analysis of PEX products using combination of **37ON**/**37ON_U^{X}** and **87ON**/**87ON_U^{X}** with Vent (exo^{-}) DNA polymerase. Lane 1 (S1): **37DNA**; lane 2 (S2): **87DNA**; lane 3 (S3): **107DNA_S**; lane 4 (N): **107DNA_P**; lanes 5-9: appropriate **107DNA_P_U^{X}**.

2.7.5. Preparation of 107DNA_F and 107DNA_F_U^X by PCR

Table S17. Used primers and prepared dsDNA products

Name	Strand	Sequence (5'→ 3') [b, c, d, e]	Length (nt)
**Prim^{20ON}-FAM**		F-GACATCATGAGAGACATCGC	20
**Prim^{16ON}-FAM**		F-GCTCGACCAGGATGGG	16
**107DNA_S**	sense	F-GACATCATGAGAGACATCGC*TAATACGACTCACTATAGG*GAG<br>AGCCGCCACCATGGTGAGCAAGGGCGAGGAGCTGTTCACC<br>GGGGTGGTGCCCATCCTGGTCGAGC	107
	antisense	F-GCTCGACCAGGATGGGCACCACCCCGGTGAACAG<br>CTCCTCGCCCTTGCTCACCATGGTGGCGGCTCTC*CCTATAGT*<br>*GAGTCGTATTA*GCGATGTCTCTCATGATGTC	
**107DNA_F**	sense	F-GACATCATGAGAGACATCGC*TAATACGACTCACTATAGG*GAG<br>AGCCGCCACCATGGTGAGCAAGGGCGAGGAGCTGTTCACC<br>GGGGTGGTGCCCATCCTGGTCGAGC	107
	antisense	F-GCTCGACCAGGATGGGCACCACCCCGGTGAACAG<br>CTCCTCGCCCTTGCTCACCATGGTGGCGGCTCTC*CCTATAGT*<br>*GAGTCGTATTA*GCGATGTCTCTCATGATGTC	
**107DNA_F_U^X** [a]	sense	F-GACATCATGAGAGACATCGCU*AAU*ACGACU*CACU*AU*AG<br>GGAGAGCCGCCACCAU*GGU*GAGCAAGGGCGAGGAGCU*G<br>U*U*CACCGGGGU*GGU*GCCCAU*CCU*GGU*CGAGC	107
	antisense	F-GCTCGACCAGGATGGGCACCACCCCGGU*GAACAGCU*CCU*<br>CGCCCU*U*GCU*CACCAU*GGU*GGCGGCU*CU*CCCU*AU*A<br>GU*GAGU*CGU*AU*U*AGCGAU*GU*CU*CU*CAU*GAU*GU*C	

[a] set of modified **dU^X** used; [b] F = 5′- 6-FAM-labelled; [c] * position of modified nucleotide; [d] primer sequences in templates underlined; [e] T7 promoter sequence in italic; DNA – double stranded

The PCR mixture (20 μL) contained primers (**Prim^{20ON}-FAM** and **Prim^{16ON}-FAM**, each 1 μM), mixture of three natural dNTPs (dATP, dGTP, dCTP; 200 μM) and **dU^{X}TP** (for natural DNA dTTP, 200 μM; 300 μM for d**U^{She}TP** and **dU^{ac}TP**), template **107DNA_S** (prepared in section 2.7.2.; 1 ng/μL, 1 μL), KOD XL buffer (2 μL) and KOD XL DNA polymerase (1 U). All reaction mixtures were run under following cycling conditions: preheating at 95 °C for 3 min, followed by 25 cycles of denaturation at 95 °C for 30 sec, annealing at 52 °C for 15 sec and extension at 72 °C for 1 min, finished by final extension at 74 °C for 5 min. The reactions were purified using QIAquick PCR purification kit (eluted in 30 μL of 5 mM Tris-Cl buffer, pH = 8.5). 1μL of each sample was pipetted into a mixture of 10 μL PAGE stop solution and 9 μL of H_2O, followed by denaturation at 95 °C for 5 min. Samples were analyzed on a 12.5%

denaturing PAGE gel and visualized using fluorescence imaging (Figure S12). The products (**107DNA_F** and **107DNA_F_U^{X}**) were used for transcription study (section 2.8.5.).

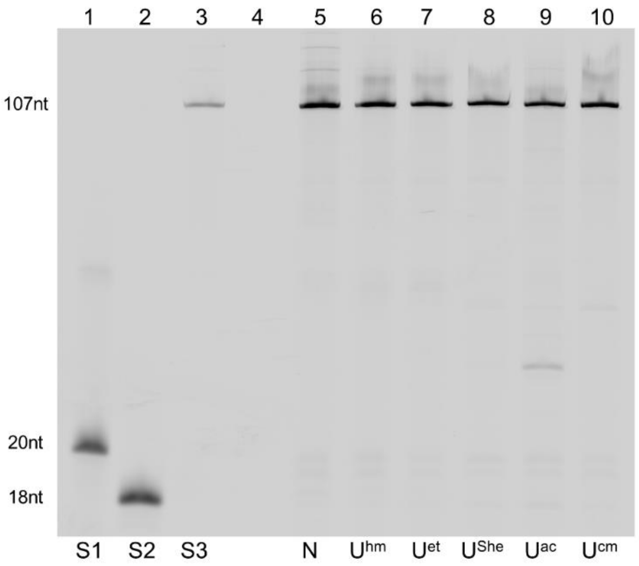

Figure S12. dPAGE analysis of PCR products using template **107DNA_S** amplified by KOD XL DNA polymerase. Lane 1 (S1): **Prim^{20ON}-FAM**; lane 2 (S2): **Prim^{16ON}-FAM**; lane 3 (S3): **107DNA_S**; lane 4: blank space; lane 5 (N): all natural dNTPs; lanes 6-10: appropriate **dU^{X}TP** with remaining natural dNTPs.

2.8. Multiple round transcription experiments

2.8.1. Quantification of templates used in *in vitro* transcriptions

Concentration of the natural DNA template (**107DNA_S**, **107DNA_A**, **107DNA_P** or **107DNA_F**) from the set of prepared templates was measured by NanoDrop and used as the standard for quantification of the modified templates. Samples were run on agarose gel (2%, 0.5X TBE) and then quantified by 6-FAM fluorescence using labelled primers, using ImageJ software. This quantification was done at least in duplicate.

2.8.2. Transcription reaction using 107DNA_S and 107DNA_S_U^{X}/107DNA_S_C^{X} containing sense-modified promoter

In vitro transcription reaction (20 μL) contained T7 transcription buffer (4 μL), $MgCl_2$ (25 mM), Triton X-100 (0.1%), rNTPs mix (2 mM), RiboLock RNase Inhibitor (20 U), either natural or

modified DNA template (**107DNA_S**, **107DNA_S_U^{X}** or **107DNA_S_C^{X}**; 10 ng, prepared according to section 2.7.2.) and T7 RNA polymerase (20 U). Once the template was added, the reactions were incubated at 37 °C for 1 h. DNase I (2 U) was then added and the reactions were further incubated at 37 °C for 15 min, followed by the addition of stop solution (40 µL) and H_2O (20 µL). The samples were denatured (65 °C for 10 min), chilled on ice and 5 µL was loaded onto 12.5 % denaturing PAGE which was run at 25 mA for 50 min. The gel was stained with SYBR Gold (diluted to 1X in 1X TBE buffer) for 15 min, scanned (Figure S13) and the relative amount of RNA quantified. The whole procedure was done in triplicate and the results were averaged (Figure S14).

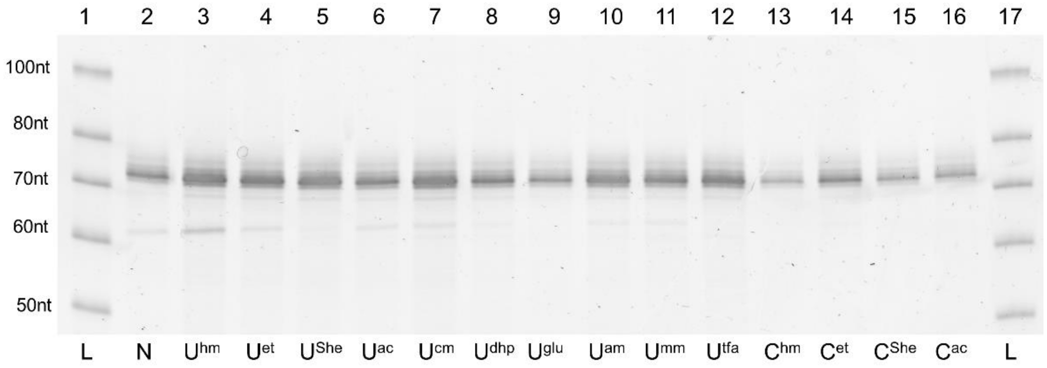

Figure S13. dPAGE analysis of transcription products using templates **107DNA_S**, **107DNA_S_U^{X}** or **107DNA_S_C^{X}**. Lane 1 and 17 (L): RNA ladder; lane 2 (N): RNA produced from **107DNA_S**; lanes 3-12: RNA produced from **107DNA_S_U^{X}**; lanes 13-16: RNA produced from **107DNA_S_C^{X}**. Gel stained with 1X SYBR Gold.

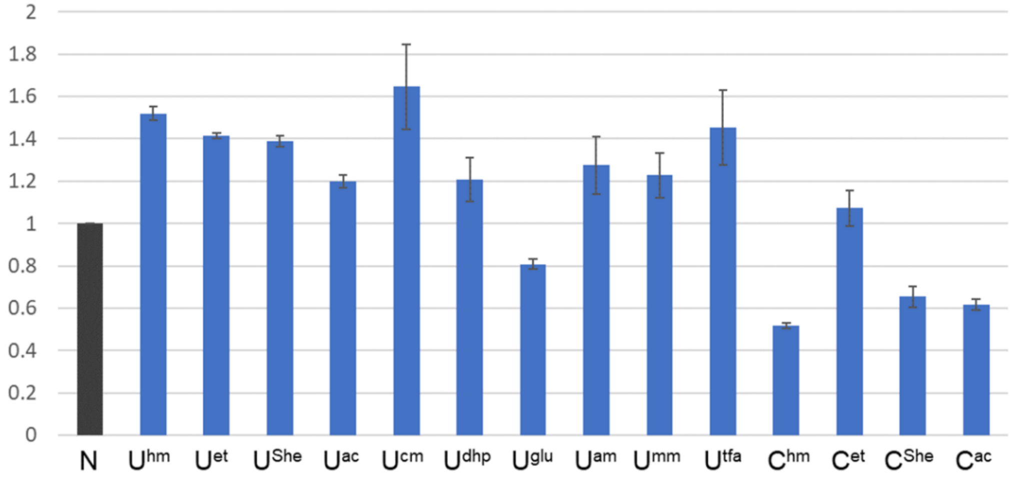

Figure S14. Relative transcription rates with templates containing sense-modified promoter. N – natural DNA.

2.8.3. Transcription reaction using 107DNA_A and 107DNA_A_U^X/107DNA_A_C^X containing antisense-modified promoter

In vitro transcription reaction (20 μL) contained T7 transcription buffer (4 μL), $MgCl_2$ (25 mM), Triton X-100 (0.1%), rNTPs mix (2 mM), RiboLock RNase Inhibitor (20 U), either natural or modified DNA template (**107DNA_A**, **107DNA_A_U^X** or **107DNA_A_C^X**; 10 ng, prepared according to section 2.7.3.) and T7 RNA polymerase (20 U). Once the template was added, the reactions were incubated at 37 °C for 1 h. DNase I (2 U) was then added and the reactions were further incubated at 37 °C for 15 min, followed by the addition of stop solution (40 μL) and H_2O (20 μL). The samples were denatured (65 °C for 10 min), chilled on ice and 5 μL was loaded onto 12.5 % denaturing PAGE which was run at 25 mA for 50 min. The gel was stained with SYBR Gold (diluted to 1X in 1X TBE buffer) for 15 min, scanned (Figures S15 and S16) and the relative amount of RNA quantified. The whole procedure was done in triplicate and the results were averaged (Figure S17).

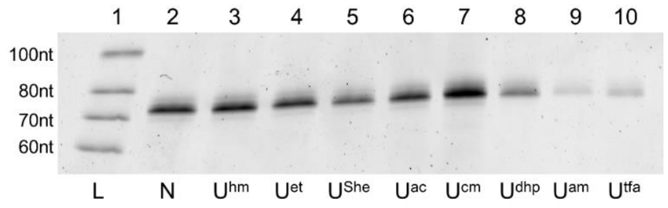

Figure S15. dPAGE analysis of transcription products using templates **107DNA_A** or **107DNA_A_U^X**. Lane 1 (L): RNA ladder; lane 2 (N): RNA produced from **107DNA_A**; lanes 3-10: RNA produced from **107DNA_A_U^X**. Gel stained with 1X SYBR Gold.

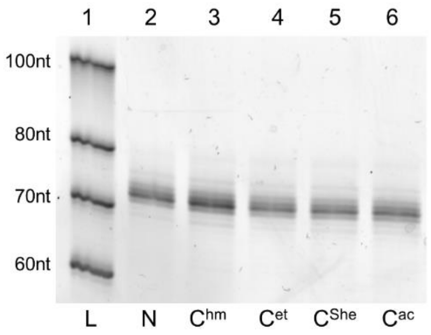

Figure S16. dPAGE analysis of transcription products using templates **107DNA_A** or **107DNA_A_C^X**. Lane 1 (L): RNA ladder; lane 2 (N): RNA produced from **107DNA_A**; lanes 3-6: RNA produced from **107DNA_A_C^X**. Gel stained with 1X SYBR Gold.

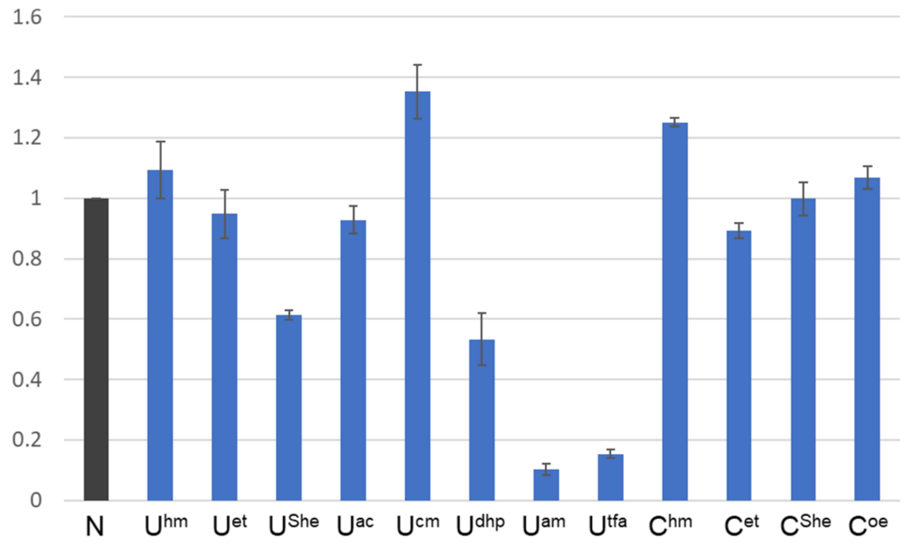

Figure S17. Relative transcription rates with templates containing antisense-modified promoter. N – natural DNA.

2.8.4. Transcription reaction using 107DNA_P and 107DNA_P_U^{X} containing fully modified promoter

In vitro transcription reaction (20 µL) contained T7 transcription buffer (4 µL), $MgCl_2$ (25 mM), Triton X-100 (0.1%), rNTPs mix (2 mM), RiboLock RNase Inhibitor (20 U), either natural or modified DNA template (**107DNA_P** or **107DNA_P_U^{X}**; 10 ng, prepared according to section 2.7.4.) and T7 RNA polymerase (20 U). Once the template was added, the reactions were incubated at 37 °C for 1 h. The reaction was stopped by the addition of stop solution (40 µL), H_2O (12 µL) and 100 mM EDTA (8 µL). The samples were denatured (95 °C for 3 min), chilled on ice and 5 µL was loaded onto 12.5 % denaturing PAGE which was run at 25 mA for 50 min. The gel was stained with SYBR Gold (diluted to 1X in 1X TBE buffer) for 15 min, scanned (Figure S18) and the relative amount of RNA quantified. The whole procedure was done in triplicate and the results were averaged (Figure S19).

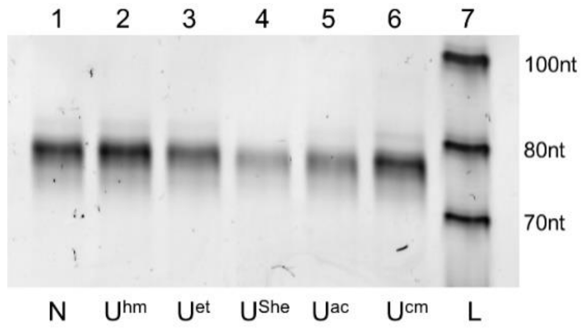

Figure S18. dPAGE analysis of transcription products using templates **107DNA_P** or **107DNA_P_U^{X}**. Lane 1 (N): RNA produced from **107DNA_P**; lanes 2-6: RNA produced from **107DNA_P_U^{X}**; lane 7 (L): RNA ladder. Gel stained with 1X SYBR Gold.

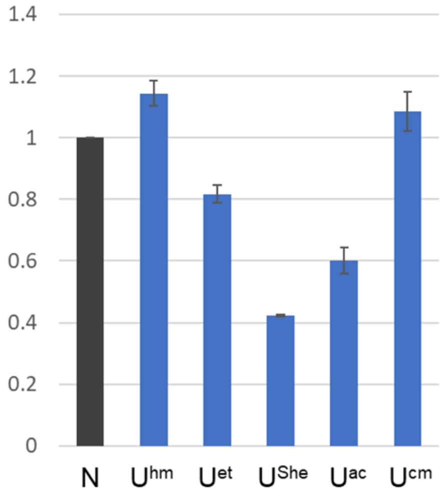

Figure S19. Relative transcription rates with templates containing fully modified promoter.
N – natural DNA.

2.8.5. Transcription reaction using 107DNA_F and fully modified 107DNA_F_U^{X}

In vitro transcription reaction (20 µL) contained T7 transcription buffer (4 µL), $MgCl_2$ (25 mM), Triton X-100 (0.1%), rNTPs mix (2 mM), RiboLock RNase Inhibitor (20 U), either natural or modified DNA template (**107DNA_F** or **107DNA_F_U^{X}**; 10 ng, prepared according to section 2.7.5.) and T7 RNA polymerase (20 U). Once the template was added, the reactions were incubated at 37 °C for 1 h. The reaction was stopped by the addition of stop solution (40 µL), H_2O (12 µL) and 100 mM EDTA (8 µL). The samples were denatured (95 °C for 3 min), chilled on ice and 5 µL was loaded onto 12.5 % denaturing PAGE which was run at 25 mA for 50 min. The gel was stained with SYBR Gold (diluted to 1X in 1X TBE buffer) for 15 min, scanned (Figure S20) and

the relative amount of RNA quantified. The whole procedure was done in triplicate and the results were averaged (Figure S21). One replicate was further used for the preparation of samples for RNA sequencing (section 2.9.).

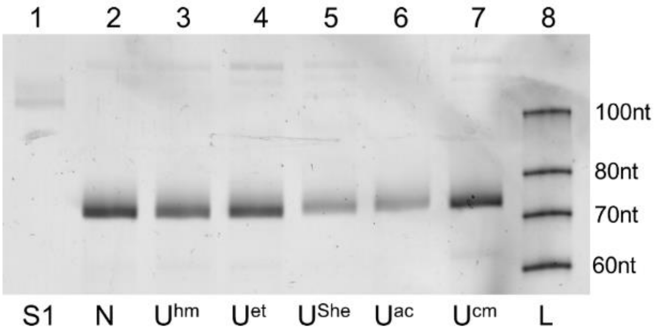

Figure S20. dPAGE analysis of transcription products using templates **107DNA_F** or **107DNA_F_U^{X}**. Lane 1 (S1): **107DNA_F**; lane 2 (N): RNA produced from **107DNA_F**; lanes 3-7: RNA produced from **107DNA_F_U^{X}**; lane 8 (L): RNA ladder. Gel stained with 1X SYBR Gold.

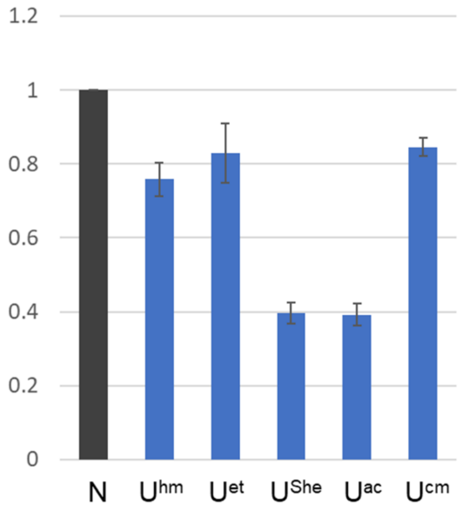

Figure S21. Relative transcription rates with fully modified templates. N – natural DNA.

2.9. Preparation of samples from RNA for next generation sequencing

2.9.1. Reverse transcription of 70RNA_F from *in vitro* transcription reaction to prepare 108RT

Table S18. Sequences for reverse transcription

Name		Sequence (5‘→ 3‘)$^{a, b, c}$	Length (nt)
**Prim_RT**^{18ON}		GCACGTAAGC*N*CGACCAG	18
**TSO**^{30ON}		AAGCAGTGGTATCAACGCAGAGTACAT**GGG**	30
**70RNA_F**		**GGGAGAGCCGCCACCAUGGUGAGCAAGGGCGAGGAGCUGUUC ACCGGGGUGGUGCCCAUCCUGGUCGAGC**	70
**108RT**	sense	AAGCAGTGGTATCAACGCAGAGTACAT**GGGGGGAGAGCCGCCAC CATGGTGAGCAAGGGCGAGGAGCTGTTCACCGGGGTGGTGCCC ATCCTGGTCGAGC**	100
	antisense	GCACGTAAGC*N*CGACCAGGATGGGCACCACCCCGGTGAACAGCT CCTCGCCCTTGCTCACCATGGTGGCGGCTCTCCCCCCATGTACTC TGCGTTGATACCACTGCTT	108

^{a} ribonucleotides in bold; ^{b} primer sequences in RNA underlined; ^{c} N – degenerated base, in italic; RNA – ribonucleic acid.

Reverse transcription mixtures (20 µL) were prepared by first mixing **Prim_RT**^{18ON} (1 µM), dNTPs mix (500 µM) and RNA **70RNA_F** (~ 30 ng each, isolated by Monarch RNA Cleanup kit from IVT reaction using **107DNA_F** and **107DNA_F_U**^{X} templates, six samples in total, section 2.8.5.) that were heated to 65 °C for 5 min and then cooled on ice. To this, 5X First-Strand buffer (4 µL), RiboLock RNase Inhibitor (30 U), DDT (10 mM) and a template-switching oligo **TSO**^{30ON} (0.75 µM) were added. The mixture was incubated at 37 °C for 2 min, followed by the addition of M-MLV reverse transcriptase (200 U). The complete mixture was heated to 37 °C for 50 min, followed by 70 °C for 15 min. Mixtures containing **108RT** were analyzed on agarose gel (3%) stained by GelRed (Figure S22) and used in the next reaction without further purification (section 2.9.2.).

2.9.2. Preparation of DNA for next generation sequencing by PCR

Table S19. Sequences for adapter and index PCRs

Name	Strand	Sequence (5‘→ 3‘) [a, b, c, d]	Length (nt)
**F_TrueSeq_TSO**		ACACTCTTTCCCTACACGACGCTCTTCCGATCTAAGCAGTGGTATCAACGCAGAG	55
**R_TrueSeq_108RT**		TGACTGGAGTTCAGACGTGTGCTCTTCCGATCTGCACGTAAGC*N*CGACCAG	51
**F_i501_tr**		AATGATACGGCGACCACCGAGATCTACACTATAGCCTACACTCTTTCCCTACACGACG	58
**R_i701_tr – R_i706_tr**		CAAGCAGAAGACGGCATACGAGATXXXXXXXXTGACTGGAGTTCAGACGTGT	53
**108RT**	sense	AAGCAGTGGTATCAACGCAGAGTACAT**GGGGGGAGAGCCGCCACCATGGTGAGCAAGGGCGAGGAGCTGTTCACCGGGGTGGTGCCCATCCTGGTCGAGC**	100
	antisense	GCACGTAAGC*N*CGACCAGGATGGGCACCACCCCGGTGAACAGCTCCTCGCCCTTGCTCACCATGGTGGCGGCTCTCCCCCCATGTACTCTGCGTTGATACCACTGCTT	108
**244DNA_NGS**	sense	AATGATACGGCGACCACCGAGATCTACACTATAGCCTACACTCTTTCCCTACACGACGCTCTTCCGATCTAAGCAGTGGTATCAACGCAGAGTACATGGGGGGAGAGCCGCCACCATGGTGAGCAAGGGCGAGGAGCTGTTCACCGGGGTGGTGCCCATCCTGGTCG*N*GCTTACGTGCAGATCGGAAGAGCACACGTCTGAACTCCAGTCAXXXXXXXXXATCTCGTATGCCGTCTTCTGCTTG	244
	antisense	CAAGCAGAAGACGGCATACGAGATXXXXXXXXTGACTGGAGTTCAGACGTGTGCTCTTCCGATCTGCACGTAAGC*N*CGACCAGGATGGGCACCACCCCGGTGAACAGCTCCTCGCCCTTGCTCACCATGGTGGCGGCTCTCCCCCCATGTACTCTGCGTTGATACCACTGCTTAGATCGGAAGAGCGTCGTGTAGGGAAAGAGTGTAGGCTATAGTGTAGATCTCGGTGGTCGCCGTATCATT	

[a] ribonucleotides in bold; [b] primer sequences underlined; [c] N – degenerated base, in italic; [d] X – variable base in index region; DNA – double stranded

To prepare the DNA for sequencing, PCR of all six samples from reverse transcription (**108RT**, section 2.9.1.) was performed to introduce the adapter followed by the index sequences. For addition of the adapter sequences, the PCR mixture with a total volume of 25 µL contained: **108RT** (1 µL of the reaction mixture), **F_TruSeq_TSO** and **R_TrueSeq_108RT** (0.5 µM each), dNTPs mix (200 µM each), 5X Q5 buffer (5 µL) and HotStart Q5 DNA Polymerase (0.5 U).The PCR reaction was performed under the following cycling conditions: preheating at 98 °C for 30 s,

followed by 15 cycles of denaturation at 98 °C for 10 sec, annealing at 67 °C for 30 sec and extension at 72 °C for 20 s, finished by final extension at 72 °C for 2 min. For PCR with index primers, the PCR mixture with a total volume of 25 µL contained: adapter-PCR mixture (2 µL used directly), **F_i501_tr** and **R_i701_tr** – **R_i706_tr** (0.5 µM each), dNTPs mix (200 µM each), 5X Q5 buffer (5 µL) and HotStart Q5 DNA Polymerase (0.5 U).The PCR reaction was performed under the following cycling conditions: preheating at 98 °C for 30 s, followed by 15 cycles of denaturation at 98 °C for 10 sec, annealing at 55 °C for 30 sec and extension at 72 °C for 30 s, finished by final extension at 72 °C for 2 min. The products of both adapter-PCR and index-PCR were first analyzed on agarose gel (3%) stained with GelRed (Figure S22) and then the index-PCR products were purified from gel as described for ligation reactions (section 2.7.1.). The purified products (**244DNA_NGS_1** to **244DNA_NGS_6**) were pooled according to the measured concentration (~100 ng each, 500 µL total) and sequenced on Illumina NovaSeq.

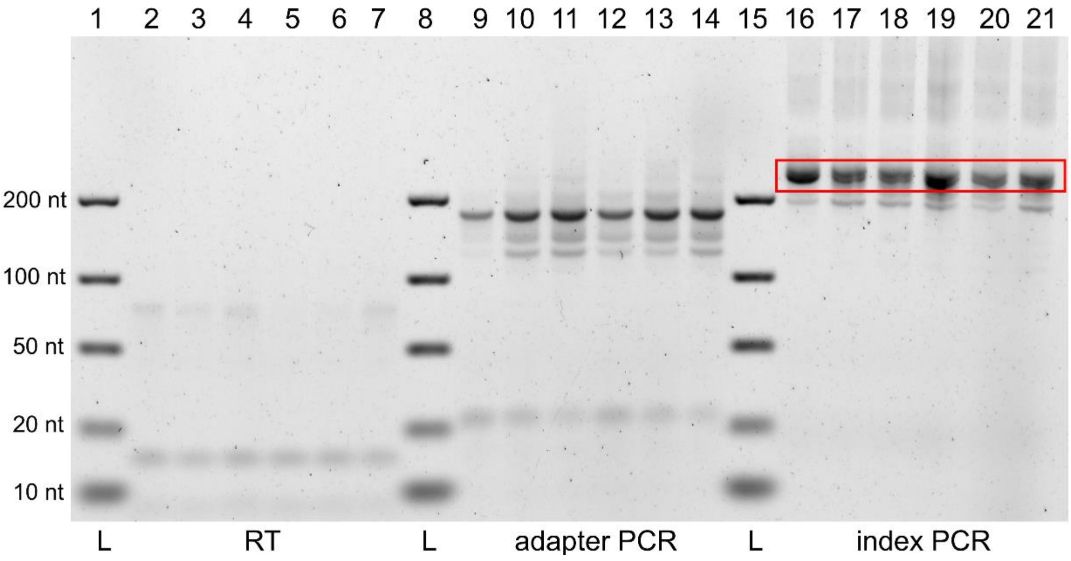

Figure S22. Agarose gel analysis of reverse transcription, adapter-PCR and index-PCR products. Lanes 1, 8, 15: ladder; lanes 2 – 7: products after reverse transcription (RT); lanes 9 – 14: products after adapter PCR; lanes 16 - 21: products after index PCR. Red rectangular shows NGS-ready products that were subsequently gel-purified.

3. LC-MS spectra

3.1. LC-MS spectra of 19DNA and 19DNA_U^X

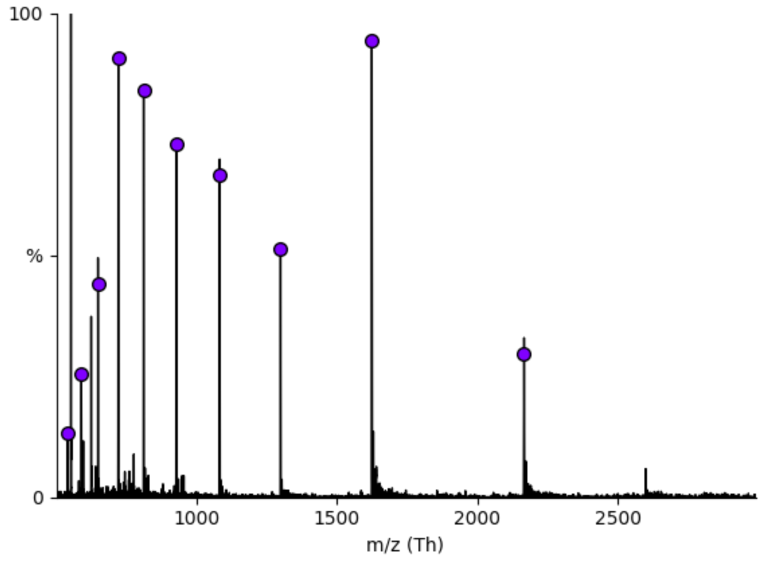

Figure S23. **19DNA**, raw spectrum.

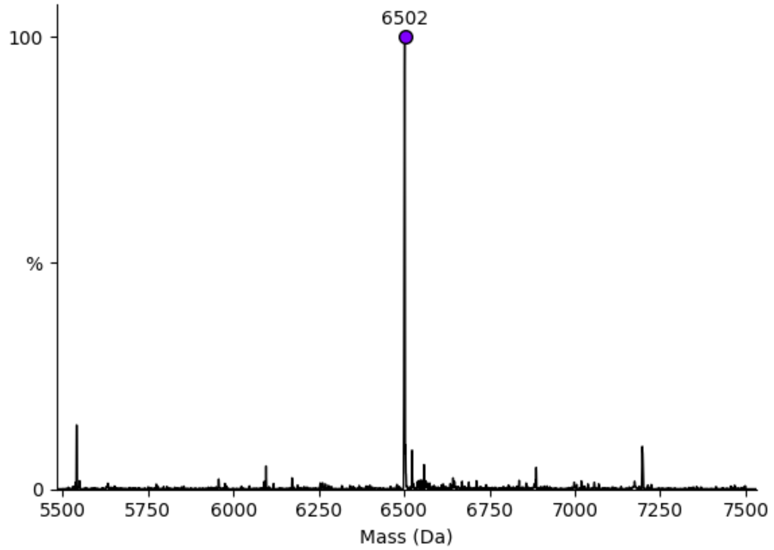

Figure S24. **19DNA**, deconvoluted spectrum, calculated mass: 6502.9 Da, found mass: 6502.0 (product).

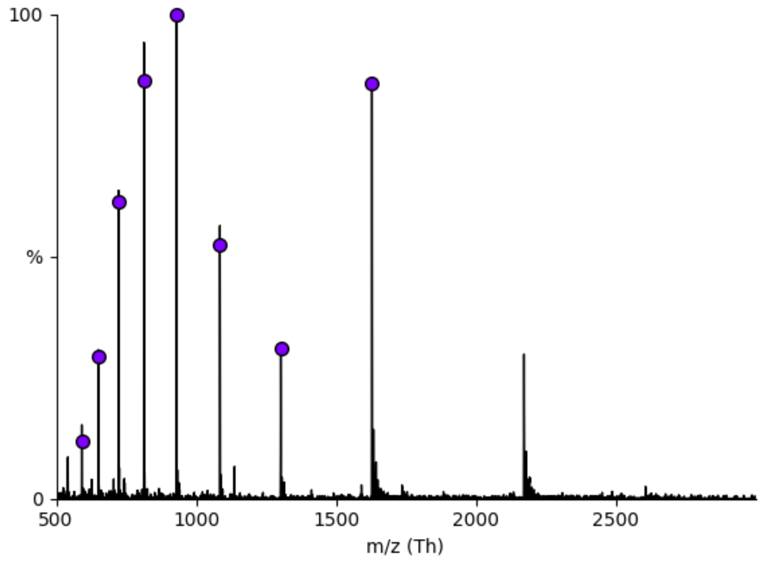

Figure S25. **19DNA_U^{am}**, raw spectrum.

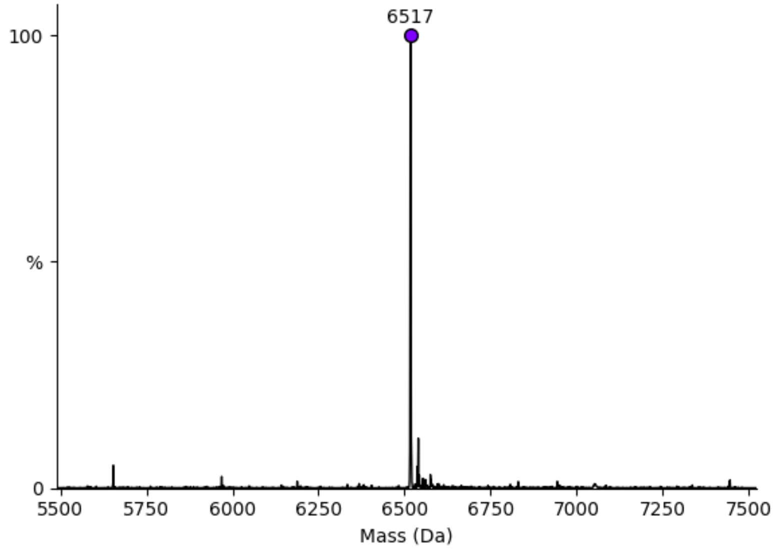

Figure S26. **19DNA_U^{am}**, deconvoluted spectrum, calculated mass: 6517.9 Da, found mass: 6517.0 (product).

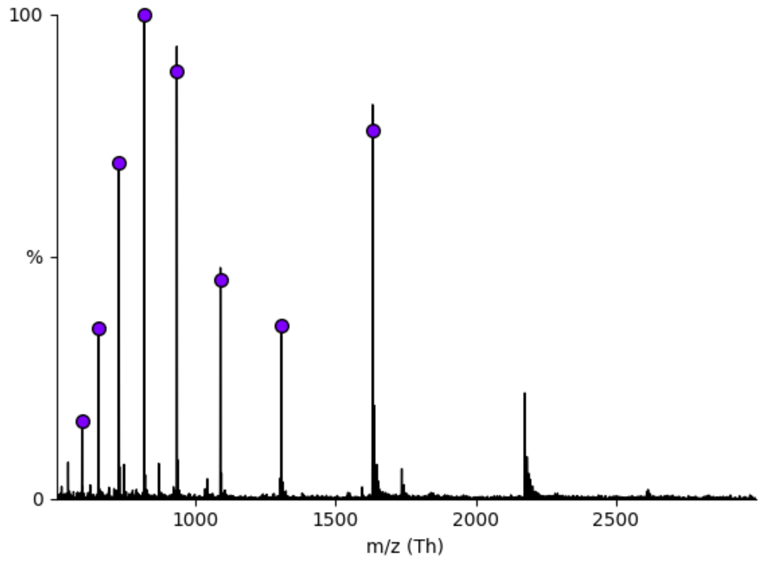

Figure S27. **19DNA_U^{mm}**, raw spectrum.

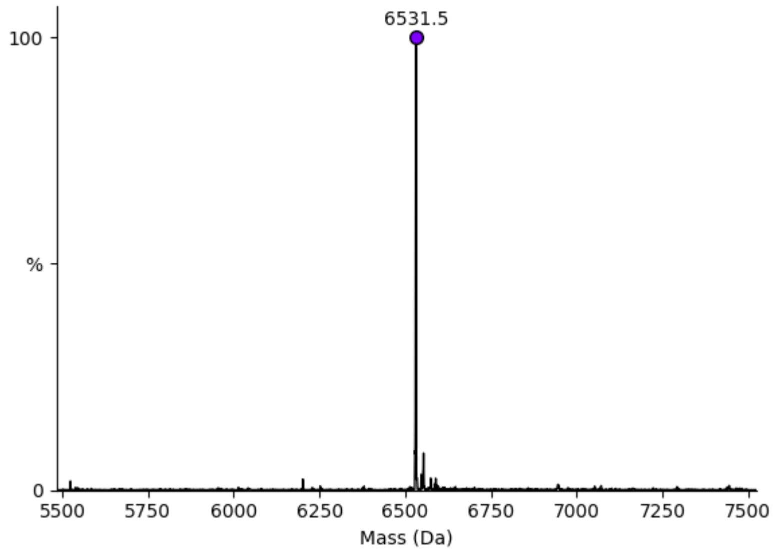

Figure S28. **19DNA_U^{mm}**, deconvoluted spectrum, calculated mass: 6531.9 Da, found mass: 6531.5 (product).

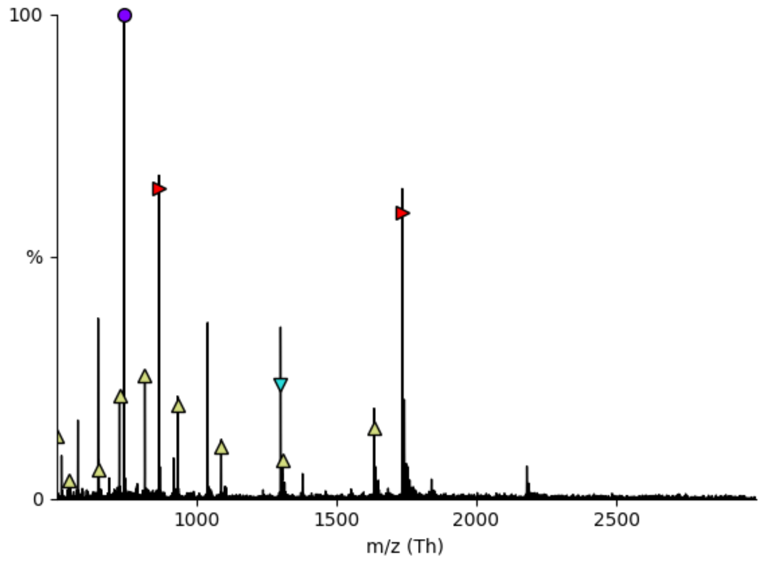

Figure S29. **19DNA_U^{dm}**, raw spectrum.

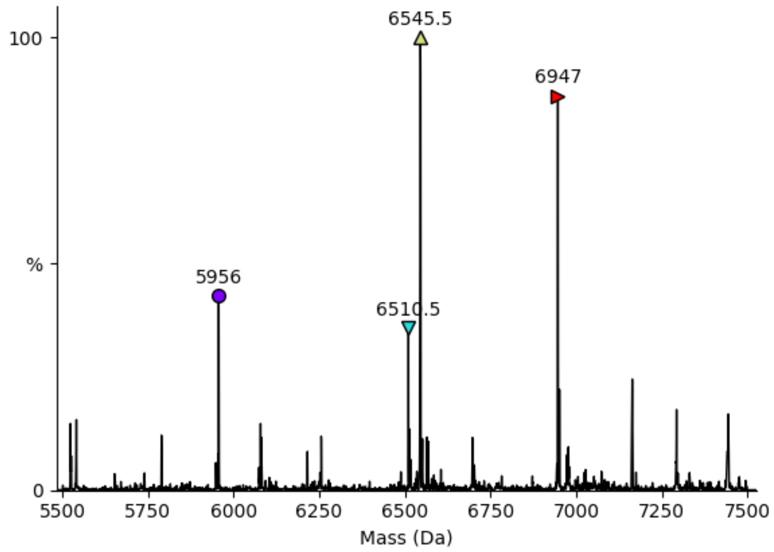

Figure S30. **19DNA_U^{dm}**, deconvoluted spectrum, calculated mass: 6546.0 Da, found mass: 6545.5 (product).

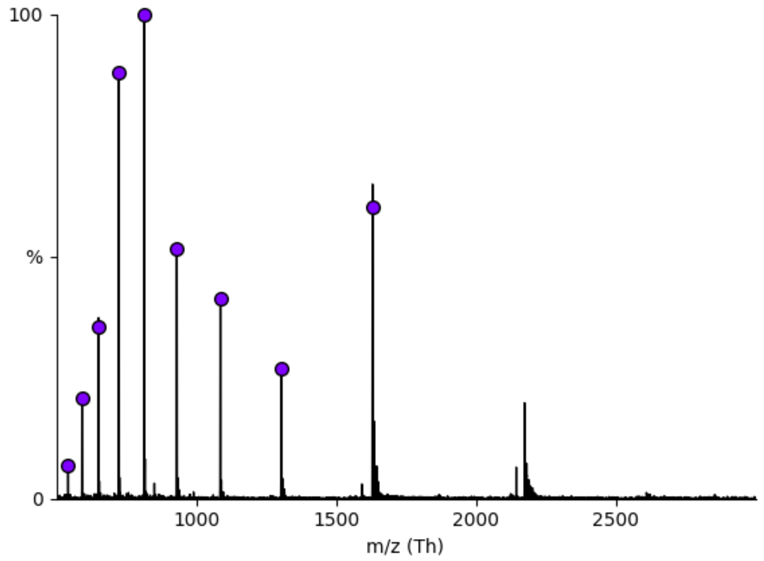

Figure S31. **19DNA_U^{cm}**, raw spectrum.

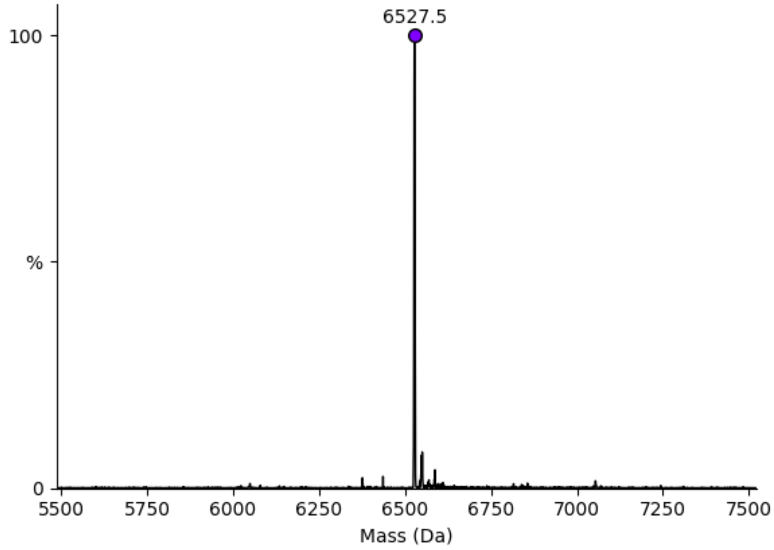

Figure S32. **19DNA_U^{cm}**, deconvoluted spectrum, calculated mass: 6527.9 Da, found mass: 6527.5 (product).

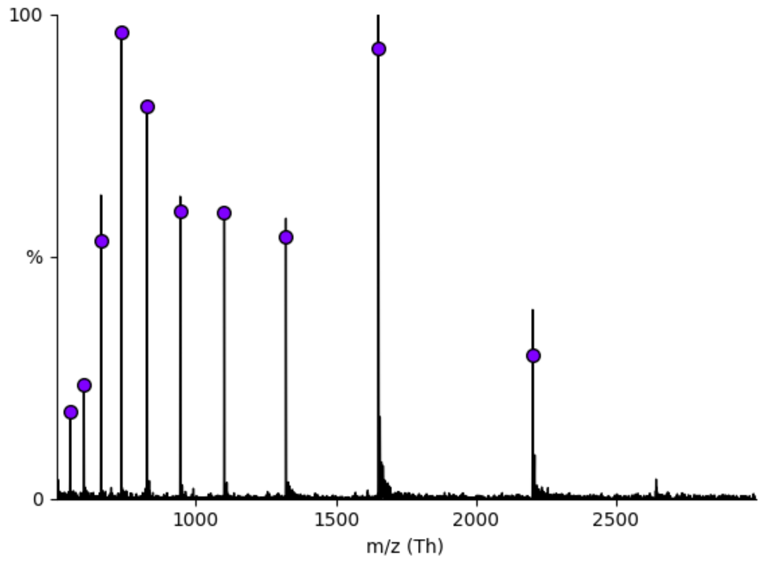

Figure S33. **19DNA_U^{tfa}**, raw spectrum.

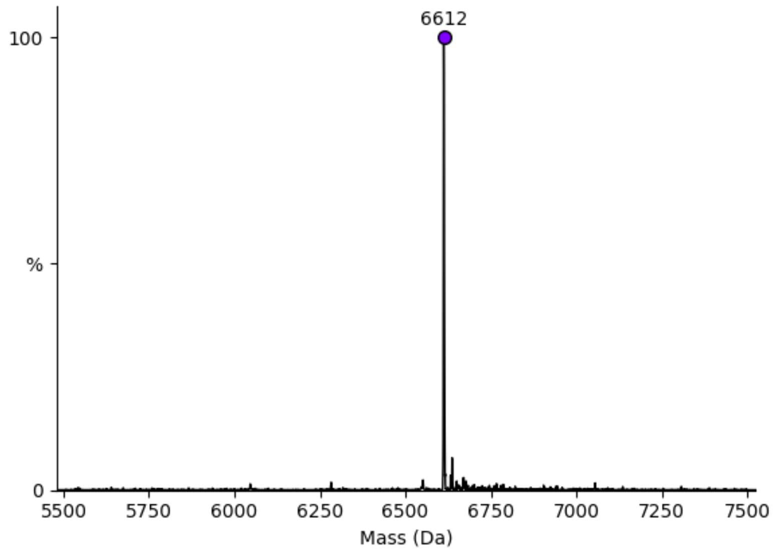

Figure S34. **19DNA_U^{tfa}**, deconvoluted spectrum, calculated mass: 6613.9 Da, found mass: 6612.0 (product).

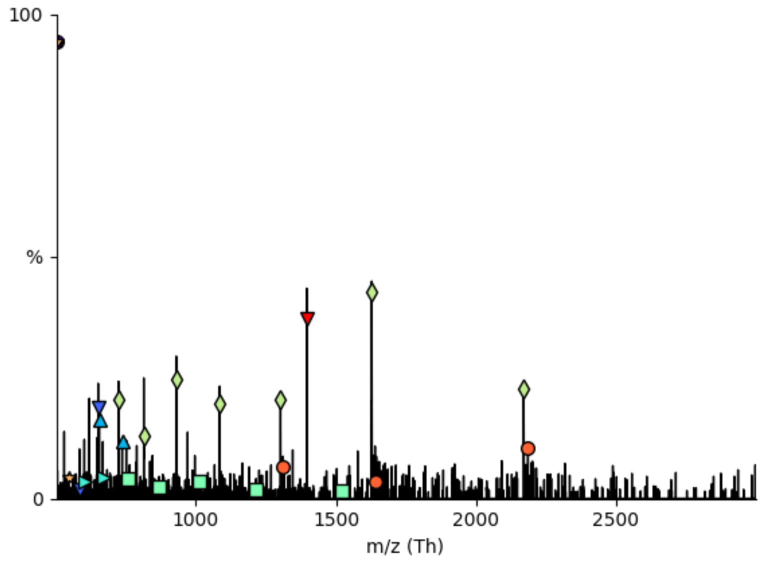

Figure S35. **19DNA_U^{tfa}**, second peak, raw spectrum.

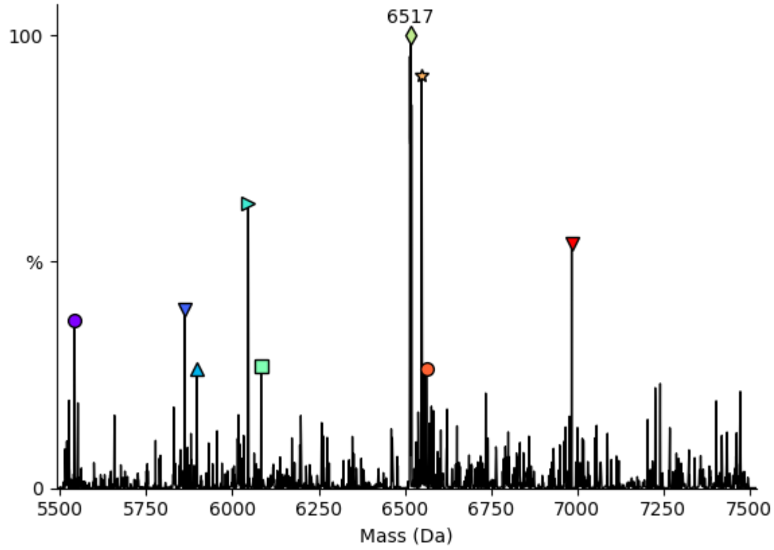

Figure S36. **19DNA_U^{tfa}**, second peak, deconvoluted spectrum, calculated mass: 6613.9 Da, found mass: 6517.0 (product lacking TFA protecting group).

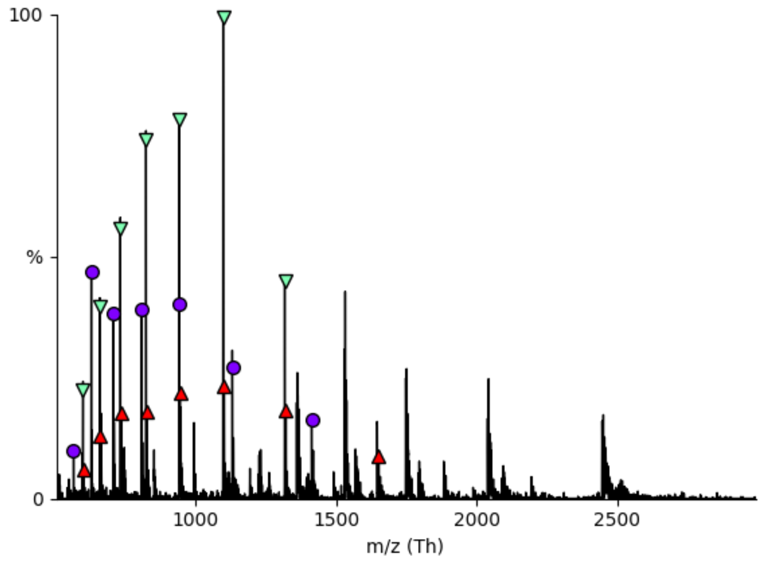

Figure S37. **19DNA_U^{put}**, raw spectrum.

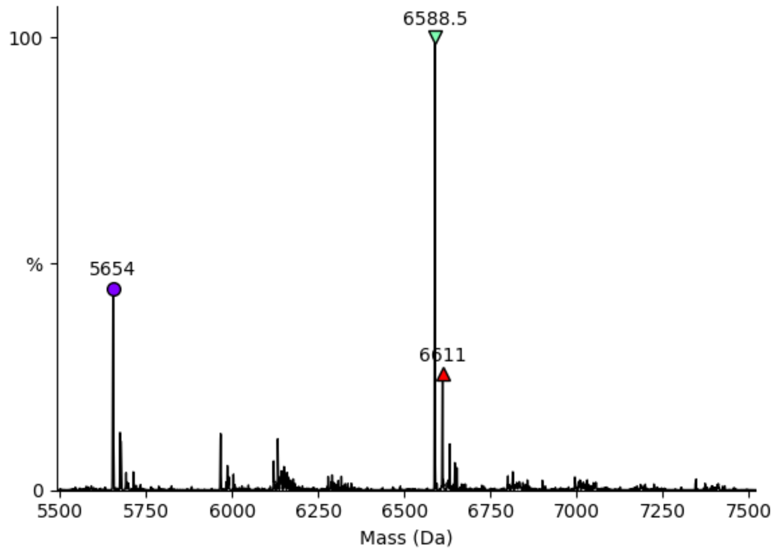

Figure S38. **19DNA_U^{put}**, deconvoluted spectrum, calculated mass: 6589.0 Da, found mass: 6588.5 (product).

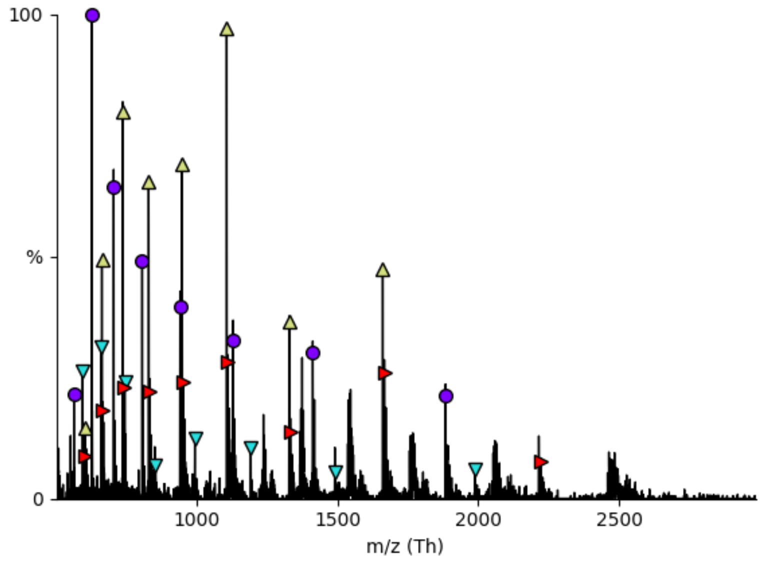

Figure S39. **19DNA_U^{glu}**, raw spectrum.

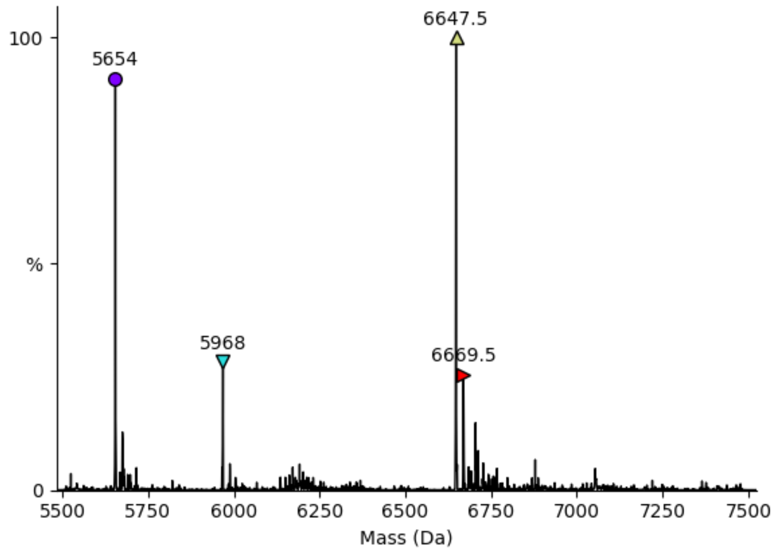

Figure S40. **19DNA_U^{glu}**, deconvoluted spectrum, calculated mass: 6647.9 Da, found mass: 6647.5 (product).

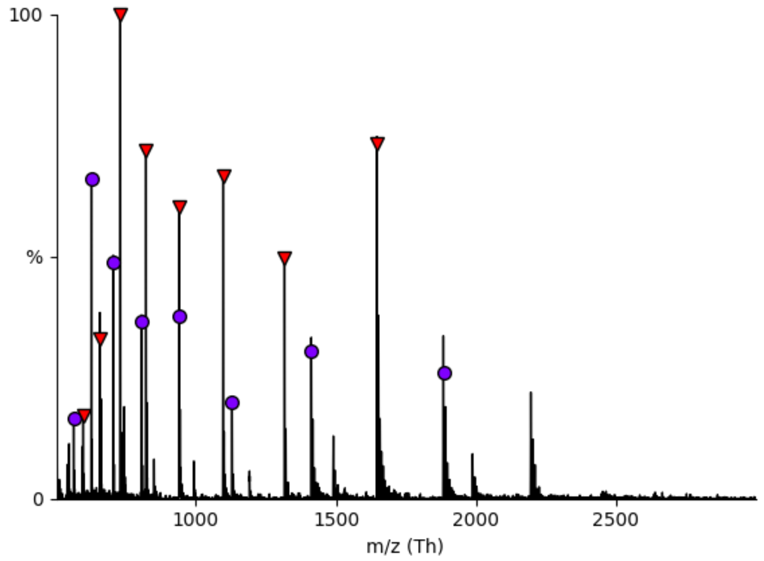

Figure S41. **19DNA_U^{dhp}**, raw spectrum.

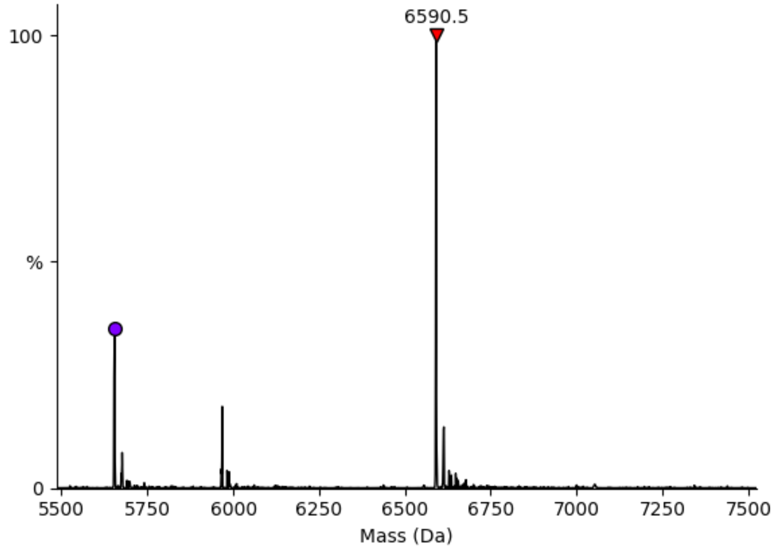

Figure S42. **19DNA_U^{dhp}**, deconvoluted spectrum, calculated mass: 6591.0 Da, found mass: 6590.5 (product).

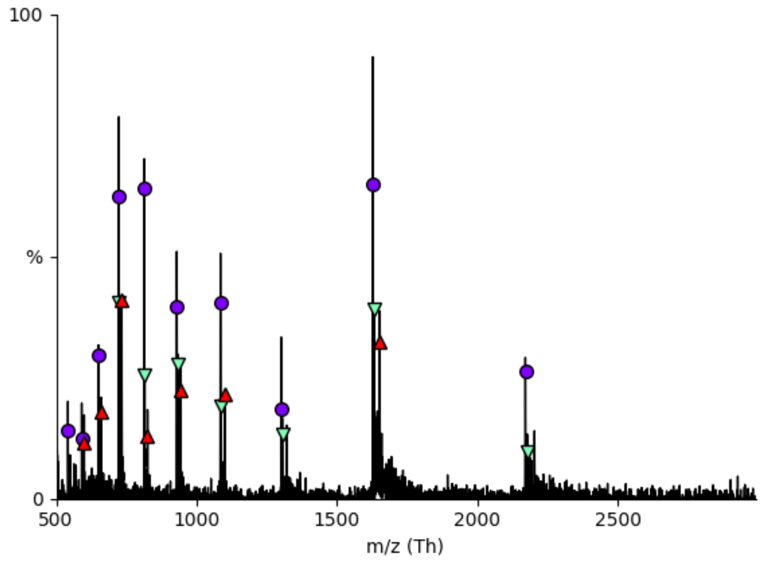

Figure S43. **19DNA_U^{sm}**, raw spectrum.

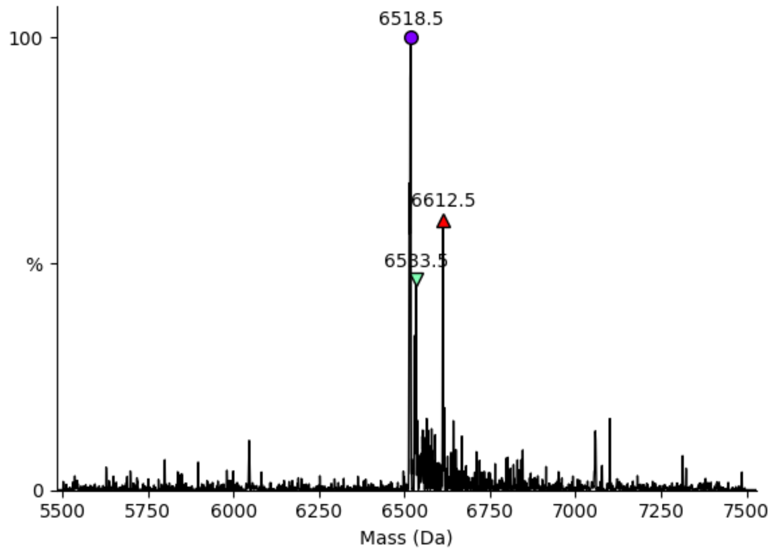

Figure S44. **19DNA_U^{sm}**, deconvoluted spectrum, calculated mass: 6534.9 Da, found mass: 6533.5 (product).

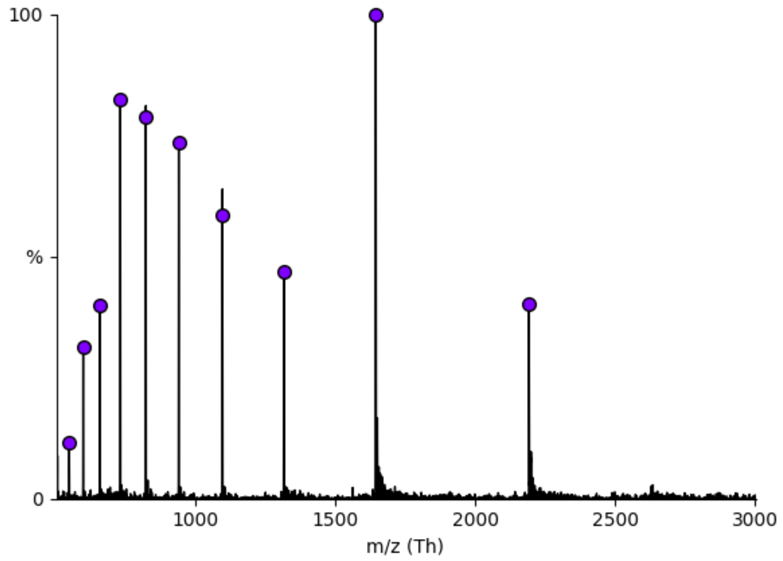

Figure S45. **19DNA_U^{asm}**, raw spectrum.

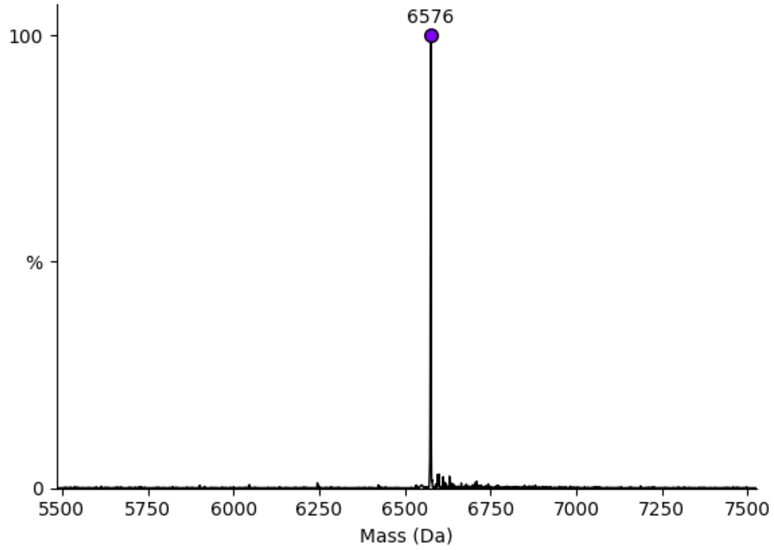

Figure S46. **19DNA_U^{asm}**, deconvoluted spectrum, calculated mass: 6576.9 Da, found mass: 6576.0 (product).

3.2. LC-MS spectra of 31DNA and 31DNA_U^X

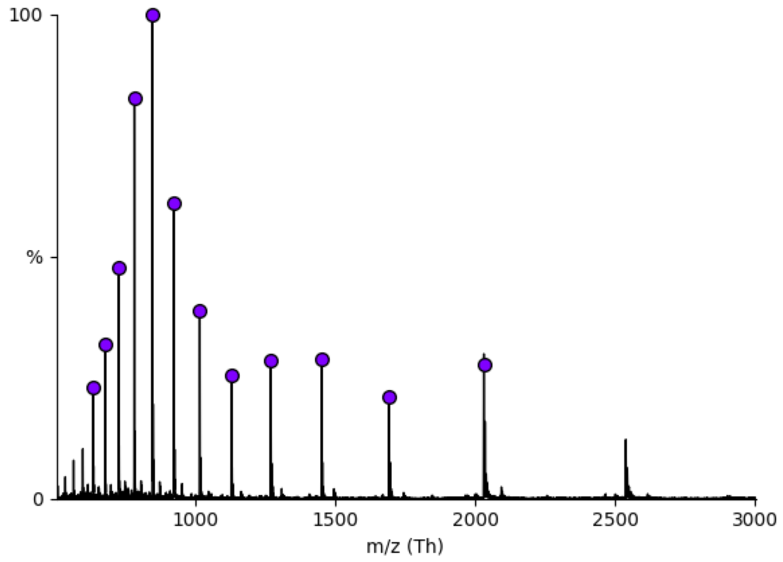

Figure S47. **31DNA**, raw spectrum.

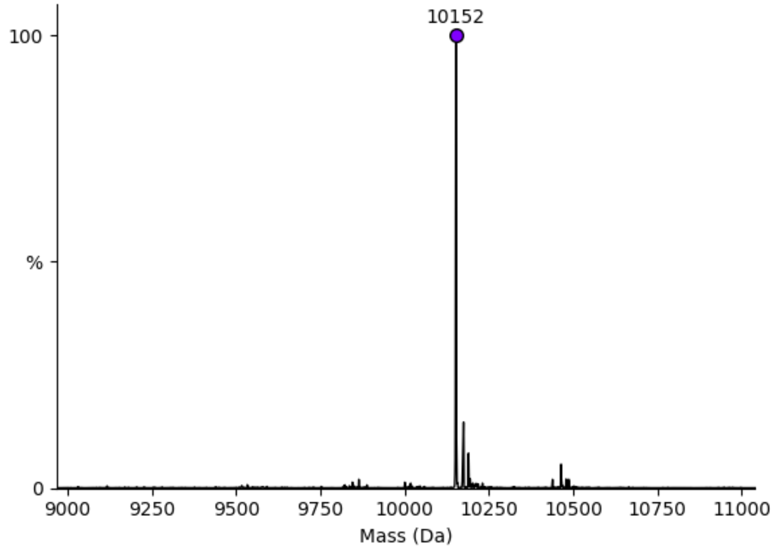

Figure S48. **31DNA**, deconvoluted spectrum, calculated mass: 10154.3 Da, found mass: 10152.0 (product).

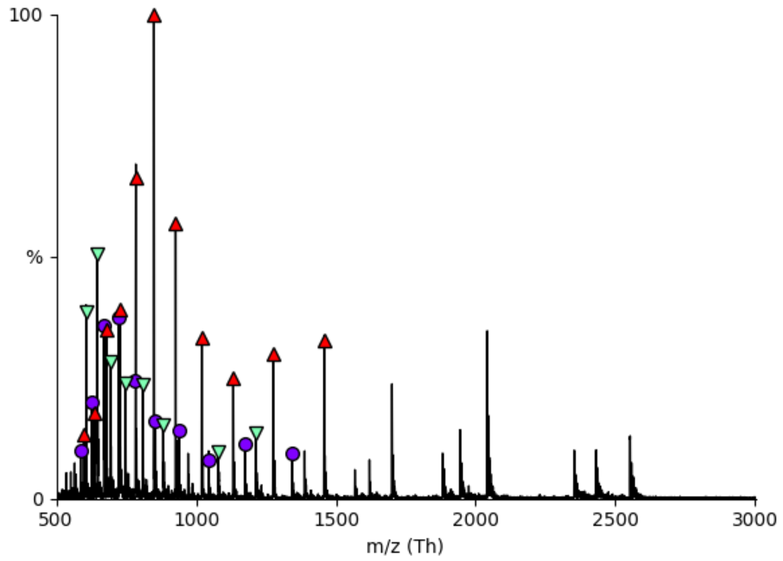

Figure S49. **31DNA_U^{am}**, raw spectrum.

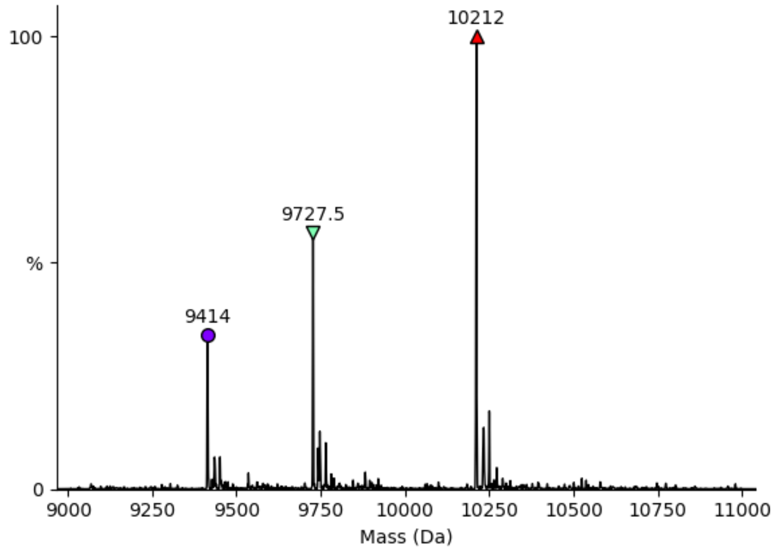

Figure S50. **31DNA_U^{am}**, deconvoluted spectrum, calculated mass: 10214.4 Da, found mass: 10212.0 (product), found mass: 9414.0 (template), found mass: 9727.5 (template + dA).

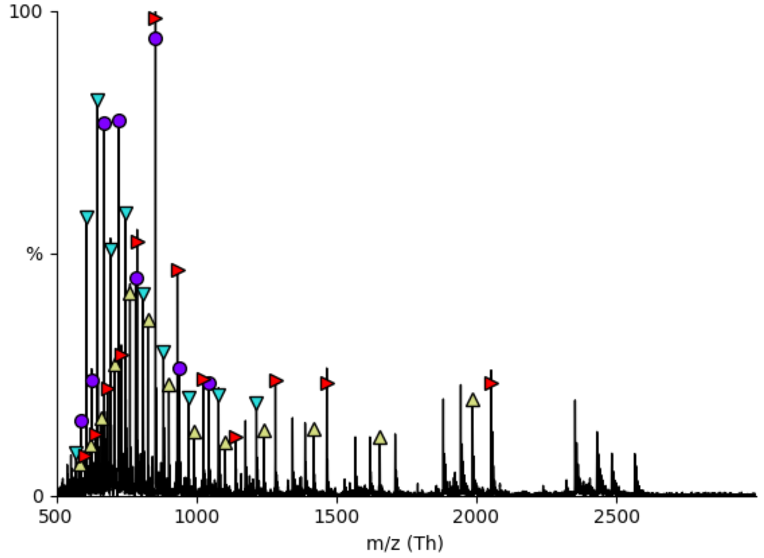

Figure S51. **31DNA_U^{mm}**, raw spectrum.

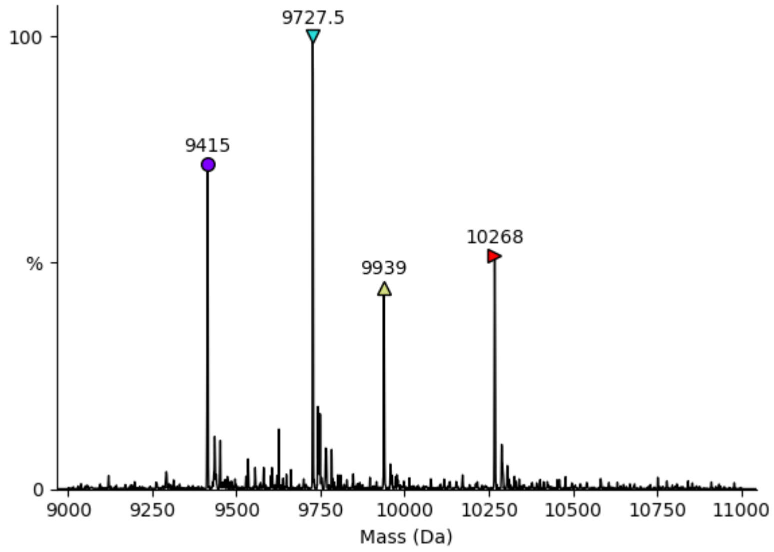

Figure S52. **31DNA_U^{mm}**, deconvoluted spectrum, calculated mass: 10270.5 Da, found mass: 10268.0 (product), found mass: 9939.0 (modified strand lacking dG), found mass: 9415.0 (template), found mass: 9727.5 (template + dA).

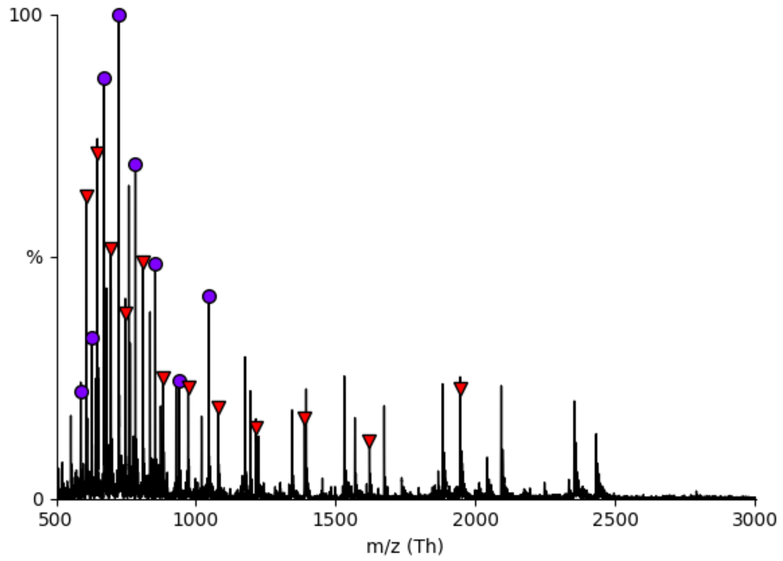

Figure S53. **31DNA_U^{dm}**, raw spectrum.

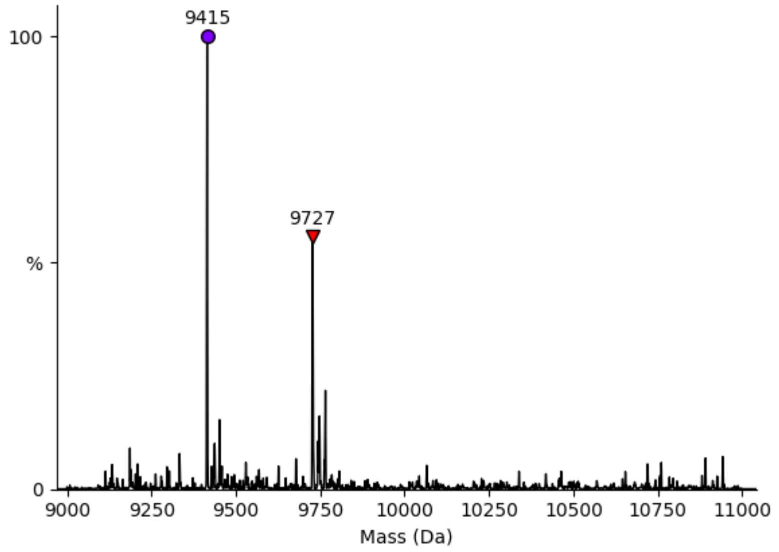

Figure S54. **31DNA_U^{dm}**, deconvoluted spectrum, calculated mass: 10326.6 Da, found mass: 9415.0 (template), found mass: 9727.0 (template + dA).

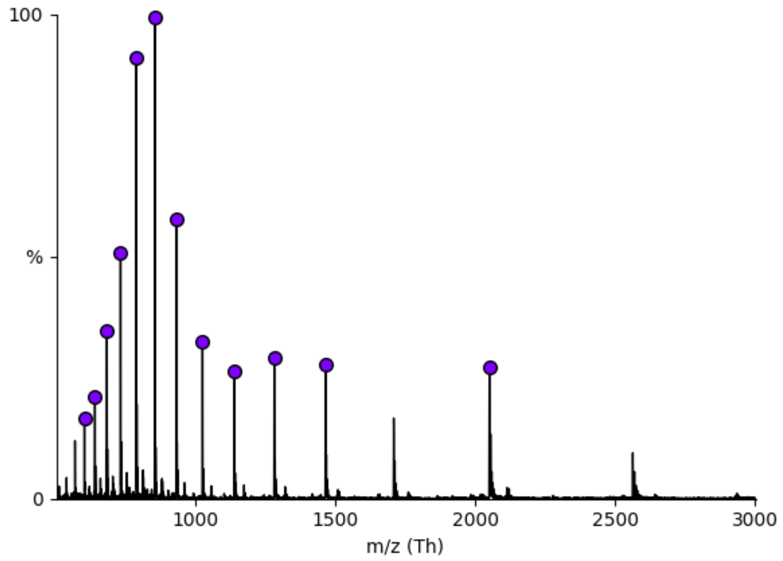

Figure S55. **31DNA_U^{cm}**, raw spectrum.

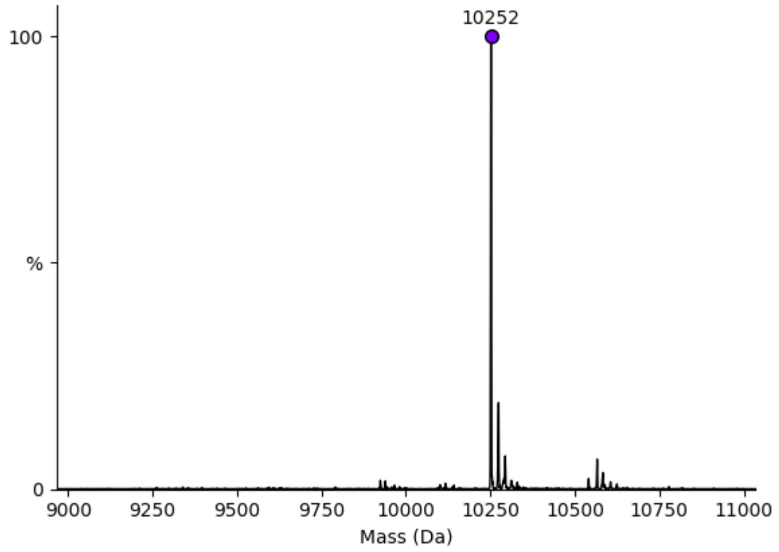

Figure S56. **31DNA_U^{cm}**, deconvoluted spectrum, calculated mass: 10254.3 Da, found mass: 10252.0 (product).

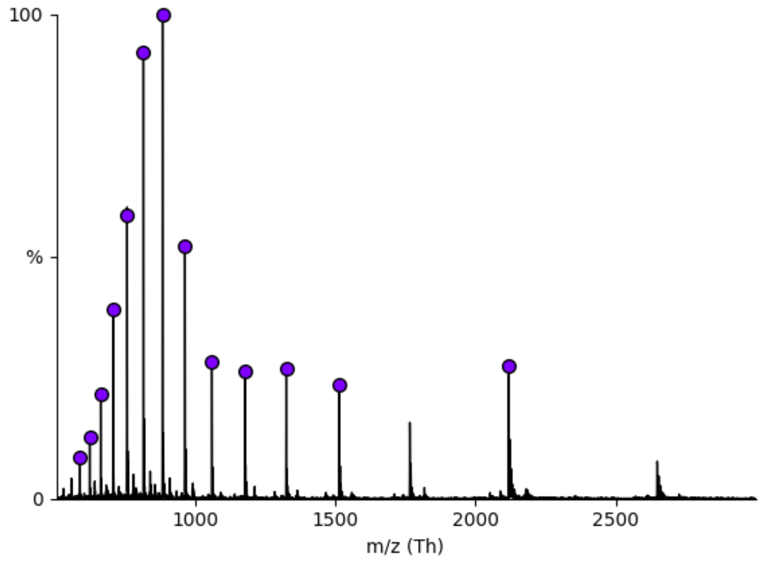

Figure S57. **31DNA_U^{tfa}**, raw spectrum.

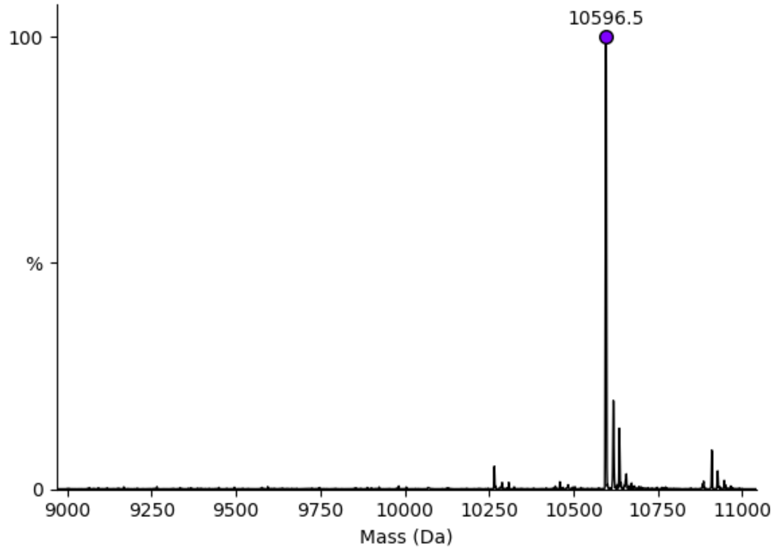

Figure S58. **31DNA_U^{tfa}**, deconvoluted spectrum, calculated mass: 10598.4 Da, found mass: 10596.5 (product).

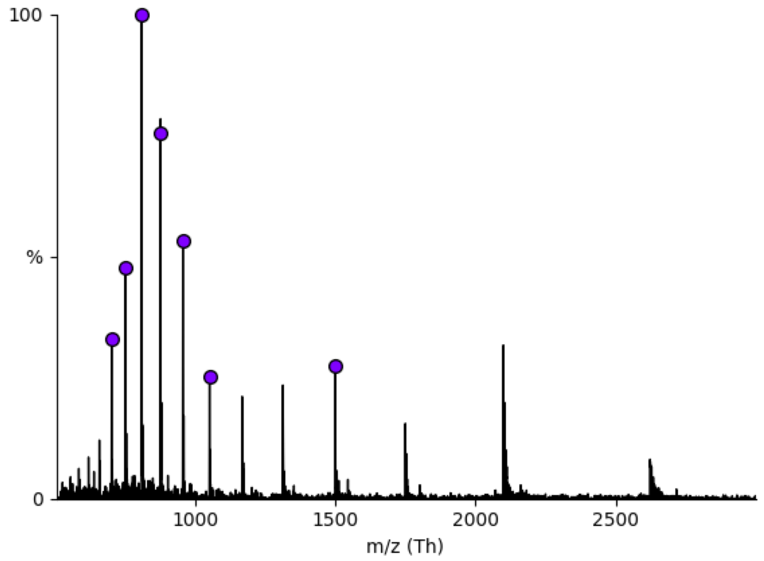

Figure S59. **31DNA_U^{tfa}**, second peak, raw spectrum.

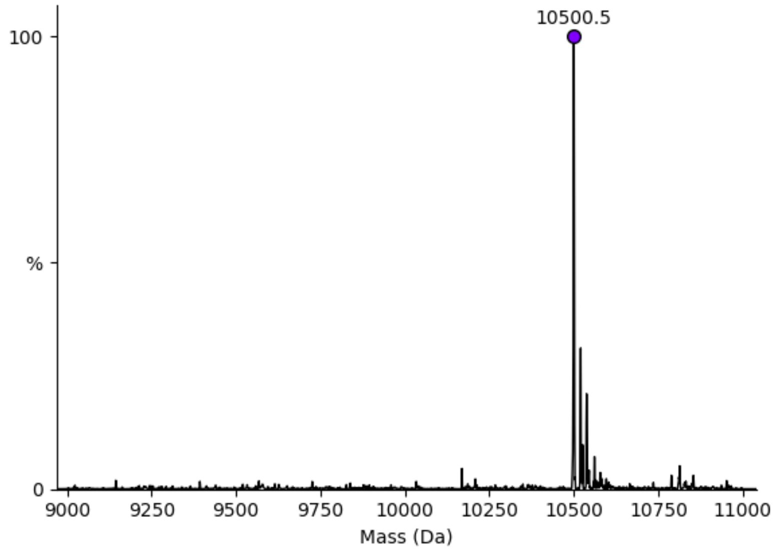

Figure S60. **31DNA_U^{tfa}**, second peak, deconvoluted spectrum, calculated mass: 10598.4 Da, found mass: 10500.5 (product lacking one TFA group).

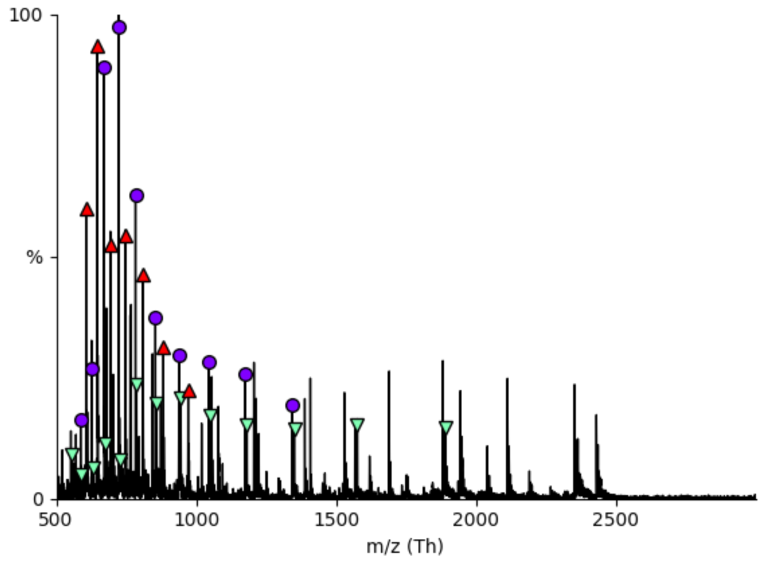

Figure S61. **31DNA_U^{put}**, raw spectrum.

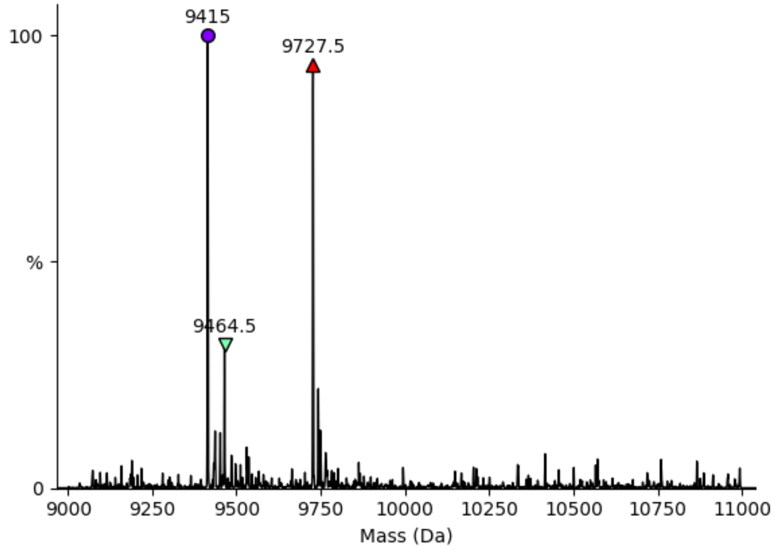

Figure S62. **31DNA_U^{put}**, deconvoluted spectrum, calculated mass: 10498.9 Da, found mass: 9415.0 (template), found mass: 9727.5 (template + dA).

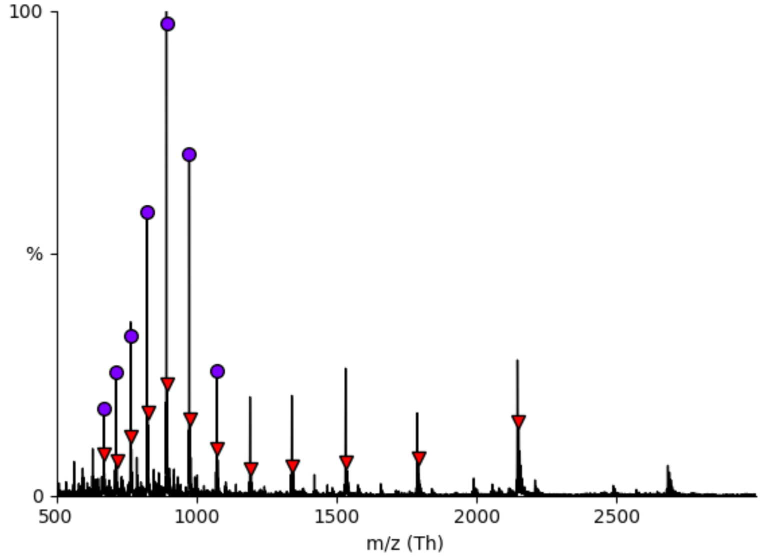

Figure S63. **31DNA_U^{glu}**, raw spectrum.

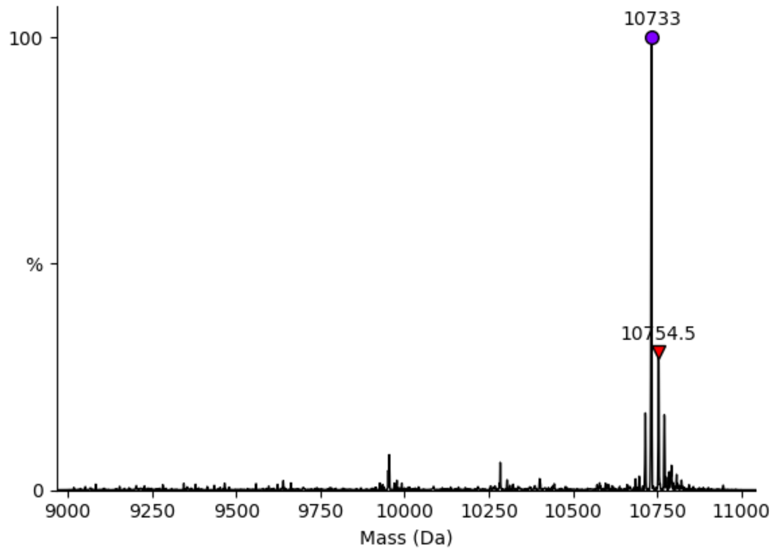

Figure S64. **31DNA_U^{glu}**, deconvoluted spectrum, calculated mass: 10734.8 Da, found mass: 10733.0 (product).

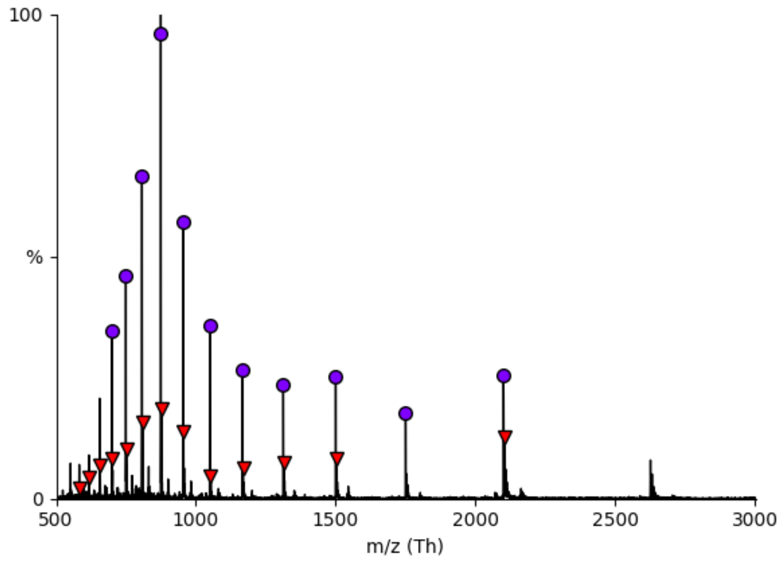

Figure S65. **31DNA_U^dhp^**, raw spectrum.

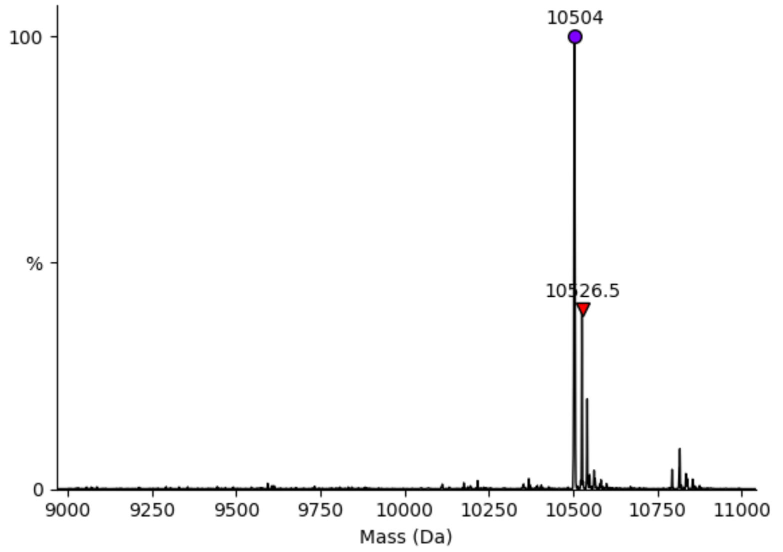

Figure S66. **31DNA_U^dhp^**, deconvoluted spectrum, calculated mass: 10506.7 Da, found mass: 10504.0 (product).

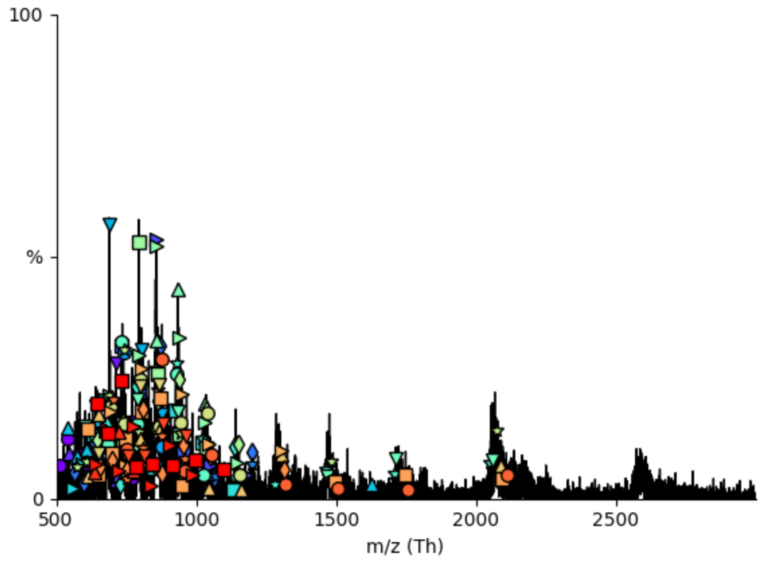

Figure S67. **31DNA_U^{sm}**, raw spectrum.

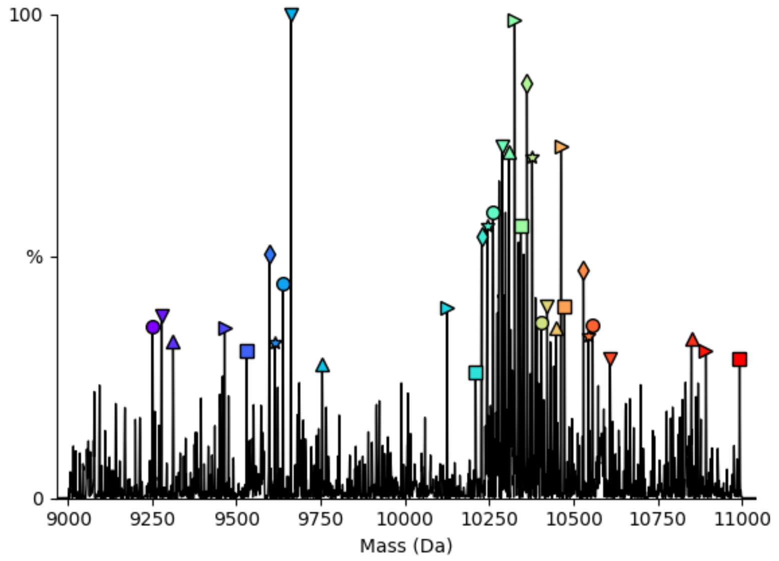

Figure S68. **31DNA_U^{sm}**, deconvoluted spectrum, calculated mass: 10282.5 Da, found mass: none detected.

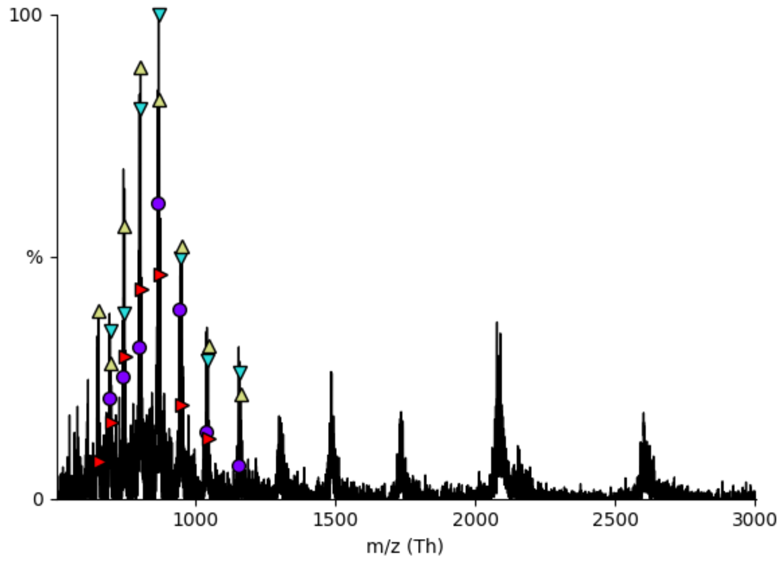

Figure S69. **31DNA_U^{asm}**, raw spectrum.

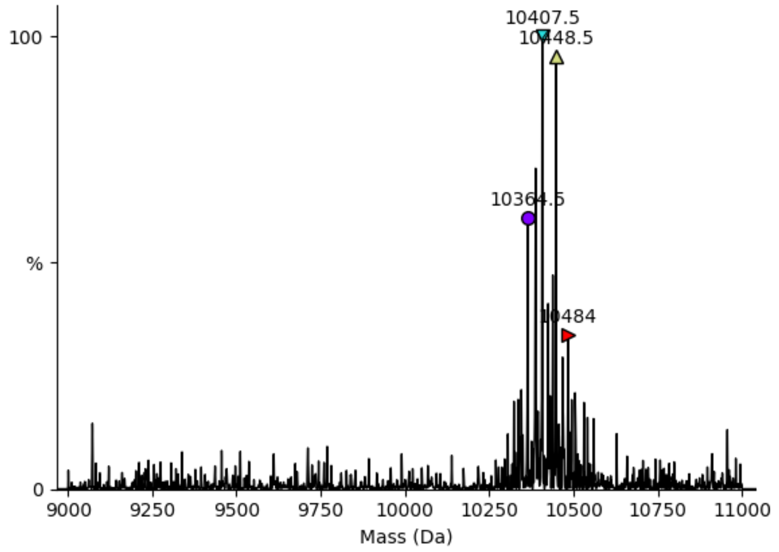

Figure S70. **31DNA_U^{asm}**, deconvoluted spectrum, calculated mass: 10450.7 Da, found mass: 10448.5 (product), found mass: 10407.5 (product lacking Ac protecting group).

3.3. LC-MS spectra of 37DNA/37DNA_U^X^/37DNA_C^X^

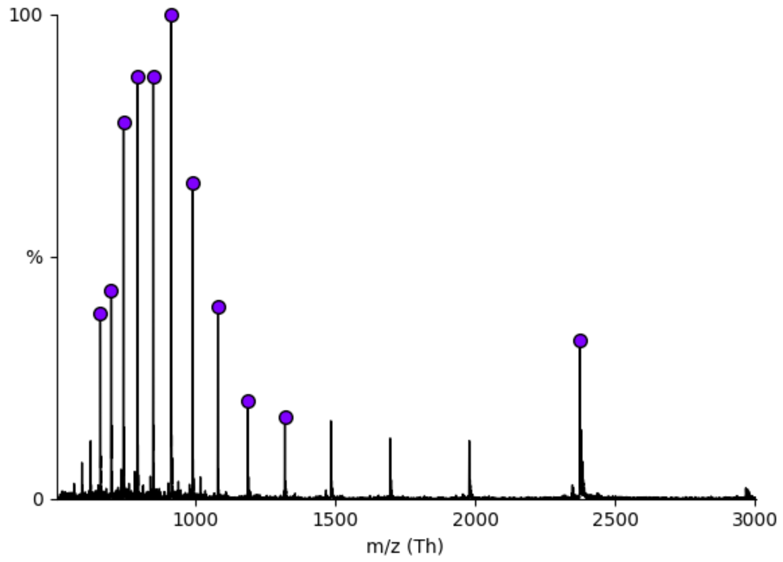

Figure S71. **37DNA**, raw spectrum.

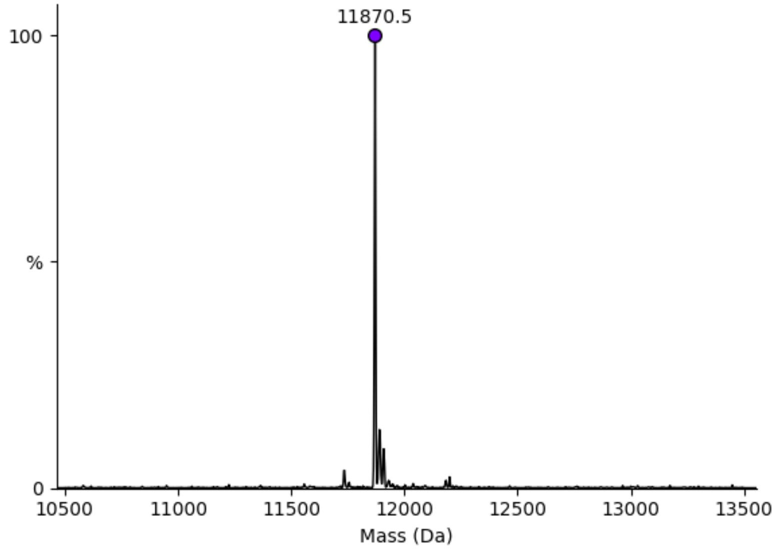

Figure S72. **37DNA**, deconvoluted spectrum, calculated mass: 11871.5 Da, found mass: 11870.5 (product).

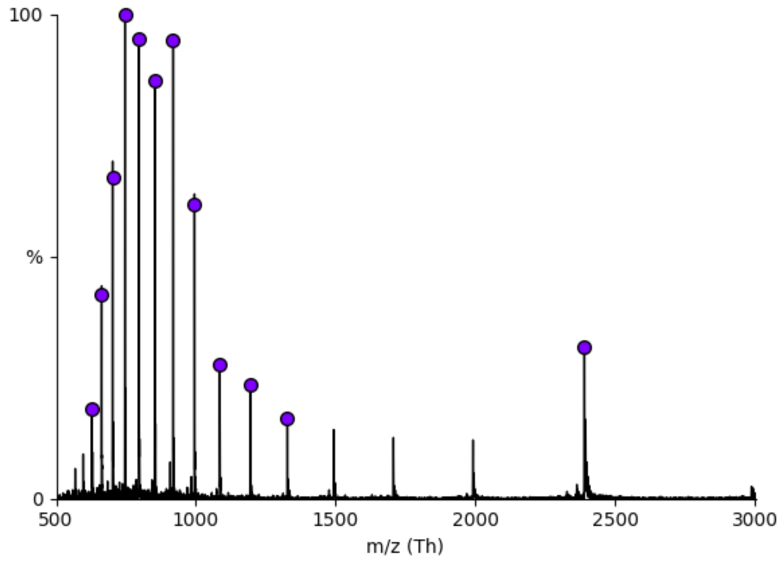

Figure S73. **37DNA_U^{hm}**, raw spectrum.

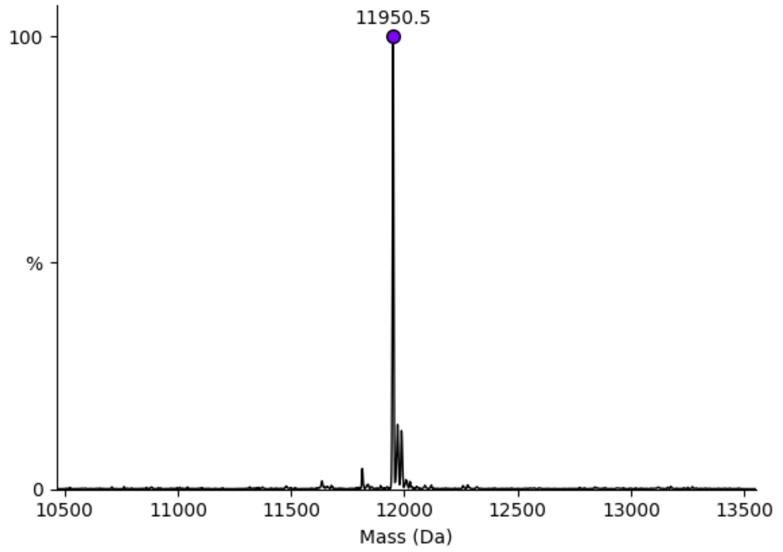

Figure S74. **37DNA_U^{hm}**, deconvoluted spectrum, calculated mass: 11951.5 Da, found mass: 11950.5 (product).

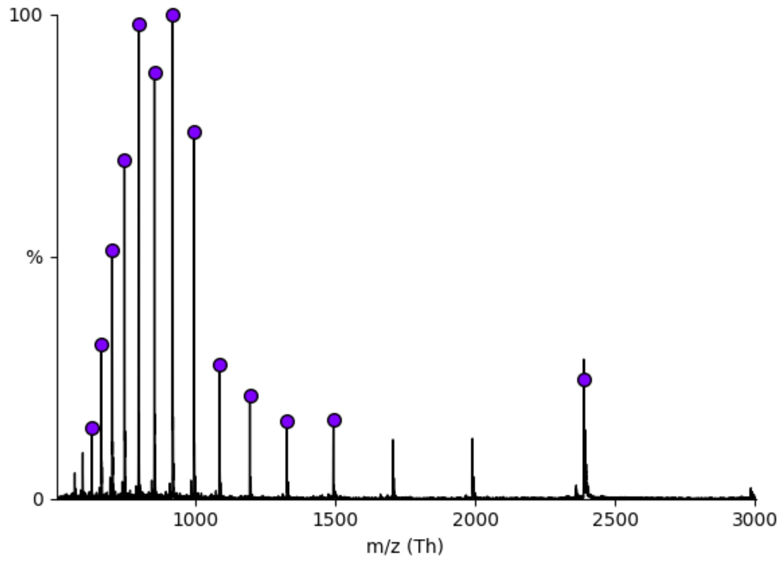

Figure S75. **37DNA_U^{et}**, raw spectrum.

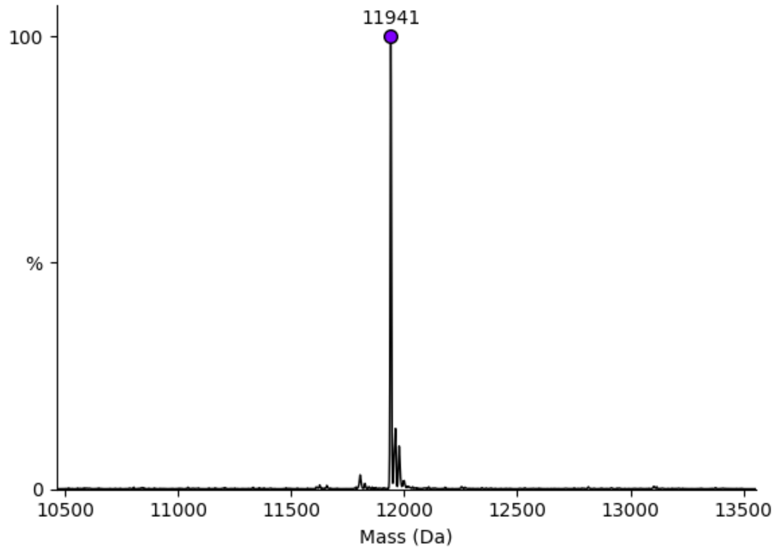

Figure S76. **37DNA_U^{et}**, deconvoluted spectrum, calculated mass: 11941.7 Da, found mass: 11941.0 (product).

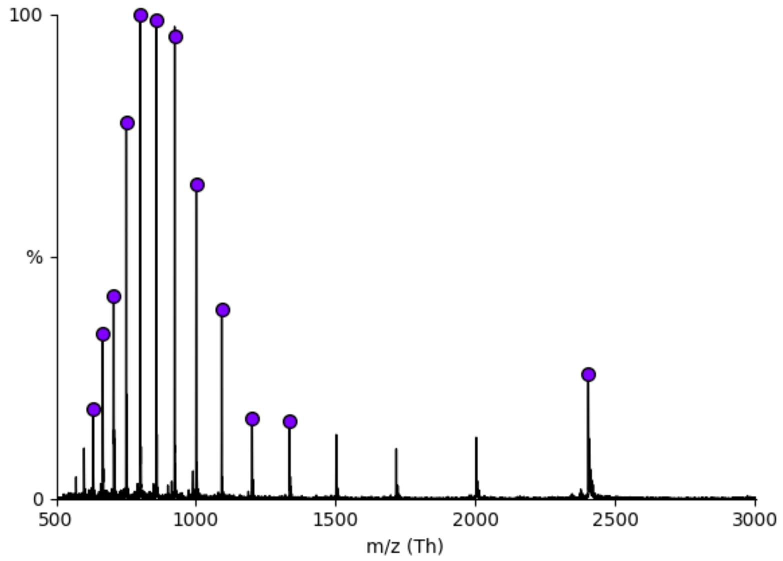

Figure S77. **37DNA_U^{She}**, raw spectrum.

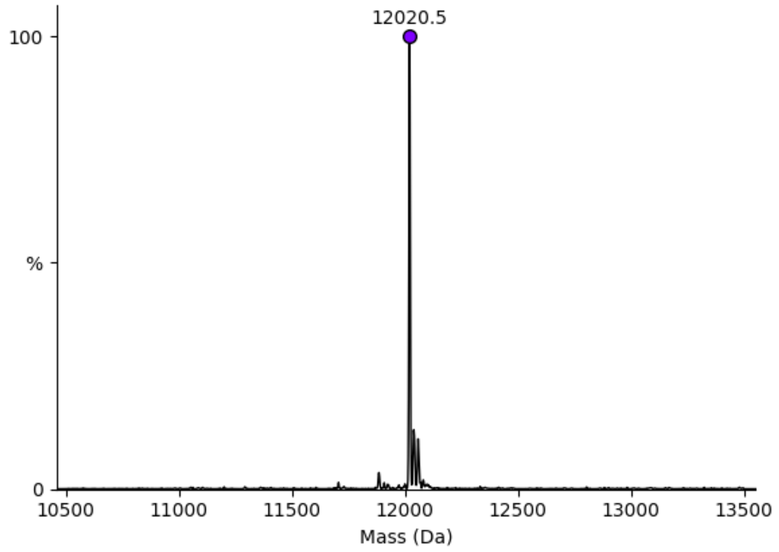

Figure S78. **37DNA_U^{She}**, deconvoluted spectrum, calculated mass: 12021.7 Da, found mass: 12020.5 (product).

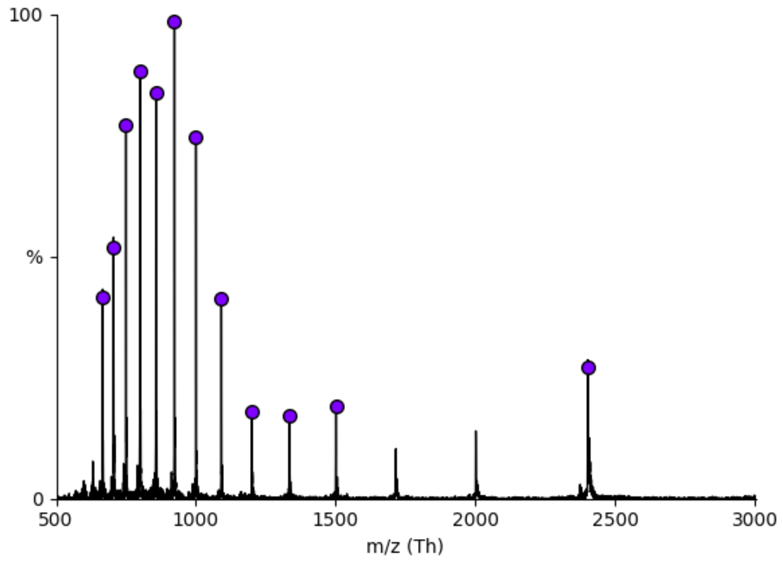

Figure S79. **37DNA_U^{ac}**, raw spectrum.

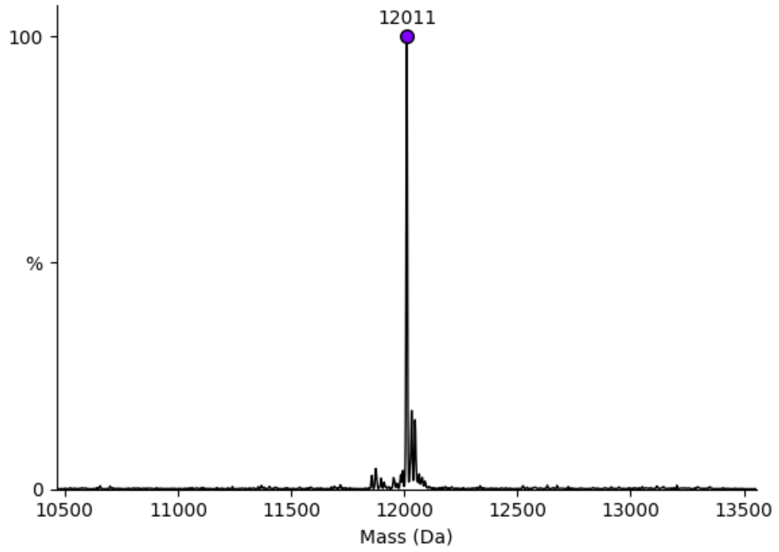

Figure S80. **37DNA_U^{ac}**, deconvoluted spectrum, calculated mass: 12011.6 Da, found mass: 12011.0 (product).

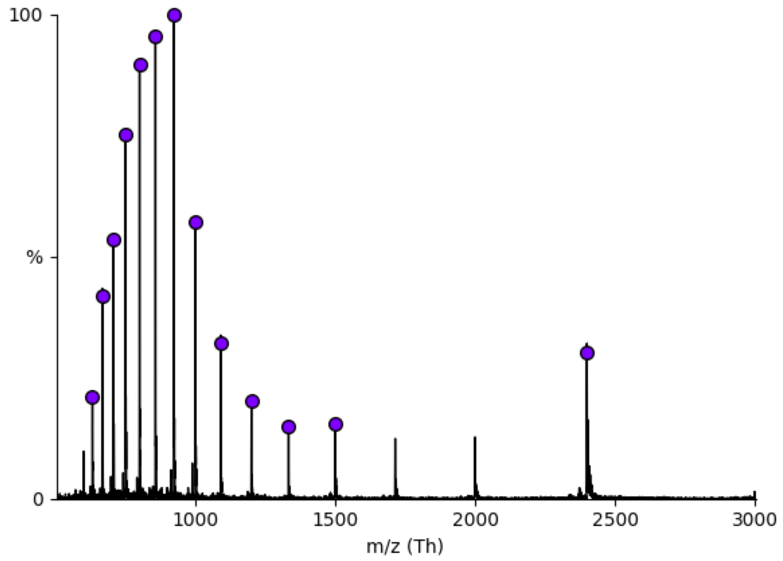

Figure S81. **37DNA_U^{cm}**, raw spectrum.

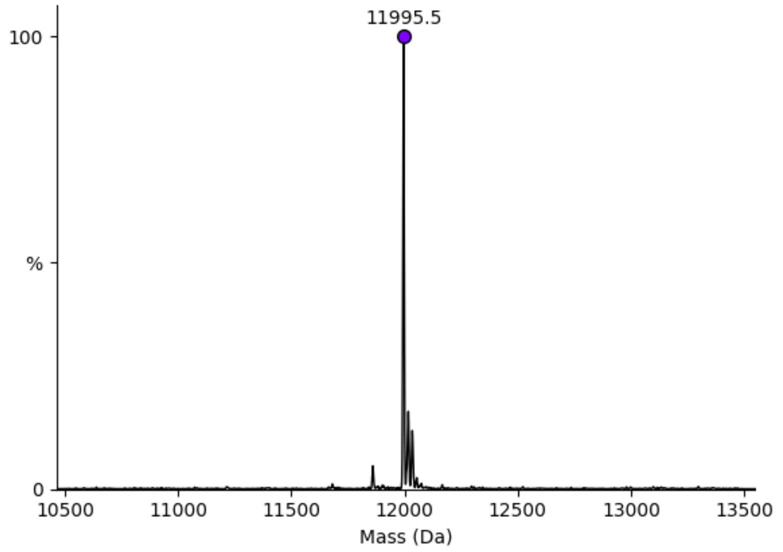

Figure S82. **37DNA_U^{cm}**, deconvoluted spectrum, calculated mass: 11996.6 Da, found mass: 11995.5 (product).

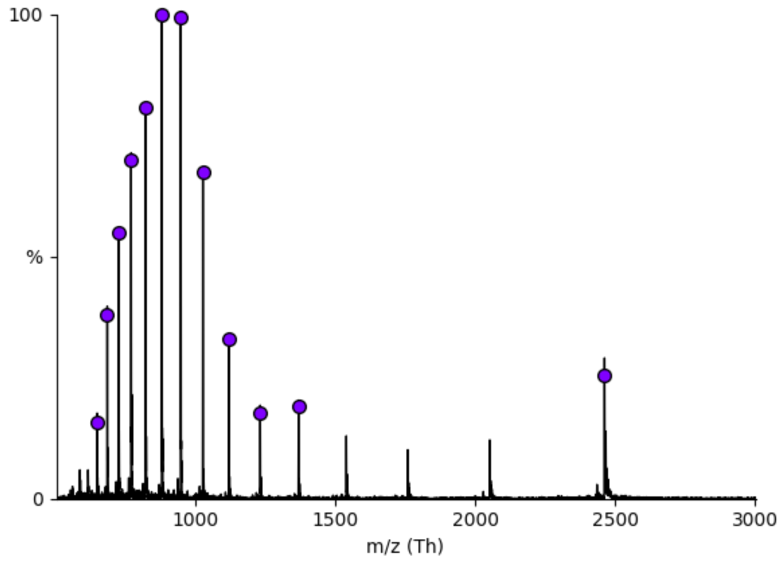

Figure S83. **37DNA_U^{dhp}**, raw spectrum.

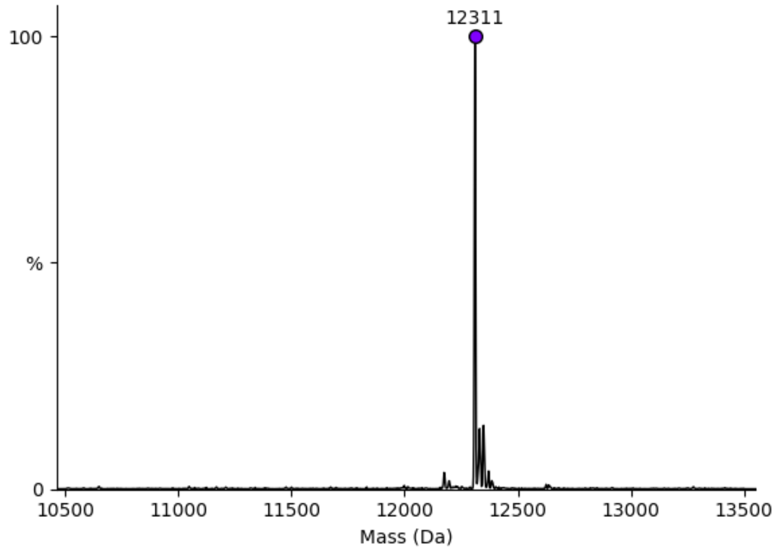

Figure S84. **37DNA_U^{dhp}**, deconvoluted spectrum, calculated mass: 12312.1 Da, found mass: 12311.0 (product).

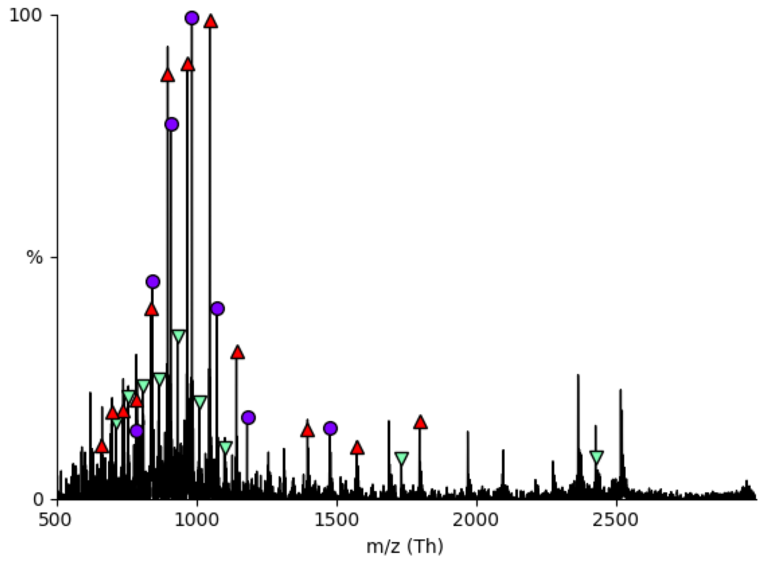

Figure S85. **37DNA_U^{glu}**, raw spectrum.

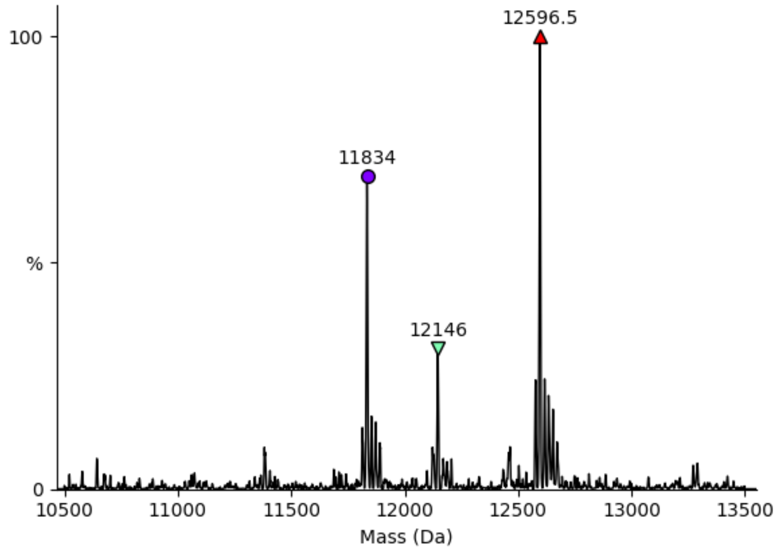

Figure S86. **37DNA_U^{glu}**, deconvoluted spectrum, calculated mass: 12597.1 Da, found mass: 12596.5 (product), found mass: 12146.0 (modified strand lacking **dU^{glu}**), found mass: 11834.0 (modified strand lacking **dU^{glu}** and dA).

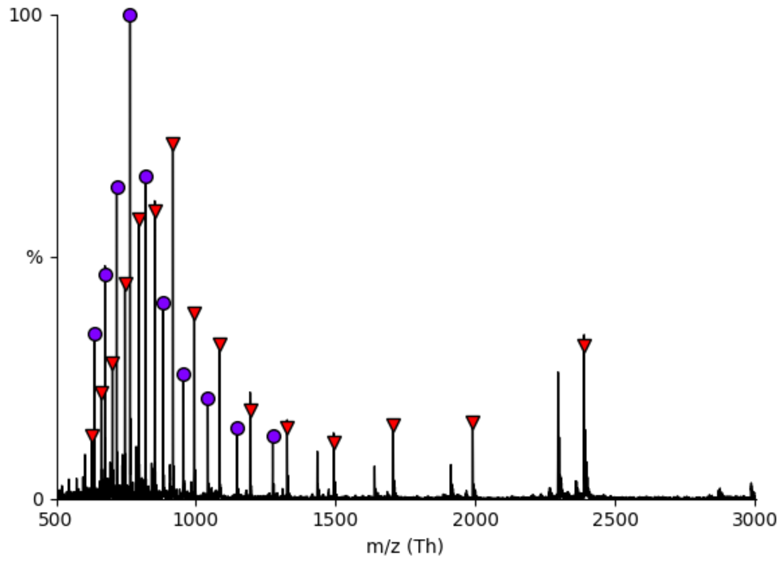

Figure S87. **37DNA_U^{am}**, raw spectrum.

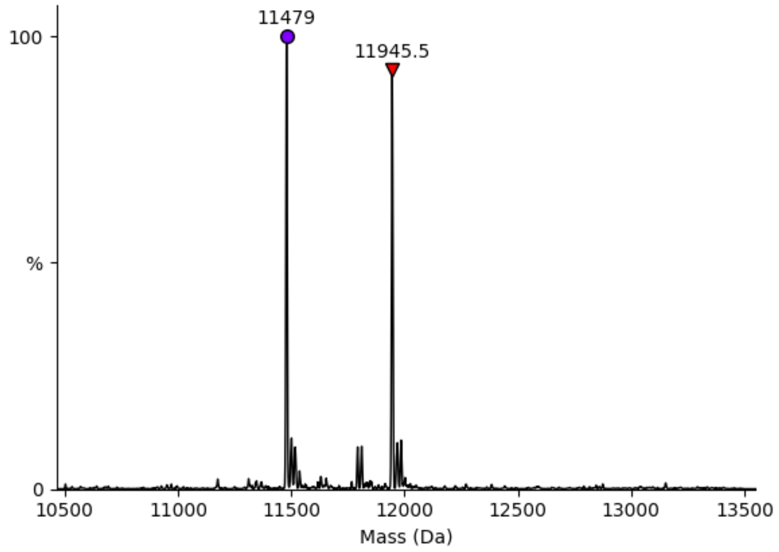

Figure S88. **37DNA_U^{am}**, deconvoluted spectrum, calculated mass: 11946.6 Da, found mass: 11945.5 (product), found mass: 11479.0 (template).

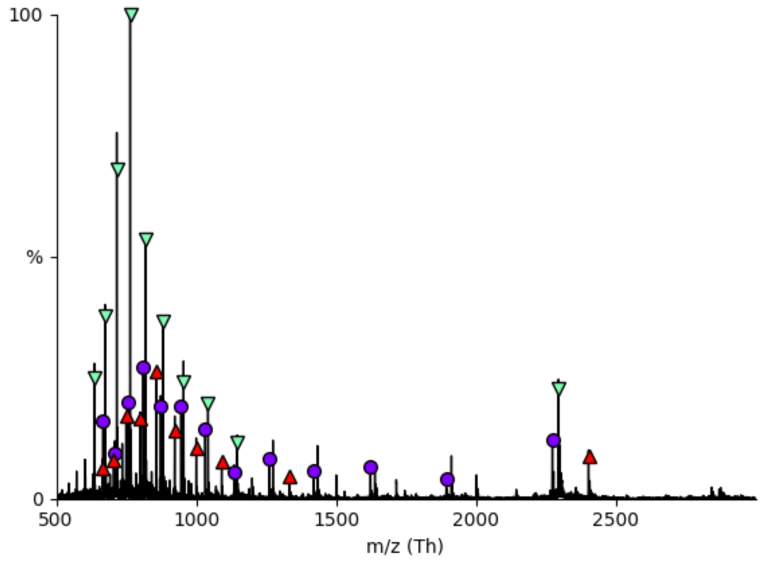

Figure S89. **37DNA_U^{mm}**, raw spectrum.

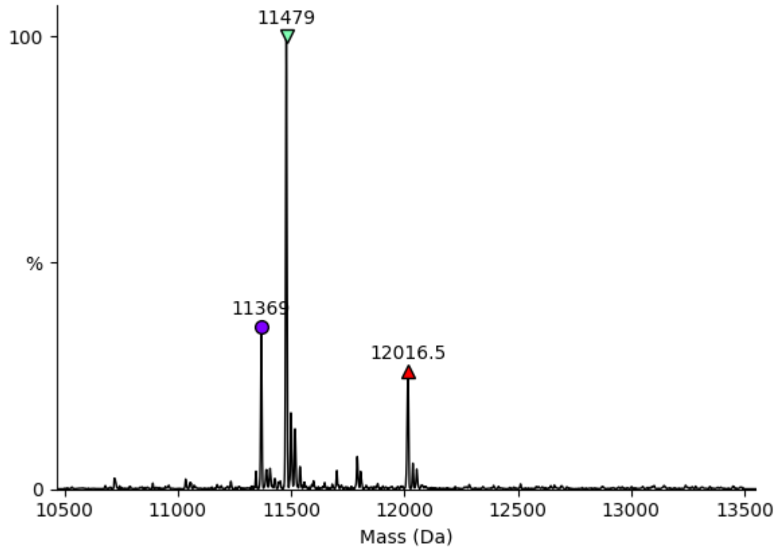

Figure S90. **37DNA_U^{mm}**, deconvoluted spectrum, calculated mass: 12016.7 Da, found mass: 12016.5 (product), found mass: 11479.0 (template), found mass: 11369.0 (modified strand lacking **dU^{mm}** and dA).

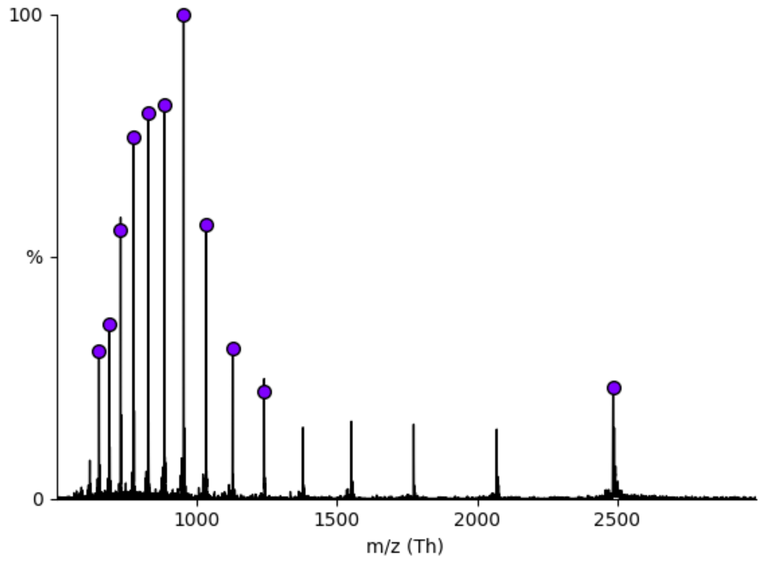

Figure S91. **37DNA_U^{tfa}**, raw spectrum.

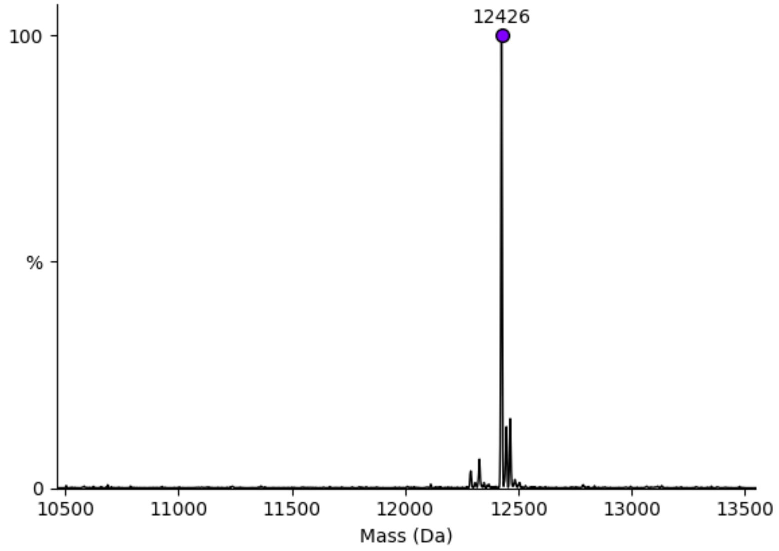

Figure S92. **37DNA_U^{tfa}**, deconvoluted spectrum, calculated mass: 12426.6 Da, found mass: 12426.0 (product).

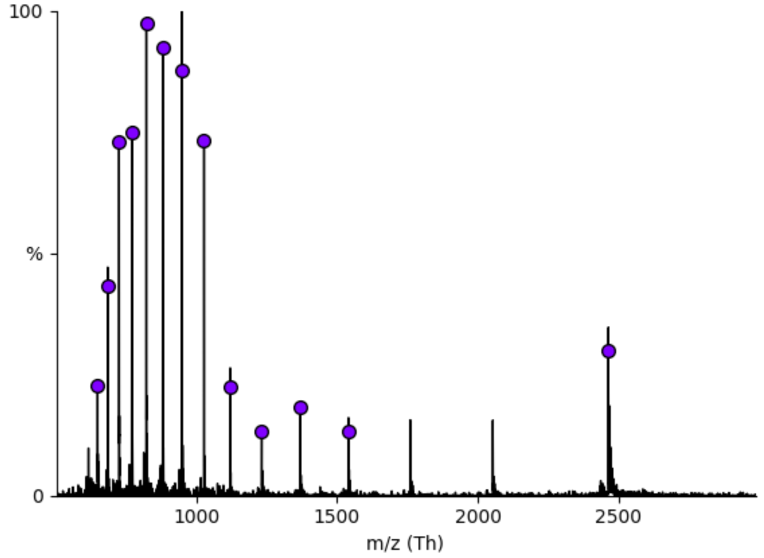

Figure S93. **37DNA_U^{tfa}**, second peak, raw spectrum.

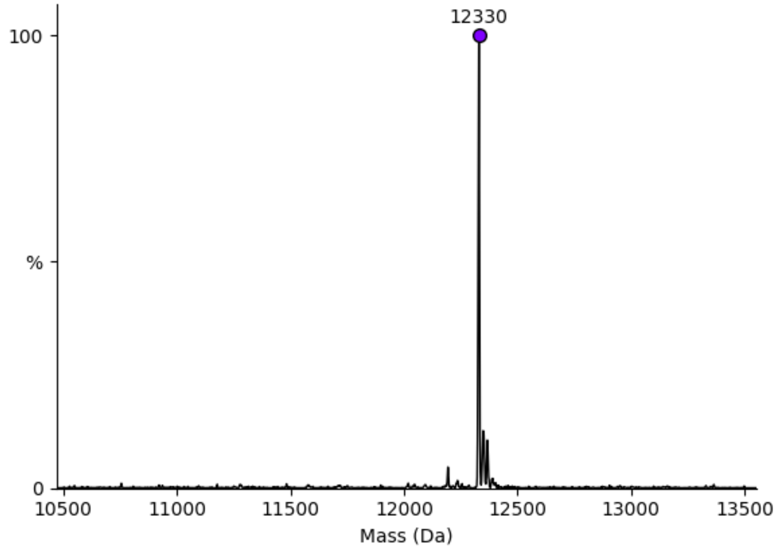

Figure S94. **37DNA_U^{tfa}**, second peak, deconvoluted spectrum, calculated mass: 12426.6 Da, found mass: 12330.0 (product lacking TFA protecting group).

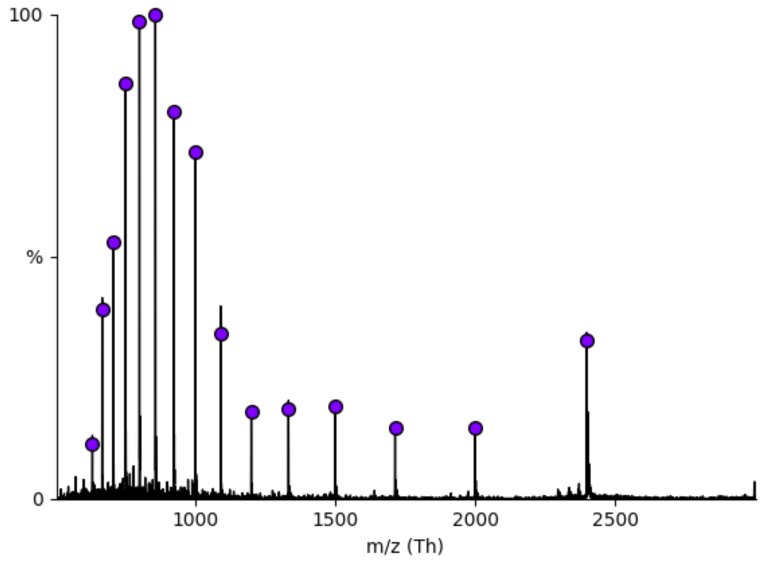

Figure S95. **37DNA_C^{hm}**, raw spectrum.

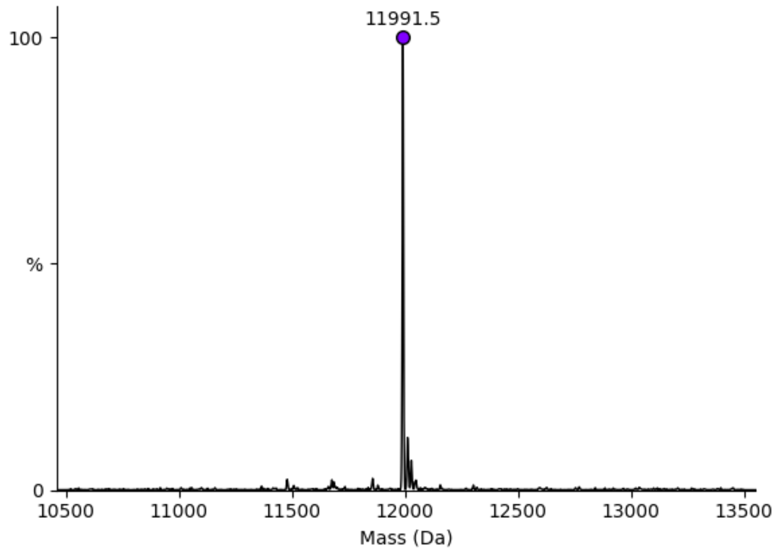

Figure S96. **37DNA_C^{hm}**, deconvoluted spectrum, calculated mass: 11991.6 Da, found mass: 11991.5 (product).

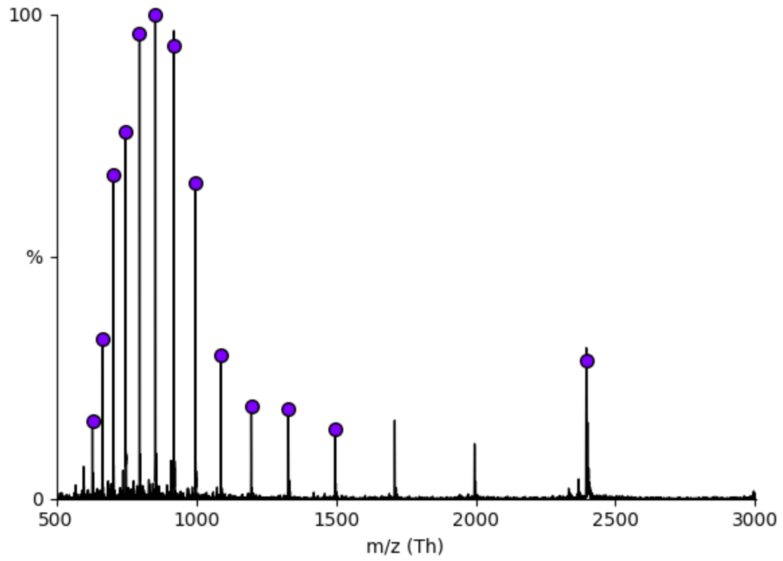

Figure S97. **37DNA_C^{et}**, raw spectrum.

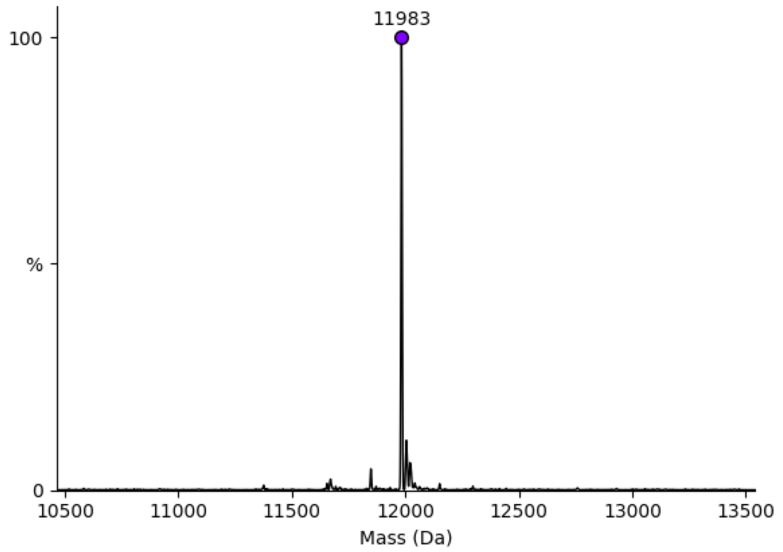

Figure S98. **37DNA_C^{et}**, deconvoluted spectrum, calculated mass: 11983.7 Da, found mass: 11983.0 (product).

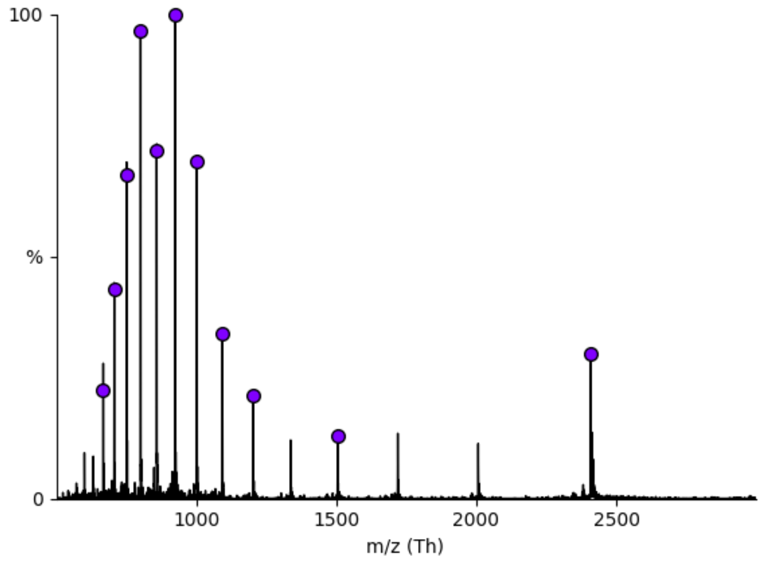

Figure S99. **37DNA_C$^{\mathbf{She}}$**, raw spectrum.

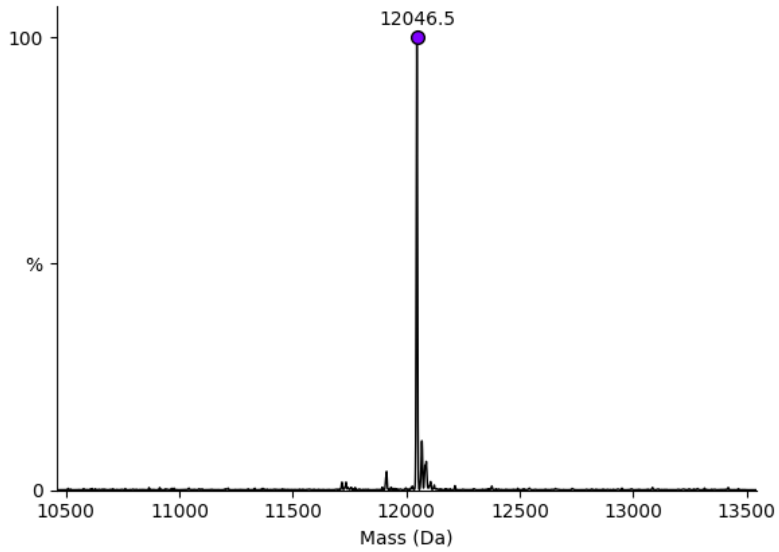

Figure S100. **37DNA_C$^{\mathbf{She}}$**, deconvoluted spectrum, calculated mass: 12047.7 Da, found mass: 12046.5 (product).

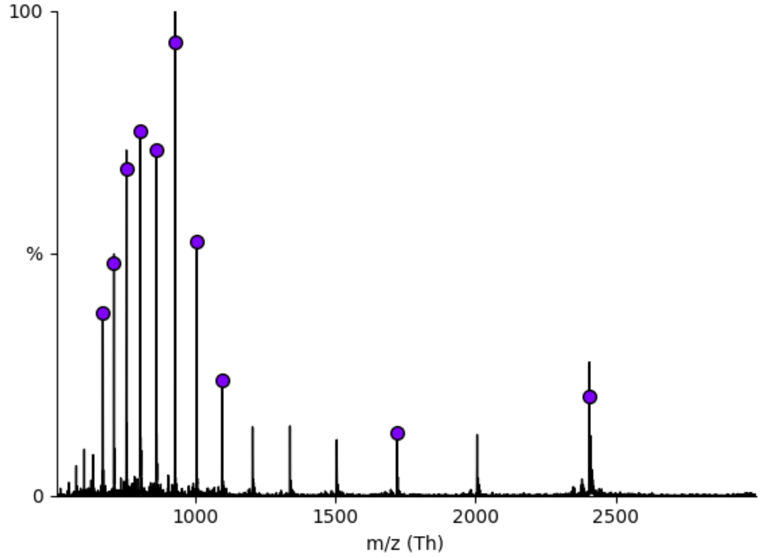

Figure S101. **37DNA_C^{ac}**, raw spectrum.

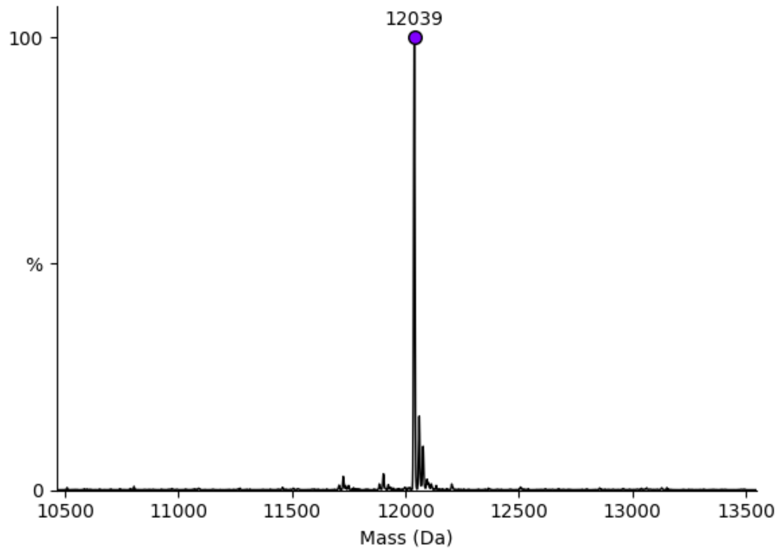

Figure S102. **37DNA_C^{ac}**, deconvoluted spectrum, calculated mass: 12039.6 Da, found mass: 12039.0 (product).

3.4. LC-MS spectra of 87DNA/87DNA_U^{X}/87DNA_C^{X}

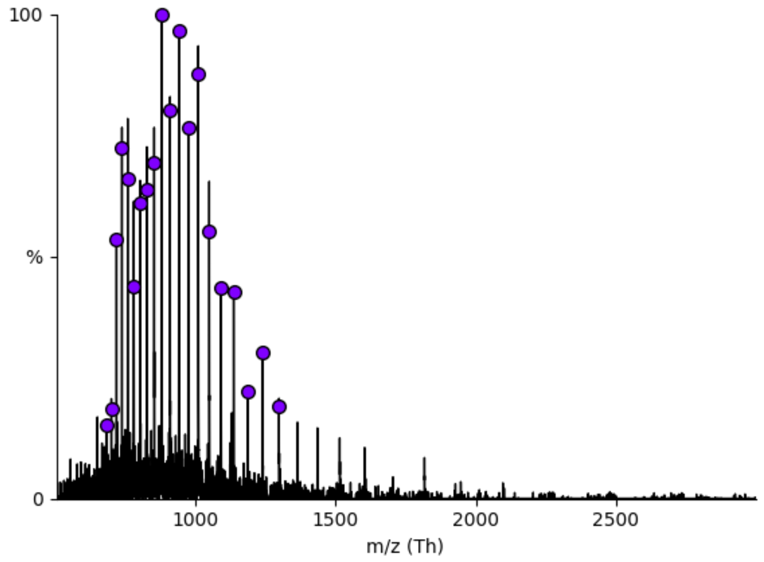

Figure S103. **87DNA**, raw spectrum.

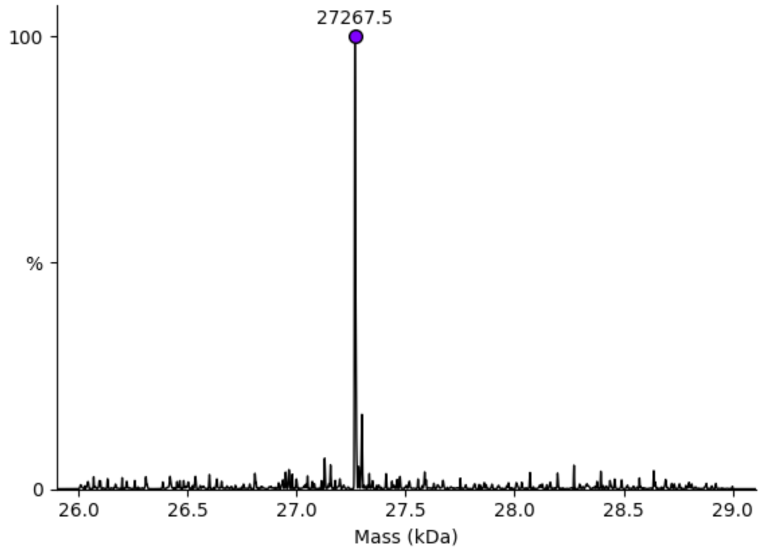

Figure S104. **87DNA**, deconvoluted spectrum, calculated mass: 27276.6 Da, found mass: 27267.5 (product).

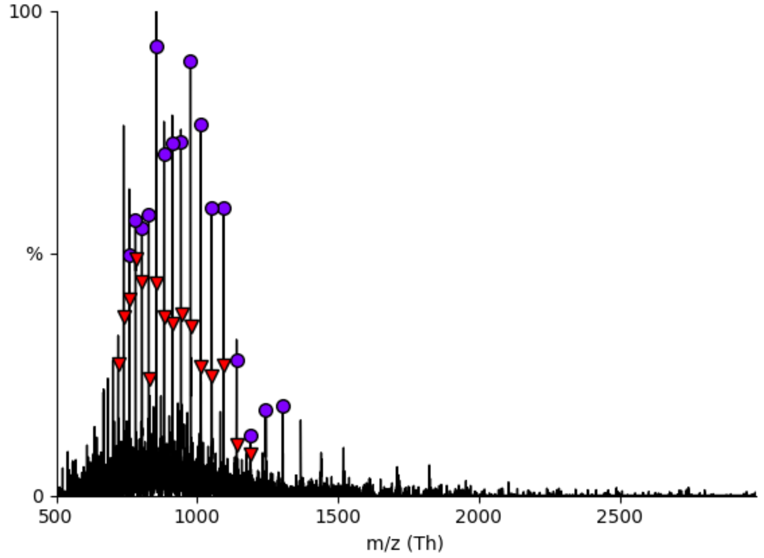

Figure S105. **87DNA_U^{hm}**, raw spectrum.

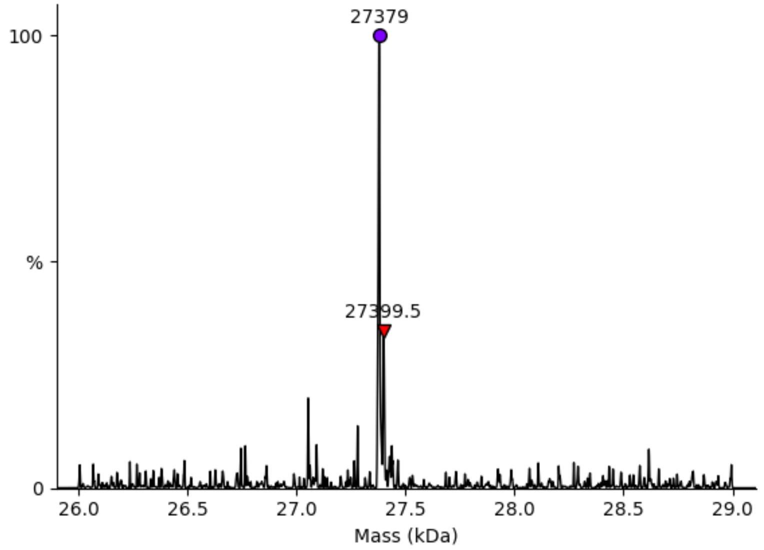

Figure S106. **87DNA_U^{hm}**, deconvoluted spectrum, calculated mass: 27388.6 Da, found mass: 27379.0 (product).

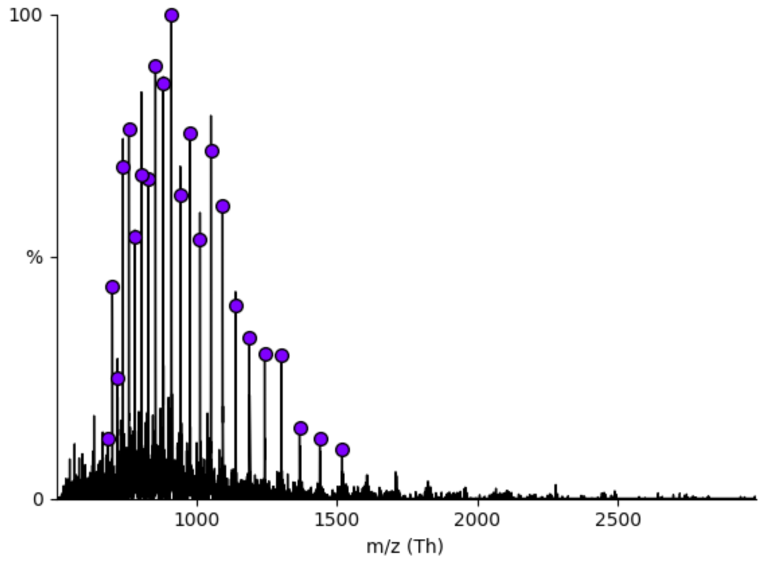

Figure S107. **87DNA_U^{et}**, raw spectrum.

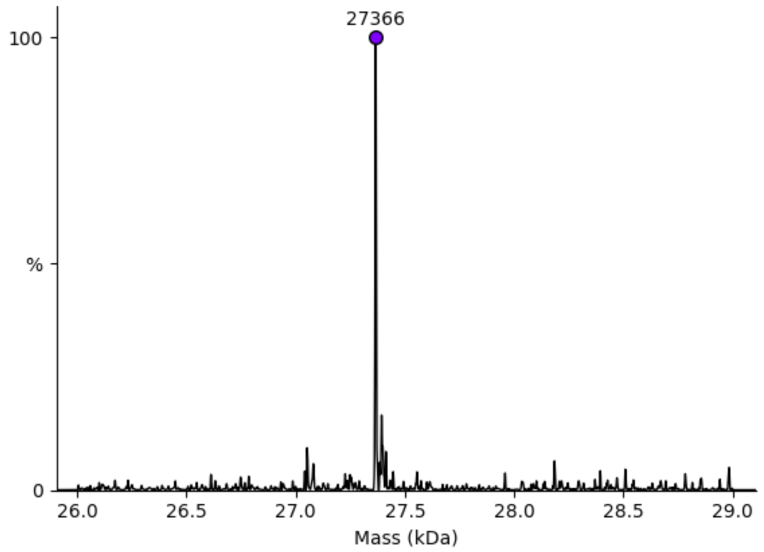

Figure S108. **87DNA_U^{et}**, deconvoluted spectrum, calculated mass: 27374.8 Da, found mass: 27366.0 (product).

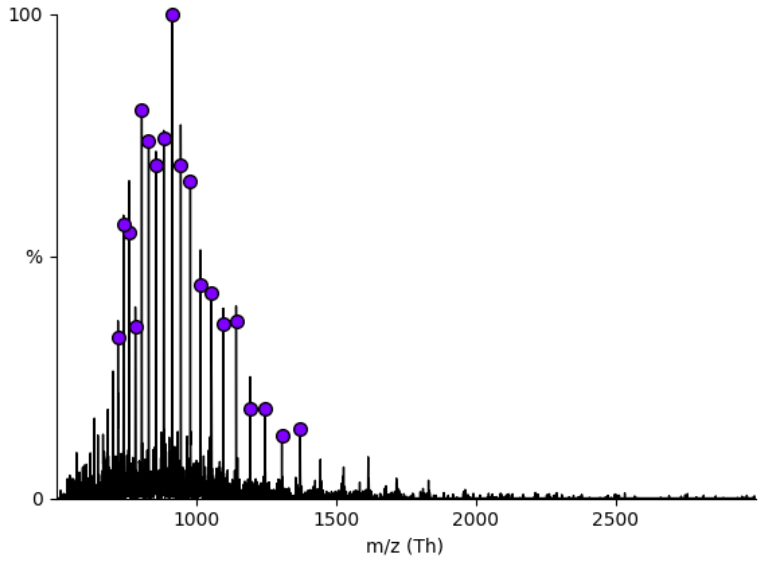

Figure S109. **87DNA_U^{She}**, raw spectrum.

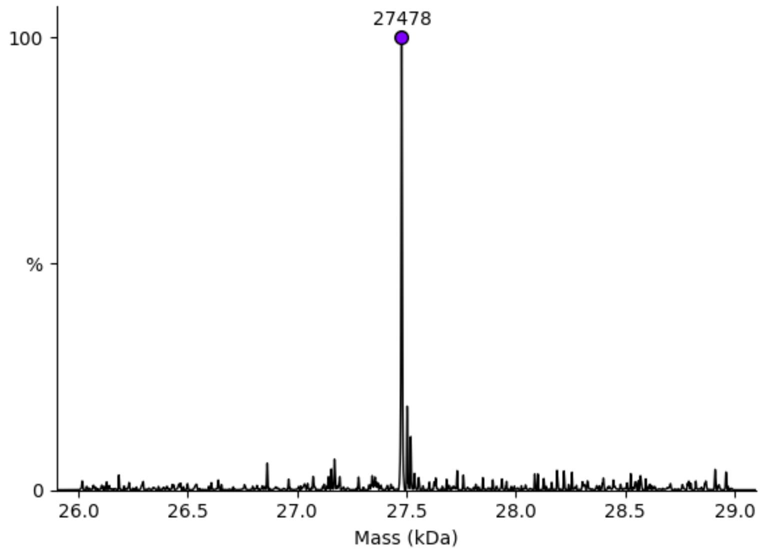

Figure S110. **87DNA_U^{She}**, deconvoluted spectrum, calculated mass: 27486.8 Da, found mass: 27478.0 (product).

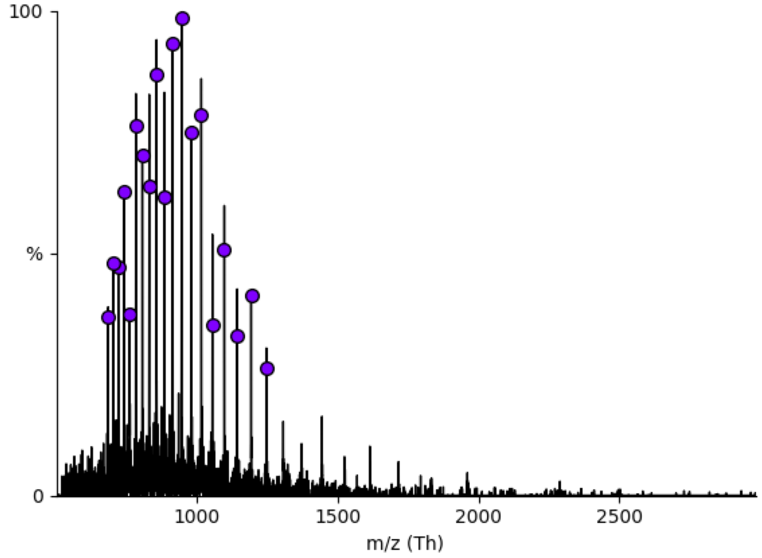

Figure S111. **87DNA_U^{ac}**, raw spectrum.

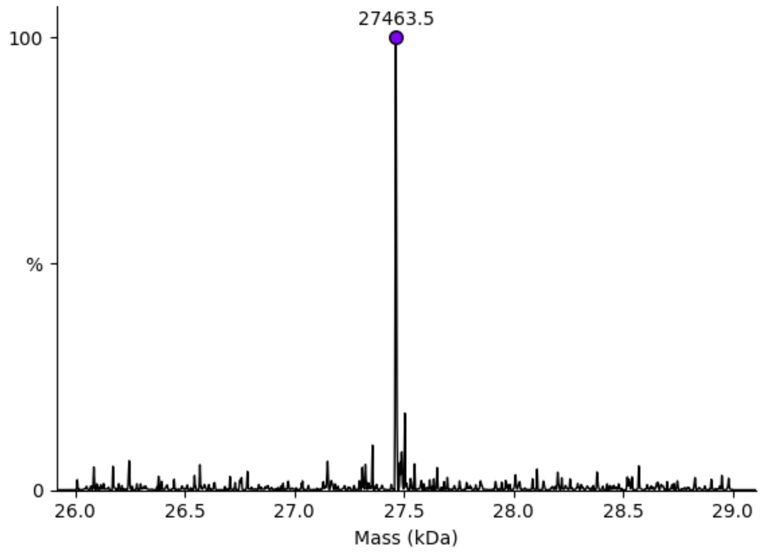

Figure S112. **87DNA_U^{ac}**, deconvoluted spectrum, calculated mass: 27472.7 Da, found mass: 27463.5 (product).

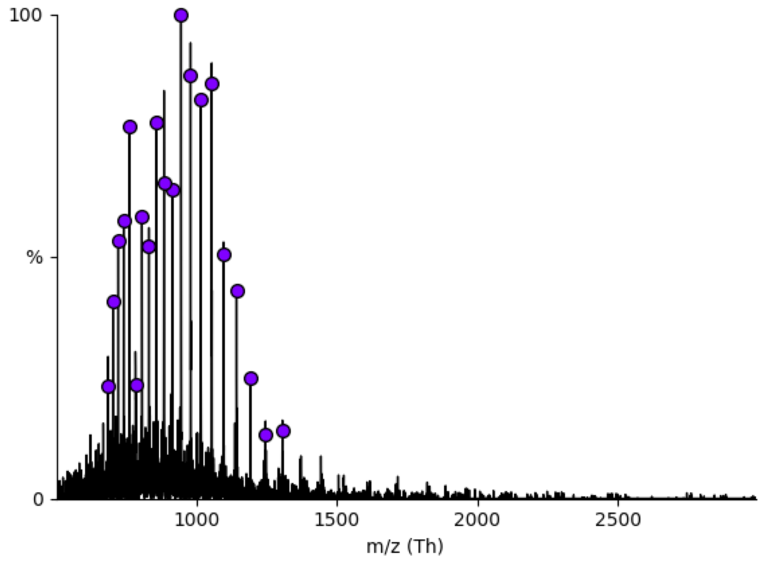

Figure S113. **87DNA_U^{cm}**, raw spectrum.

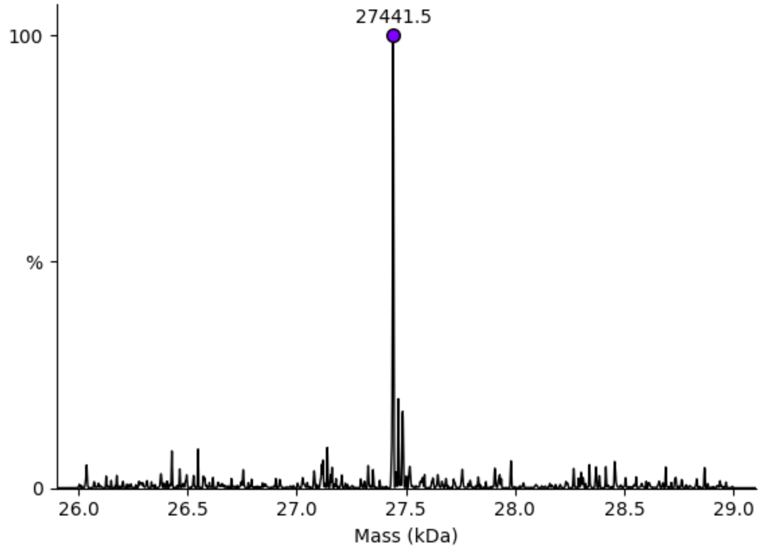

Figure S114. **87DNA_U^{cm}**, deconvoluted spectrum, calculated mass: 27451.7 Da, found mass: 27441.5 (product).

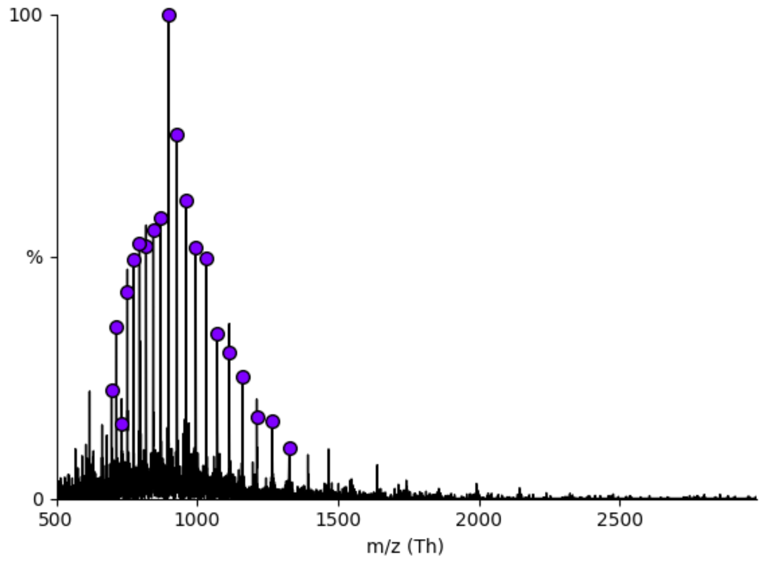

Figure S115. **87DNA_U^{dhp}**, raw spectrum.

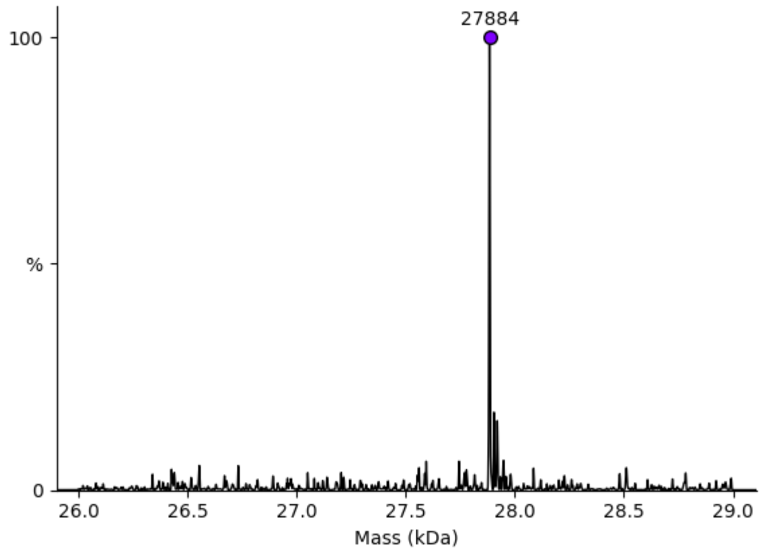

Figure S116. **87DNA_U^{dhp}**, deconvoluted spectrum, calculated mass: 27893.4 Da, found mass: 27884.0 (product).

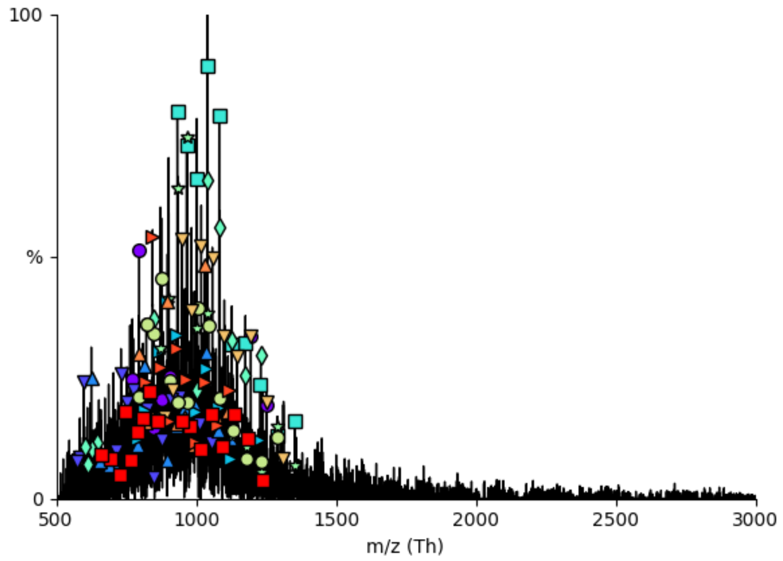

Figure S117. **87DNA_U^{glu}**, raw spectrum.

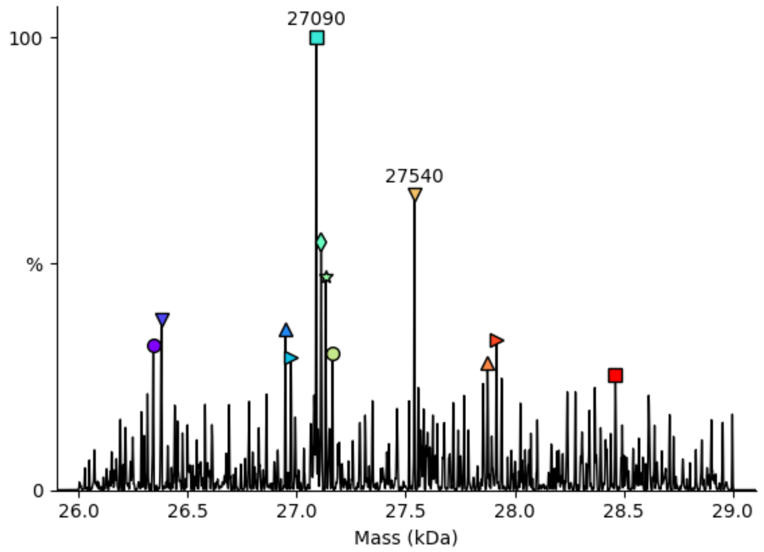

Figure S118. **87DNA_U^{glu}**, deconvoluted spectrum, calculated mass: 28292.4 Da, found mass: 27540.0 (modified strand lacking **dU^{glu}** and dA), found mass: 27090.0 (modified strand lacking two **dU^{glu}** and dA).

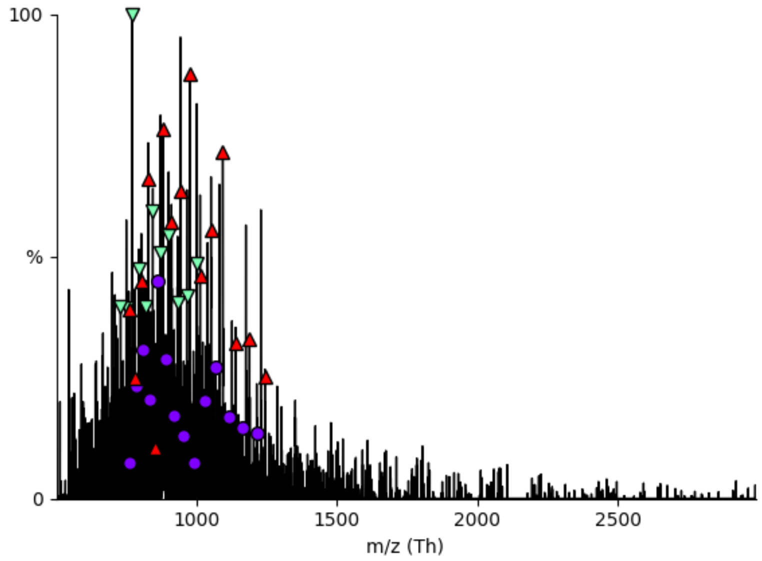

Figure S119. **87DNA_U^{am}**, raw spectrum.

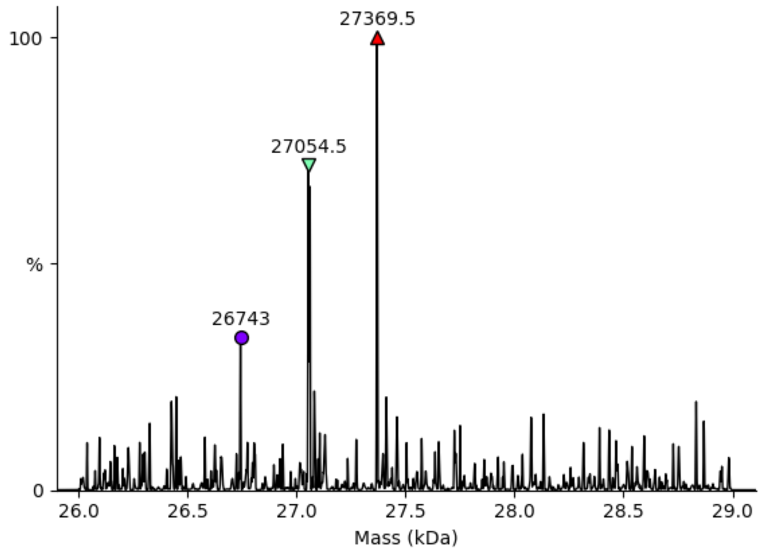

Figure S120. **87DNA_U^{am}**, deconvoluted spectrum, calculated mass: 27381.7 Da, found mass: 27369.5 (product), found mass: 27054.5 (modified strand lacking dA).

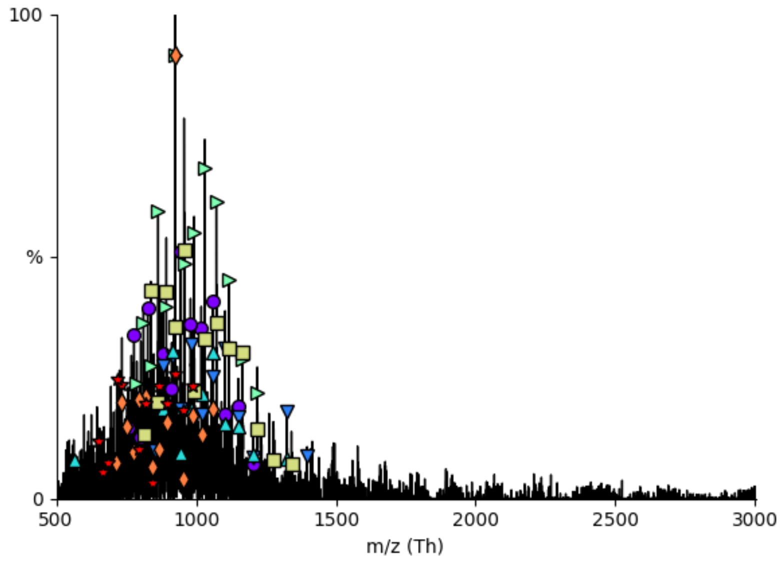

Figure S121. **87DNA_U^{mm}**, raw spectrum.

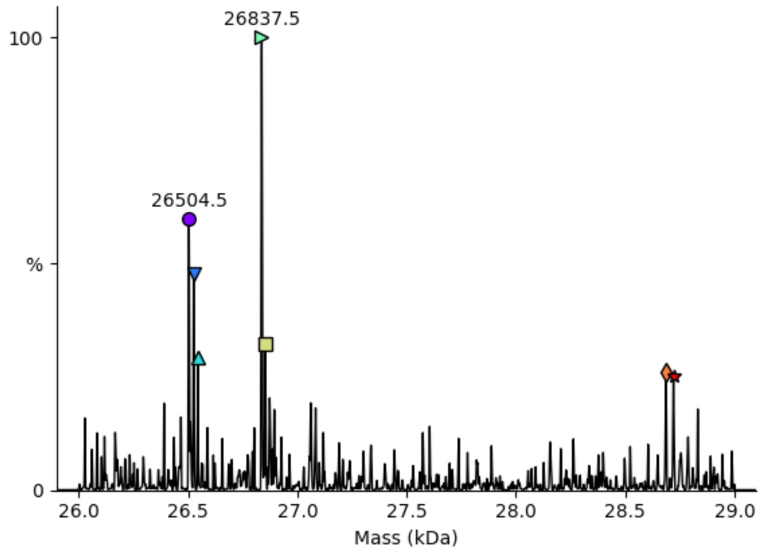

Figure S122. **87DNA_U^{mm}**, deconvoluted spectrum, calculated mass: 27479.9 Da, found mass: 26837.5 (modified strand lacking **dU^{mm}** and dA).

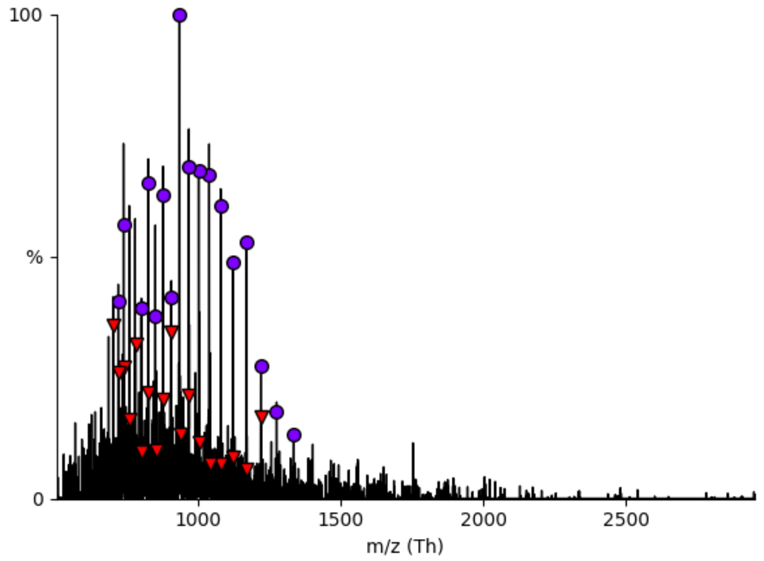

Figure S123. **87DNA_U^{tfa}**, raw spectrum.

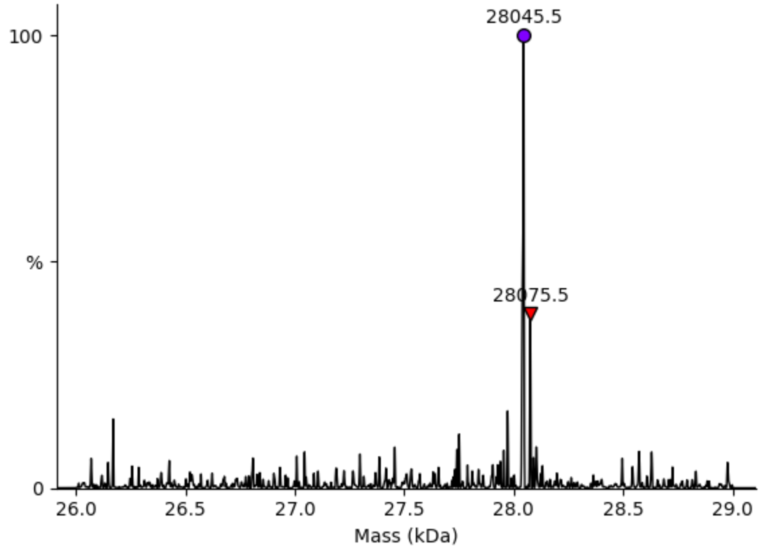

Figure S124. **87DNA_U^{tfa}**, deconvoluted spectrum, calculated mass: 28053.8 Da, found mass: 28045.5 (product).

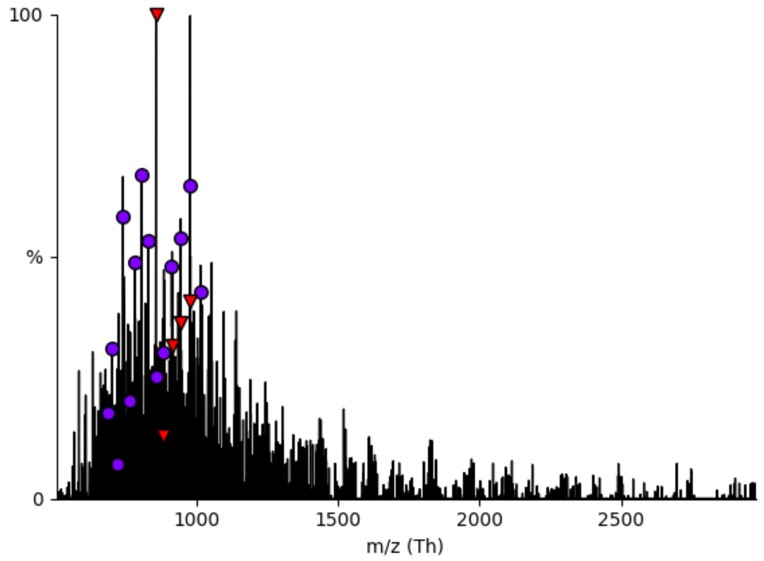

Figure S125. **87DNA_C^{hm}**, raw spectrum.

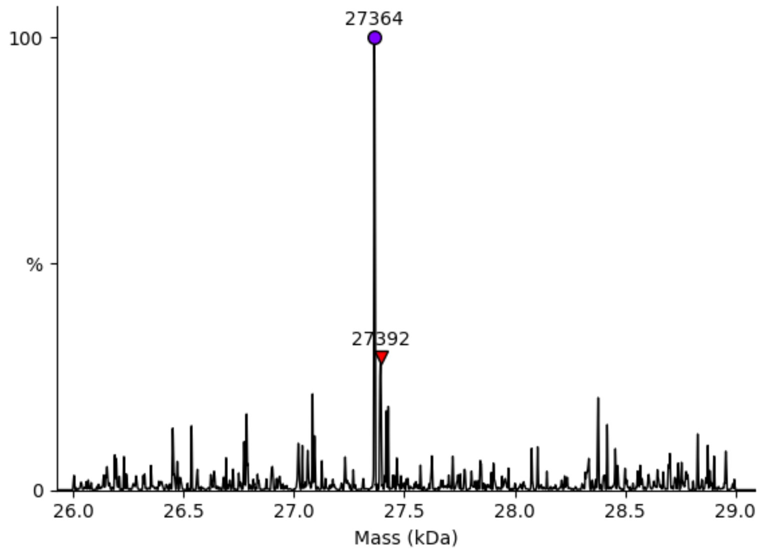

Figure S126. **87DNA_C^{hm}**, deconvoluted spectrum, calculated mass: 27366.7 Da, found mass: 27364.0 (product).

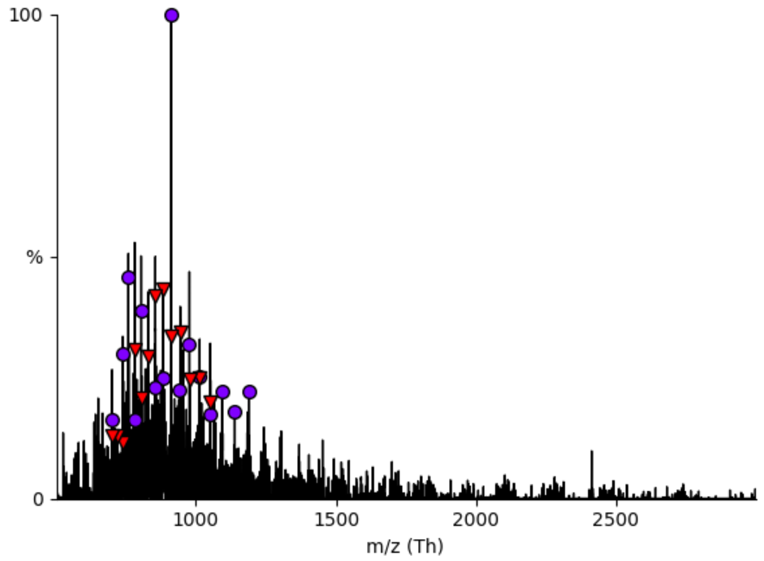

Figure S127. **87DNA_C^{et}**, raw spectrum.

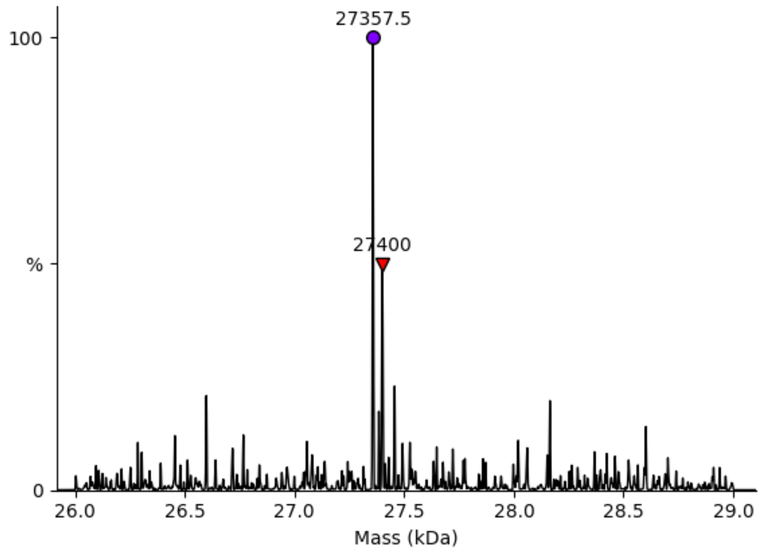

Figure S128. **87DNA_C^{et}**, deconvoluted spectrum, calculated mass: 27360.8 Da, found mass: 27357.5 (product).

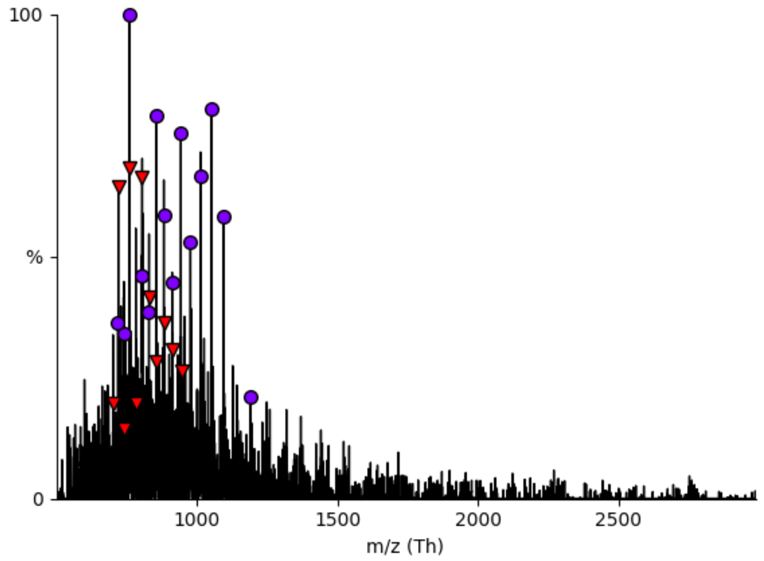

Figure S129. **87DNA_C^{She}**, raw spectrum.

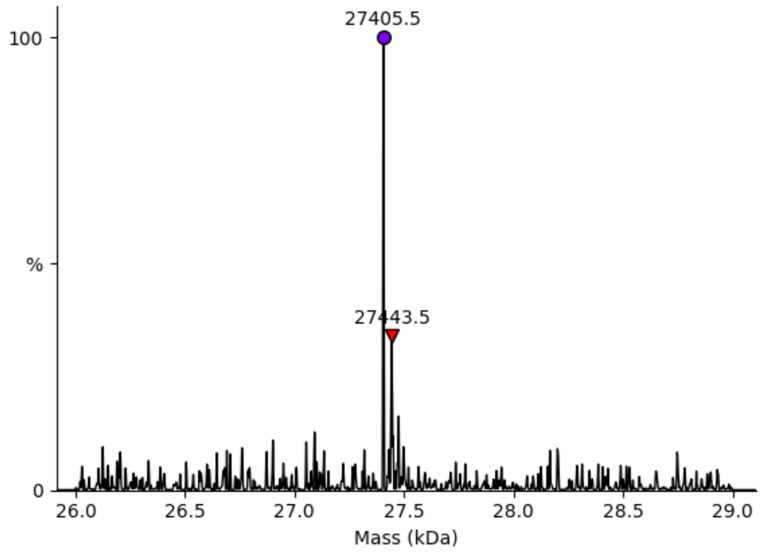

Figure S130. **87DNA_C^{She}**, deconvoluted spectrum, calculated mass: 27408.8 Da, found mass: 27405.5 (product).

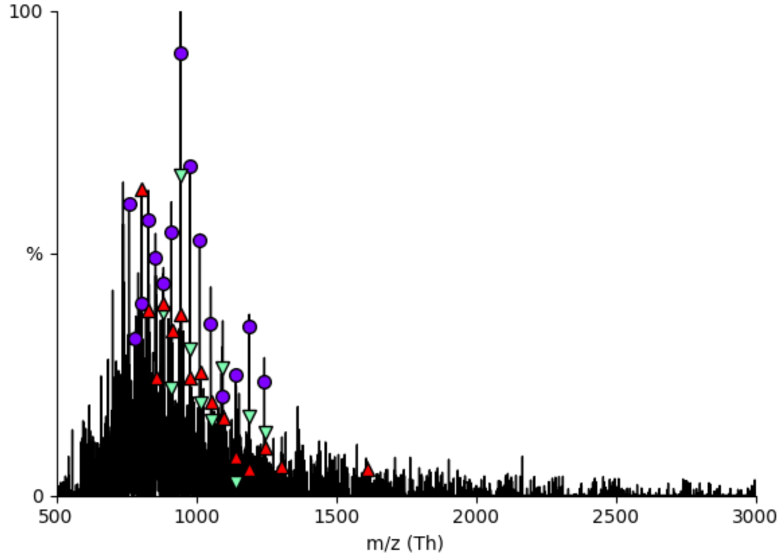

Figure S131. **87DNA_C**^{ac}, raw spectrum.

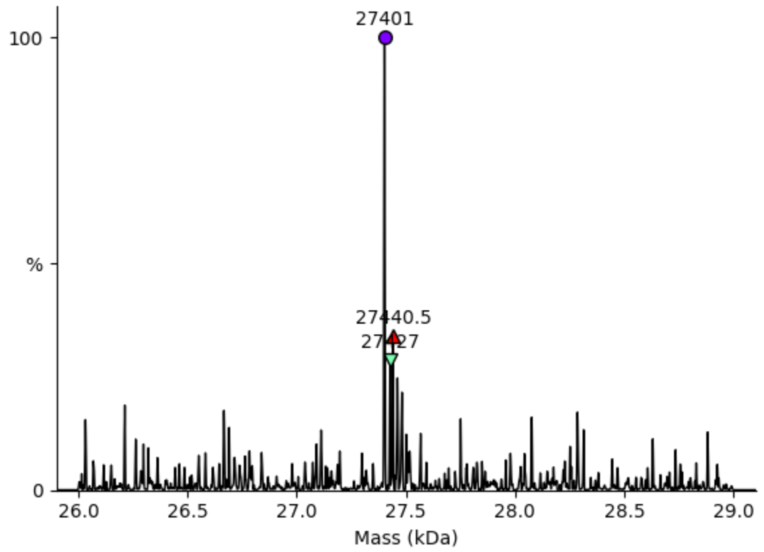

Figure S132. **87DNA_C**^{ac}, deconvoluted spectrum, calculated mass: 27402.7 Da, found mass: 27401.0 (product).

3.5. LC-MS spectra of 98DNA_PCR/98DNA_U^{tfa} and 98DNA_U^{am}

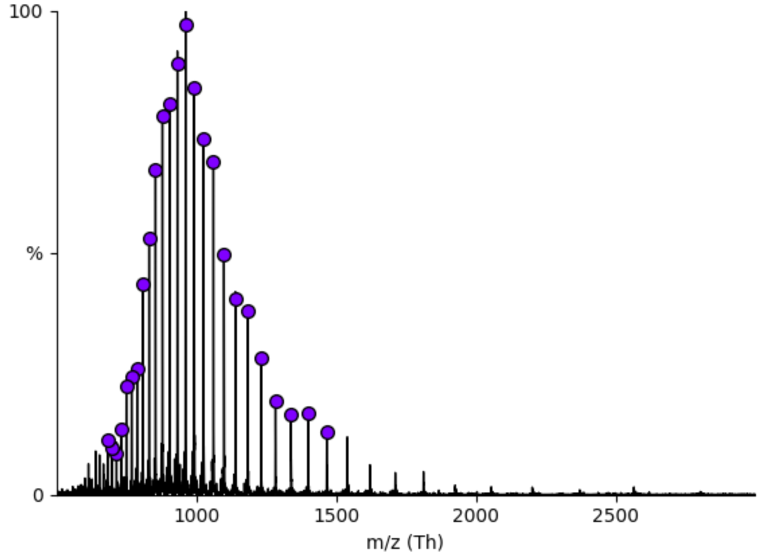

Figure S133. **98DNA_PCR** (sense strand), raw spectrum.

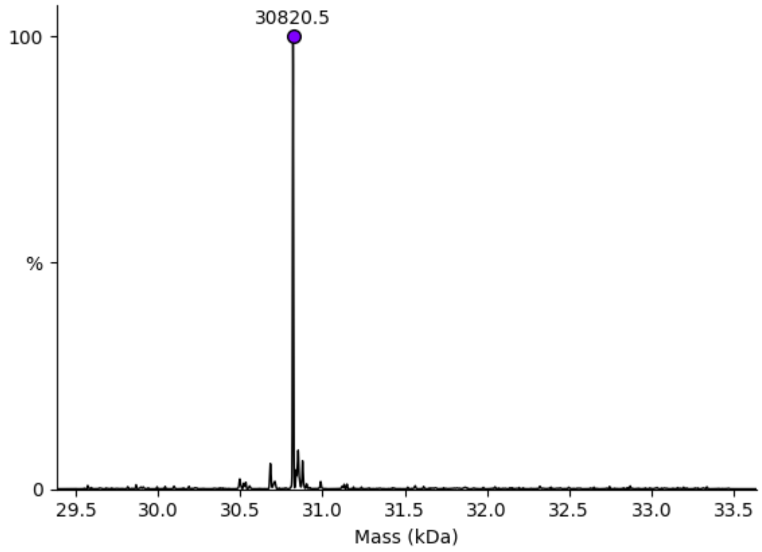

Figure S134. **98DNA_PCR** (sense strand), deconvoluted spectrum, calculated mass: 30823.7 Da, found mass: 30820.5 (product).

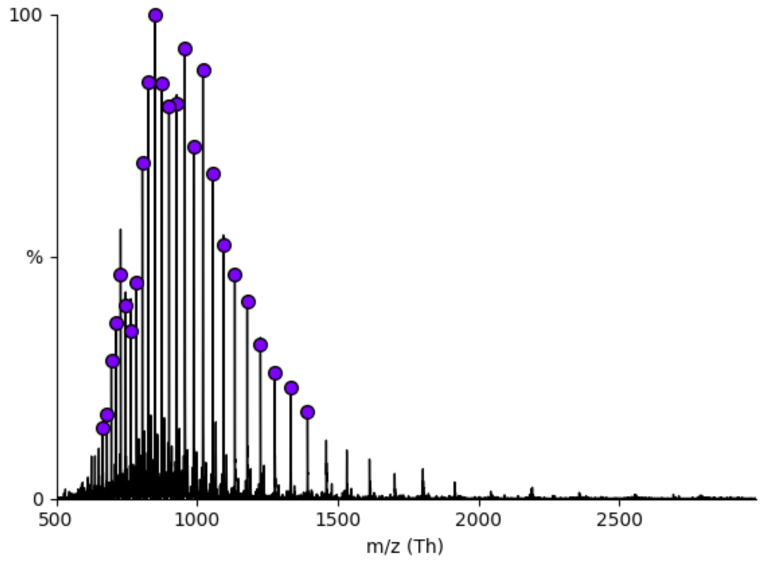

Figure S135. **98DNA_PCR** (antisense strand), raw spectrum.

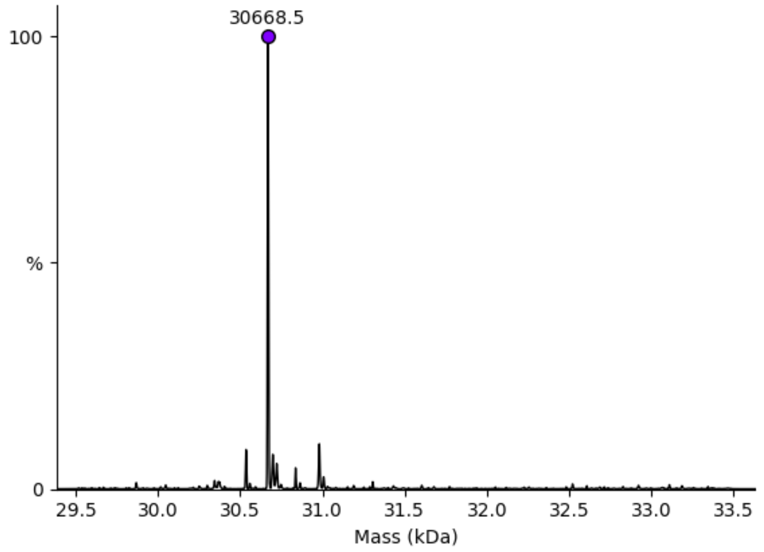

Figure S136. **98DNA_PCR** (antisense strand), deconvoluted spectrum, calculated mass: 30672.6 Da, found mass: 30668.5 (product).

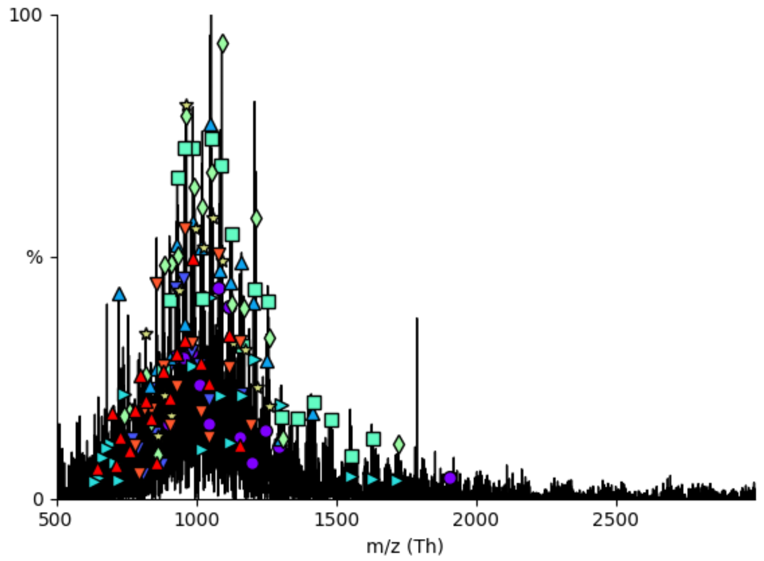

Figure S137. **98DNA_U^{tfa}** (sense strand), raw spectrum.

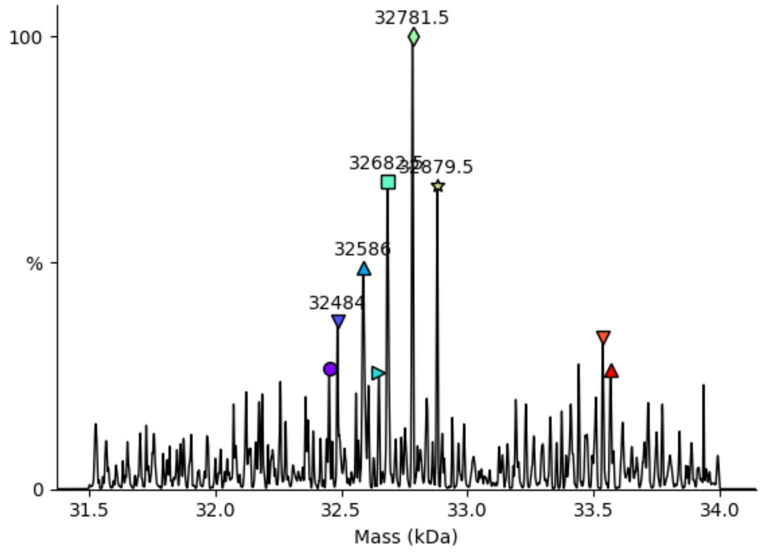

Figure S138. **98DNA_U^{tfa}** (sense strand), deconvoluted spectrum, calculated mass: 33266.2 Da, found mass: 32879.5, 32781.5, 32682.5, 32586.0 (assigned to product lacking 4 to 7 TFA protecting groups).

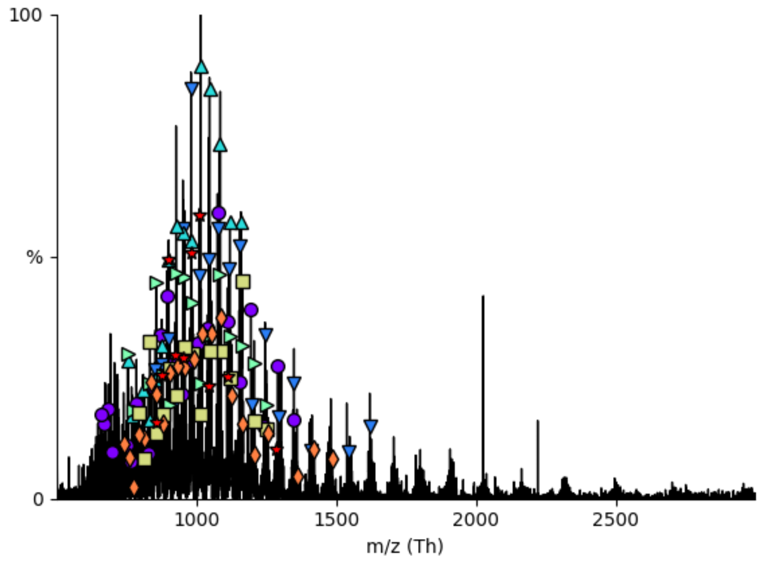

Figure S139. **98DNA_U^{tfa}** (antisense strand), raw spectrum.

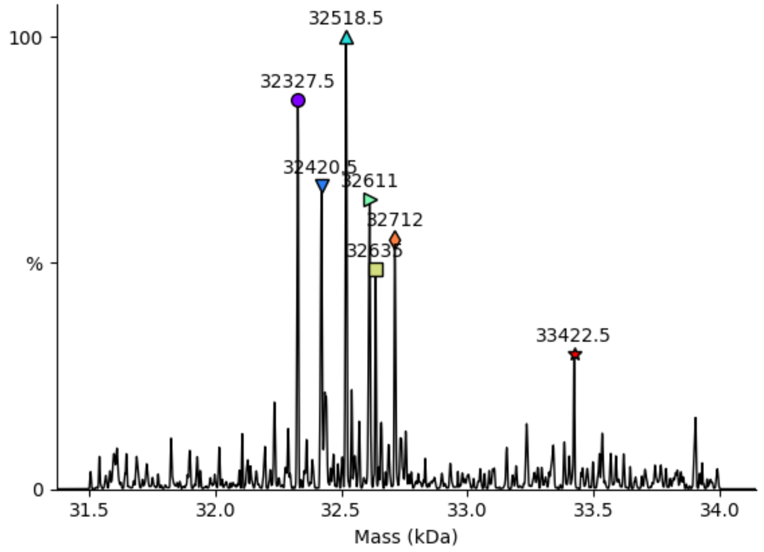

Figure S140. **98DNA_U^{tfa}** (antisense strand), deconvoluted spectrum, calculated mass: 33004.1 Da, found mass: 32712.0, 32611.0, 32518.5, 32420.5, 32327.5 (assigned to product lacking 3 to 7 TFA protecting groups).

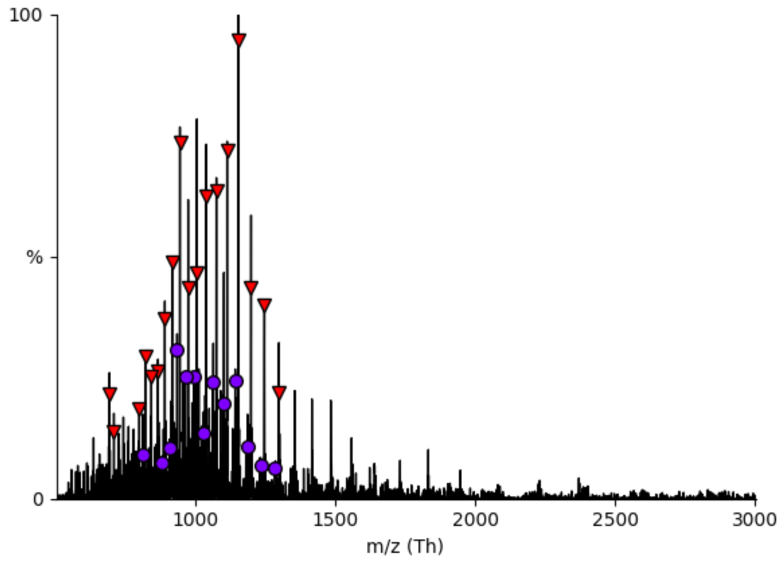

Figure S141. **98DNA_U^{am}** (sense strand, after desired deprotection of **98DNA_U^{tfa}**), raw spectrum.

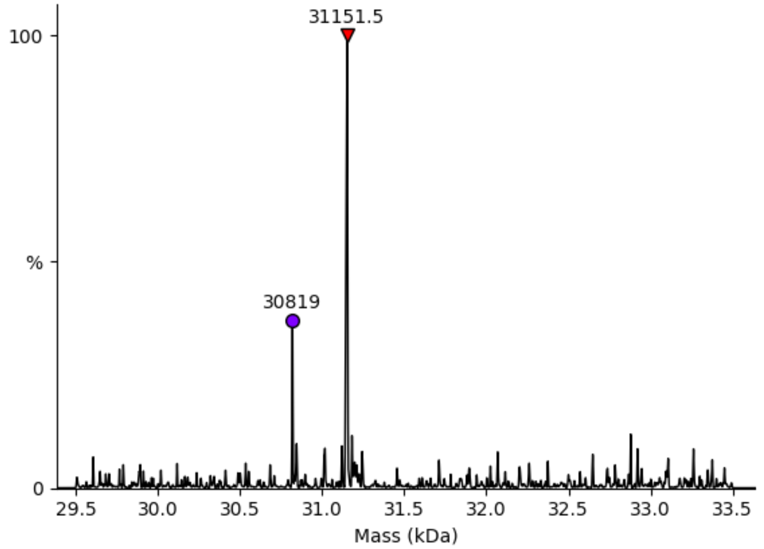

Figure S142. **98DNA_U^{am}** (sense strand, after desired deprotection of **98DNA_U^{tfa}**), deconvoluted spectrum, calculated mass: 31154.0 Da, found mass: 31151.5 (product).

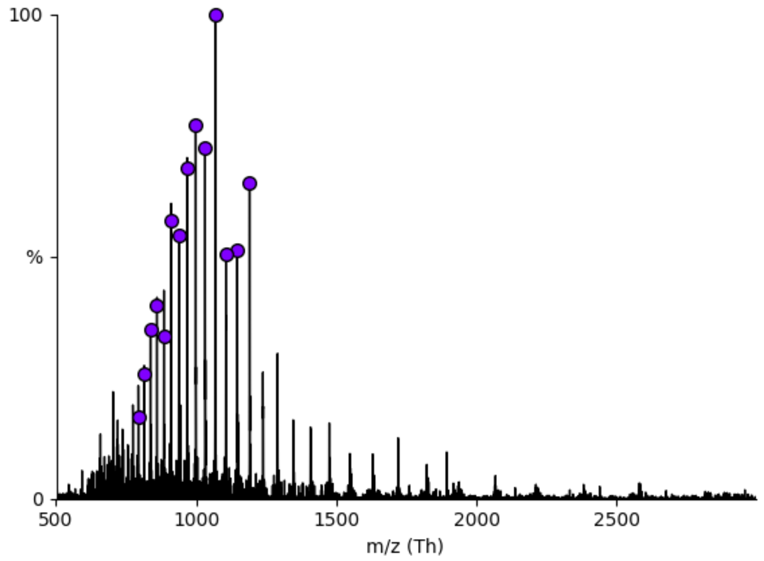

Figure S143. **98DNA_U^{am}** (antisense strand, after desired deprotection of **98DNA_U^{tfa}**), raw spectrum.

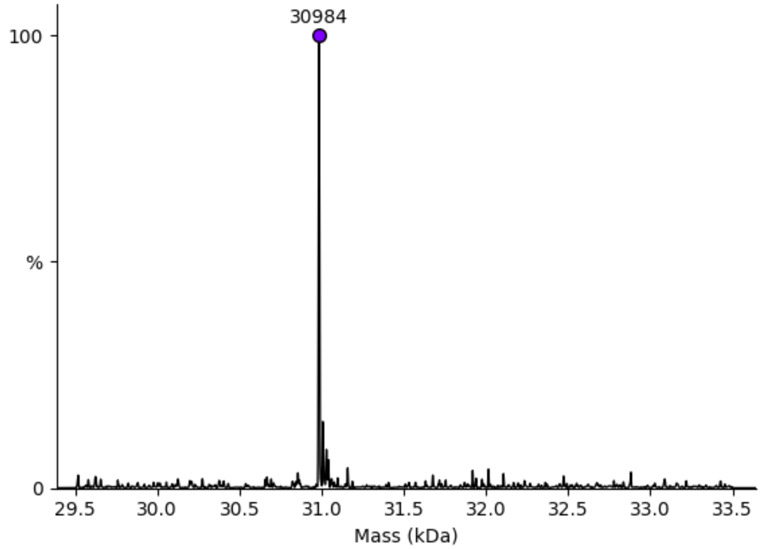

Figure S144. **98DNA_U^{am}** (antisense strand, after desired deprotection of **98DNA_U^{tfa}**), deconvoluted spectrum, calculated mass: 30987.9 Da, found mass: 30984.0 (product).

4. Copies of IR spectra

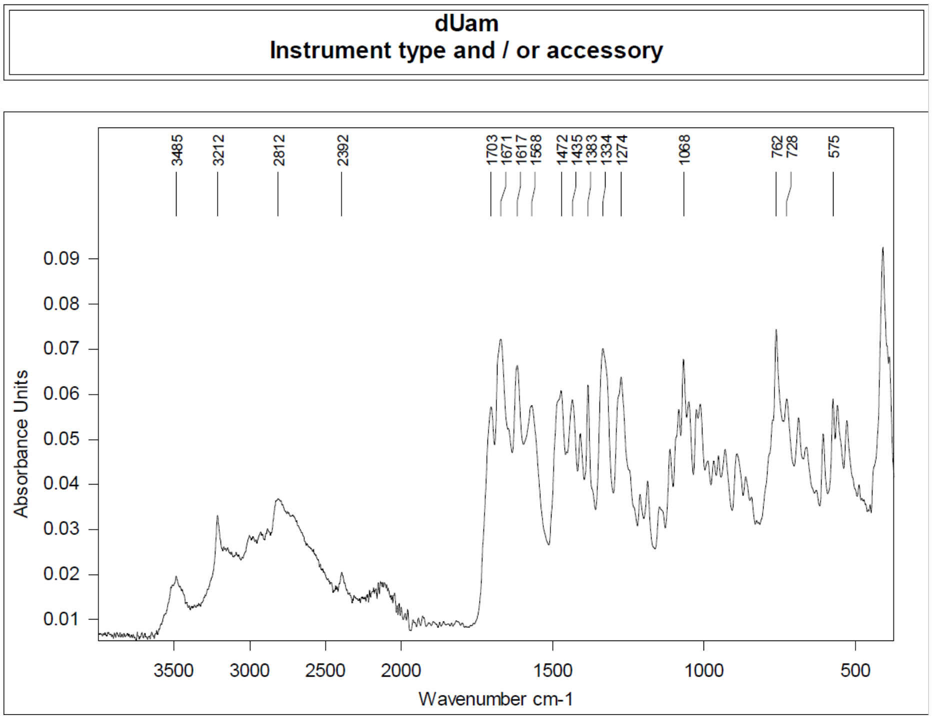

Figure S145. IR spectrum of **dU**$^{\mathbf{am}}$

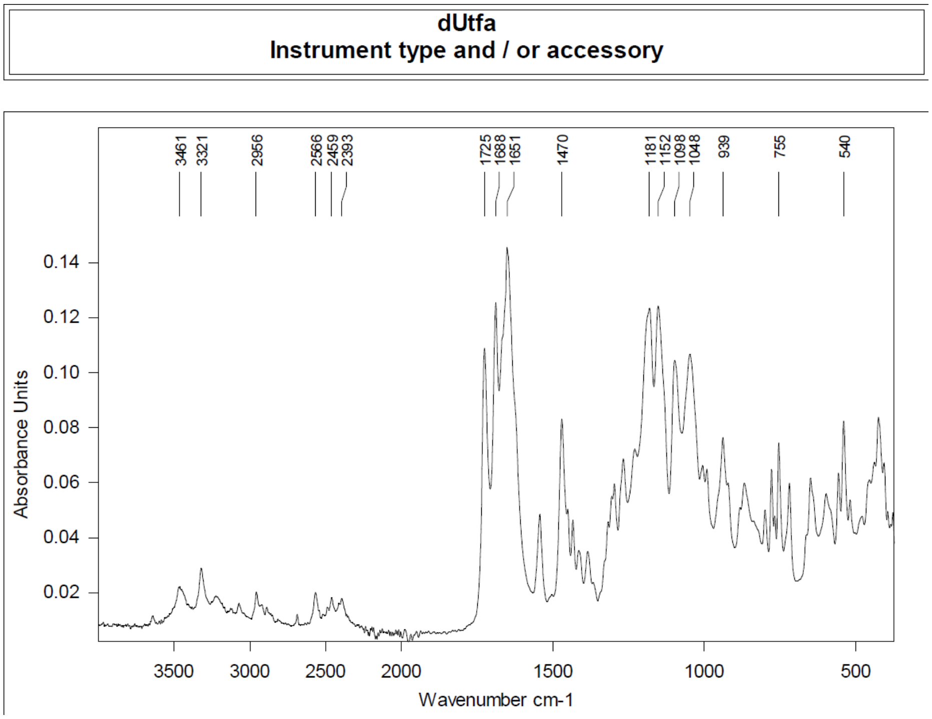

Figure S146. IR spectrum of **dU**$^{\mathbf{tfa}}$

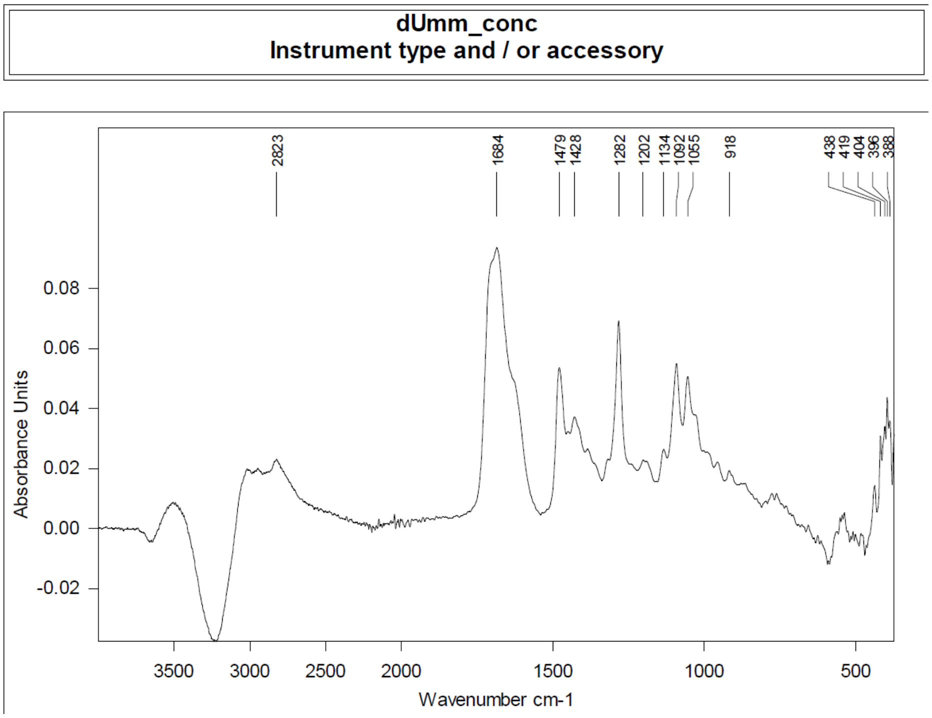

Figure S147. IR spectrum of **dU^{mm}**

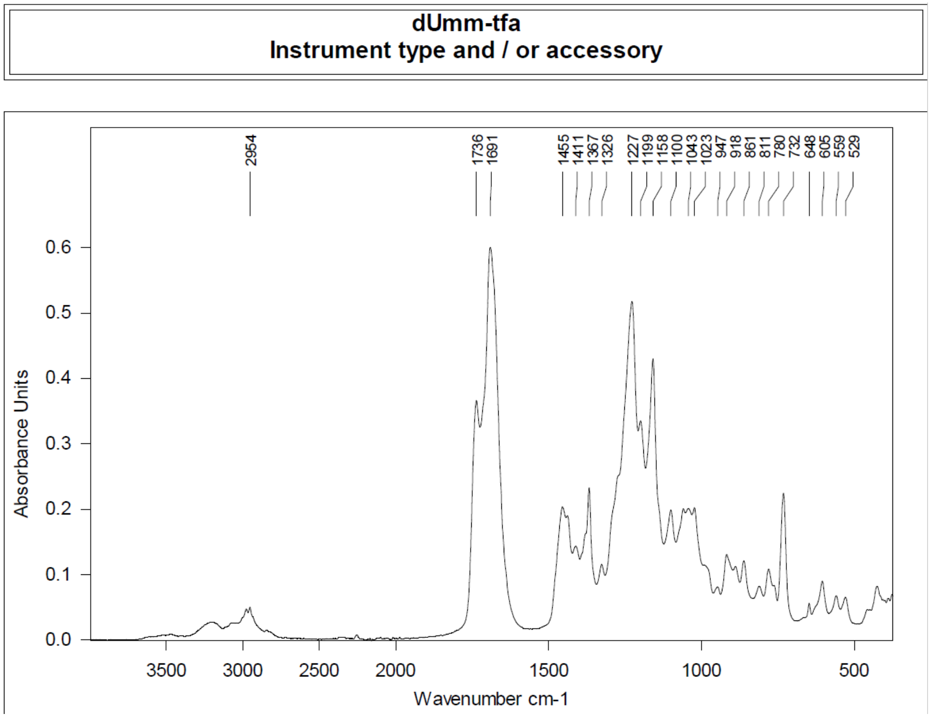

Figure S148. IR spectrum of **dU$^{mm\text{-}tfa}$**

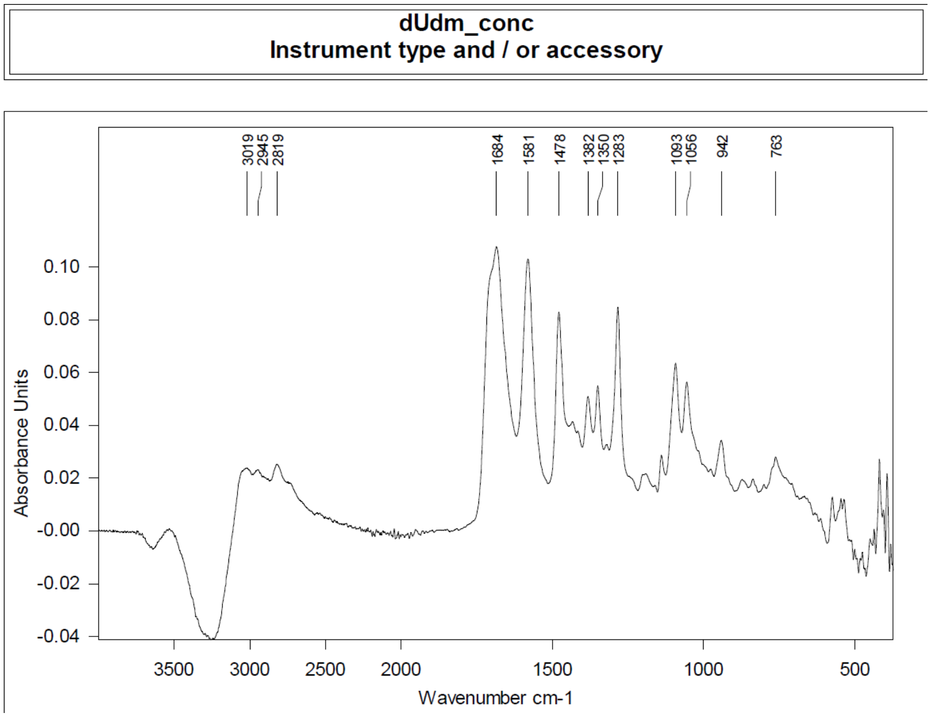

Figure S149. IR spectrum of **dU^{dm}**

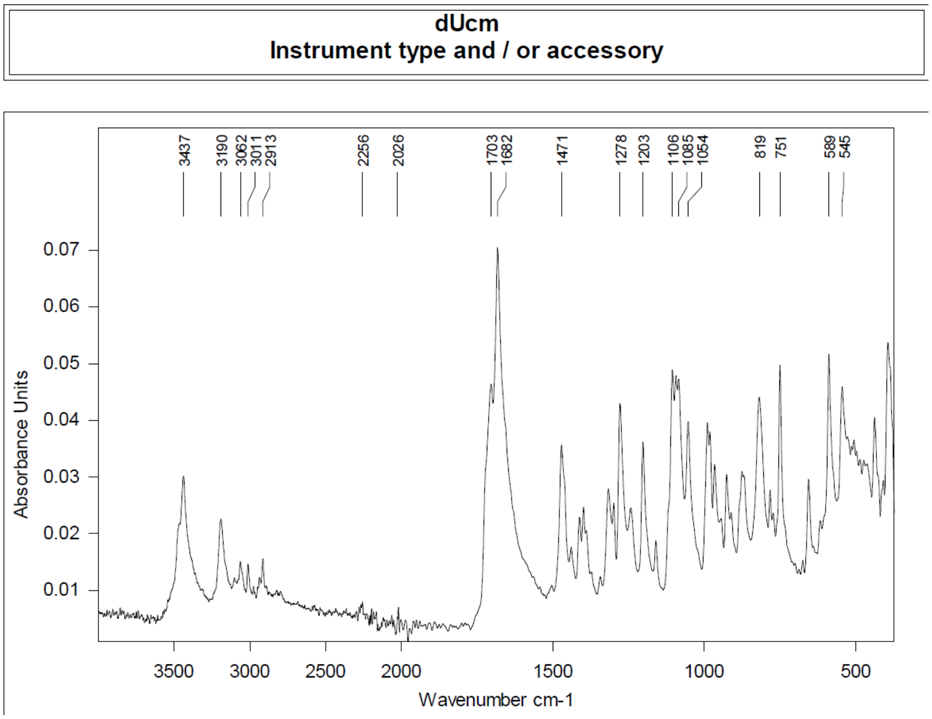

Figure S150. IR spectrum of **dU^{cm}**

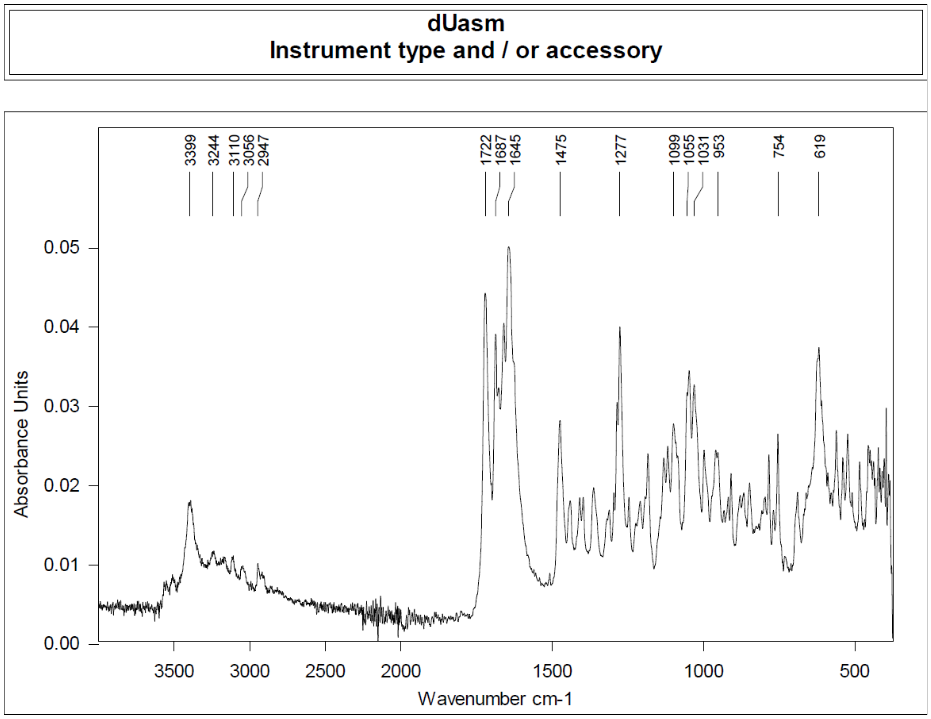

Figure S151. IR spectrum of **dU^{asm}**

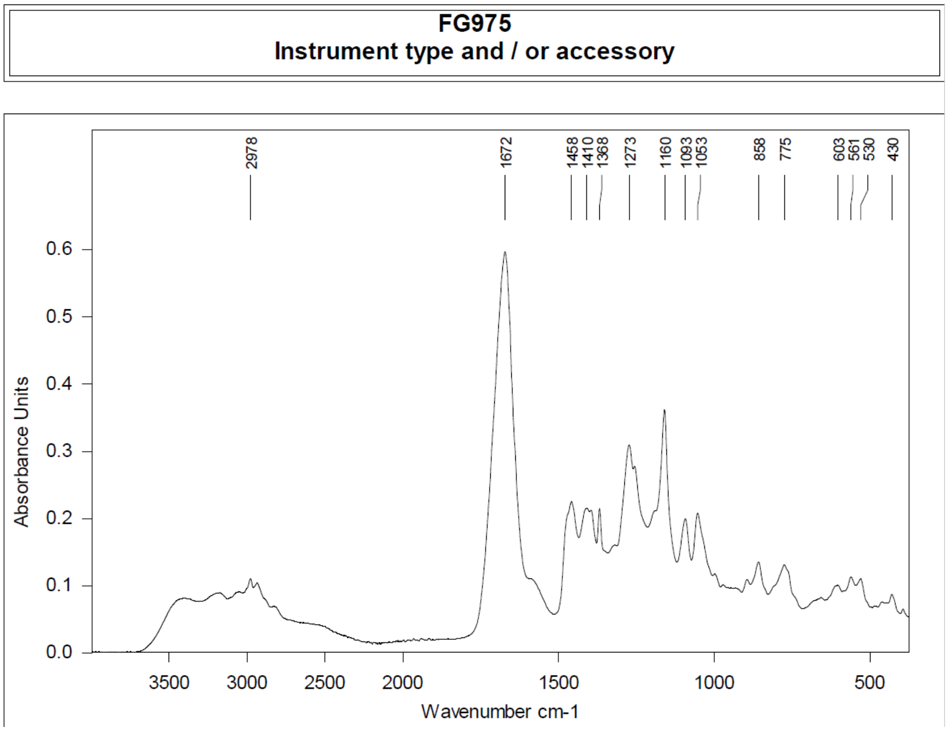

Figure S152. IR spectrum of **6**

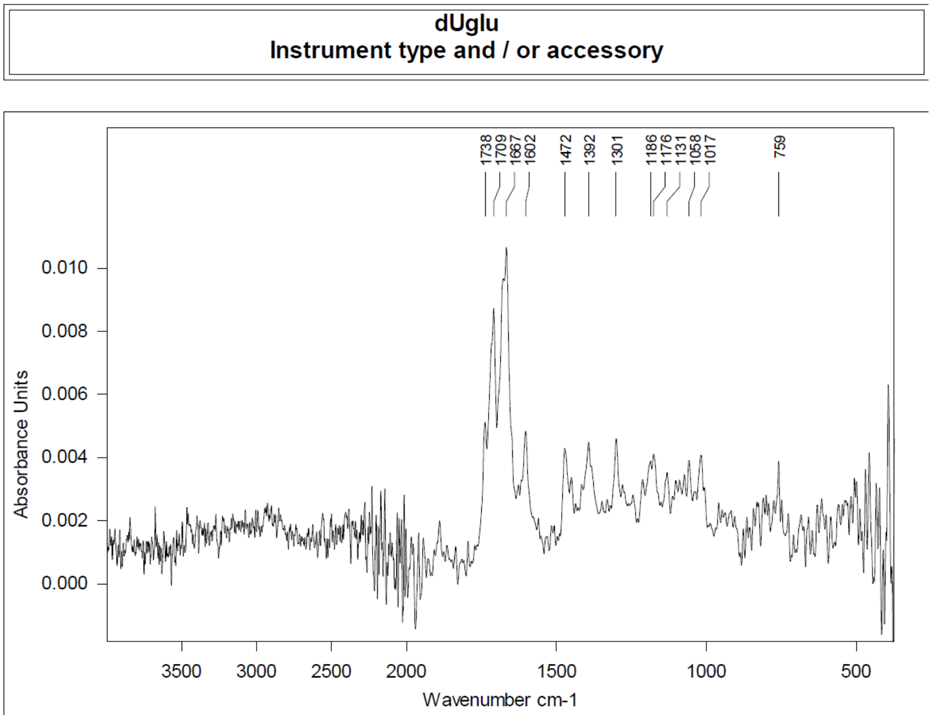

Figure S153. IR spectrum of **dU**$^{\mathbf{glu}}$

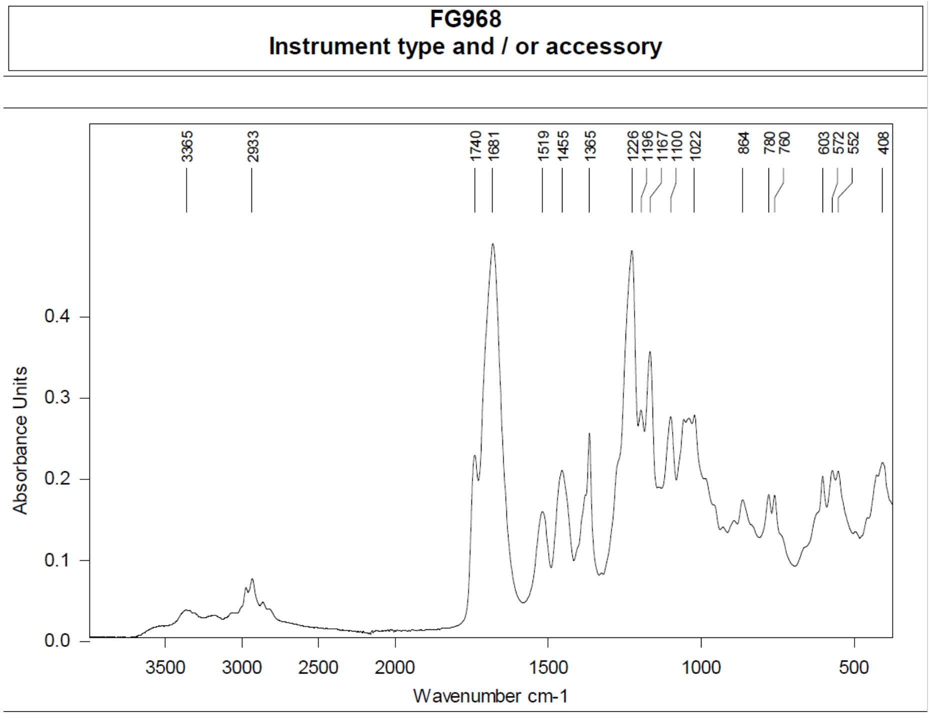

Figure S154. IR spectrum of **7**

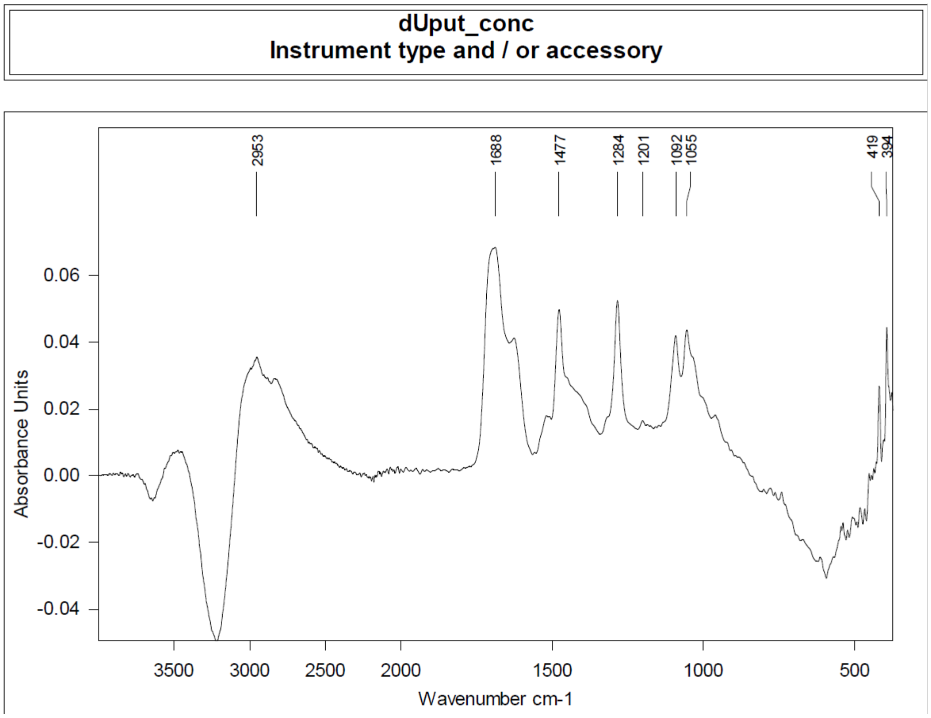

Figure S155. IR spectrum of **dU^{put}**

5. Next generation sequencing

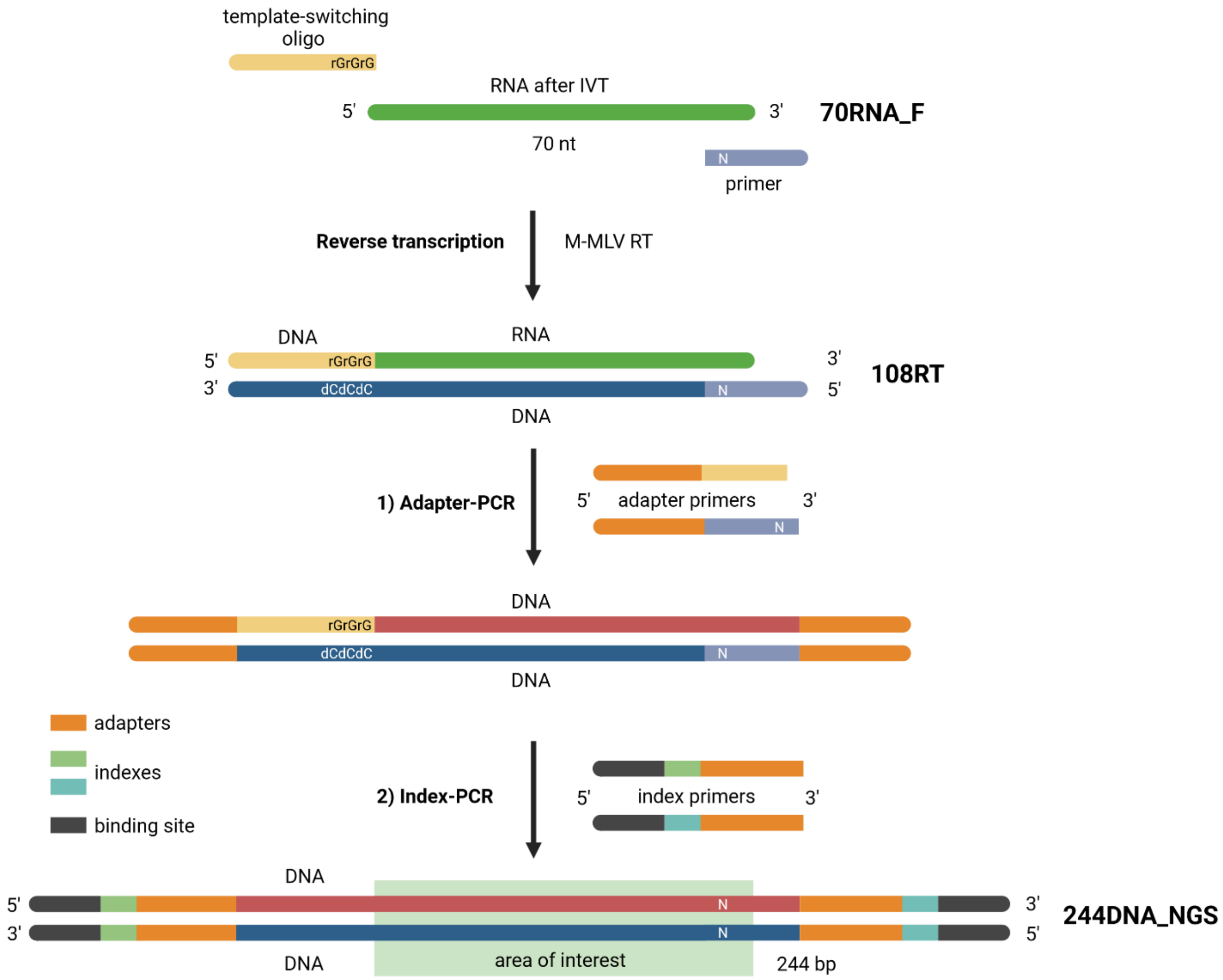

Figure S156. Preparation of NGS-ready DNA sequences from RNA. Created in BioRender. Gracias, F. (2024) BioRender.com/x27d527

5.1. NGS results

NGS of 244DNA_NGS_1

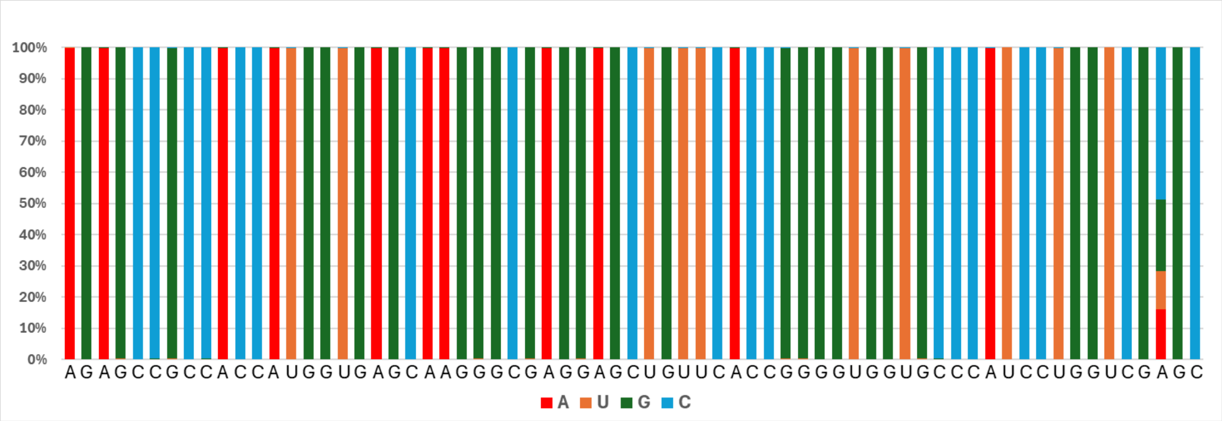

Figure S157. Fidelity visualisation of RNA produced by **107DNA_F**. Sequence below represents target RNA. First 3 nucleotides (GGG) are not included.

NGS of 244DNA_NGS_2

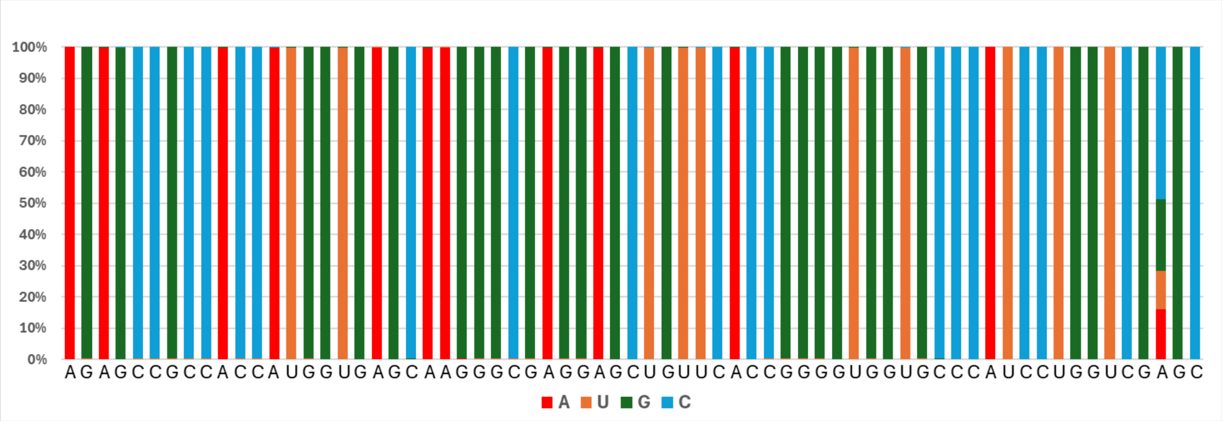

Figure S158. Fidelity visualisation of RNA produced by **107DNA_F_U^{hm}**. Sequence below represents target RNA. First 3 nucleotides (GGG) are not included.

NGS of 244DNA_NGS_3

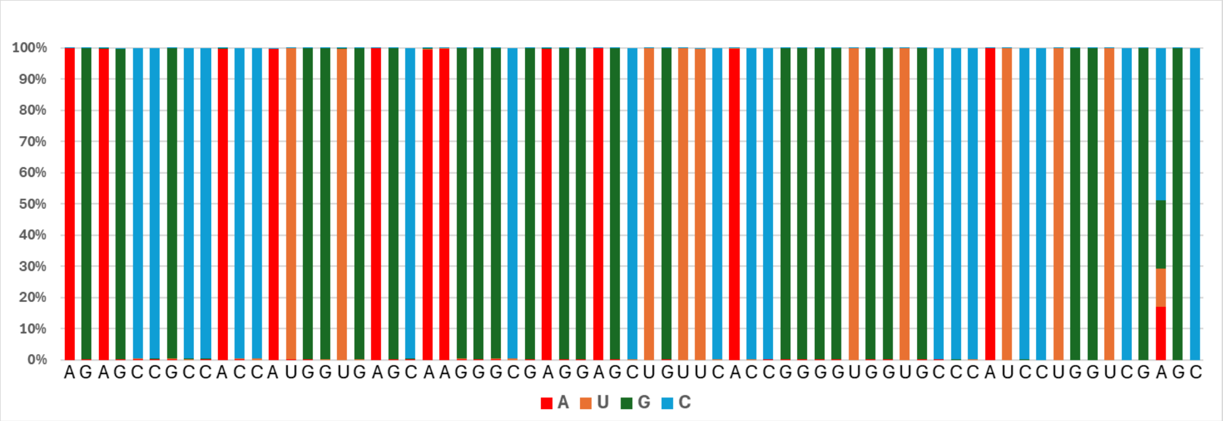

Figure S159. Fidelity visualisation of RNA produced by **107DNA_F_U^{et}**. Sequence below represents target RNA. First 3 nucleotides (GGG) are not included.

NGS of 244DNA_NGS_4

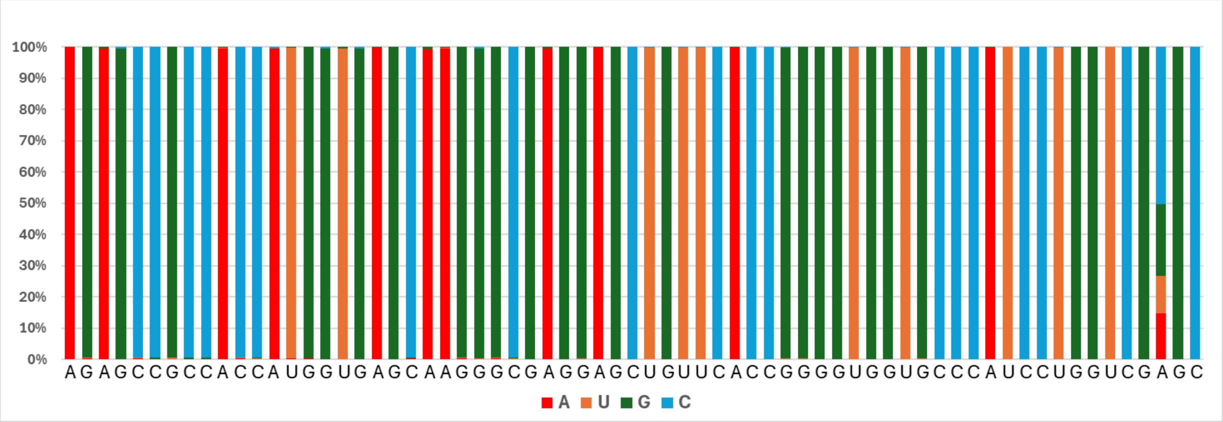

Figure S160. Fidelity visualisation of RNA produced by **107DNA_F_U^{She}**. Sequence below represents target RNA. First 3 nucleotides (GGG) are not included.

NGS of 244DNA_NGS_5

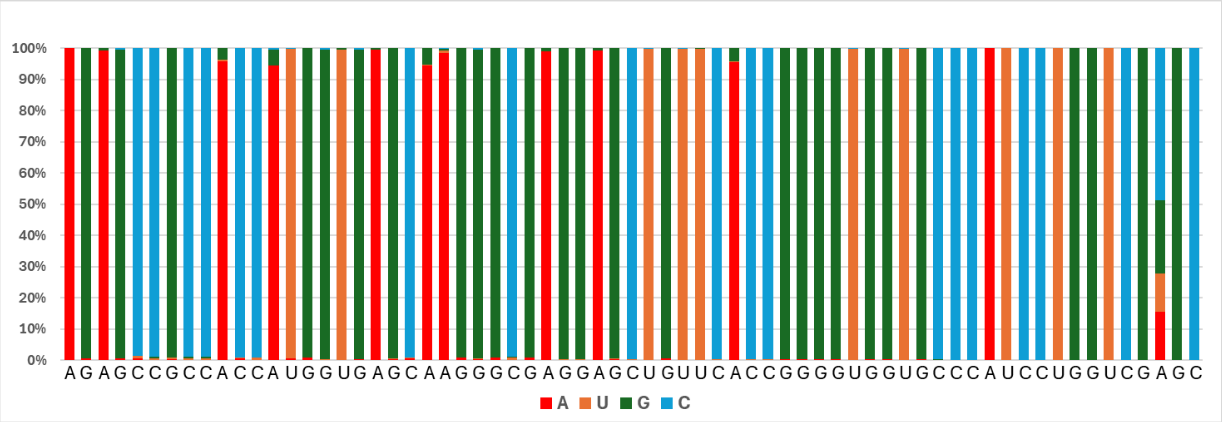

Figure S161. Fidelity visualisation of RNA produced by **107DNA_F_U^{ac}**. Sequence below represents target RNA. First 3 nucleotides (GGG) are not included. Visible rA→rG mutations present.

NGS of 244DNA_NGS_6

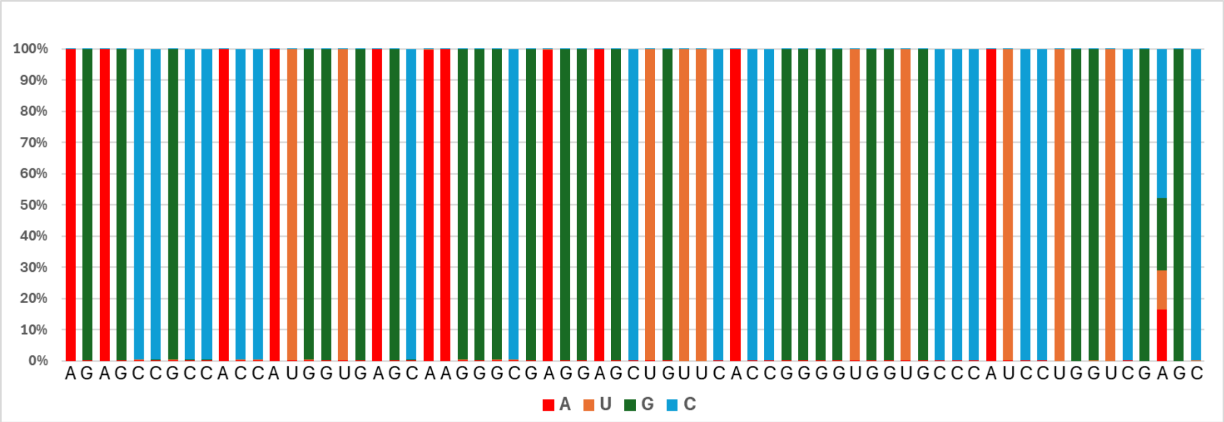

Figure S162. Fidelity visualisation of RNA produced by **107DNA_F_U^{cm}**. Sequence below represents target RNA. First 3 nucleotides (GGG) are not included.

6. Supplementary references

1 Kodr, D.; Yenice, C. P.; Simonova, A.; Saftić, D. P.; Pohl, R.; Sýkorová, V.; Ortiz, M.; Havran, L.; Fojta, M.; Lesnikowski, Z. J.; *et al.* Carborane- or Metallacarborane-Linked Nucleotides for Redox Labeling. Orthogonal Multipotential Coding of All Four DNA Bases for Electrochemical Analysis and Sequencing. *Journal of the American Chemical Society* **2021**, *143*, 7124–7134.

2 Gracias, F.; Ruiz-Larrabeiti, O.; Vaňková Hausnerová, V.; Pohl, R.; Klepetářová, B.; Sýkorová, V.; Krásný, L.; Hocek, M. Homologues of Epigenetic Pyrimidines: 5-Alkyl-, 5-Hydroxyalkyl and 5-Acyluracil and -Cytosine Nucleotides: Synthesis, Enzymatic Incorporation into DNA and Effect on Transcription with Bacterial RNA Polymerase. *RSC Chemical Biology* **2022**, *3*, 1069–1075.

3 Pospíšil, Š.; Panattoni, A.; Gracias, F.; Sýkorová, V.; Hausnerová, V. V.; Vítovská, D.; Šanderová, H.; Krásný, L.; Hocek, M. Epigenetic Pyrimidine Nucleotides in Competition with Natural dNTPs as Substrates for Diverse DNA Polymerases. *ACS Chemical Biology* **2022**, *17*, 2781–2788.

4 No, Z.; Shin, D. S.; Song, B. J.; Ahn, M.; Ha, D.-C. A Facile One-Pot Synthesis of 2,3’-Anhydro-2’-Deoxyuridines via 3’-O-Imidazolylsulfonates. *Synthetic Communications* **2000**, *30*, 3873–3882.

5 Shiau, G. T.; Schinazi, R. F.; Chen, M. S.; Prusoff, W. H. Synthesis and Biological Activities of 5-(Hydroxymethyl, Azidomethyl, or Aminomethyl)-2’-Deoxyuridine and Related 5’-Substituted Analogs. *Journal of Medicinal Chemistry* **1980**, *23*, 127–133.

6 Takeda, T.; Ikeda, K.; Mizuno, Y.; Ueda, T. Synthesis and Properties of Deoxyoligonucleotides Containing Putrescinylthymine (Nucleosides and Nucleotides. LXXVI). *Chemical and Pharmaceutical Bulletin* **1987**, *35*, 3558–3567.

7 Edelman, M. S.; Barfknecht, R. L.; Huet-Rose, R.; Boguslawski, S.; Mertes, M. P. Thymidylate Synthetase Inhibitors. Synthesis of N-Substituted 5-Aminomethyl-2’-Deoxyuridine 5’-Phosphates. *Journal of Medicinal Chemistry* **1977**, *20*, 669–673.

8 Bornemann, B.; Marx, A. Synthesis of DNA Oligonucleotides Containing 5-(Mercaptomethyl)-2′-Deoxyuridine Moieties. *Bioorganic & Medicinal Chemistry* **2006**, *14*, 6235–6238.

9 Donohoe, T. J.; Ironmonger, A.; Kershaw, N. M. Synthesis of (−)-(z)-deoxypukalide. *Angewandte Chemie International Edition* **2008**, *47*, 7314–7316.

10 Marty, M. T.; Baldwin, A. J.; Marklund, E. G.; Hochberg, G. K.; Benesch, J. L.; Robinson, C. V. Bayesian Deconvolution of Mass and Ion Mobility Spectra: From Binary Interactions to Polydisperse Ensembles. *Analytical Chemistry* **2015**, *87*, 4370–4376.
