# Supplementary material for: Bacteriophage-related epigenetic natural and non-natural pyrimidine nucleotides and their influence on transcription with T7 RNA polymerase
Source: Commun Chem. 2024 Nov 9;7:256. doi: 10.1038/s42004-024-01354-5 (PMC11550810; doi:10.1038/s42004-024-01354-5)
Supplement: Supplementary file 3 — Description of Additional Supplementary Files [file 42004_2024_1354_MOESM3_ESM.pdf]

Description of Additional Supplementary Files

File name: Supplementary Data 1

Description: Copies of NMR spectra
