# Supplementary material for: Bacteriophage-related epigenetic natural and non-natural pyrimidine nucleotides and their influence on transcription with T7 RNA polymerase
Source: Commun Chem. 2024 Nov 9;7:256. doi: 10.1038/s42004-024-01354-5 (PMC11550810; doi:10.1038/s42004-024-01354-5)

**$^{1}$H NMR (401.0 MHz, $D_2O$), compound dU$^{am}$**

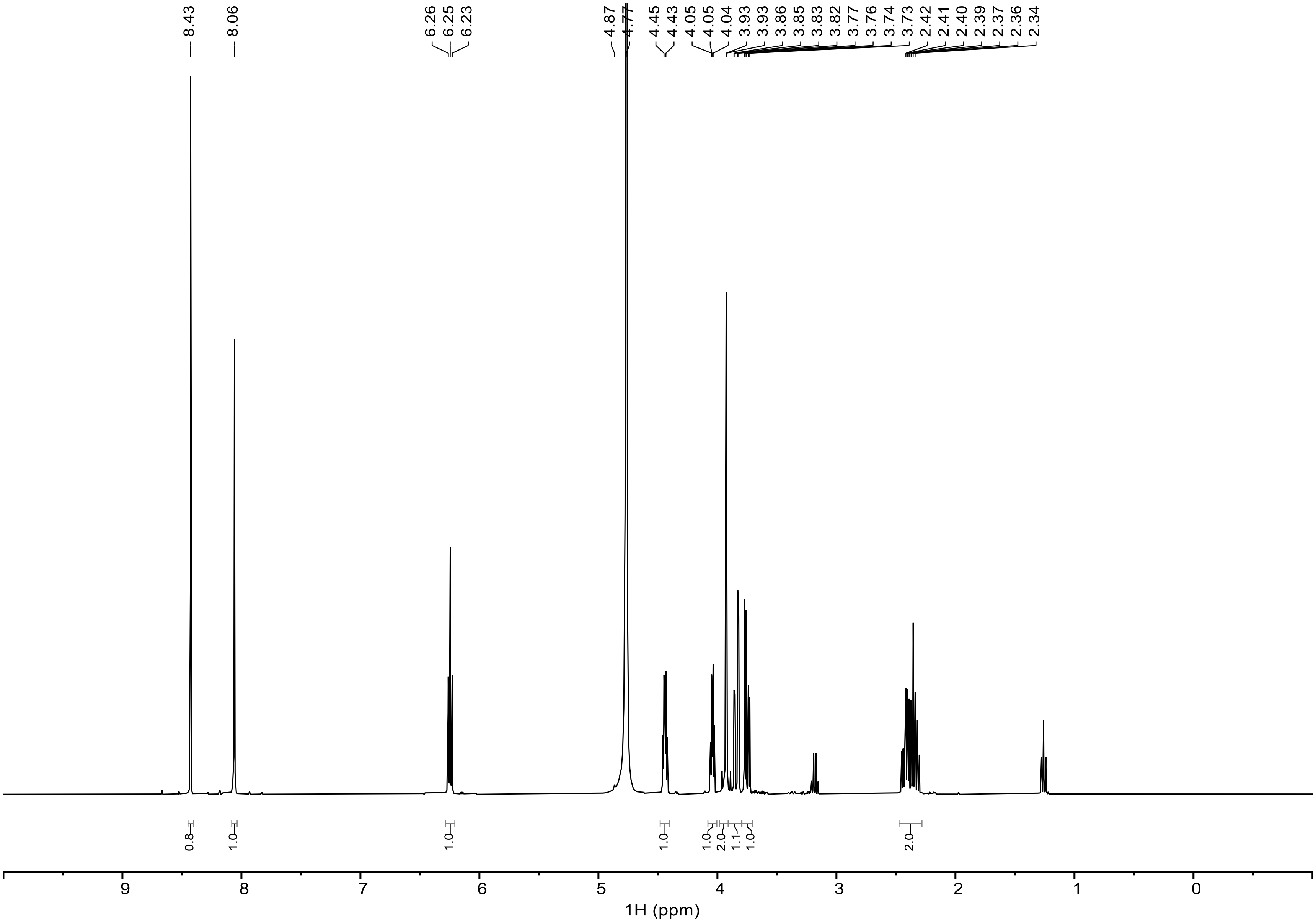

**$^{13}$C APT NMR (100.8 MHz, $D_2O$), compound dU$^{am}$**

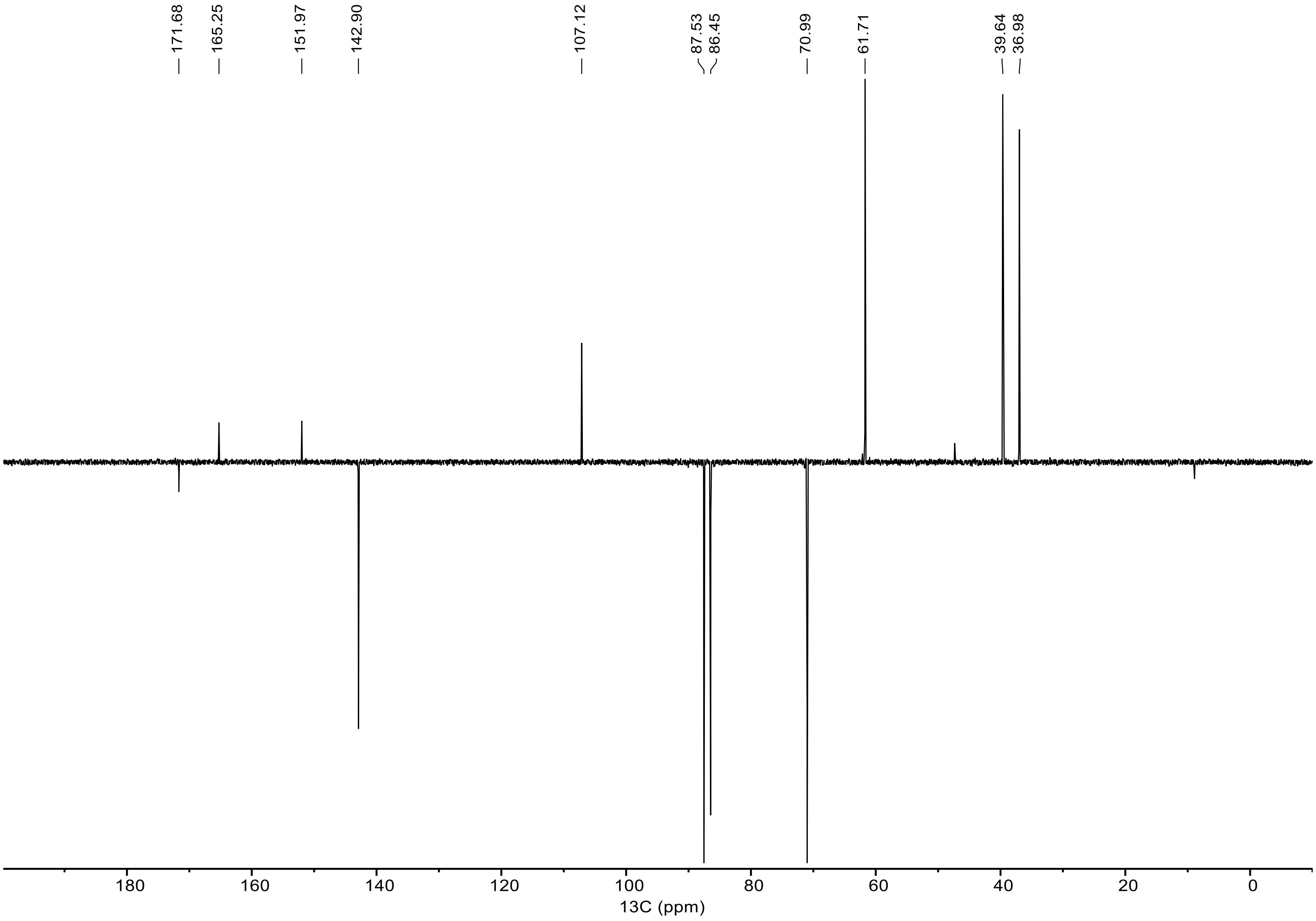

**$^1$H NMR (401.0 MHz, $CD_3OD$), compound dU$^{tfa}$**

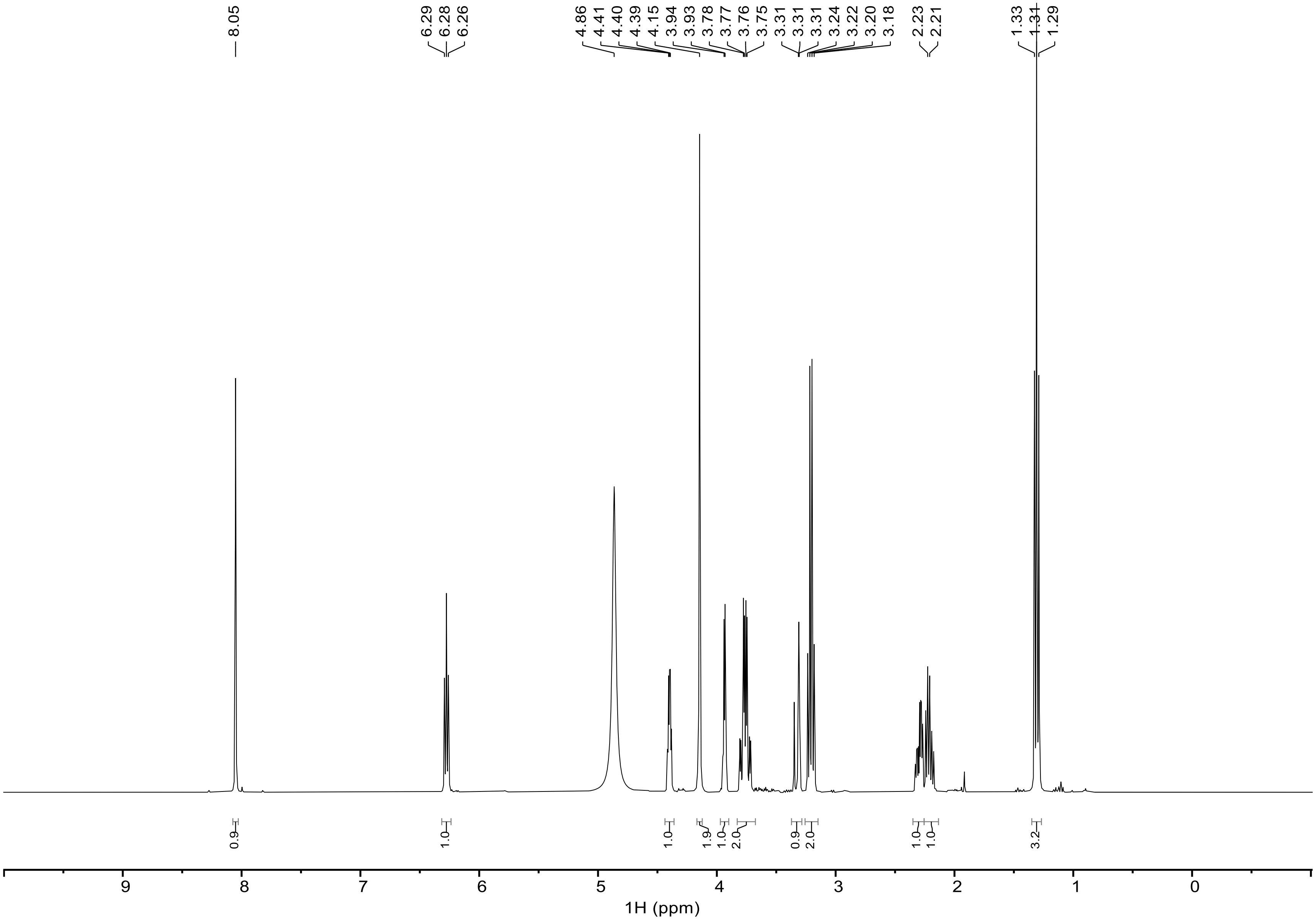

## $^{13}C$ APT NMR (100.8 MHz, $CD_3OD$), compound dU$^{tfa}$

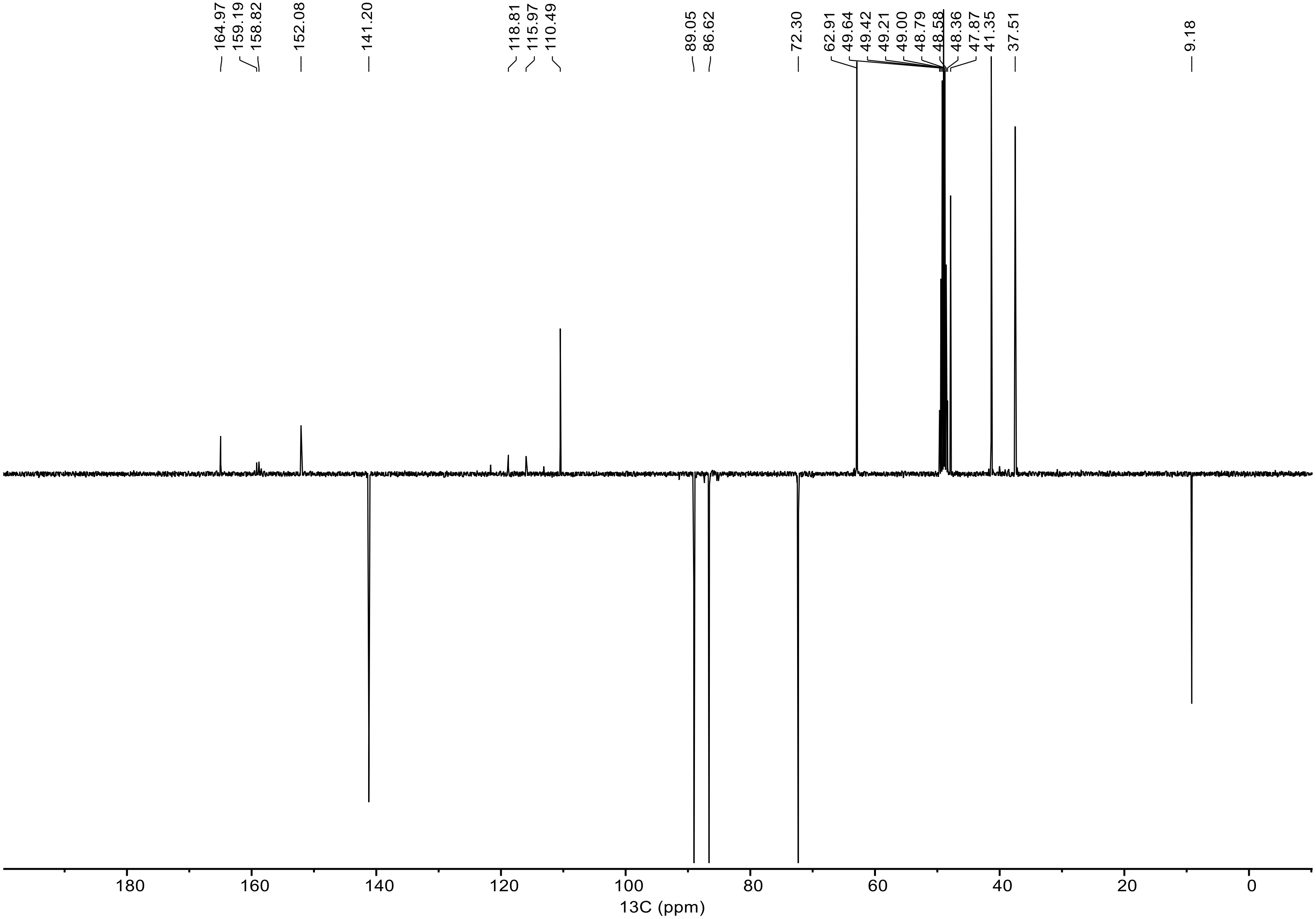

**$^{1}$H NMR (401.0 MHz, $D_2O$), compound dU[mm]**

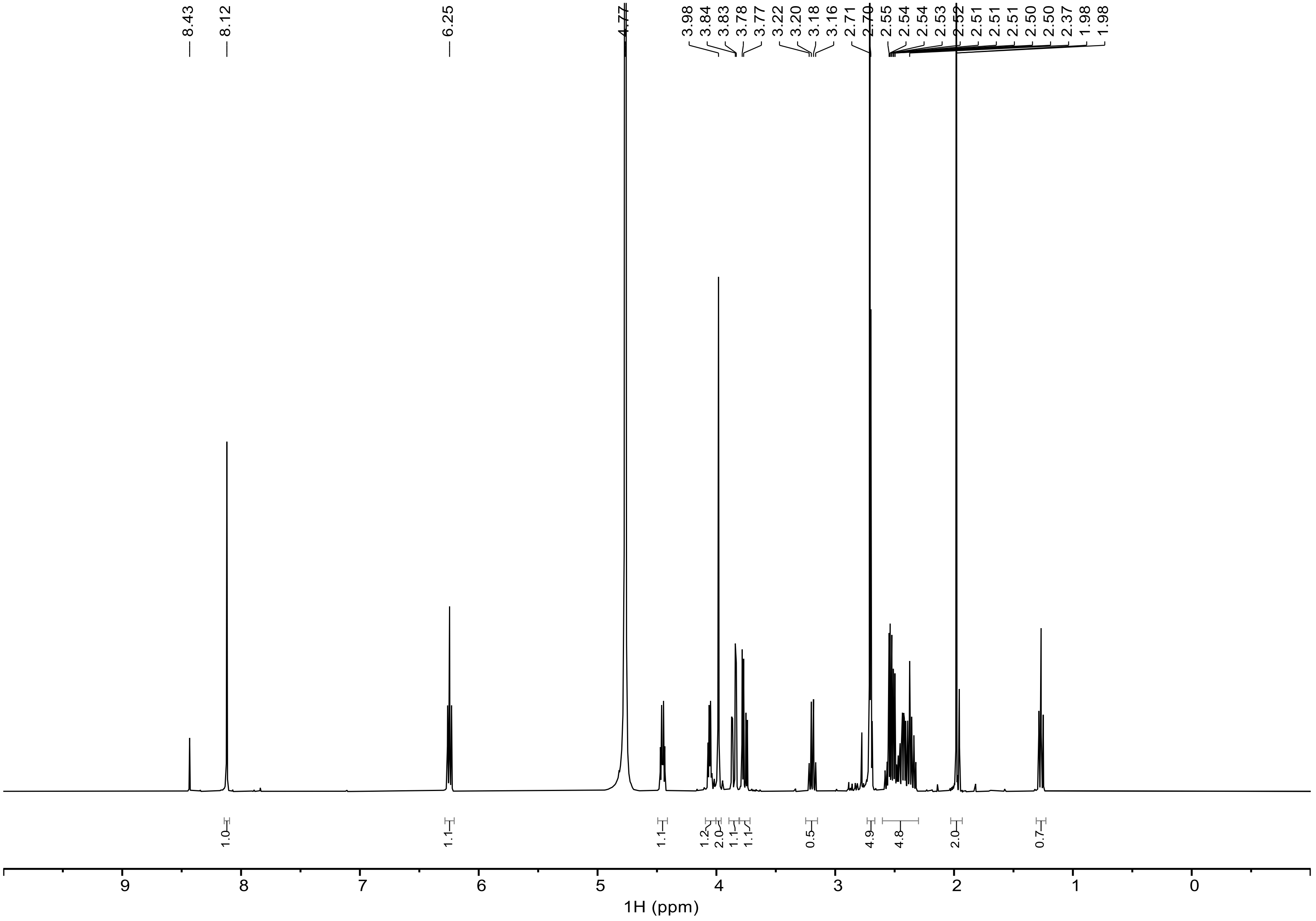

## $^{13}$C APT NMR (100.8 MHz, $D_2O$), compound dU[mm]

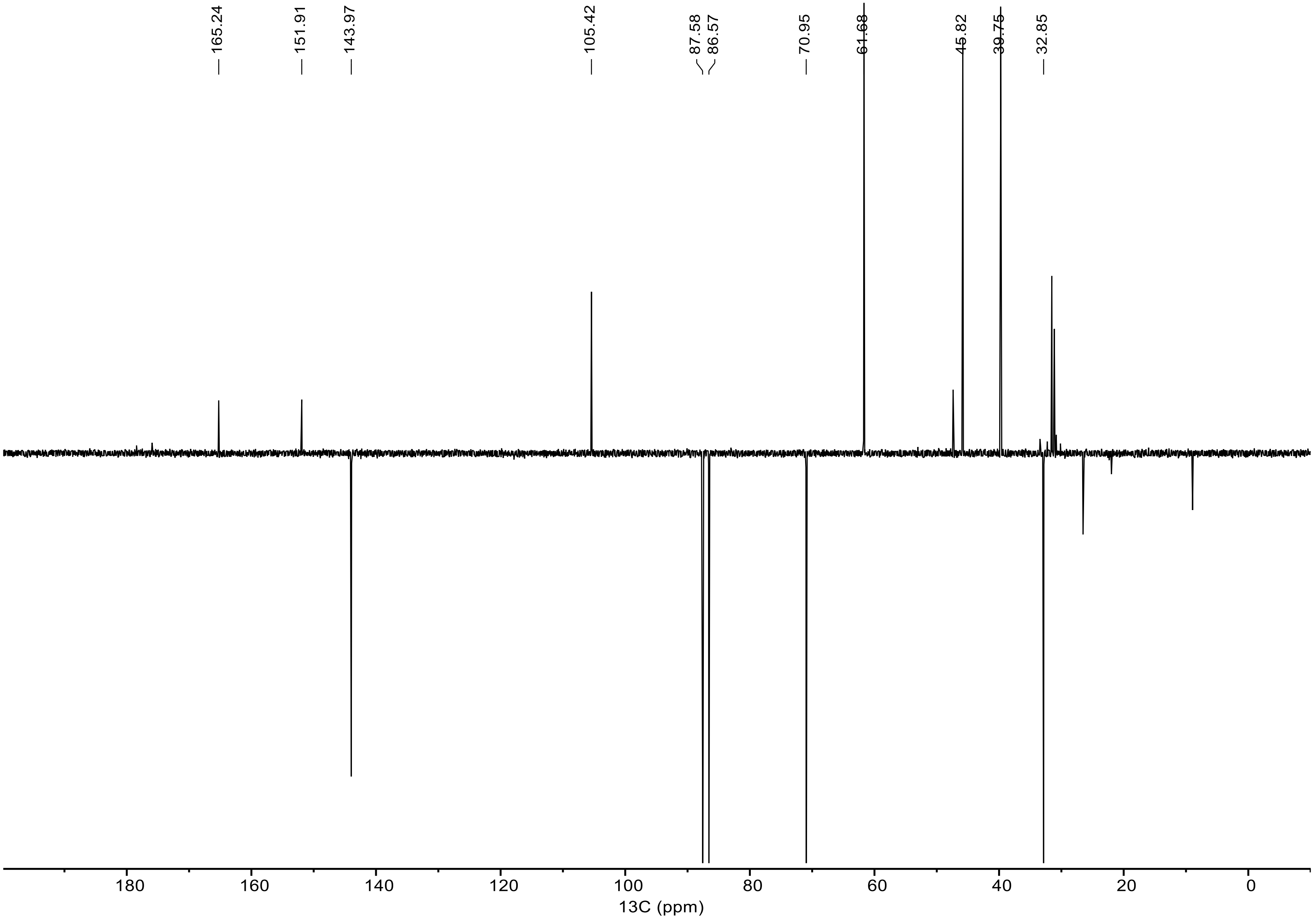

**$^{1}H$ NMR (401.0 MHz, $CD_3OD$), compound dU$^{mm\text{-}tfa}$**

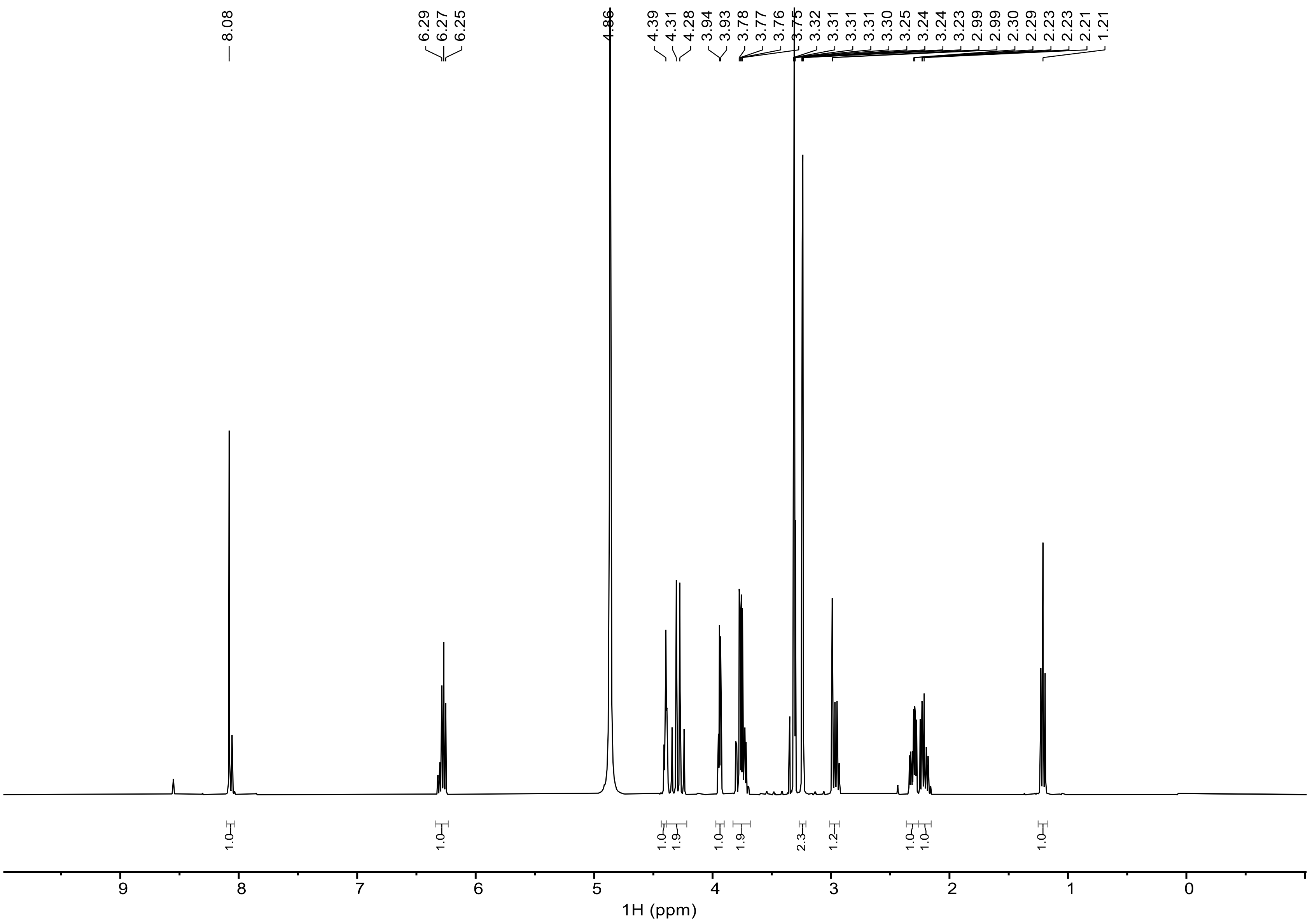

**$^{13}C$ APT NMR (100.8 MHz, $CD_3OD$), compound dU$^{mm\text{-}tfa}$**

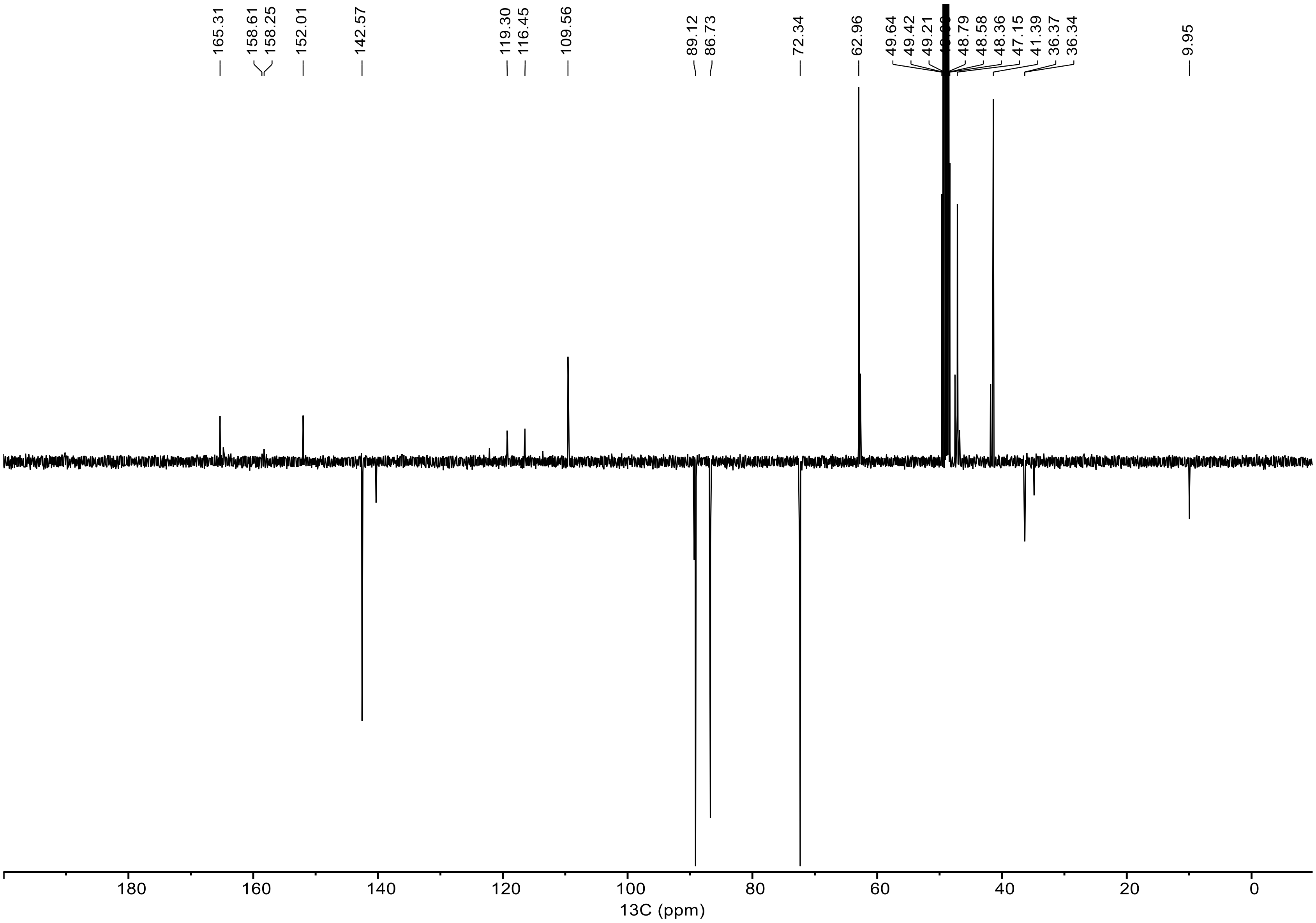

$^1H$ NMR (401.0 MHz, $D_2O$), compound dU$^{dm}$

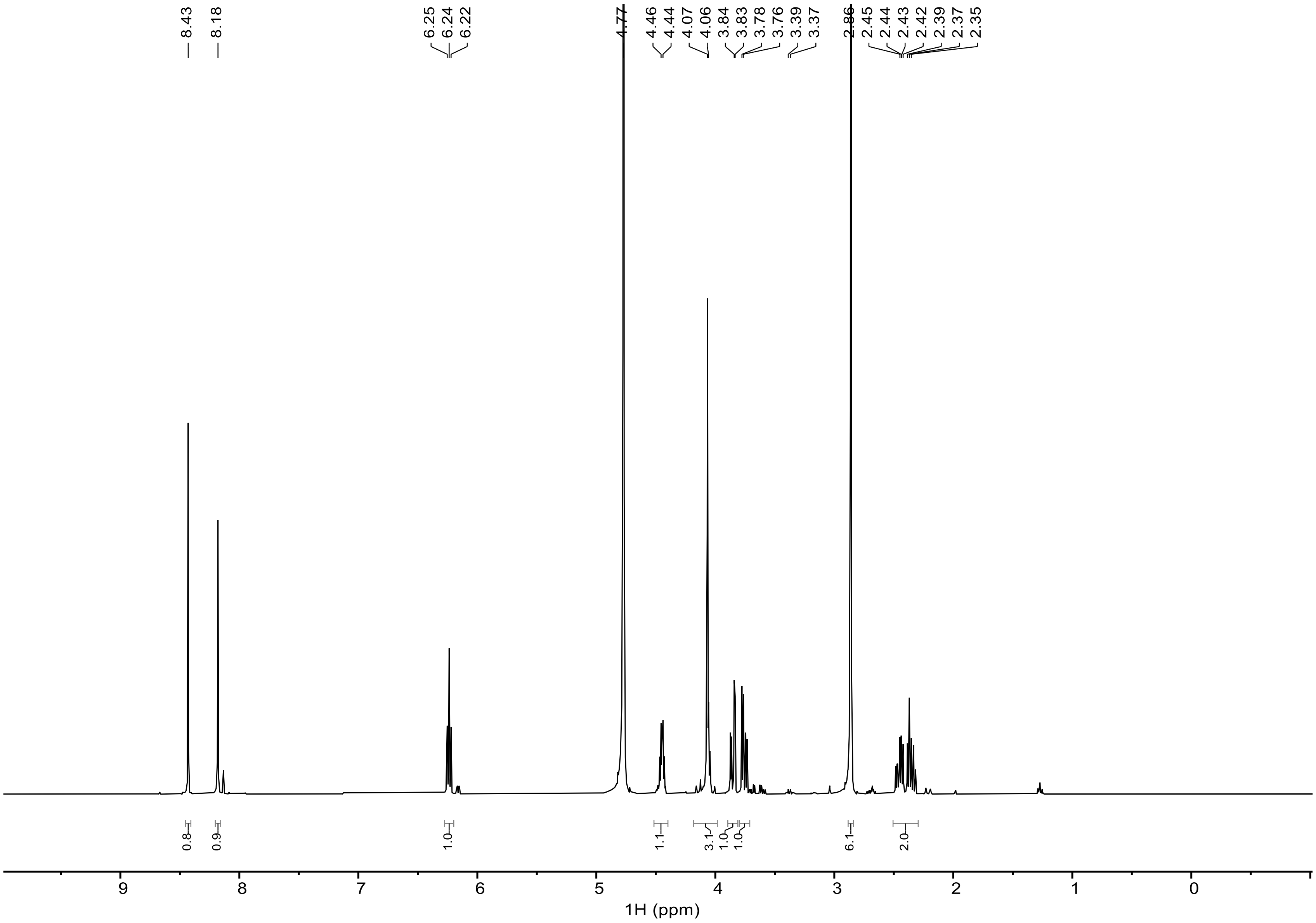

$^{13}C$ APT NMR (100.8 MHz, $D_2O$), compound dU$^{dm}$

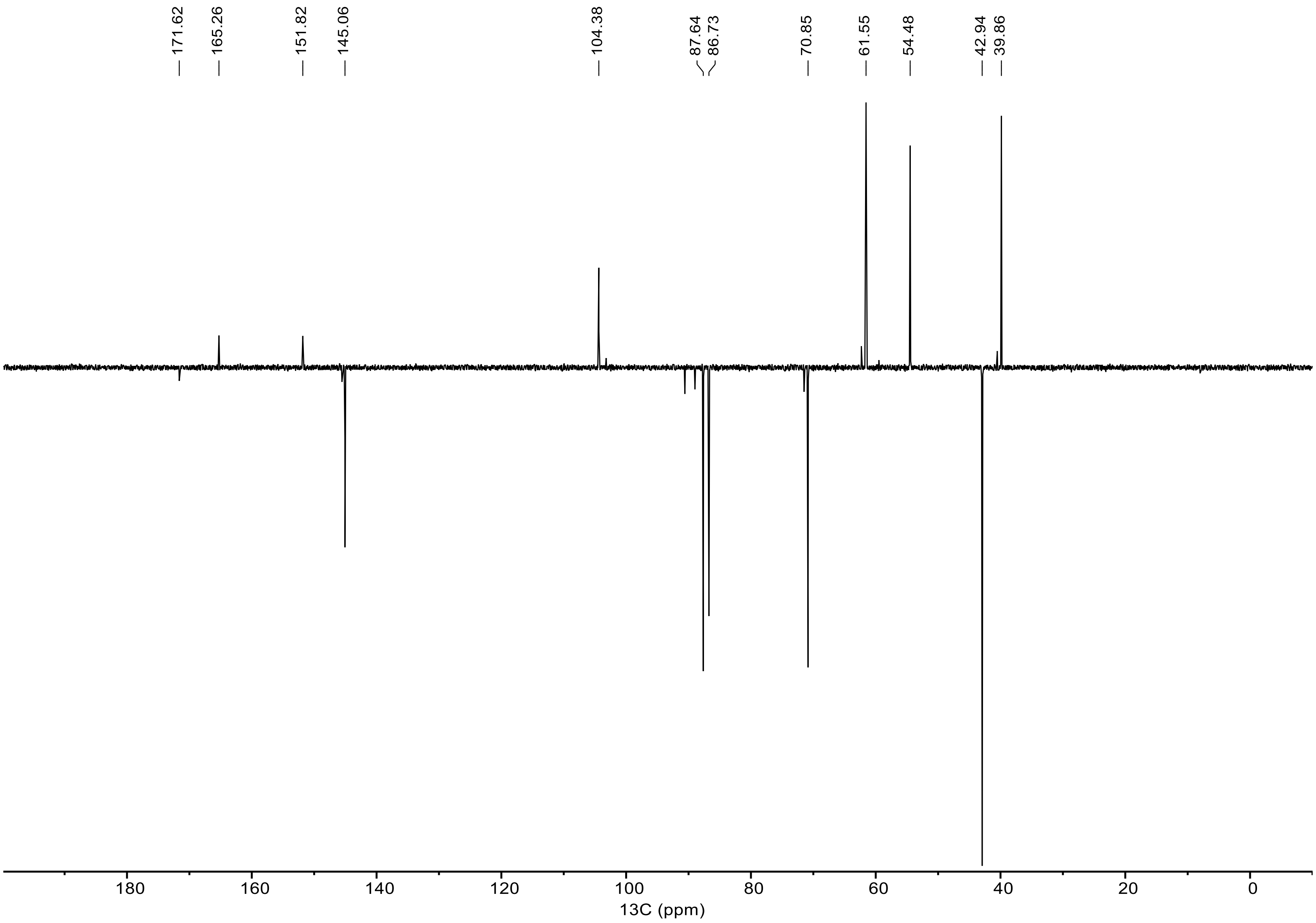

**$^{1}H$ NMR (401.0 MHz, $D_2O$), compound dU$^{cm}$**

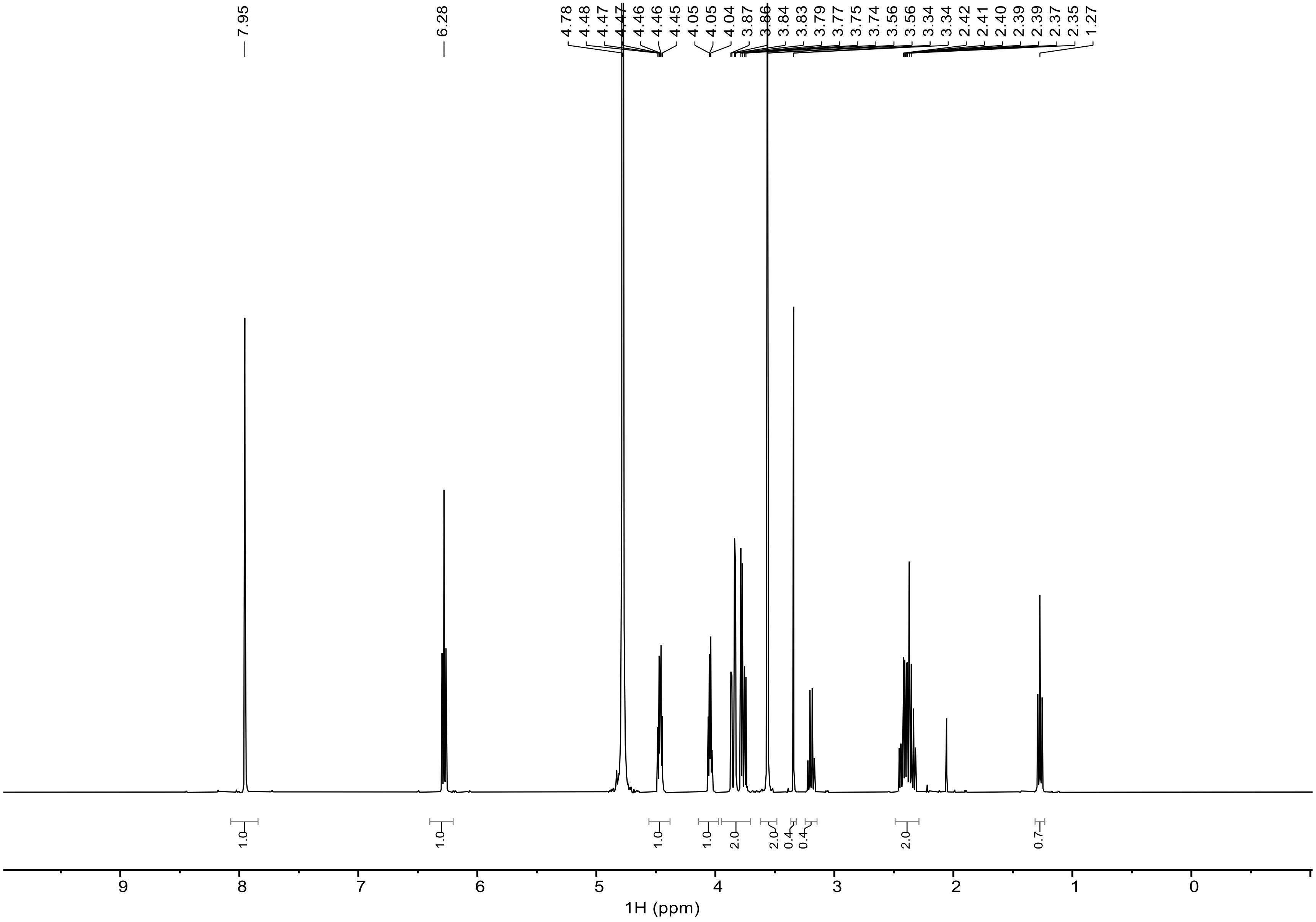

## $^{13}C$ NMR (100.8 MHz, $D_2O$), compound dU$^{cm}$

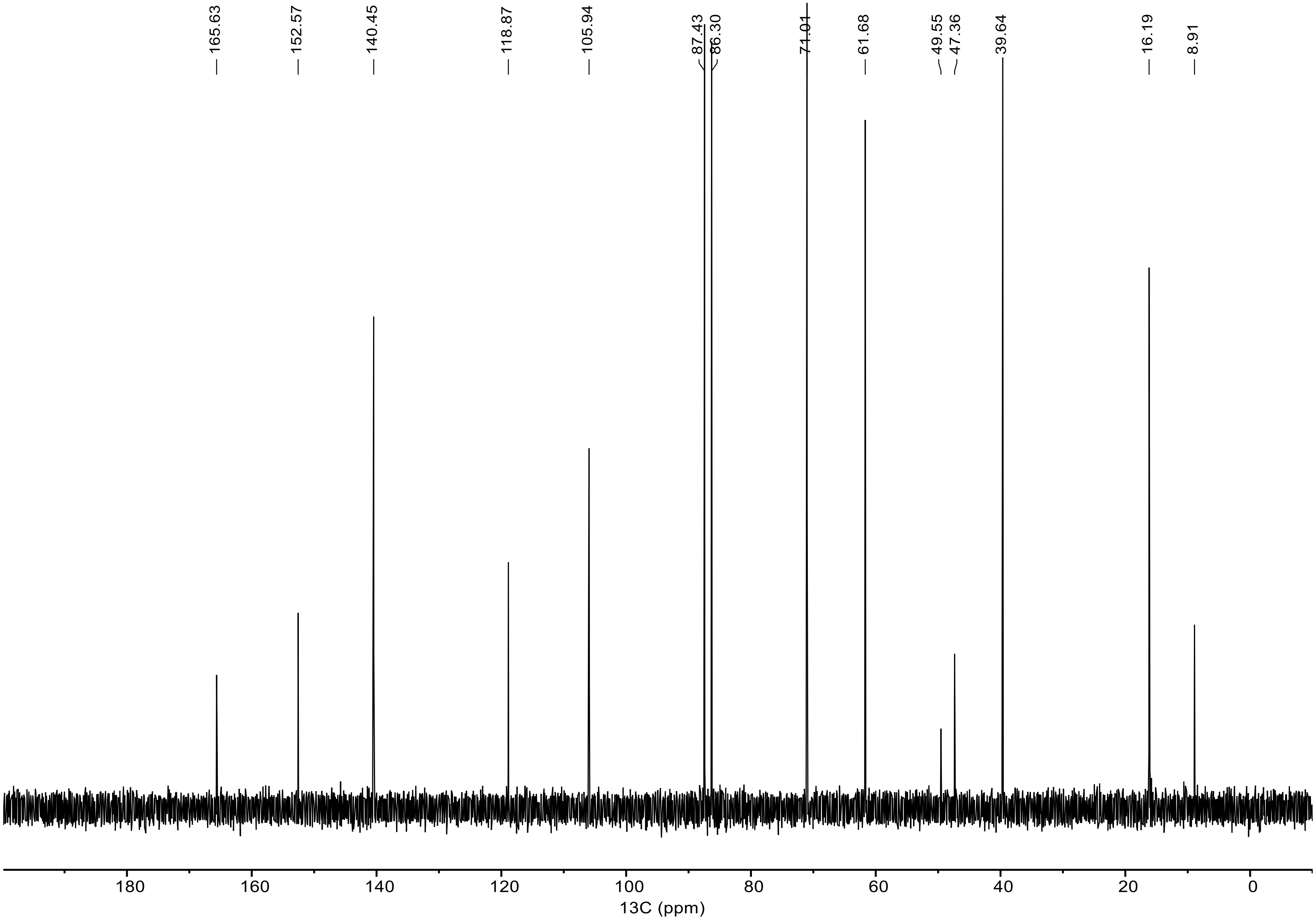

### $^{1}H$ NMR (401.0 MHz, $D_2O$), compound dU$^{asm}$

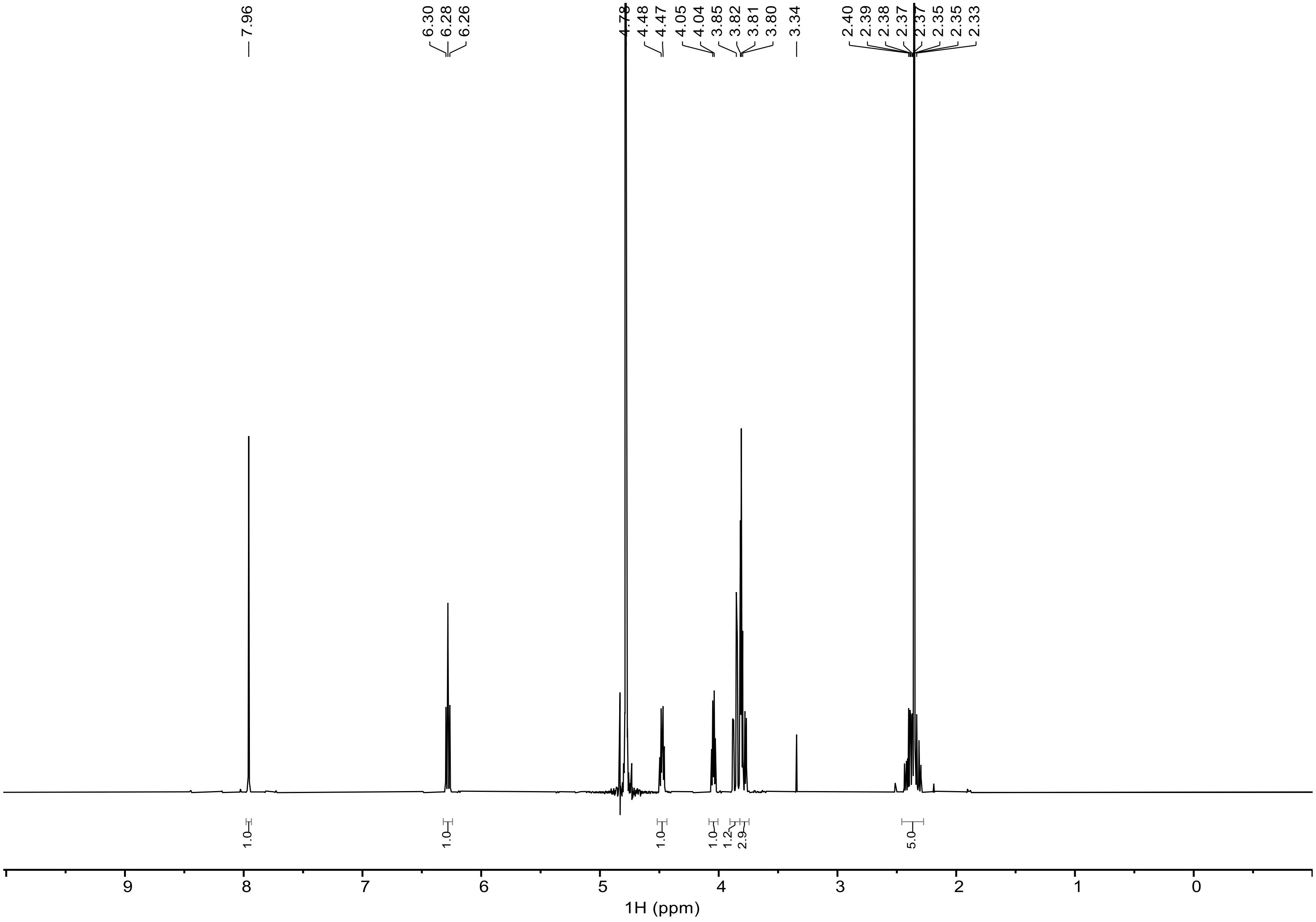

**$^{13}C$ APT NMR (100.8 MHz, $D_2O$), compound dU$^{asm}$**

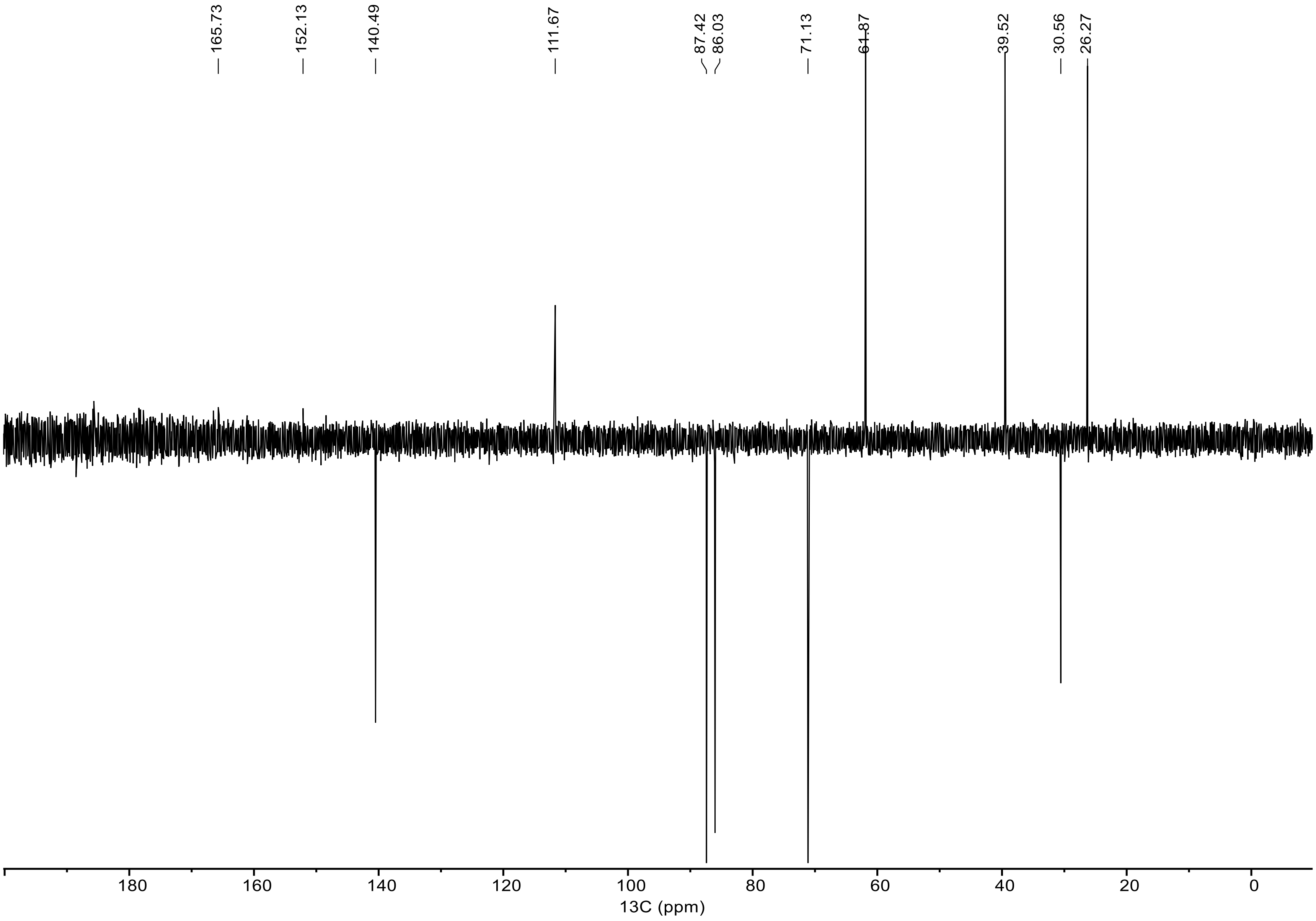

**$^{1}$H NMR (401.0 MHz, $CDCl_3$), compound 4**

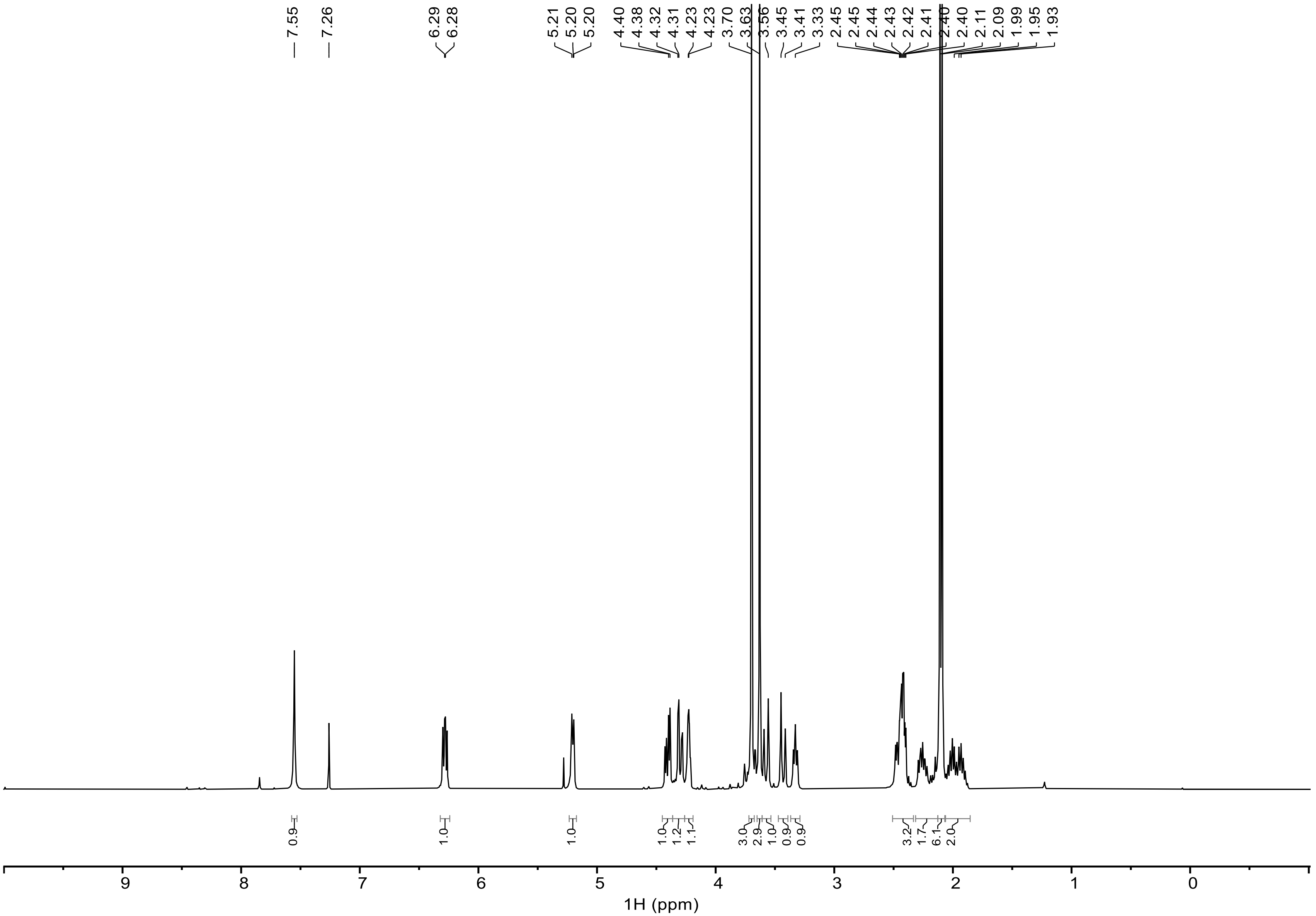

**$^{13}C$ APT NMR (100.8 MHz, $CDCl_3$), compound 4**

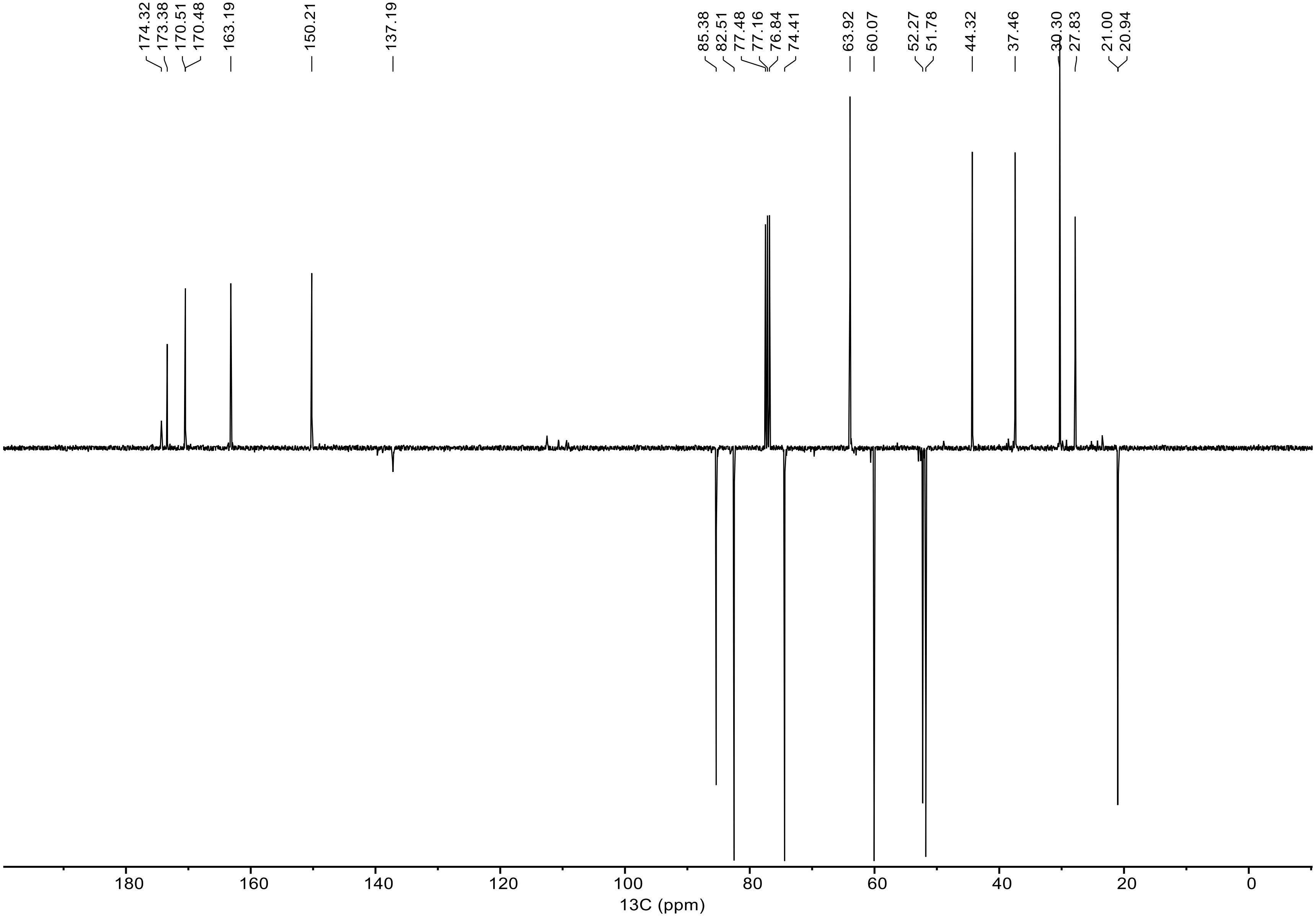

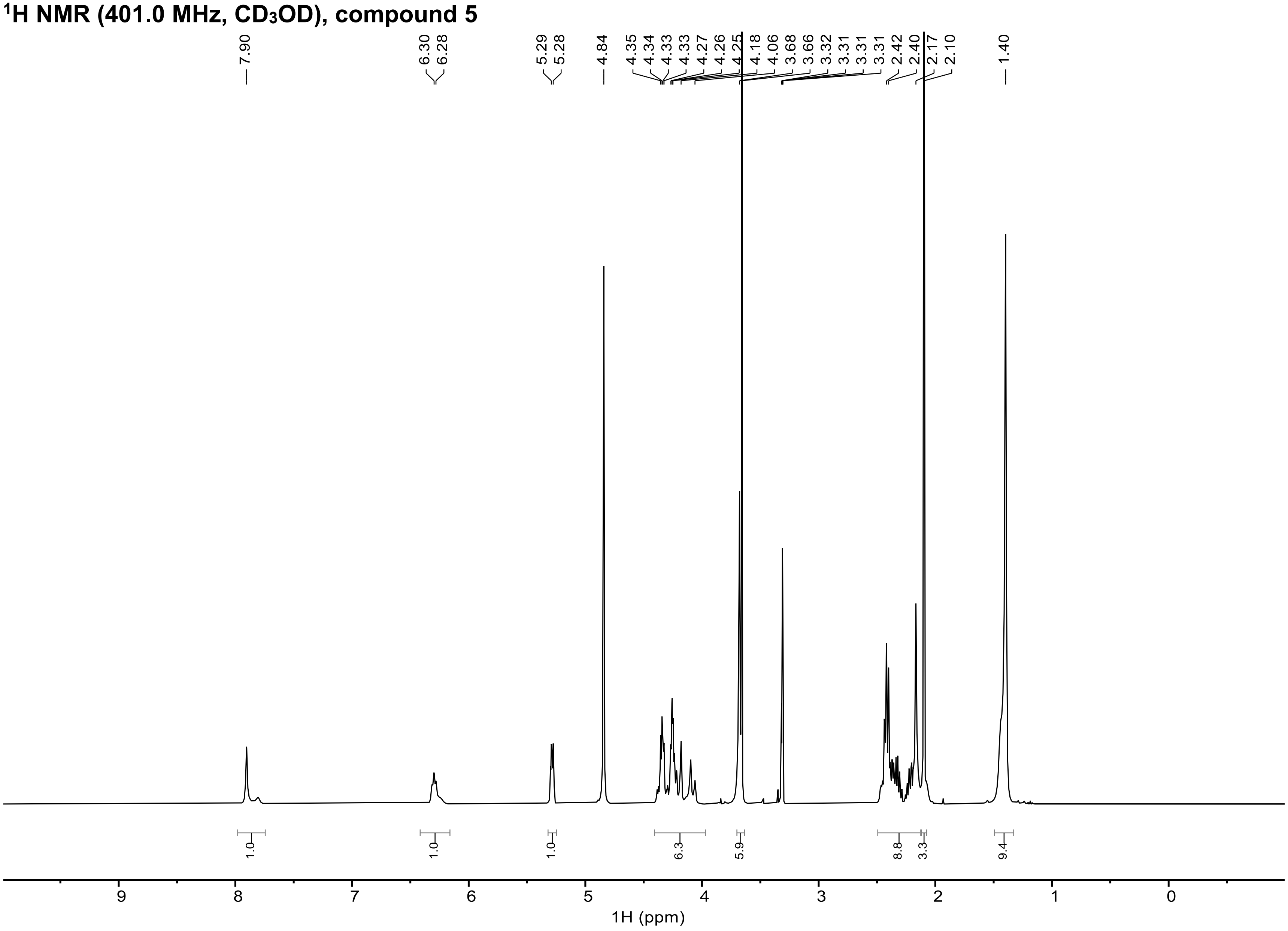

$^1$H NMR (401.0 MHz, CD$_3$OD), compound 5
7.90
6.30
6.28
5.29
5.28
4.84
4.35
4.34
4.33
4.33
4.27
4.26
4.25
4.18
4.06
3.68
3.66
3.32
3.31
3.31
3.31
2.42
2.40
2.17
2.10
1.40
1.0
1.0
1.0
6.3
5.9
8.8
3.3
9.4
9
8
7
6
5
4
3
2
1
0
1H (ppm)

**$^{13}$C APT NMR (100.8 MHz, $CD_3OD$), compound 5**

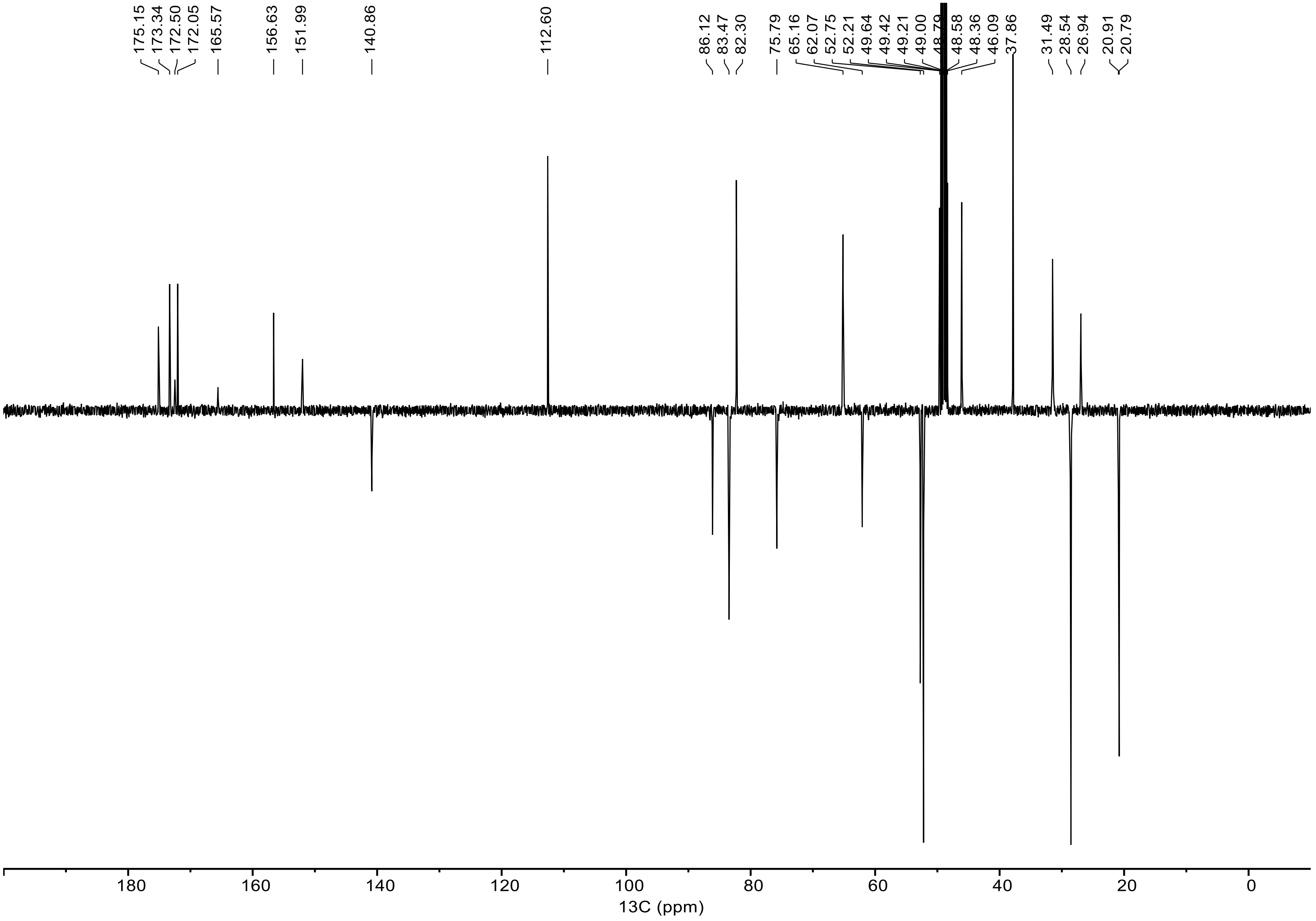

**$^{1}$H NMR (401.0 MHz, $CD_3OD$), compound 6**

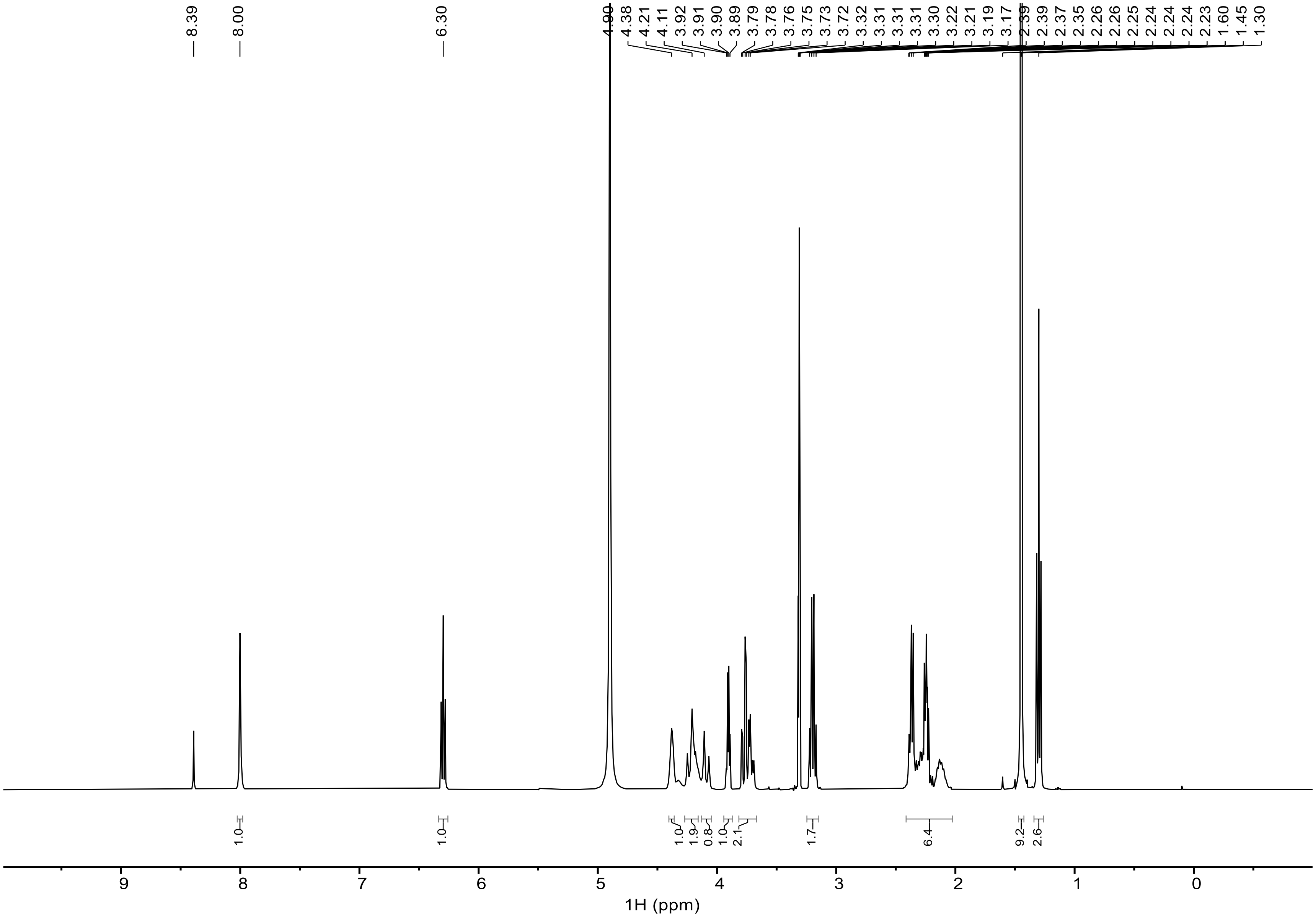

**$^{13}$C APT NMR (100.8 MHz, $CD_3OD$), compound 6**

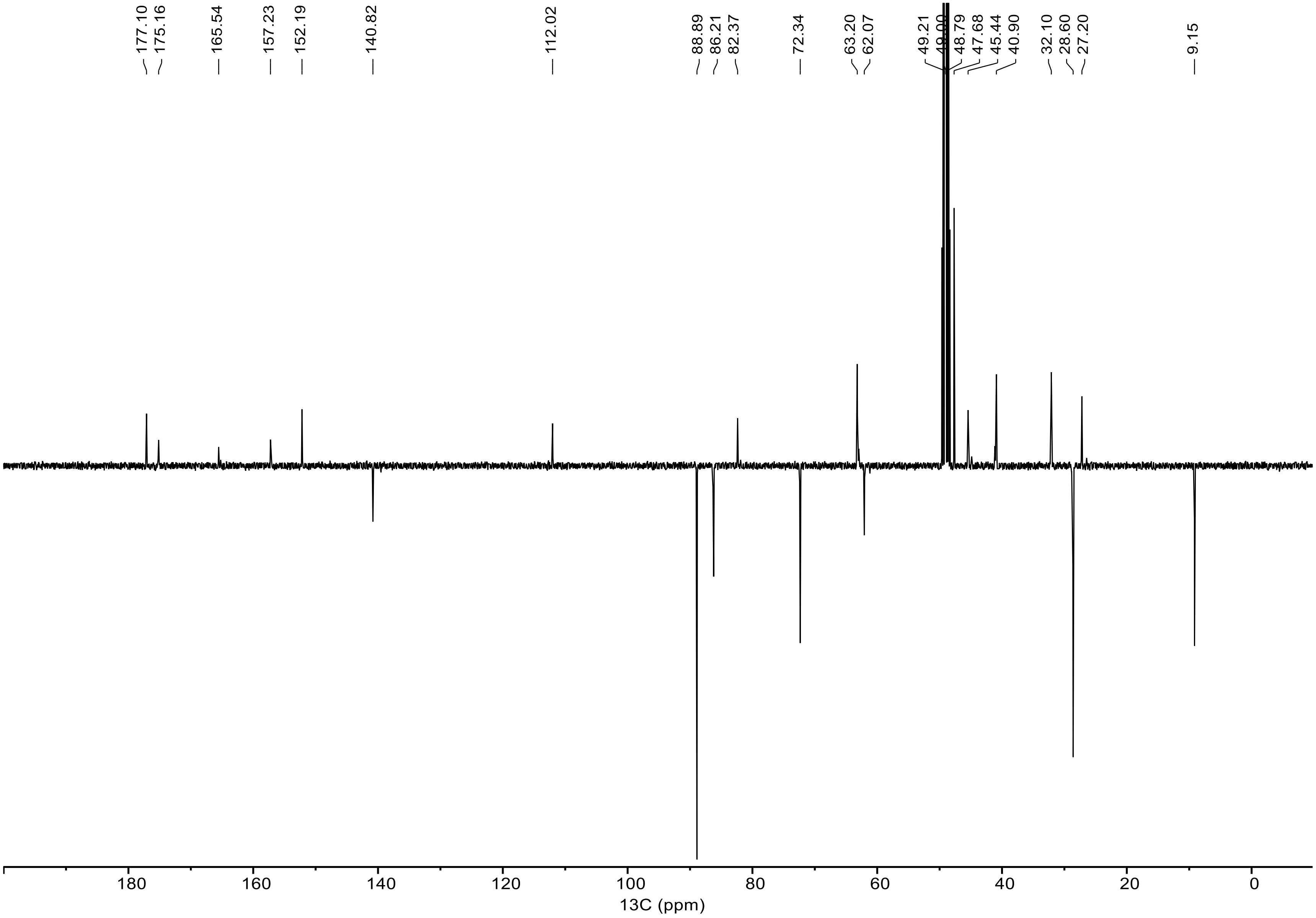

$^{1}$H NMR (401.0 MHz, $CD_3OD$), compound dU[glu]

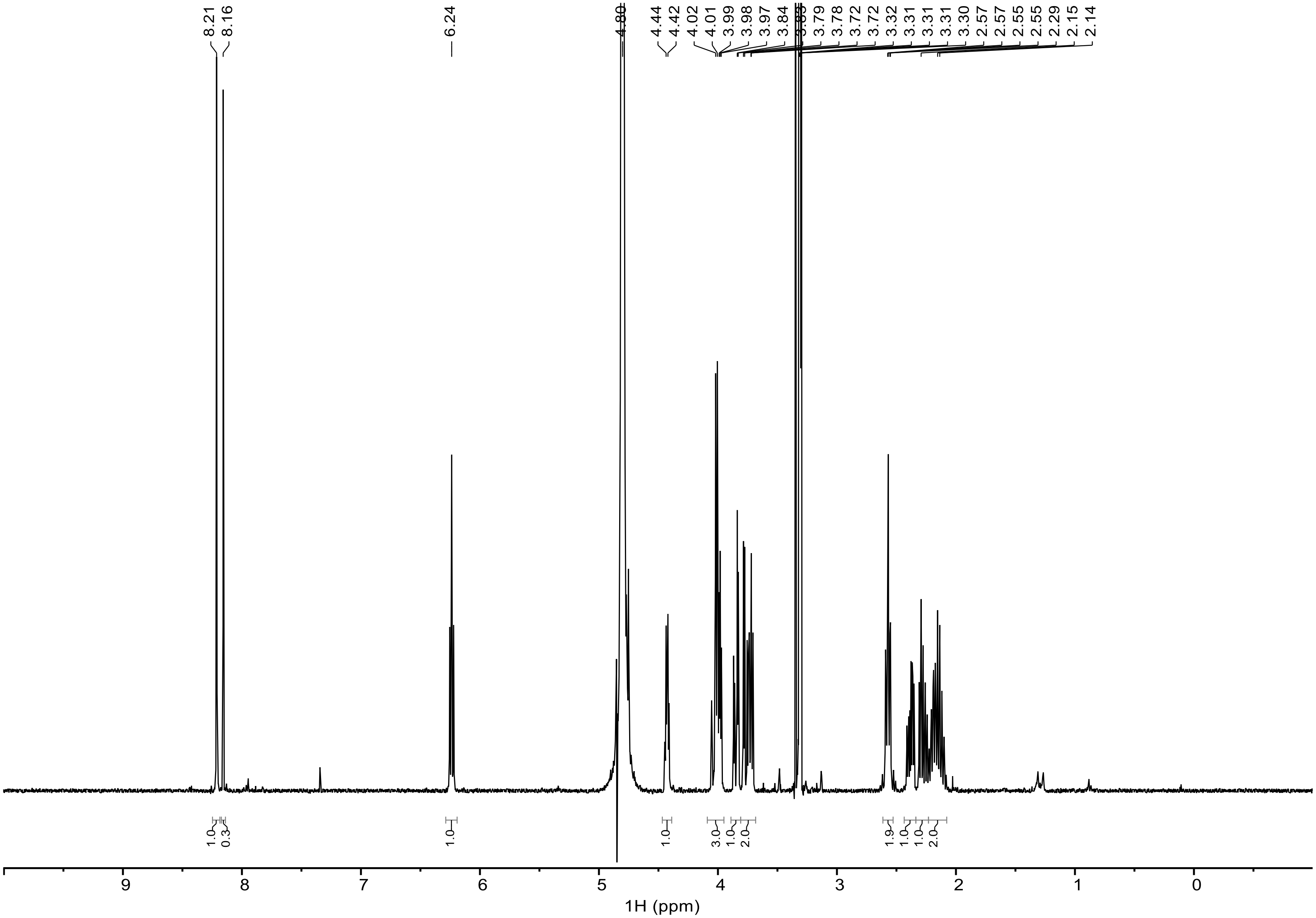

## $^{13}C$ APT NMR (100.8 MHz, $CD_3OD$), compound dU$^{glu}$

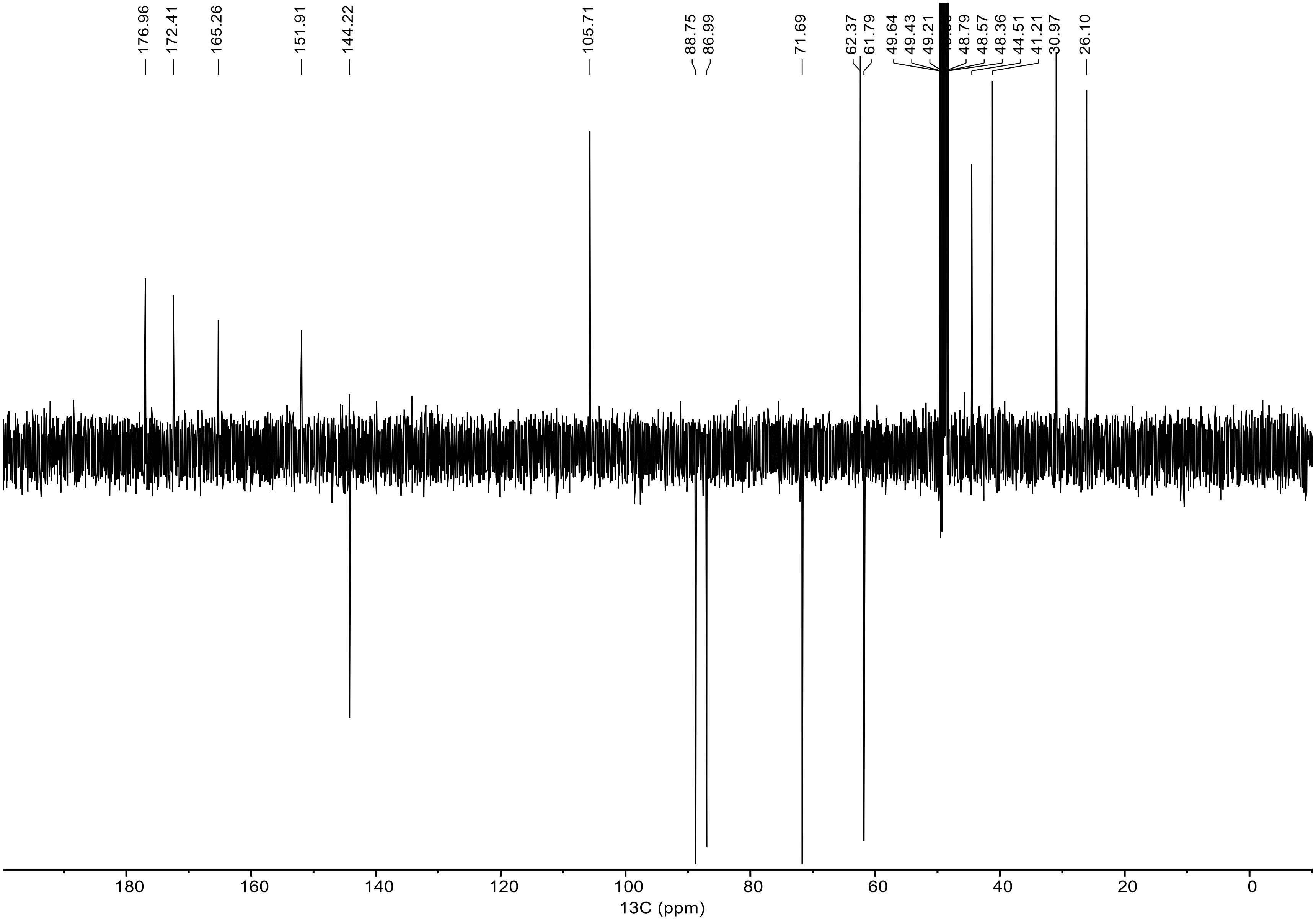

**$^{1}$H NMR (401.0 MHz, $CD_3OD$), compound 7**

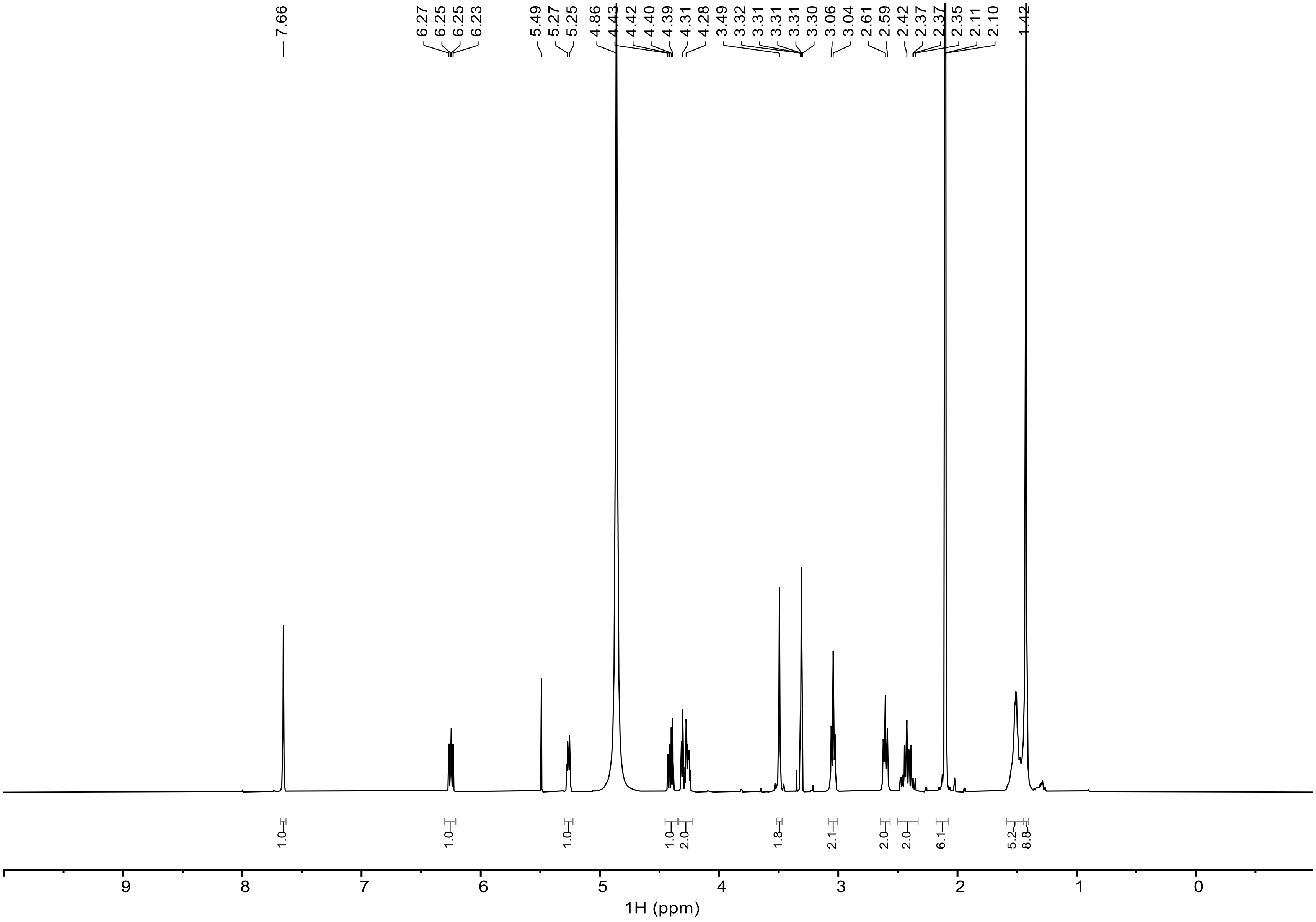

**$^{13}C$ APT NMR (100.8 MHz, $CD_3OD$), compound 7**

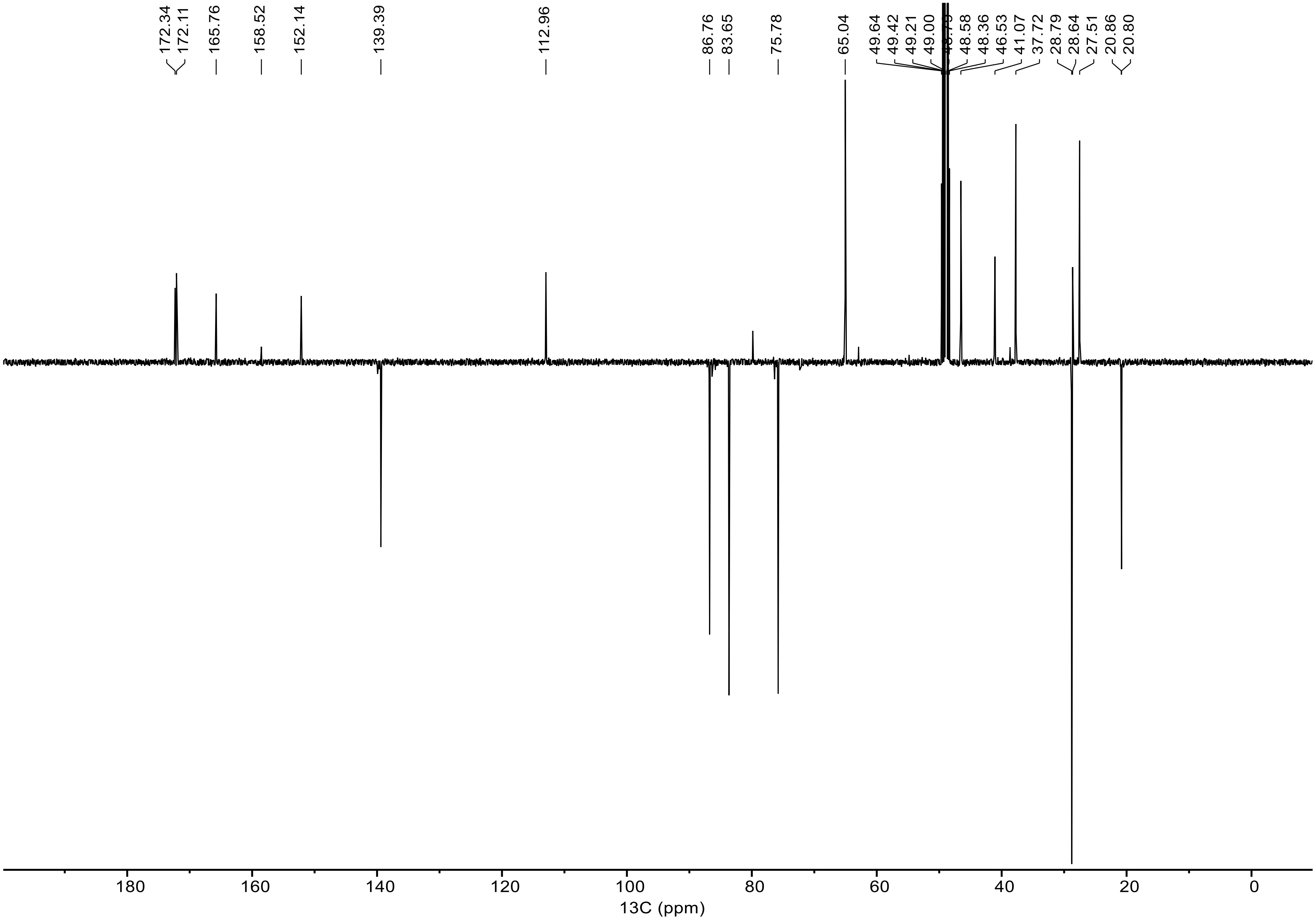

## $^1H$ NMR (401.0 MHz, $D_2O$), compound dU$^{put}$

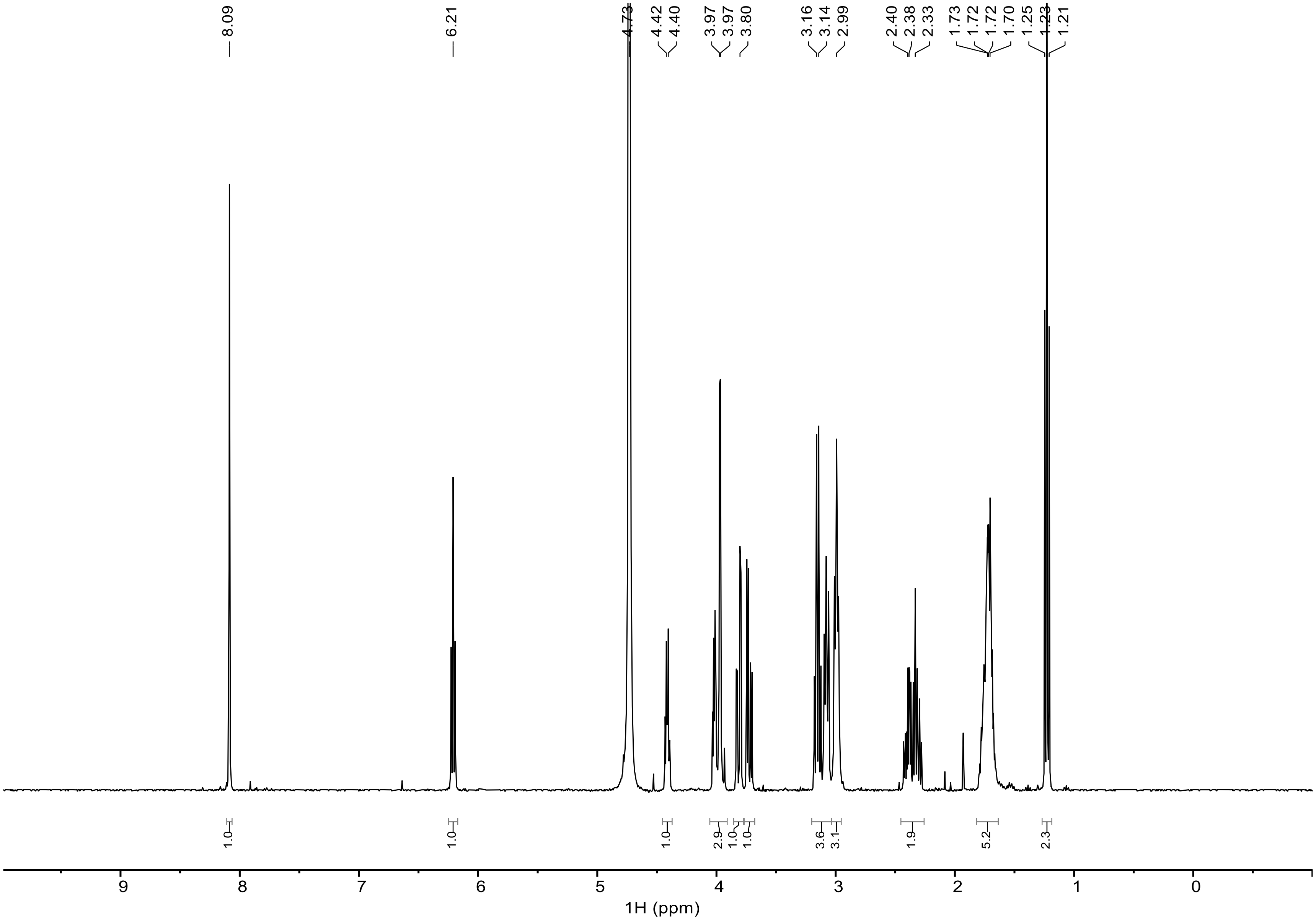

## $^{13}C$ APT NMR (100.8 MHz, $D_2O$), compound dU$^{put}$

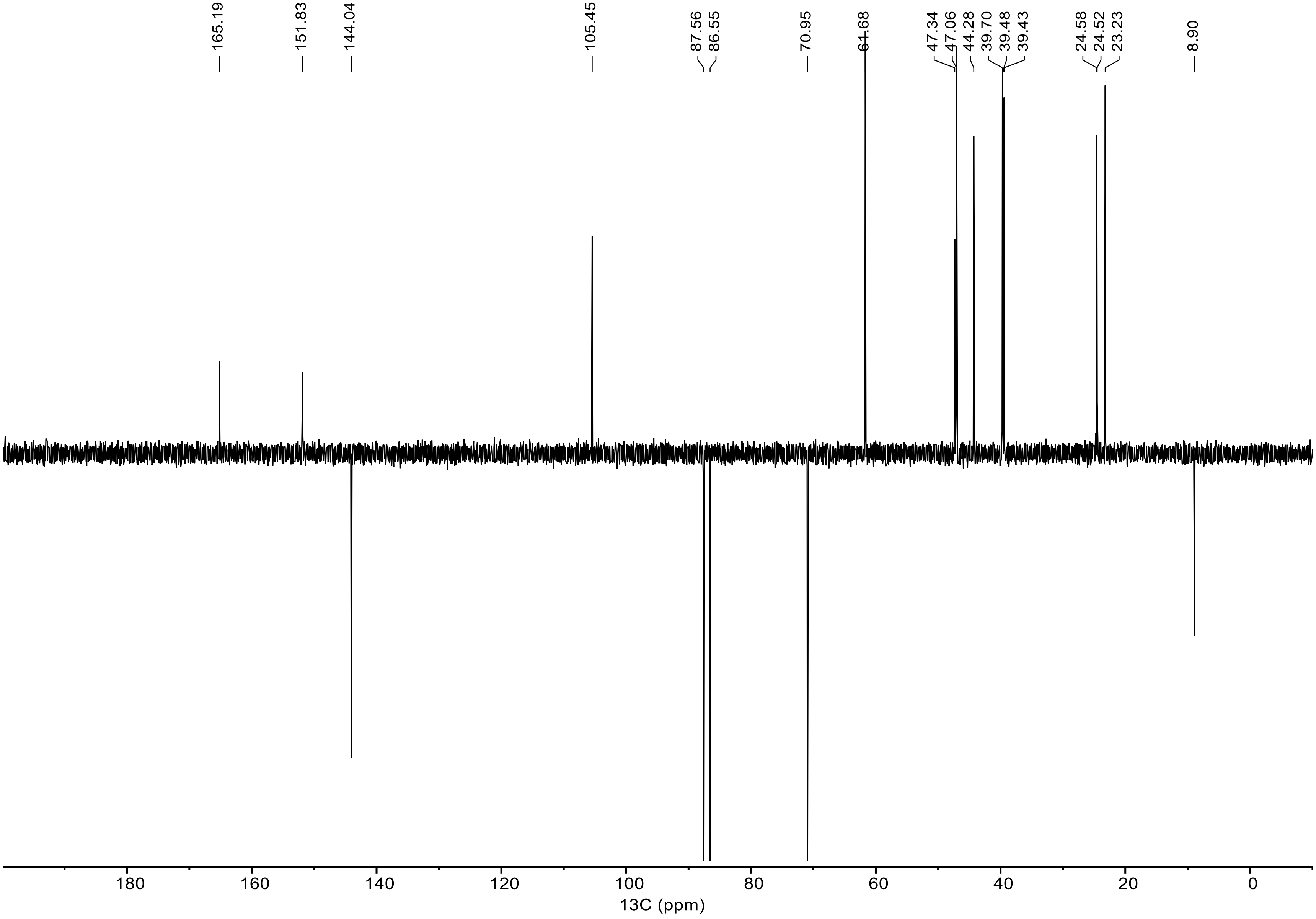

$^1H$ NMR (401.0 MHz, $CD_3OD$), compound dU[put-tfa]

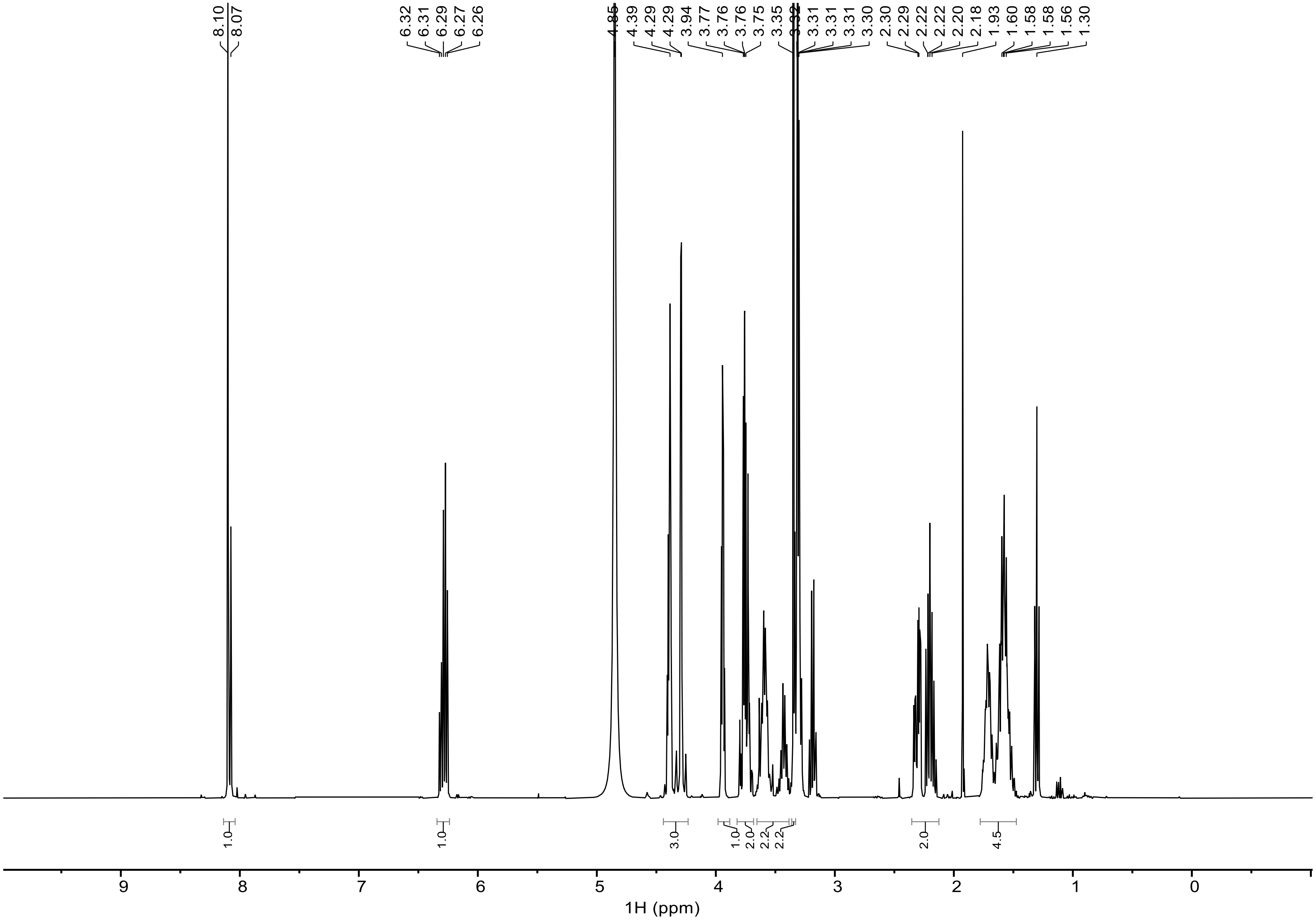

## $^{13}C$ NMR (100.8 MHz, $CD_3OD$), compound dU$^{put\text{-}tfa}$

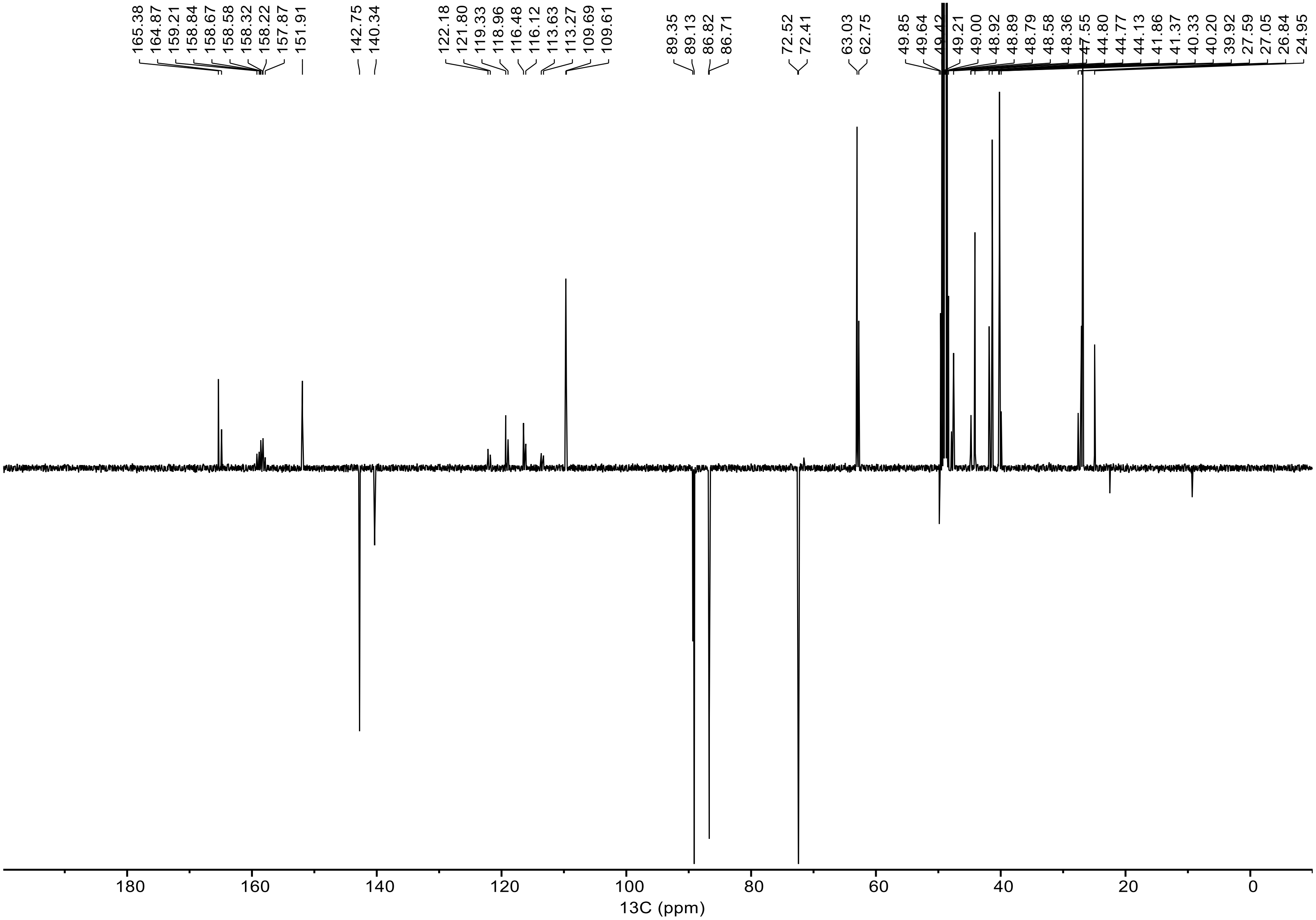

**$^{1}H$ NMR (500.0 MHz, $CD_3OD$), compound $dU^{dhp}TP$**

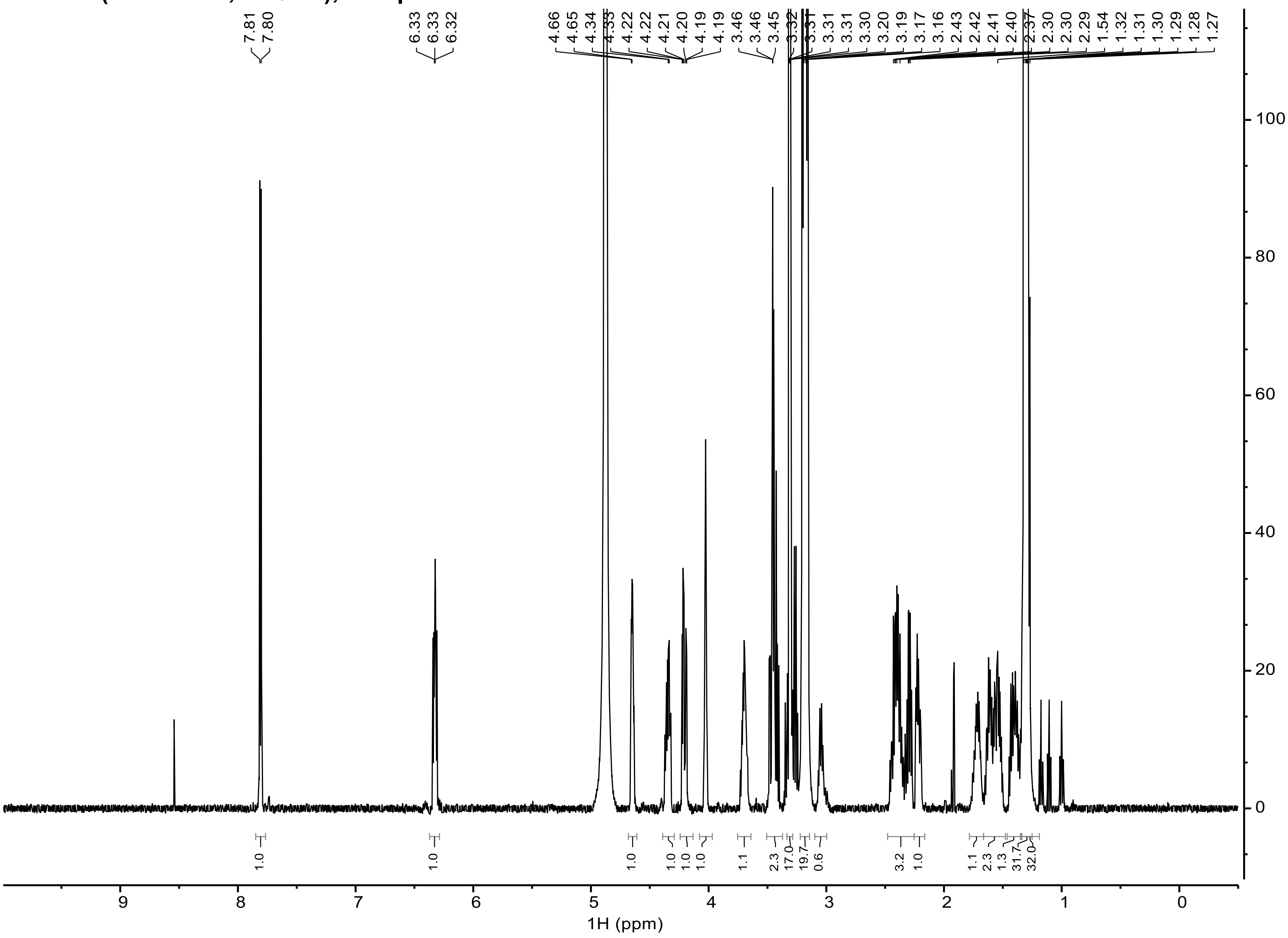

**$^{13}$C APT NMR (125.7 MHz, $CD_3OD$), compound dU$^{dhp}$TP**

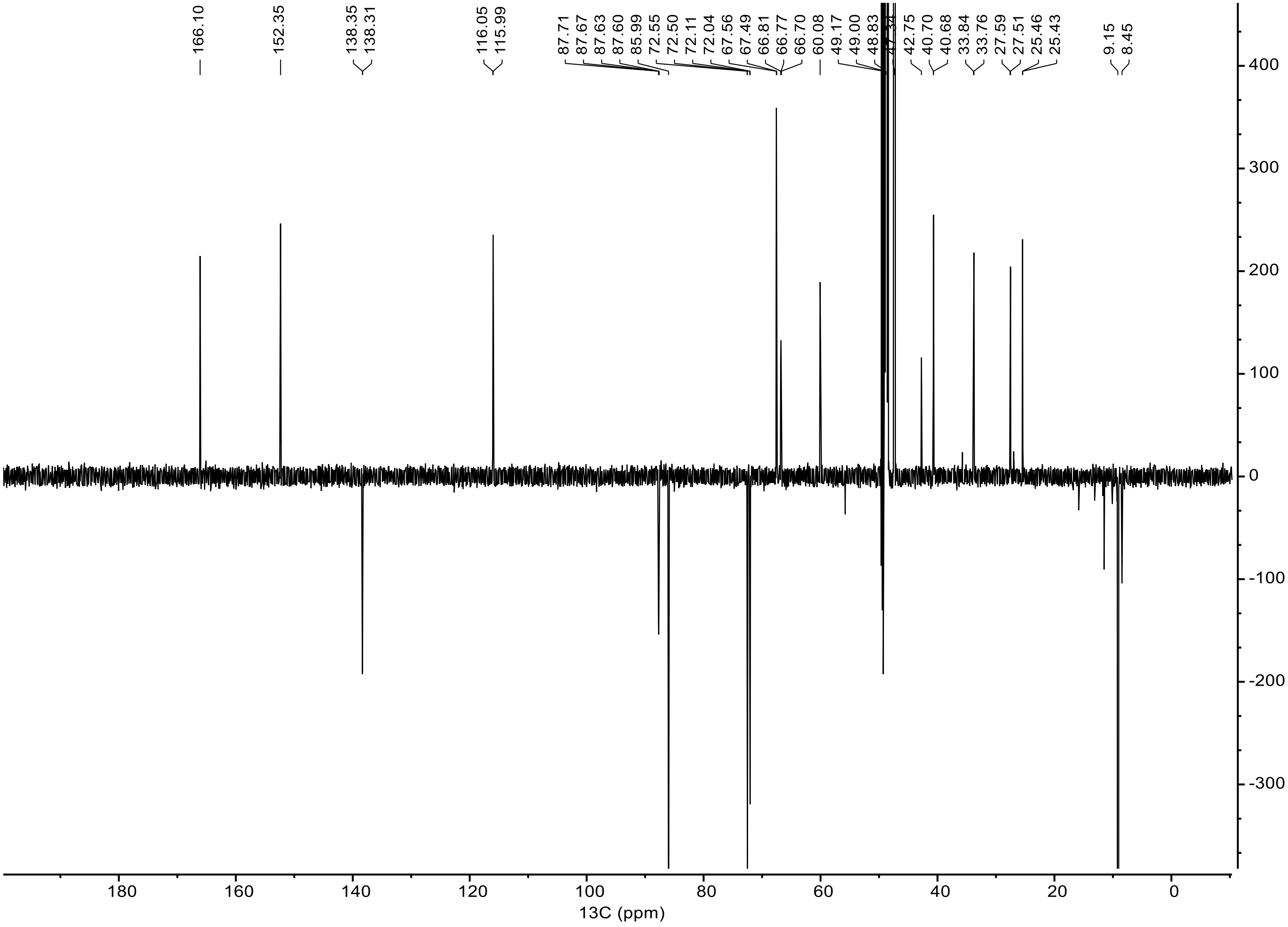

$^{31}P\{^1H\}$ NMR (202.4 MHz, $CD_3OD$), compound dU$^{dhp}$TP

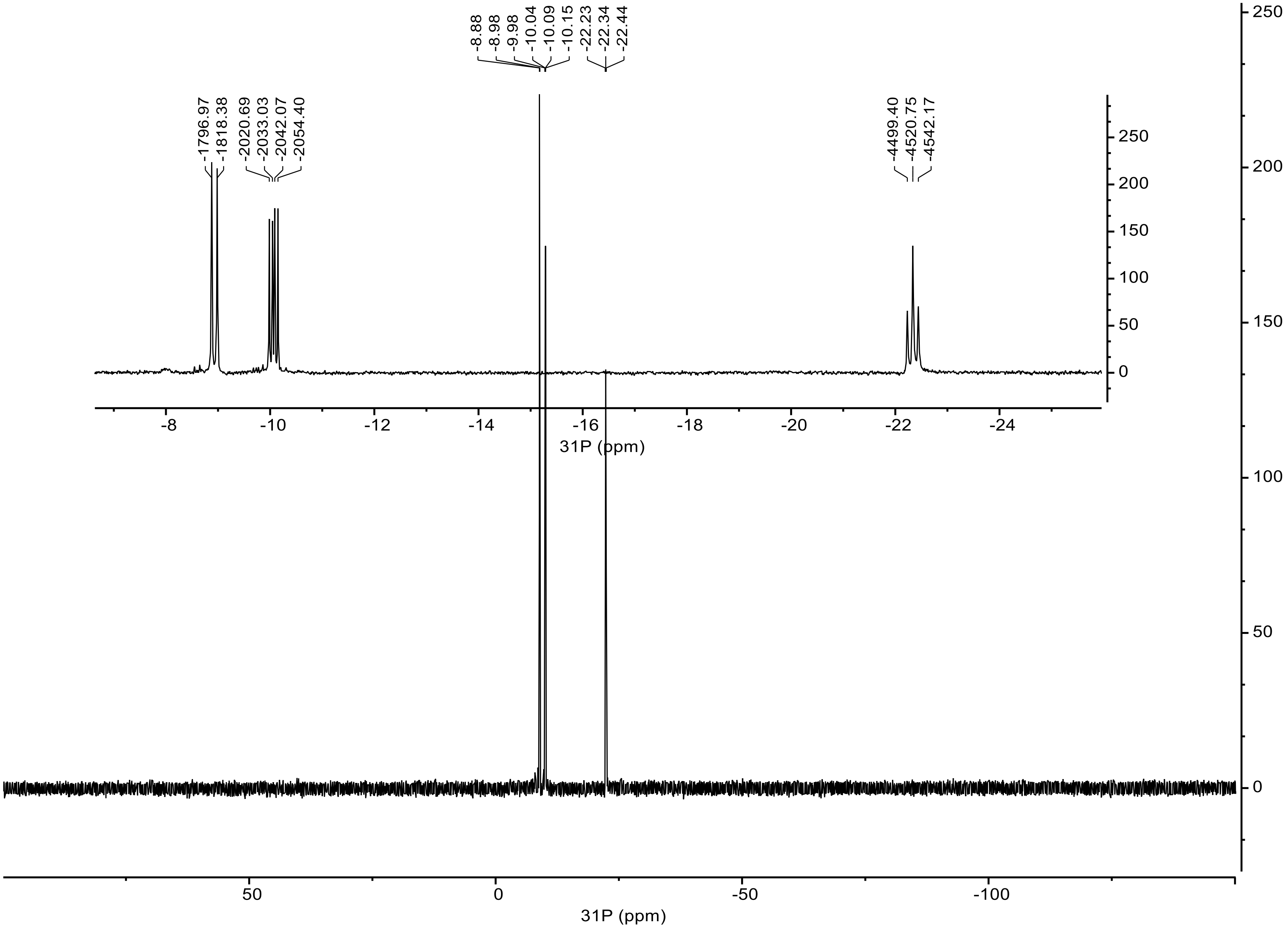

**$^{1}H$ NMR (500.0 MHz, $D_2O$), compound dU$^{glu}$TP**

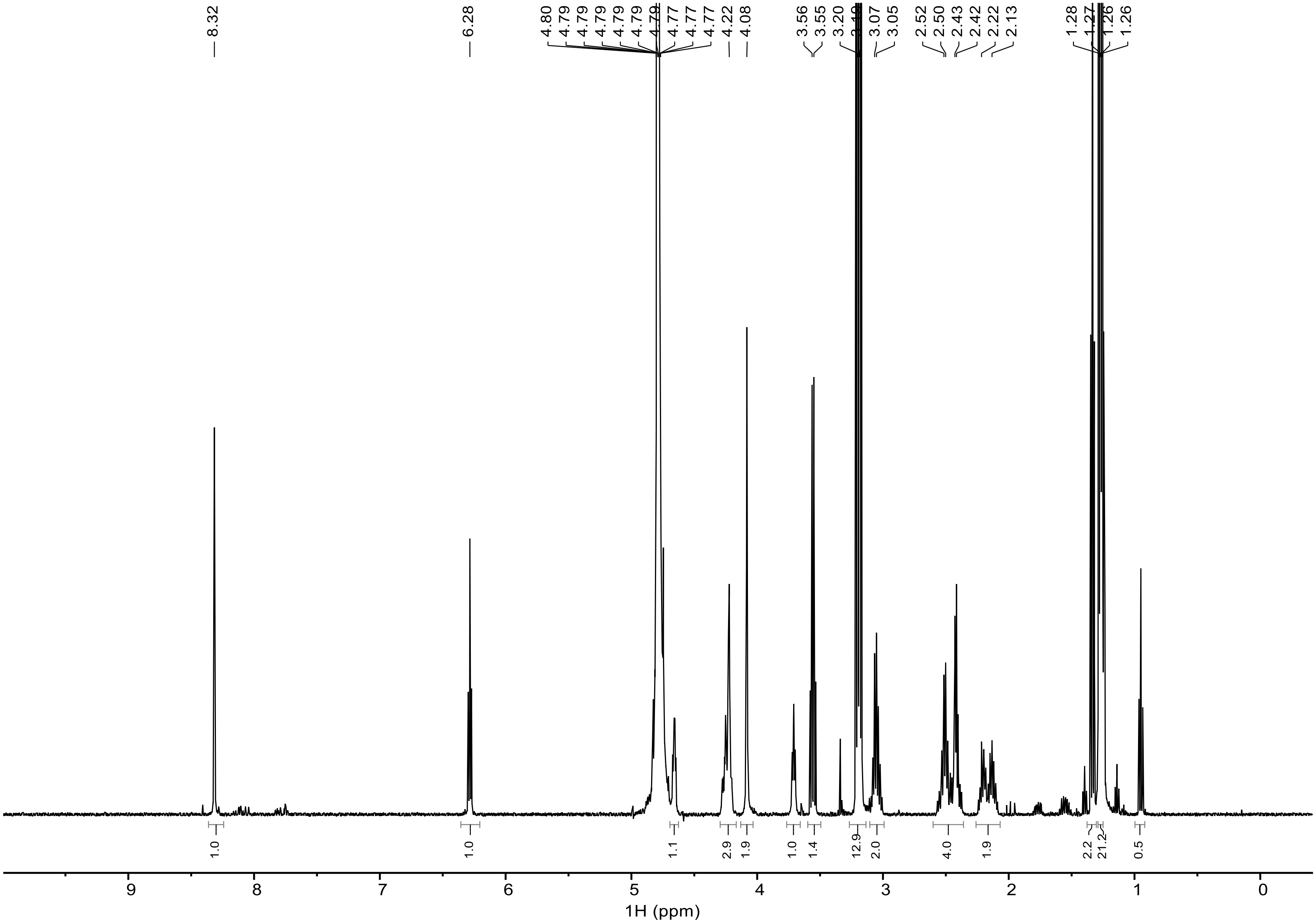

## $^{13}C$ APT NMR (125.7 MHz, $D_2O$), compound dU$^{glu}$TP

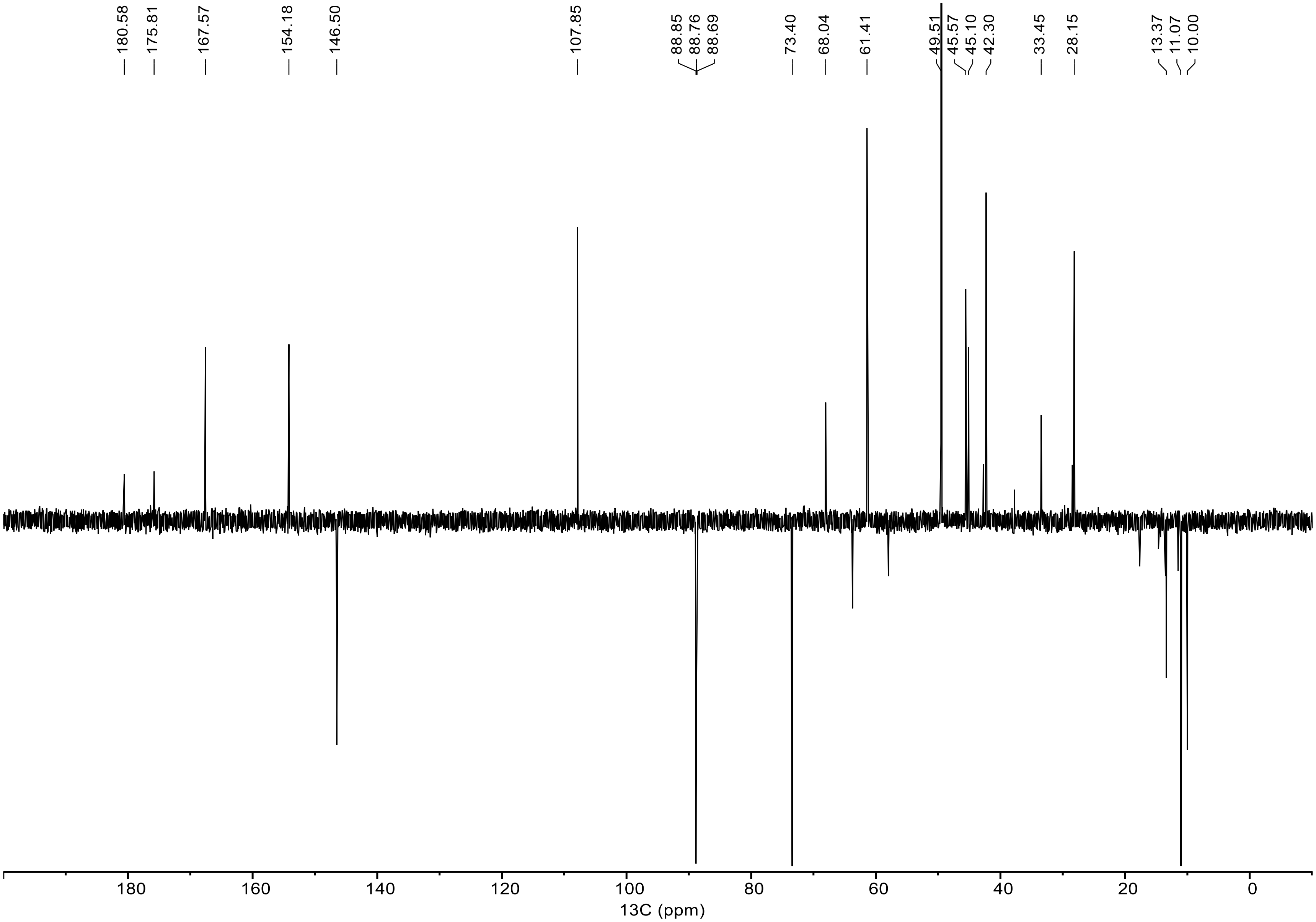

## $^{31}P\{^1H\}$ NMR (202.4 MHz, $D_2O$), compound dU$^{glu}$TP

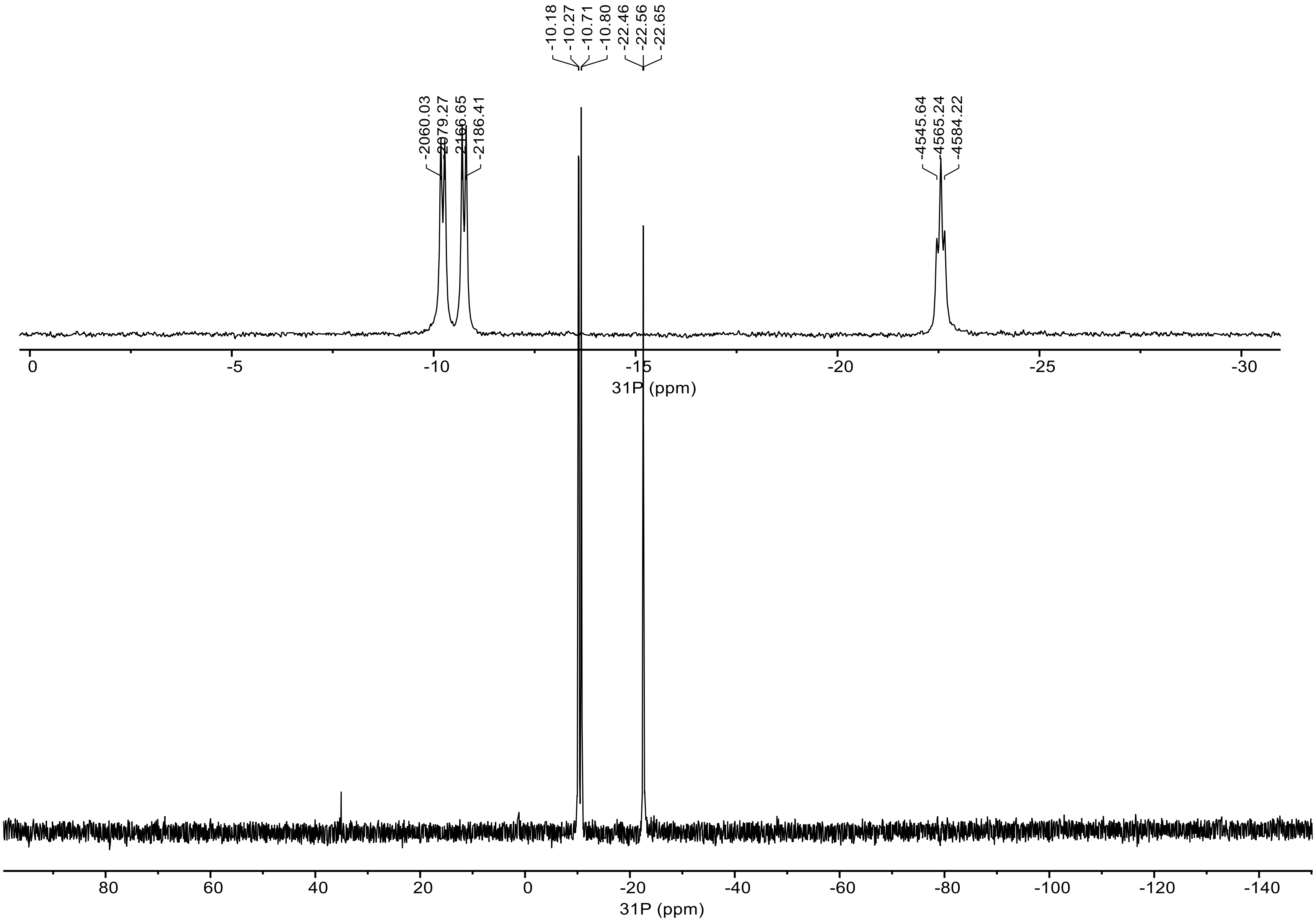

**$^1$H NMR (500.0 MHz, $CD_3OD$), compound dU$^{put}$TP**

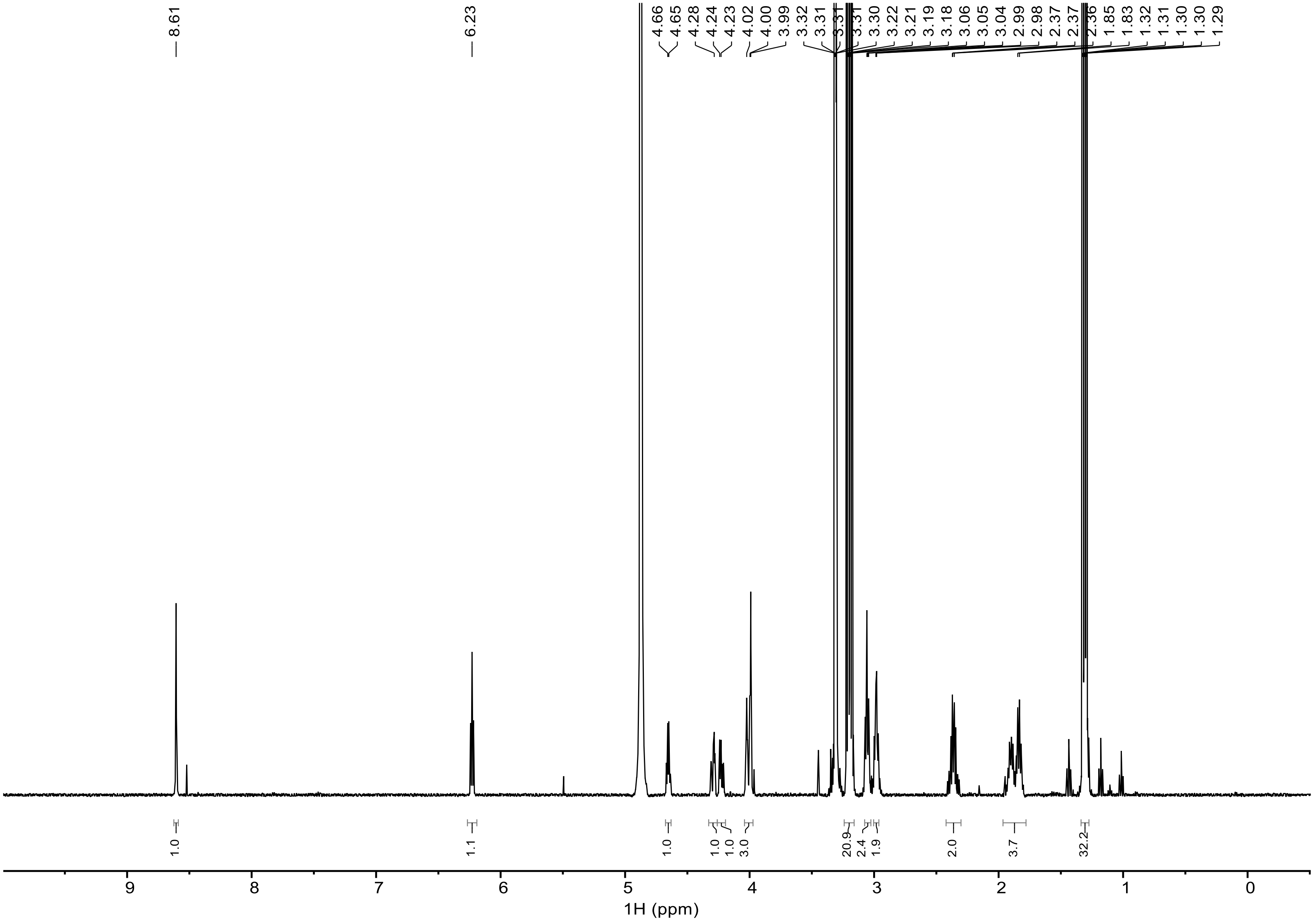

**$^{13}$C APT NMR (125.7 MHz, $CD_3OD$), compound dU$^{put}$TP**

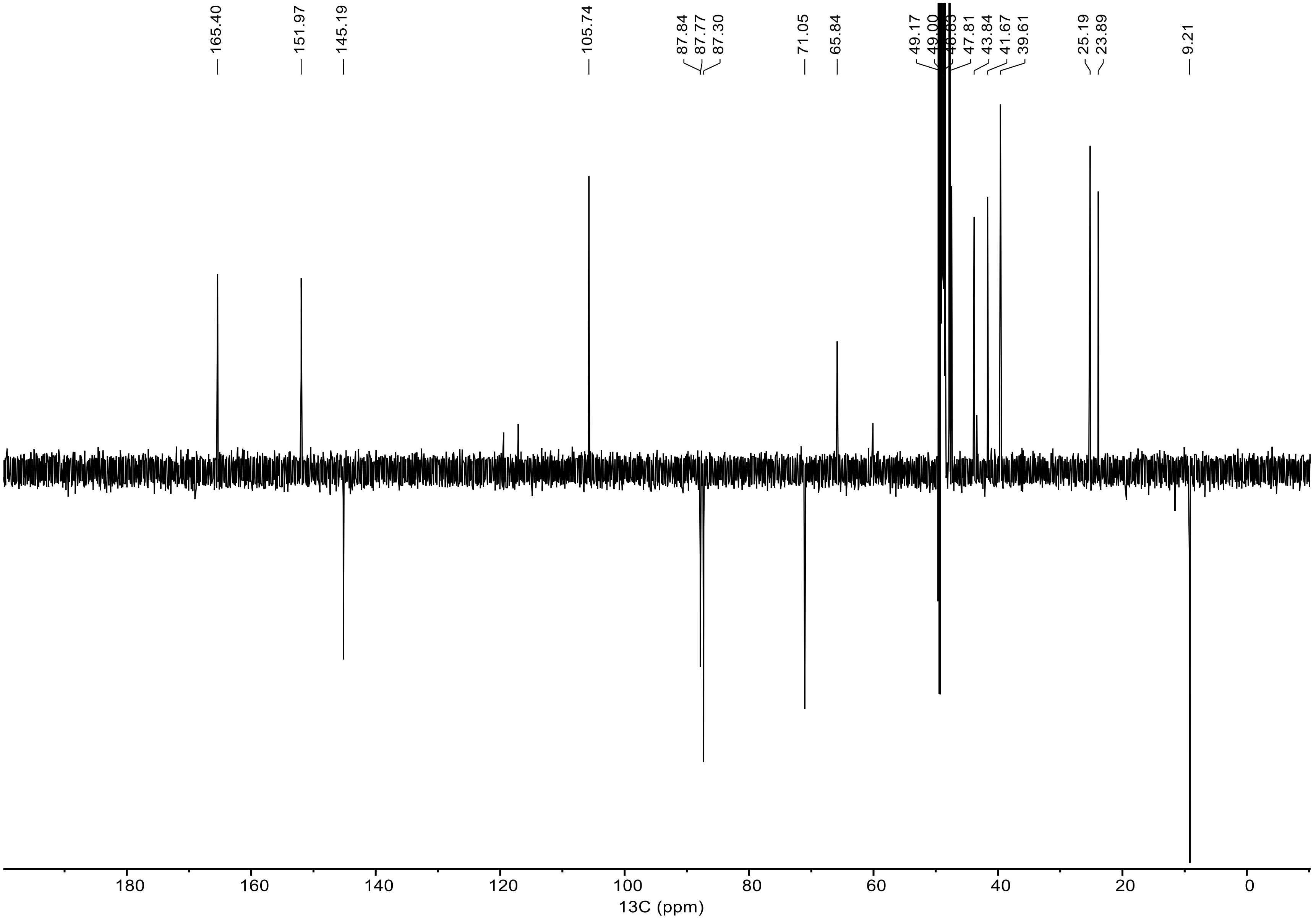

**$^{31}P\{^{1}H\}$ NMR (202.4 MHz, $CD_3OD$), compound dU[put]TP**

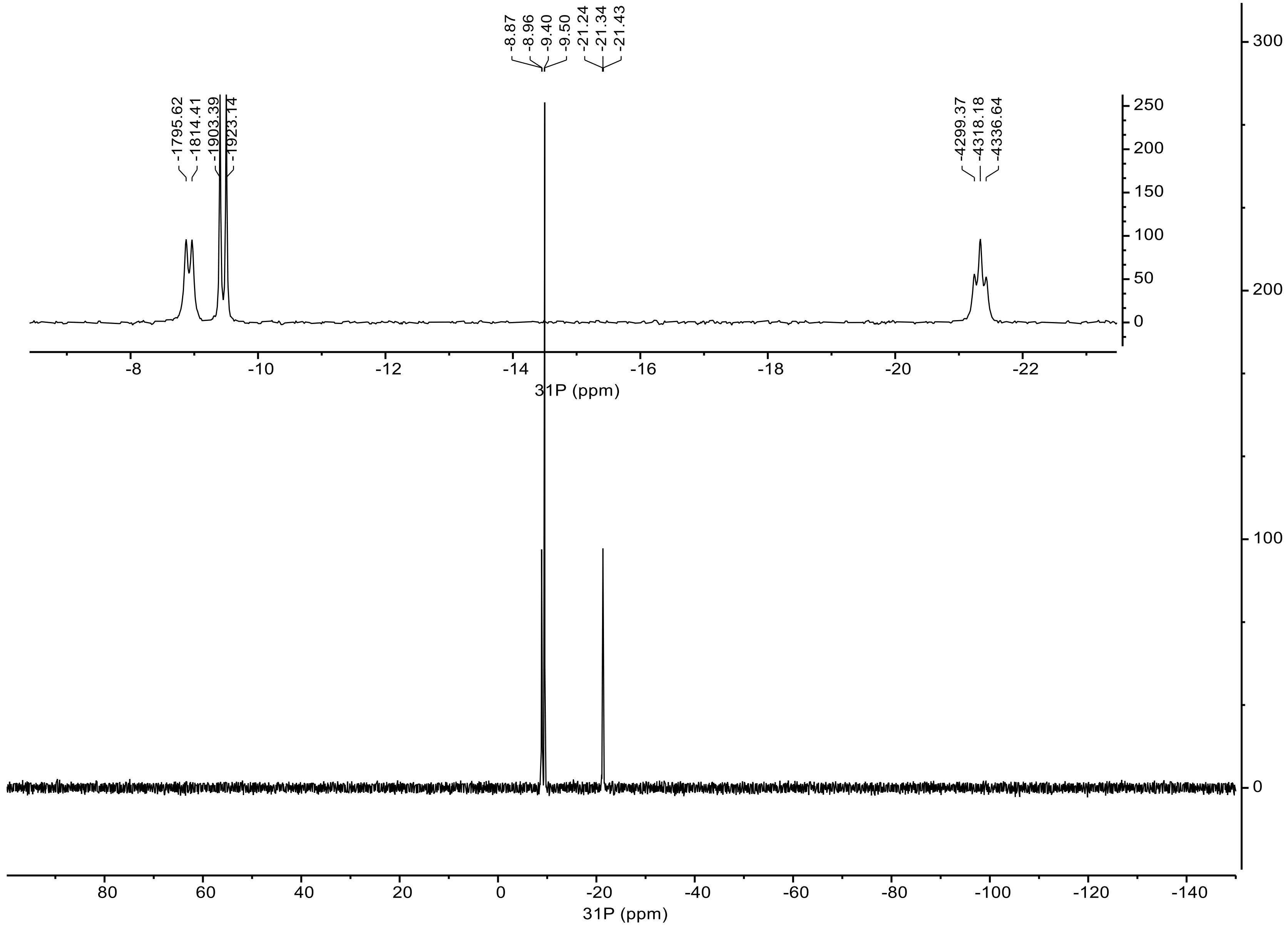

## $^1$H NMR (500.0 MHz, $D_2O$), compound dU$^{am}$TP

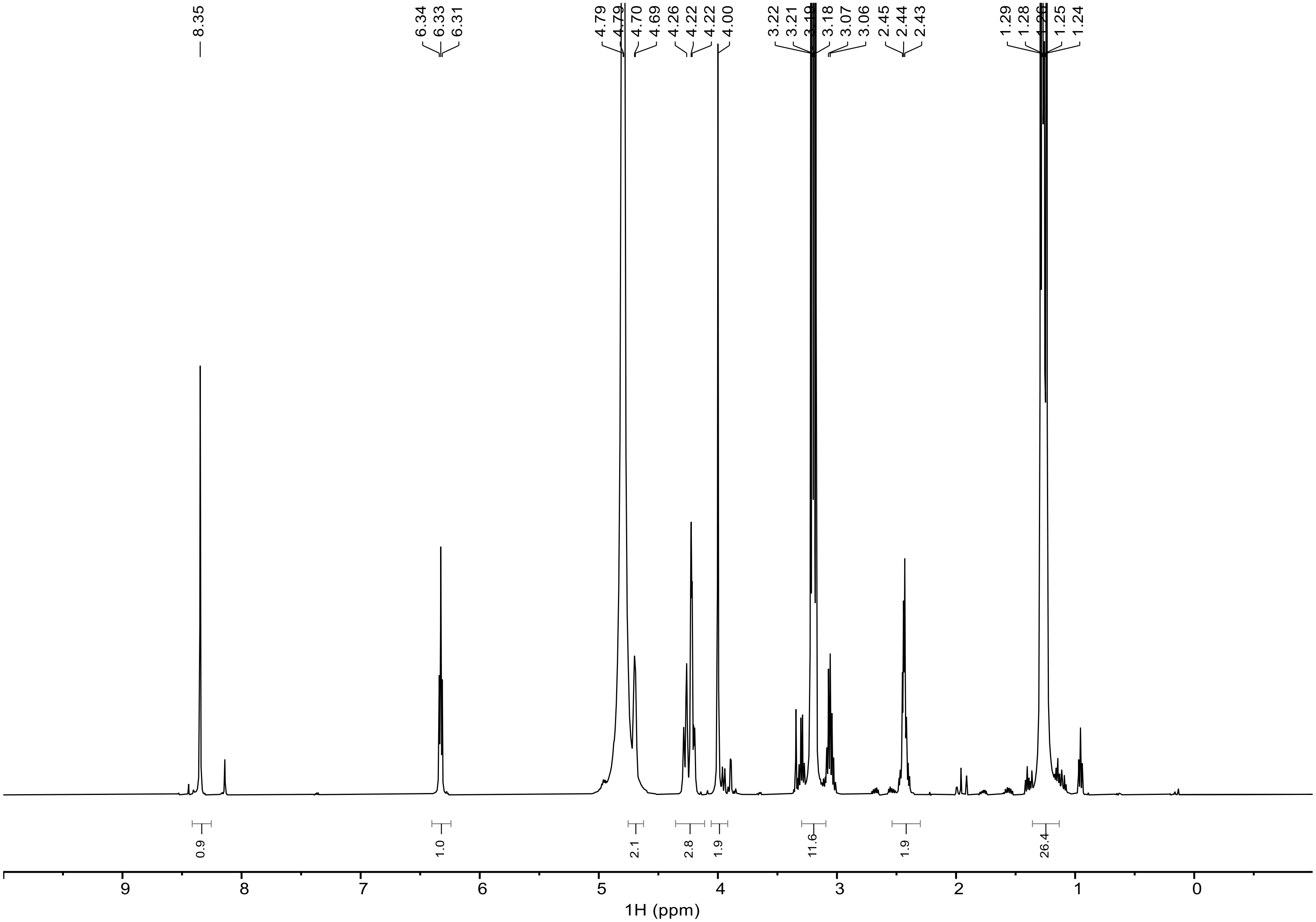

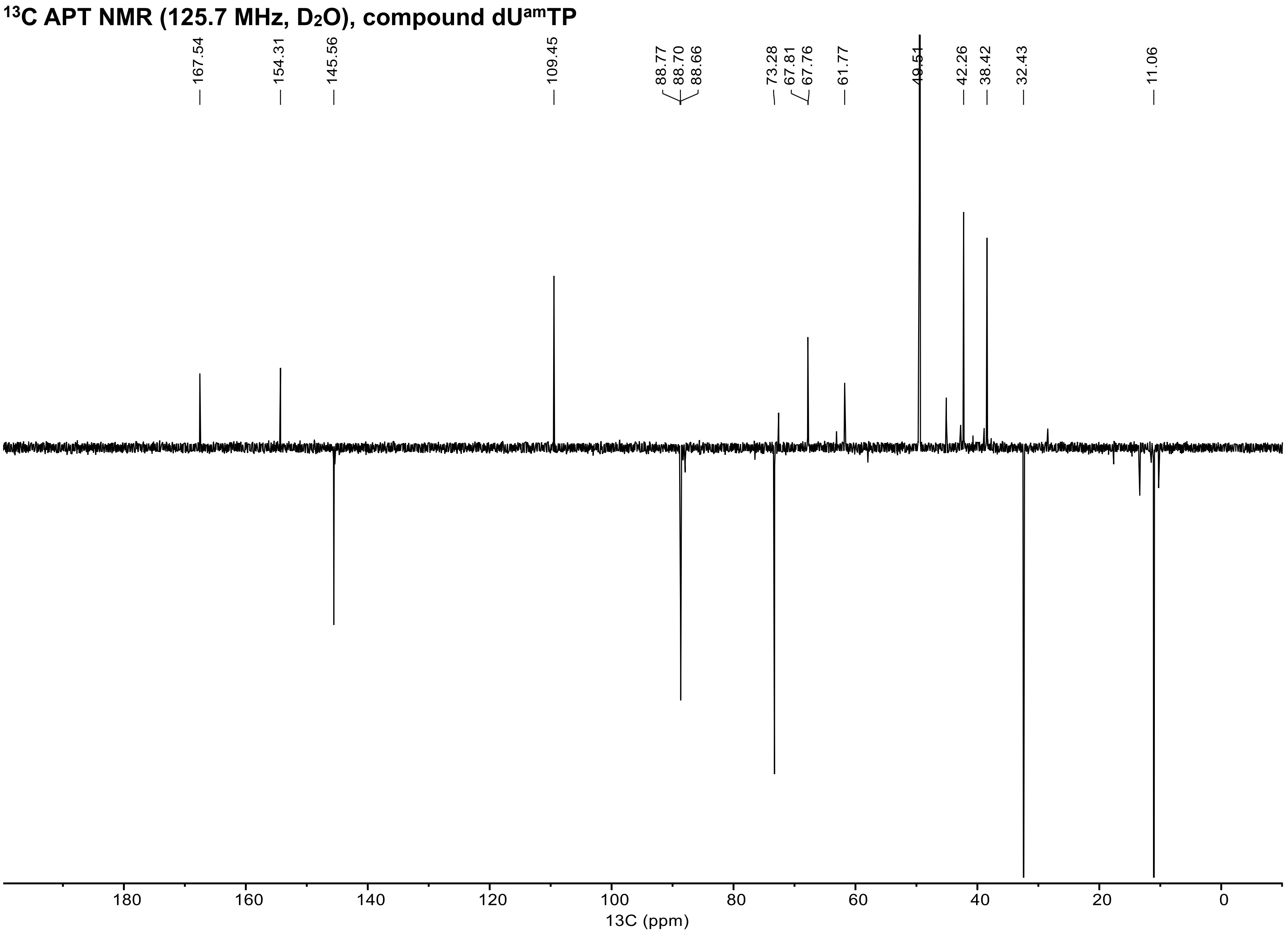

13C APT NMR (125.7 MHz, D2O), compound dUamTP
167.54
154.31
145.56
109.45
88.77
88.70
88.66
73.28
67.81
67.76
61.77
49.51
42.26
38.42
32.43
11.06
180
160
140
120
100
80
60
40
20
0
13C (ppm)

**$^{31}P\{^{1}H\}$ NMR (202.4 MHz, $D_2O$), compound dU$^{am}$TP**

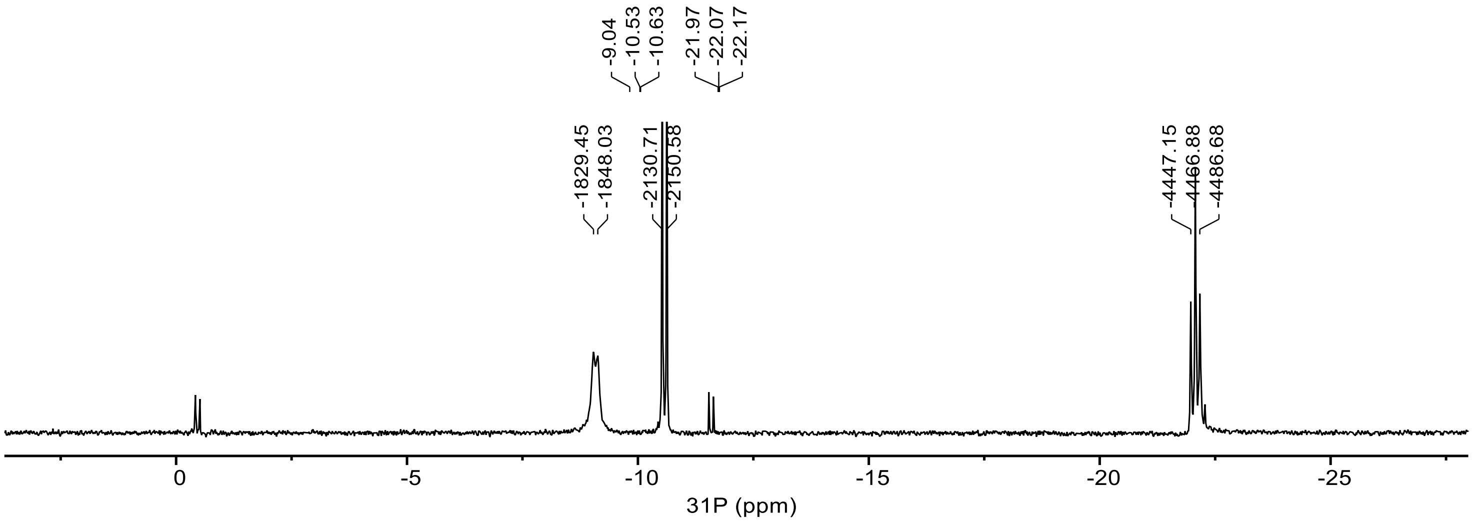


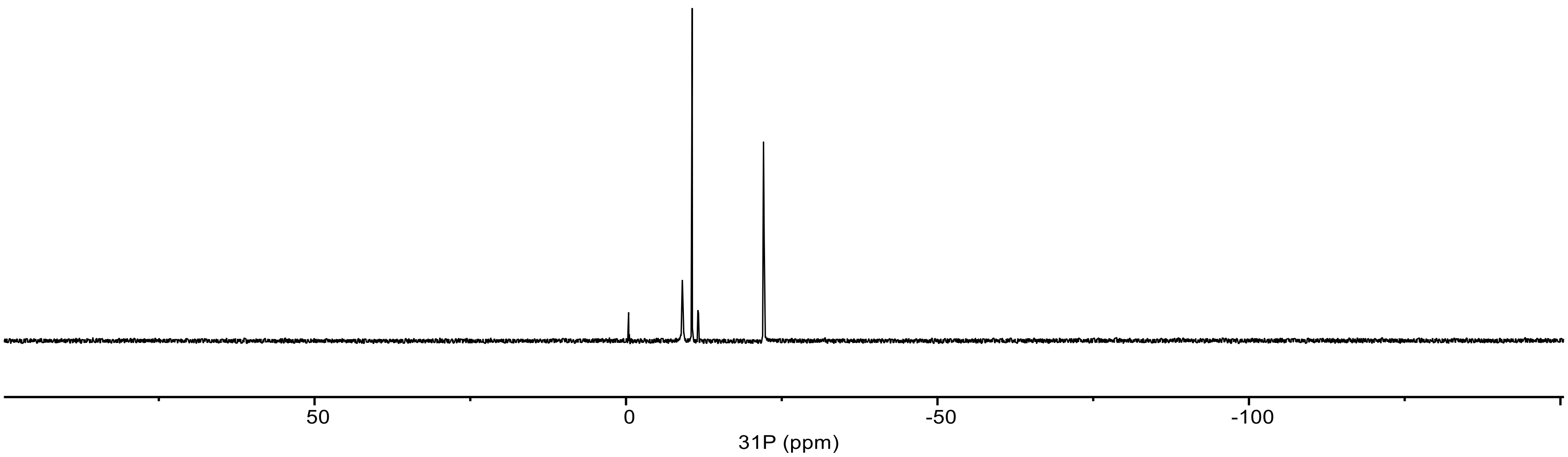

**$^1$H NMR (500.0 MHz, $D_2O$), compound dU$^{mm}$TP**

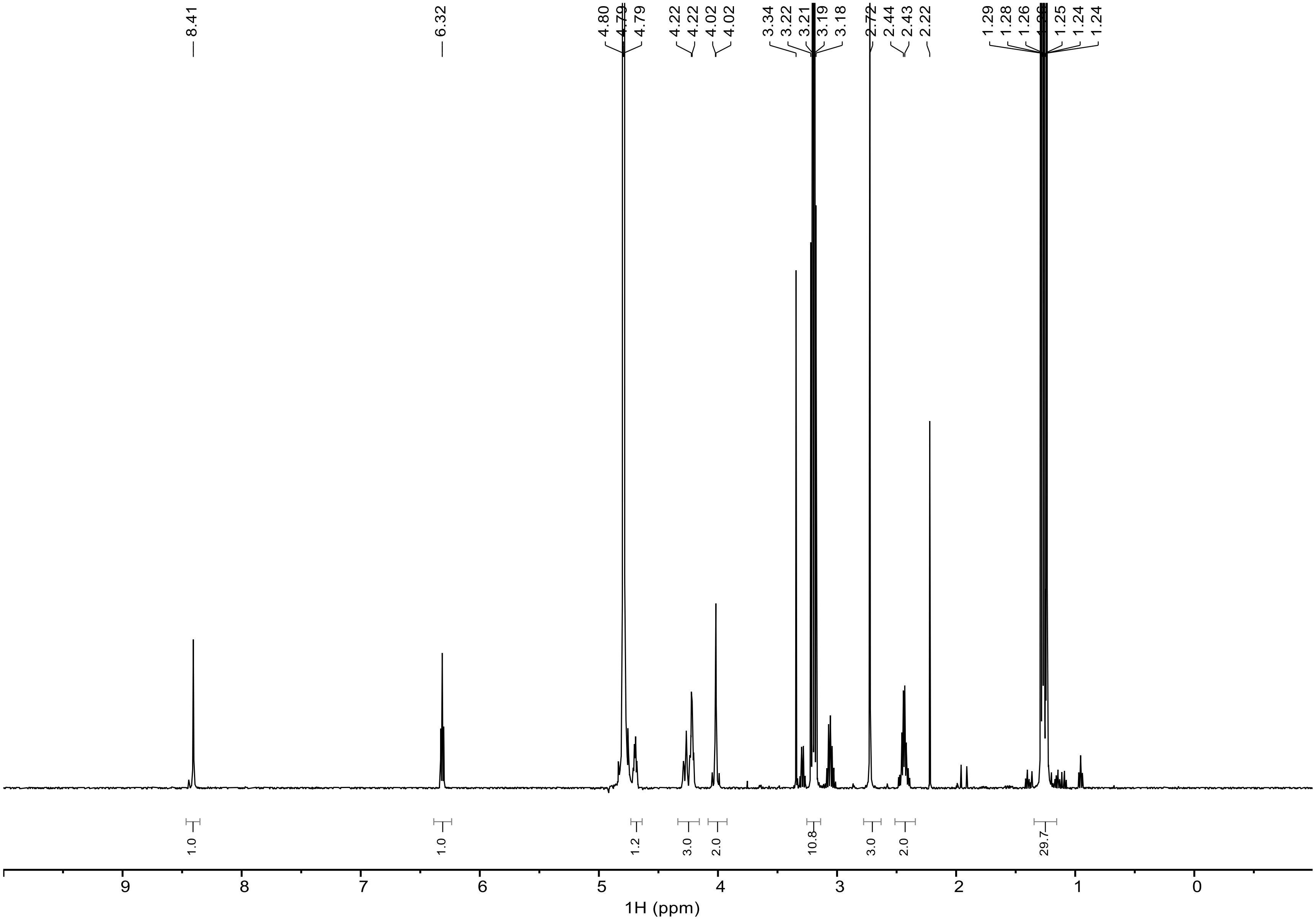

**$^{13}$C APT NMR (125.7 MHz, $D_2O$), compound dU[mm]TP**

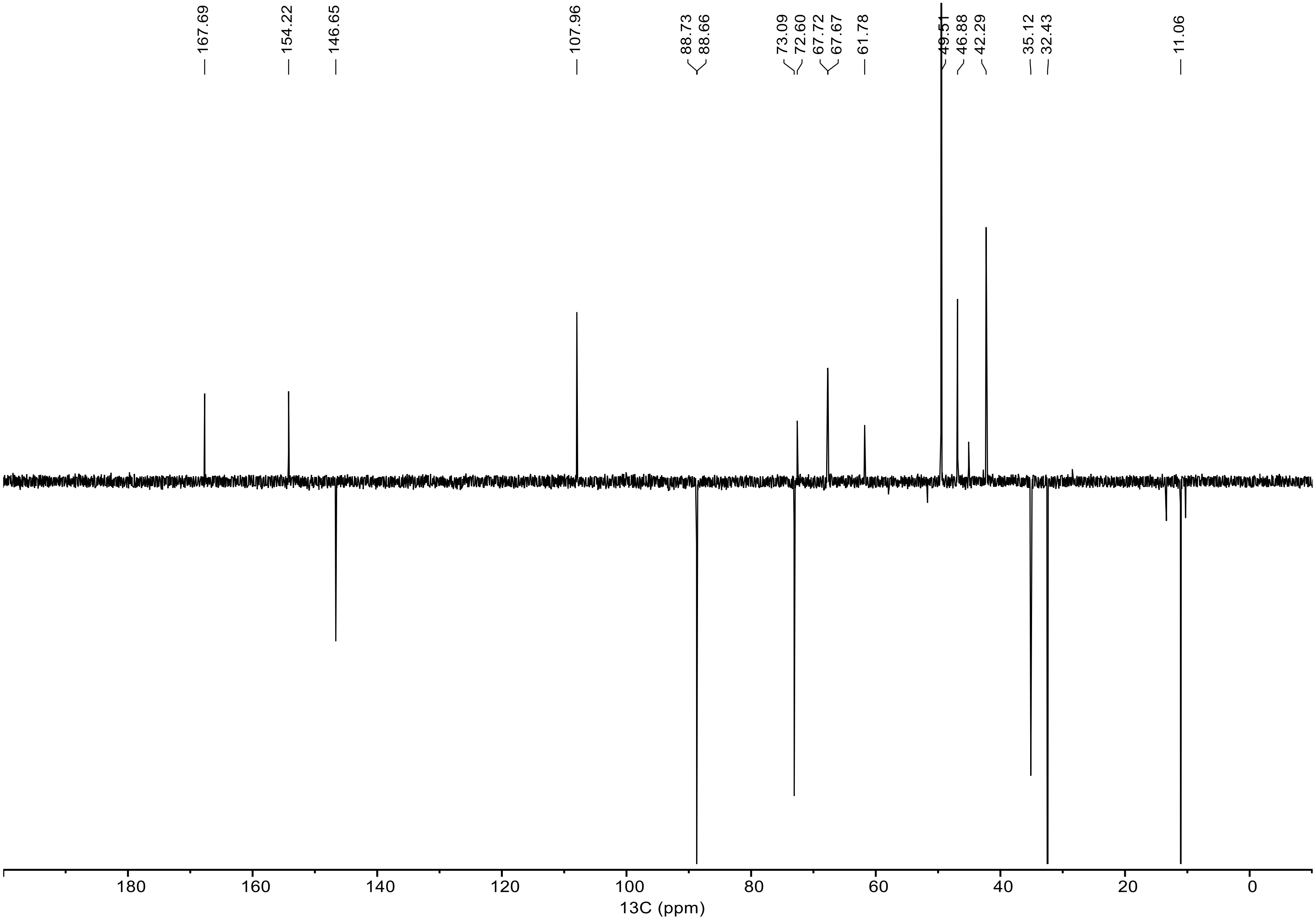

$^{31}P\{^1H\}$ NMR (202.4 MHz, $D_2O$), compound $dU^{mm}TP$

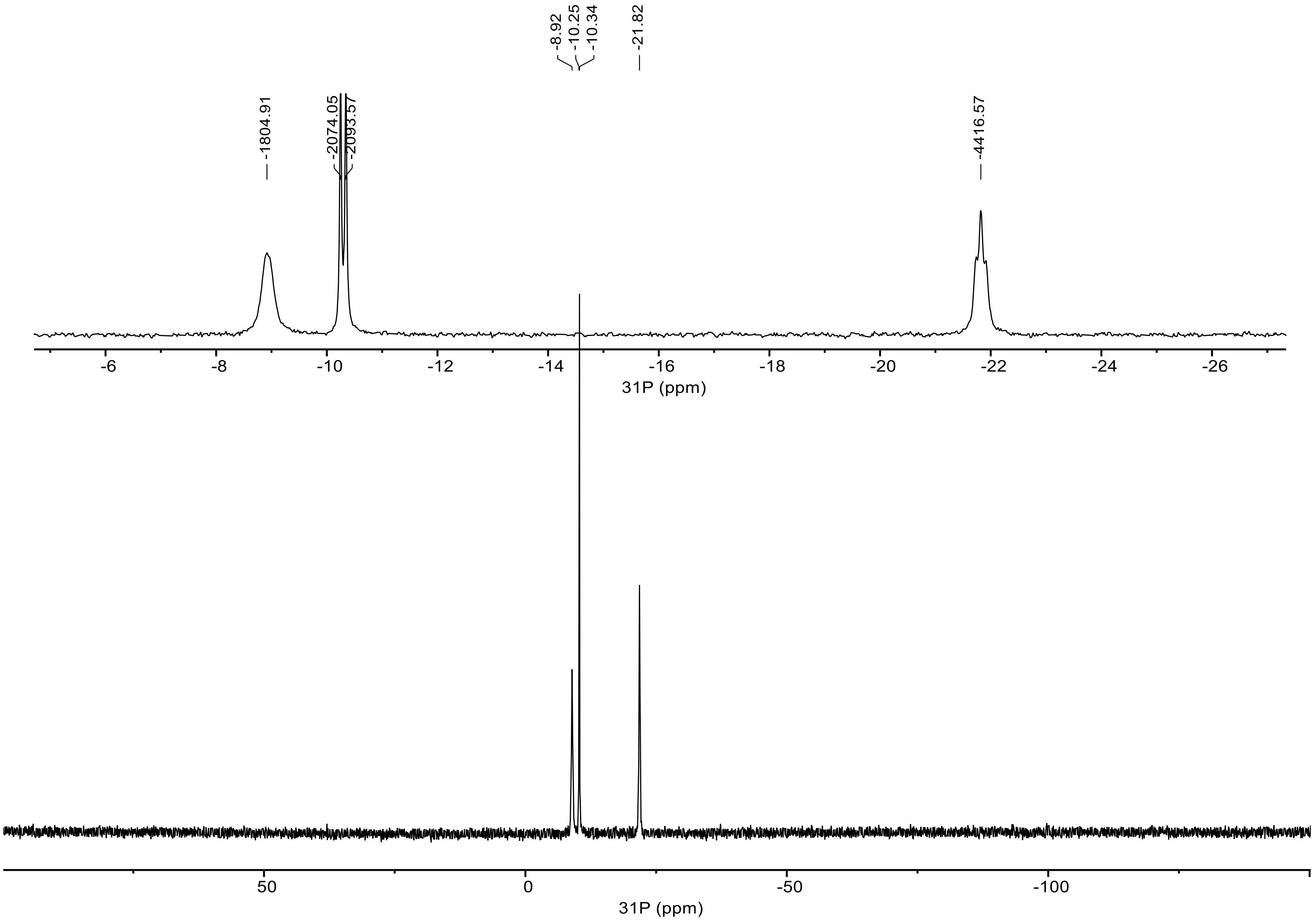

**$^{1}$H NMR (500.0 MHz, $D_2O$), compound dU[dm]TP**

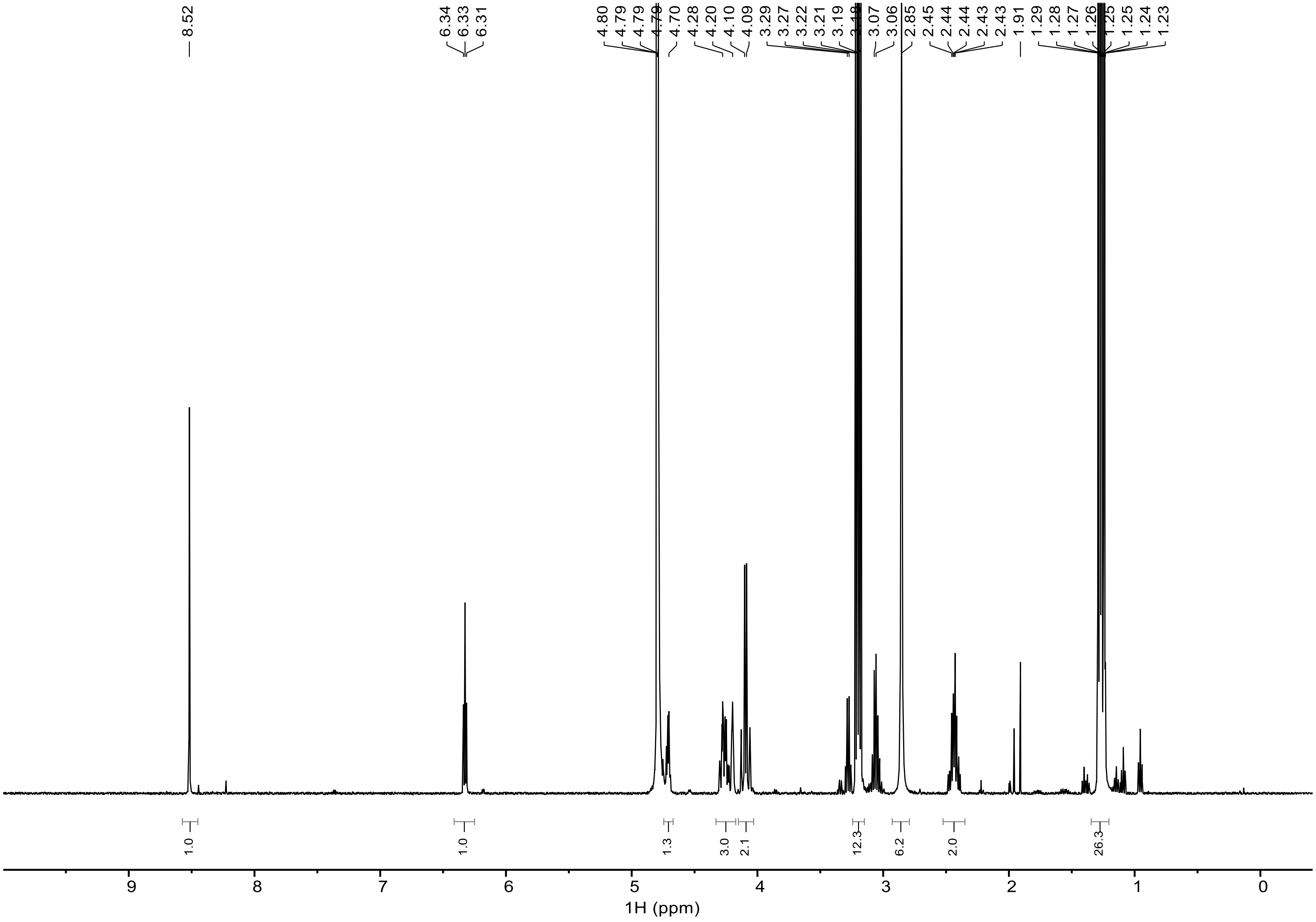

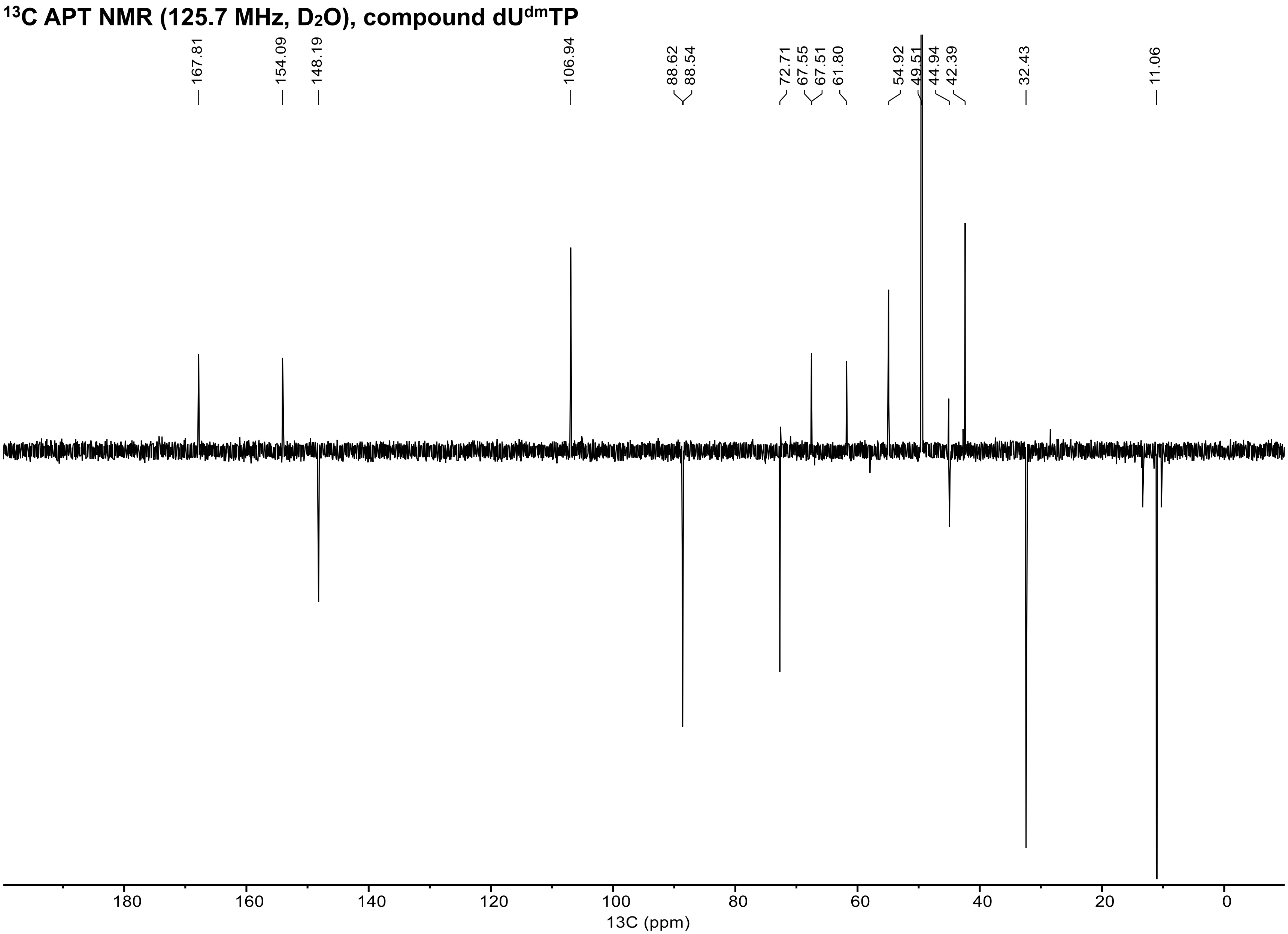

$^{13}$C APT NMR (125.7 MHz, $D_2O$), compound dU$^{dm}$TP
167.81
154.09
148.19
106.94
88.62
88.54
72.71
67.55
67.51
61.80
54.92
49.51
44.94
42.39
32.43
11.06
180
160
140
120
100
80
60
40
20
0
13C (ppm)

$^{31}P\{^1H\}$ NMR (202.4 MHz, $D_2O$), compound dU$^{dm}$TP

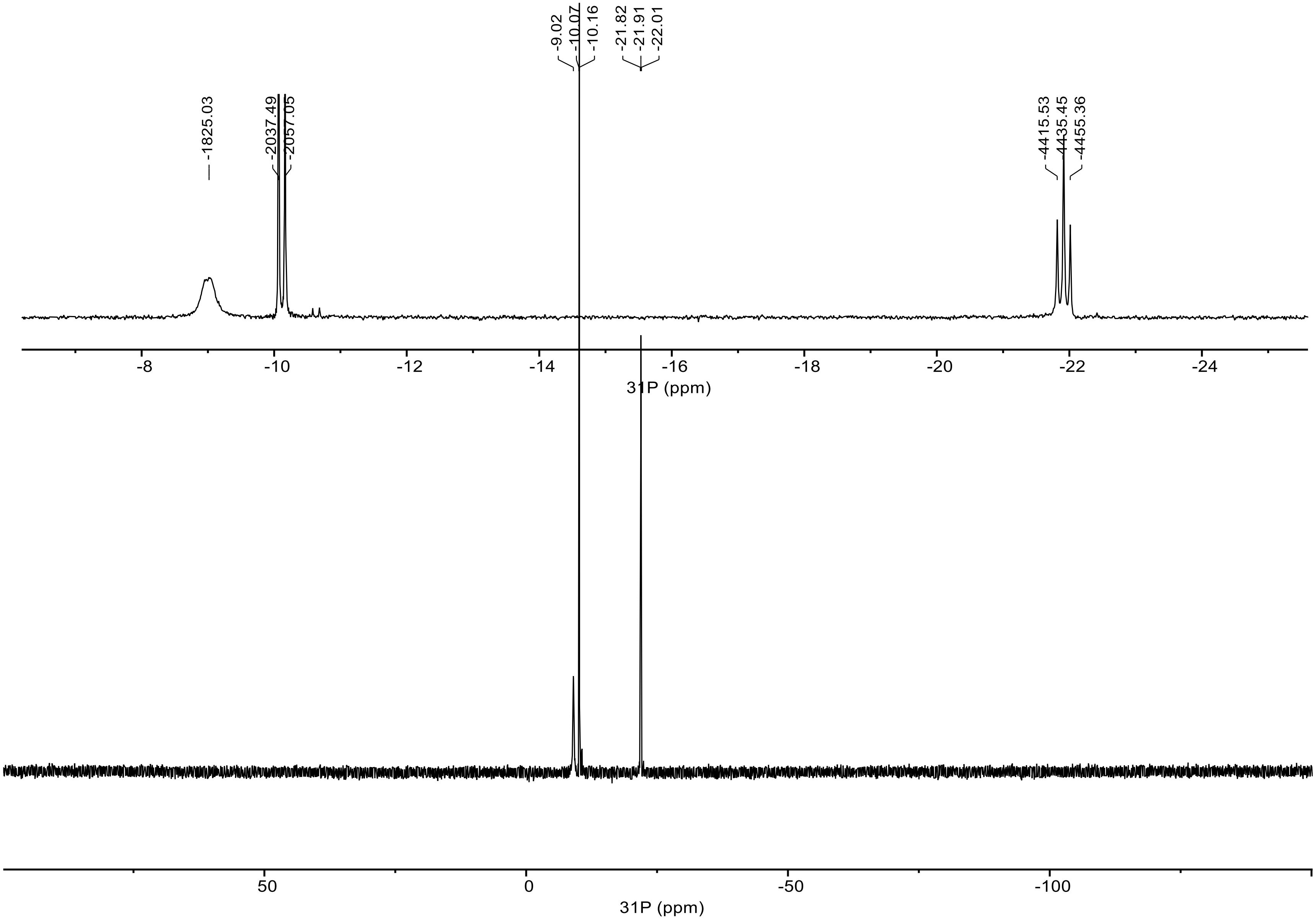

**$^1$H NMR (500.0 MHz, $D_2O$), compound dU$^{cm}$TP**

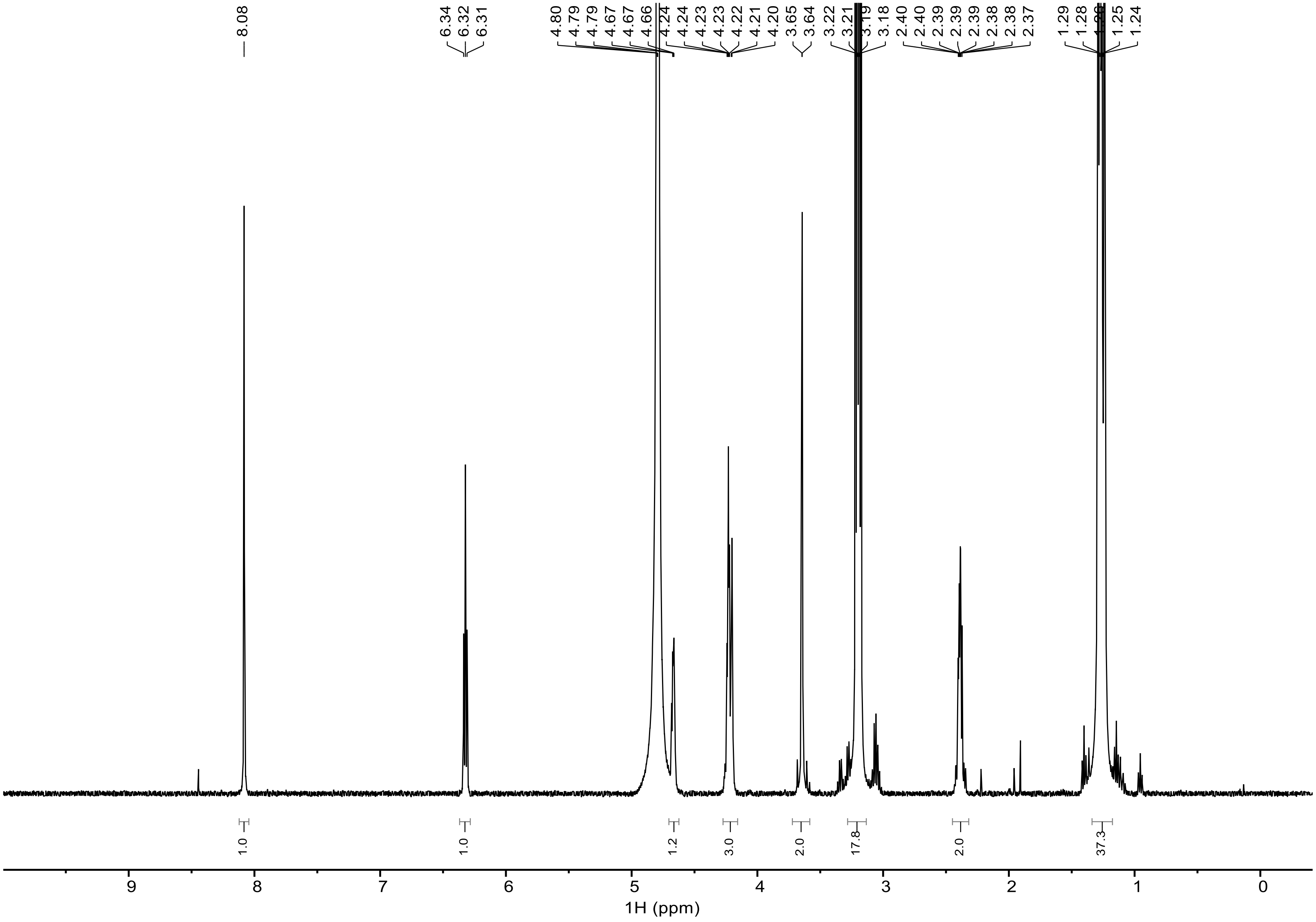

## $^{13}$C APT NMR (125.7 MHz, $D_2O$), compound dU$^{cm}$TP

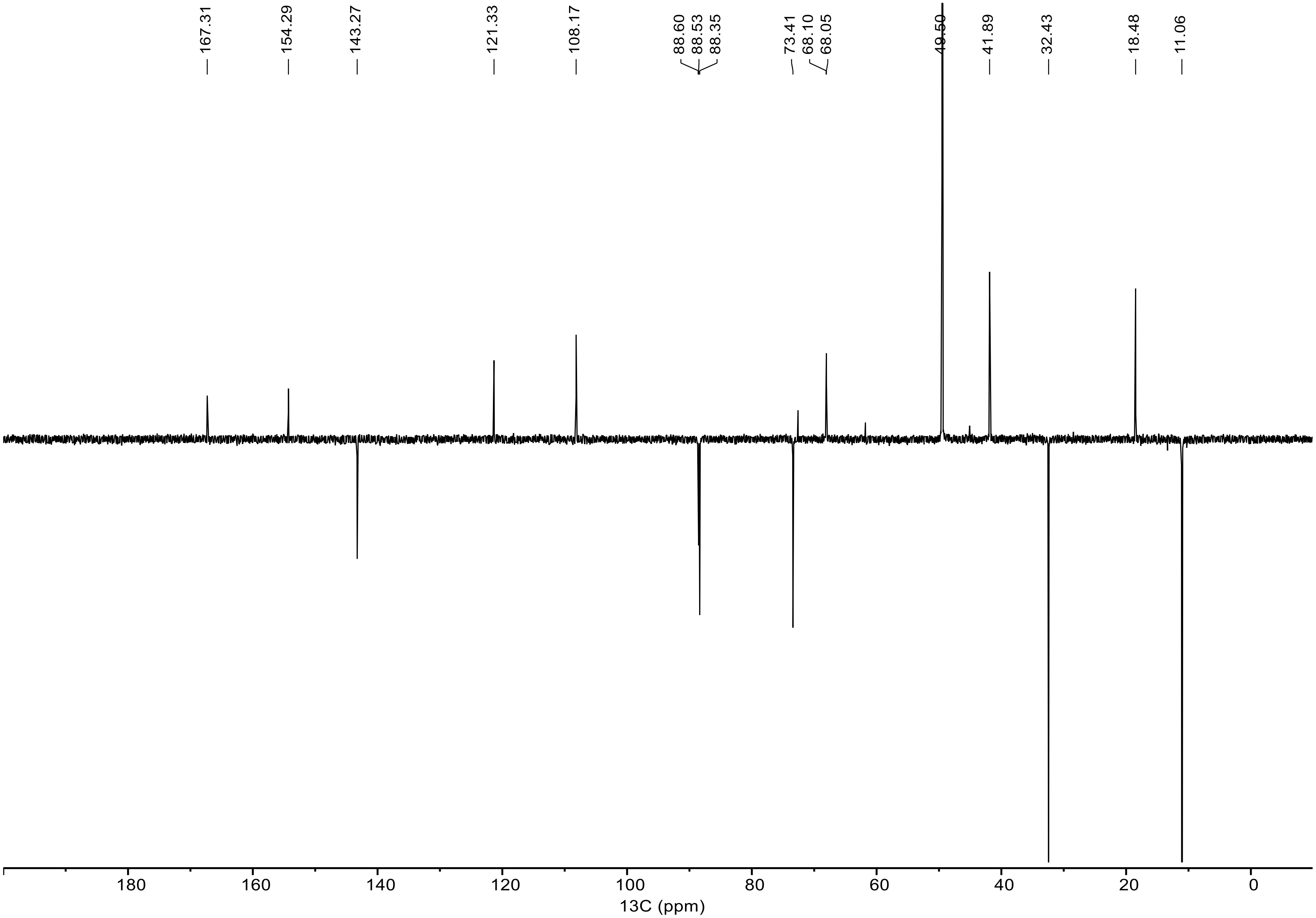

**$^{31}P\{^{1}H\}$ NMR (202.4 MHz, $D_2O$), compound dU$^{cm}$TP**

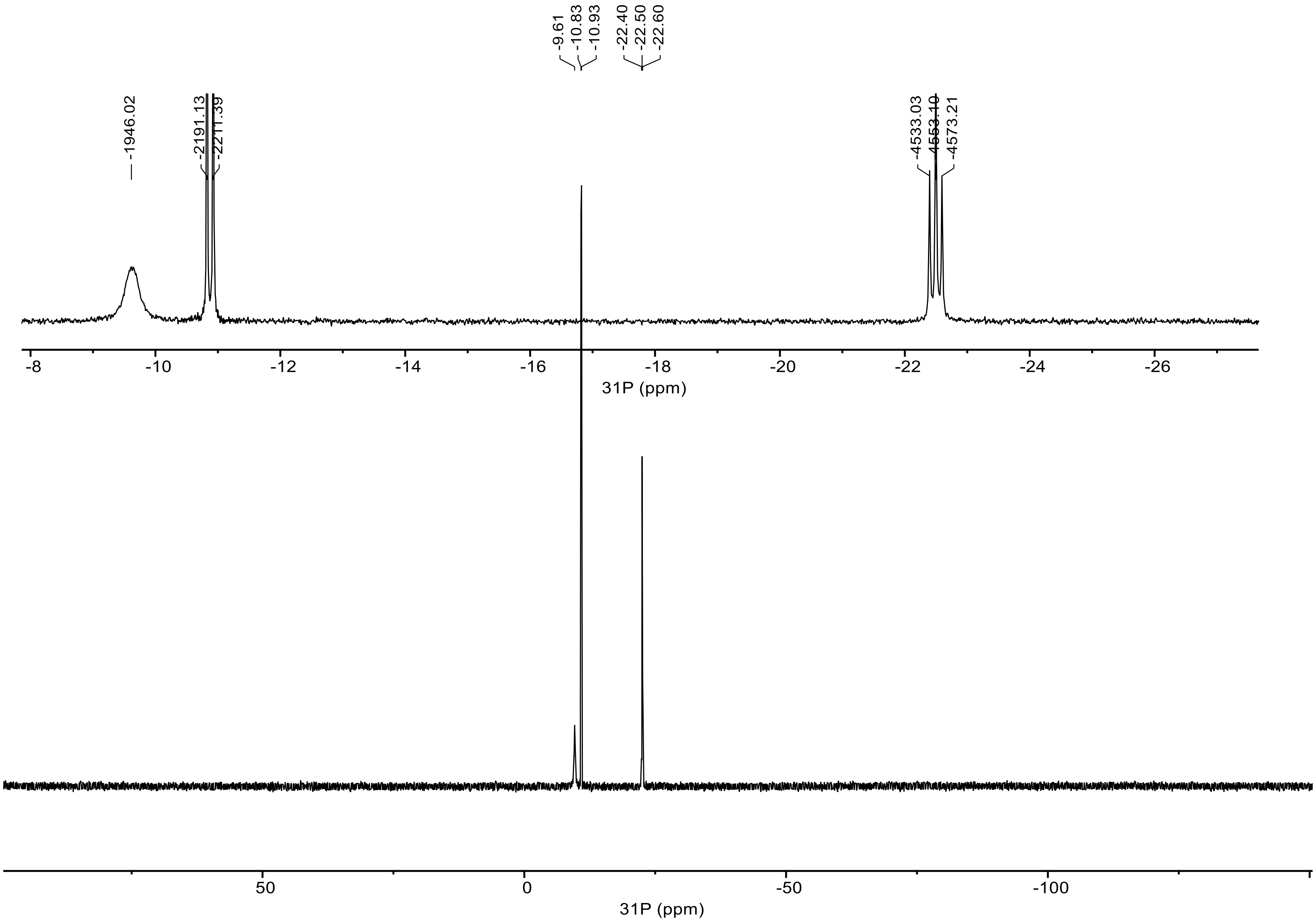

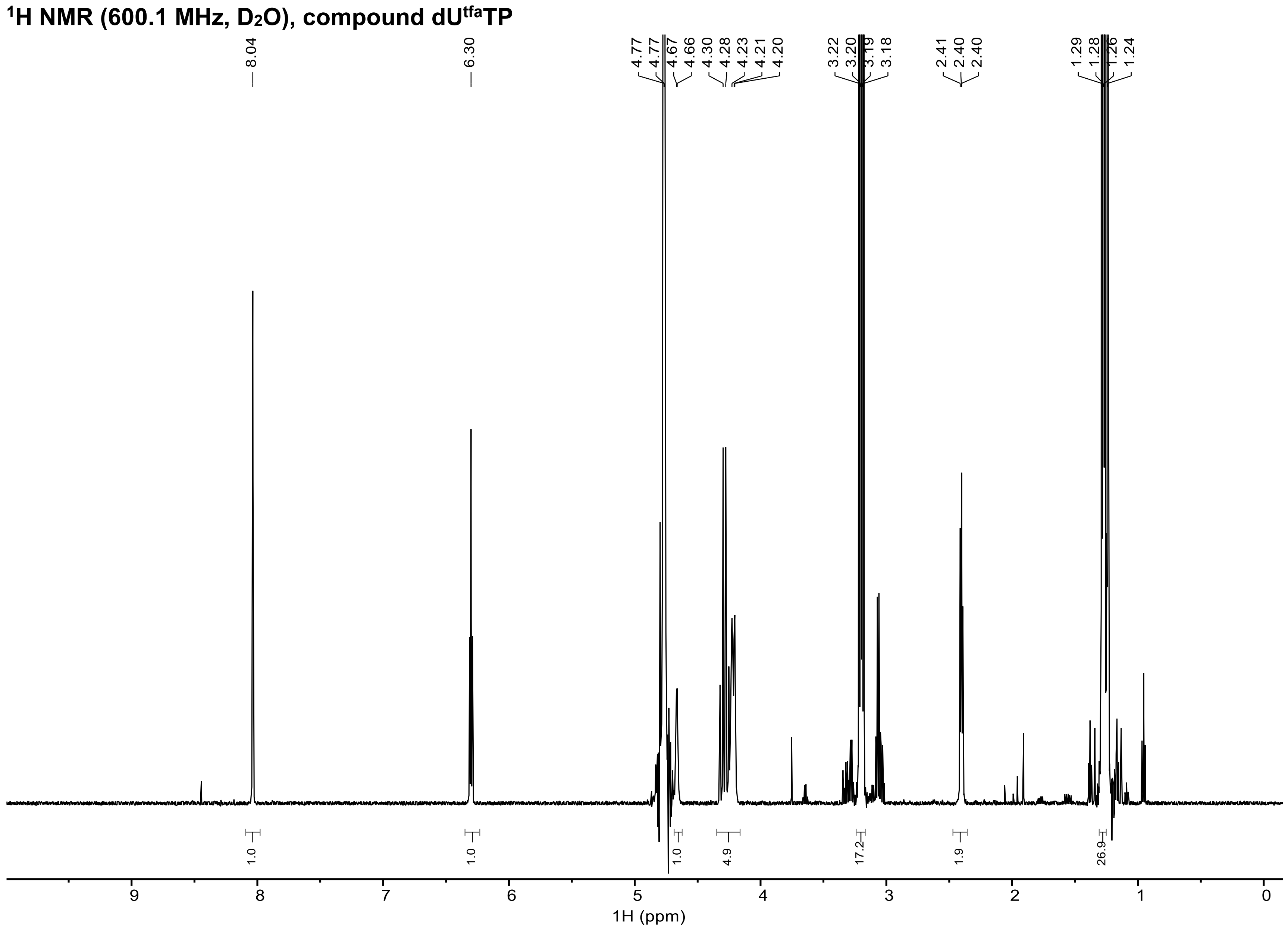

1H NMR (600.1 MHz, D2O), compound dUtfaTP
8.04
6.30
4.77
4.77
4.67
4.66
4.30
4.28
4.23
4.21
4.20
3.22
3.20
3.19
3.18
2.41
2.40
2.40
1.29
1.28
1.26
1.24
1.0
1.0
1.0
4.9
17.2
1.9
26.9
9
8
7
6
5
4
3
2
1
0
1H (ppm)

**$^{13}$C APT NMR (150.9 MHz, $D_2O$), compound dU$^{tfa}$TP**

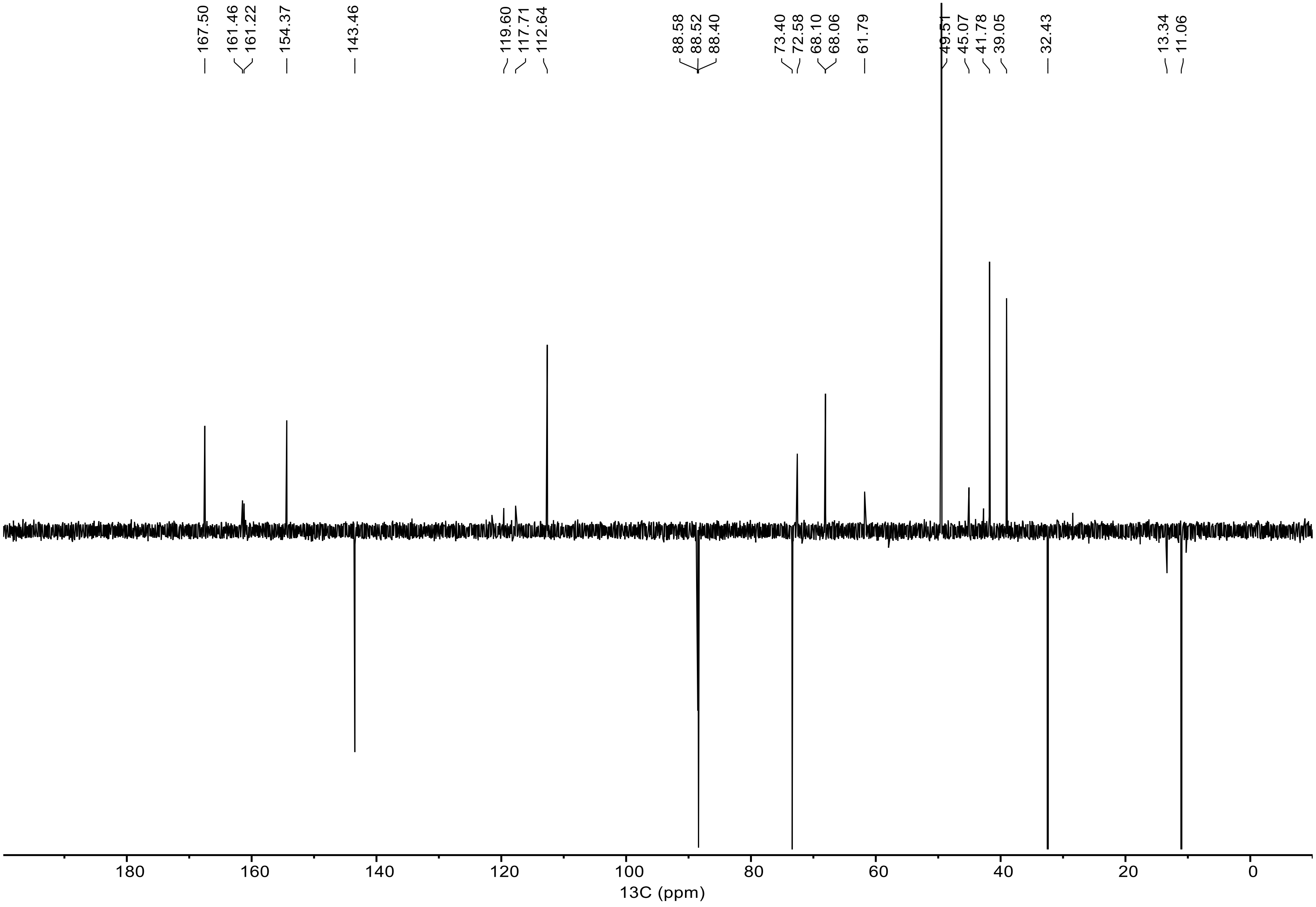

**$^{19}F$ NMR (470.7 MHz, $D_2O$), compound dU$^{tfa}$TP**

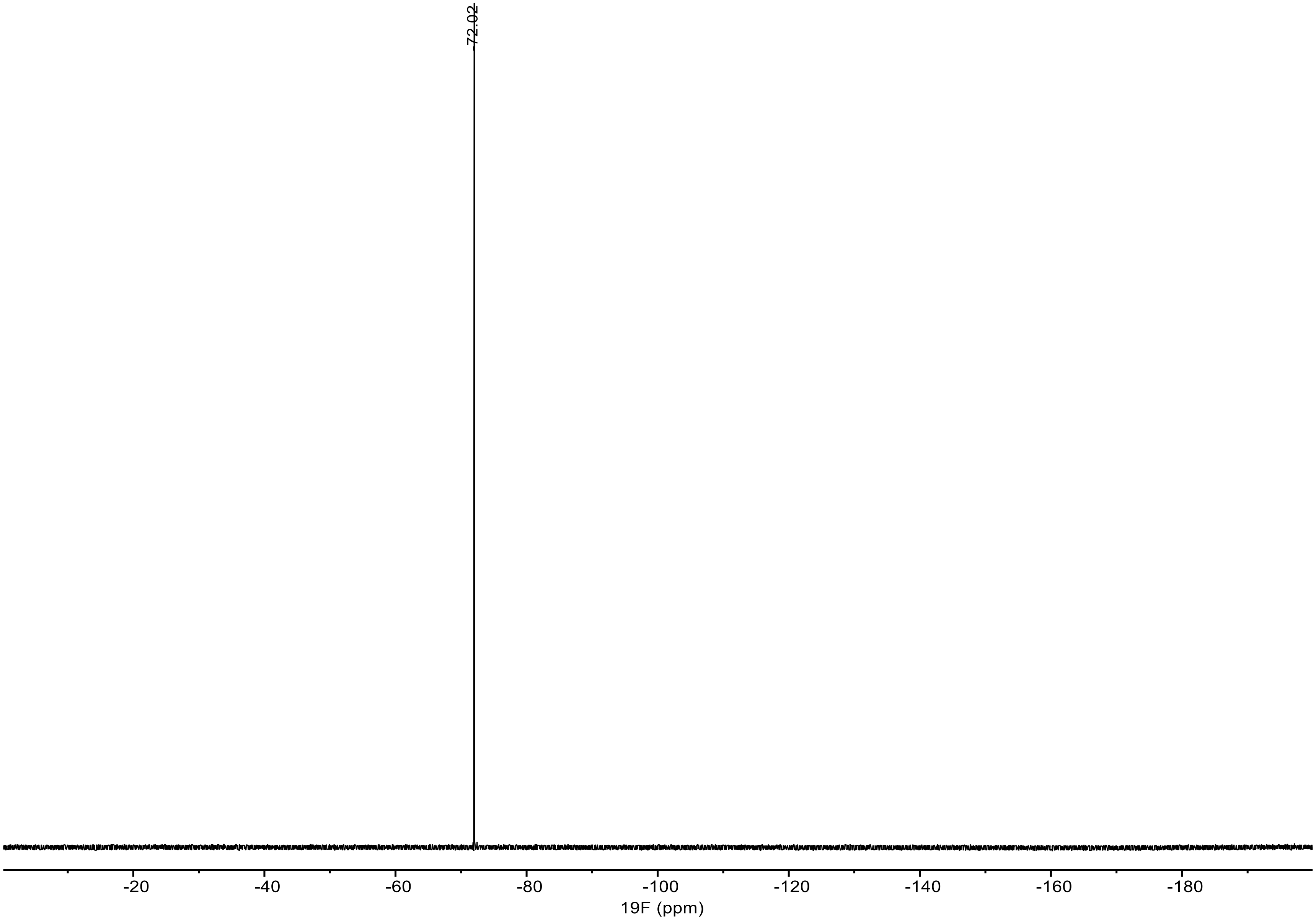

**$^{31}P\{^{1}H\}$ NMR (202.4 MHz, $D_2O$), compound dU[tfa]TP**

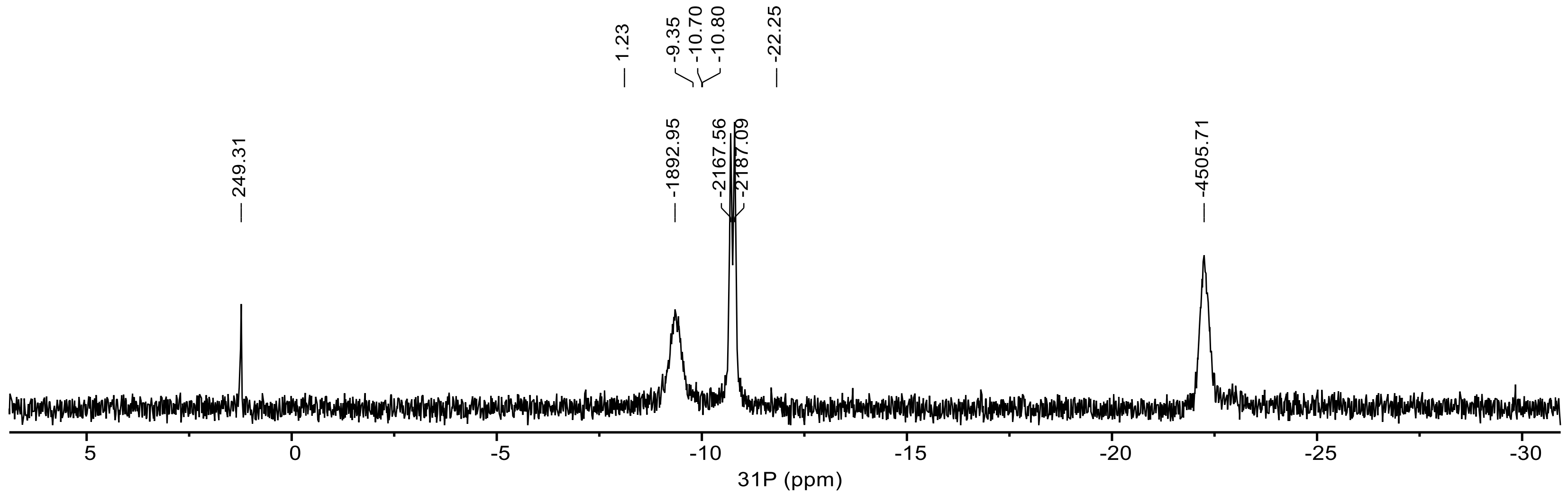


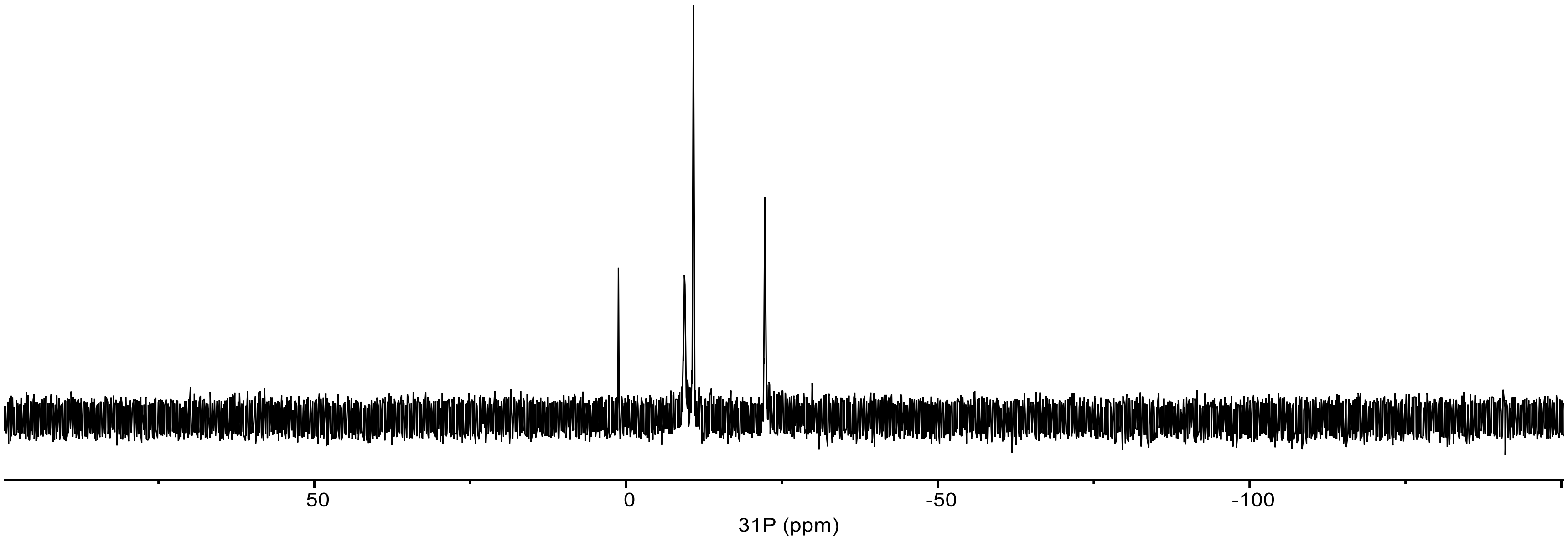

## $^1$H NMR (500.0 MHz, $D_2O$), compound dU$^{asm}$TP

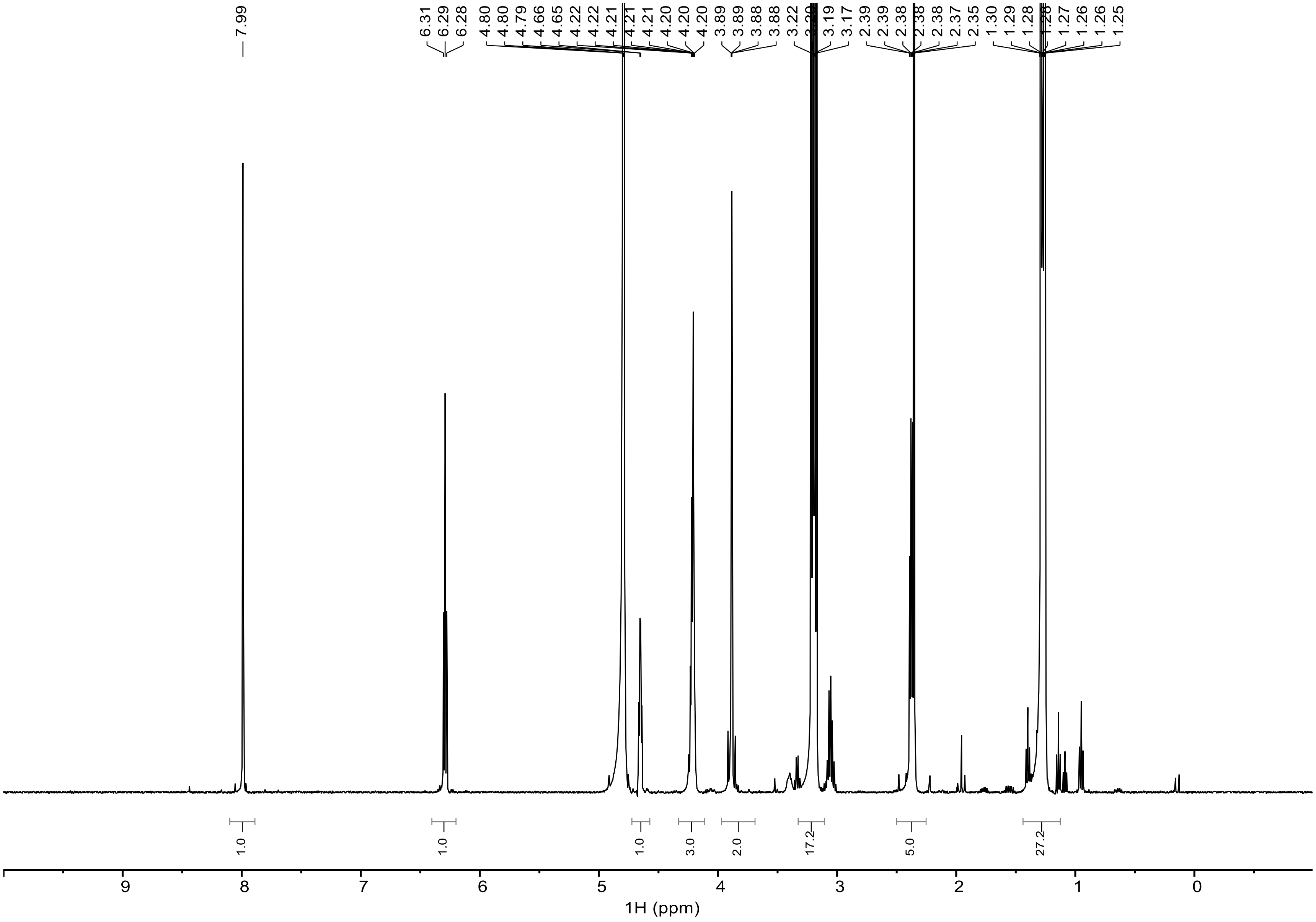

## $^{13}C$ APT NMR (125.7 MHz, $D_2O$), compound dU$^{asm}$TP

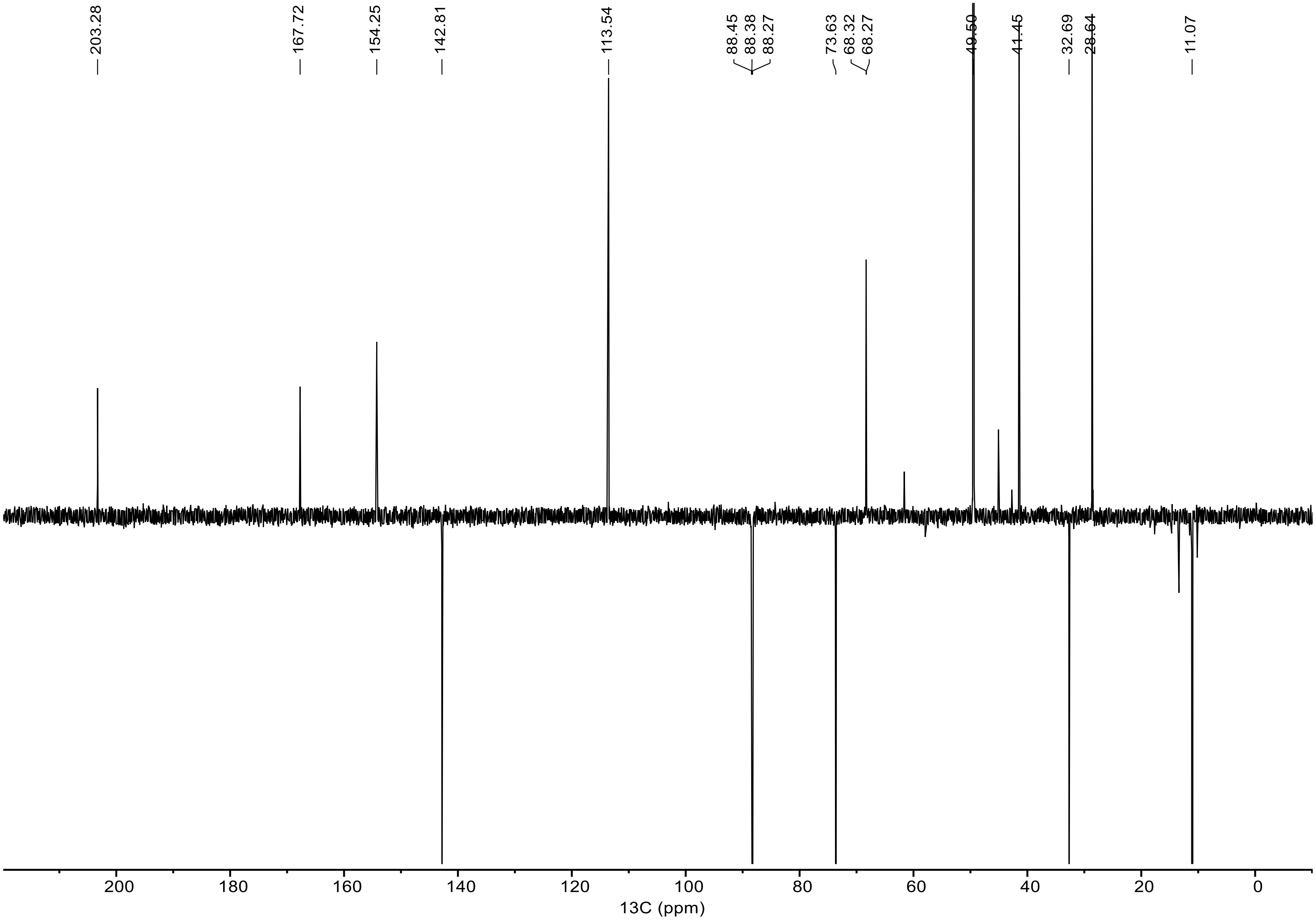

**$^{31}P\{^{1}H\}$ NMR (202.4 MHz, $D_2O$), compound dU$^{asm}$TP**

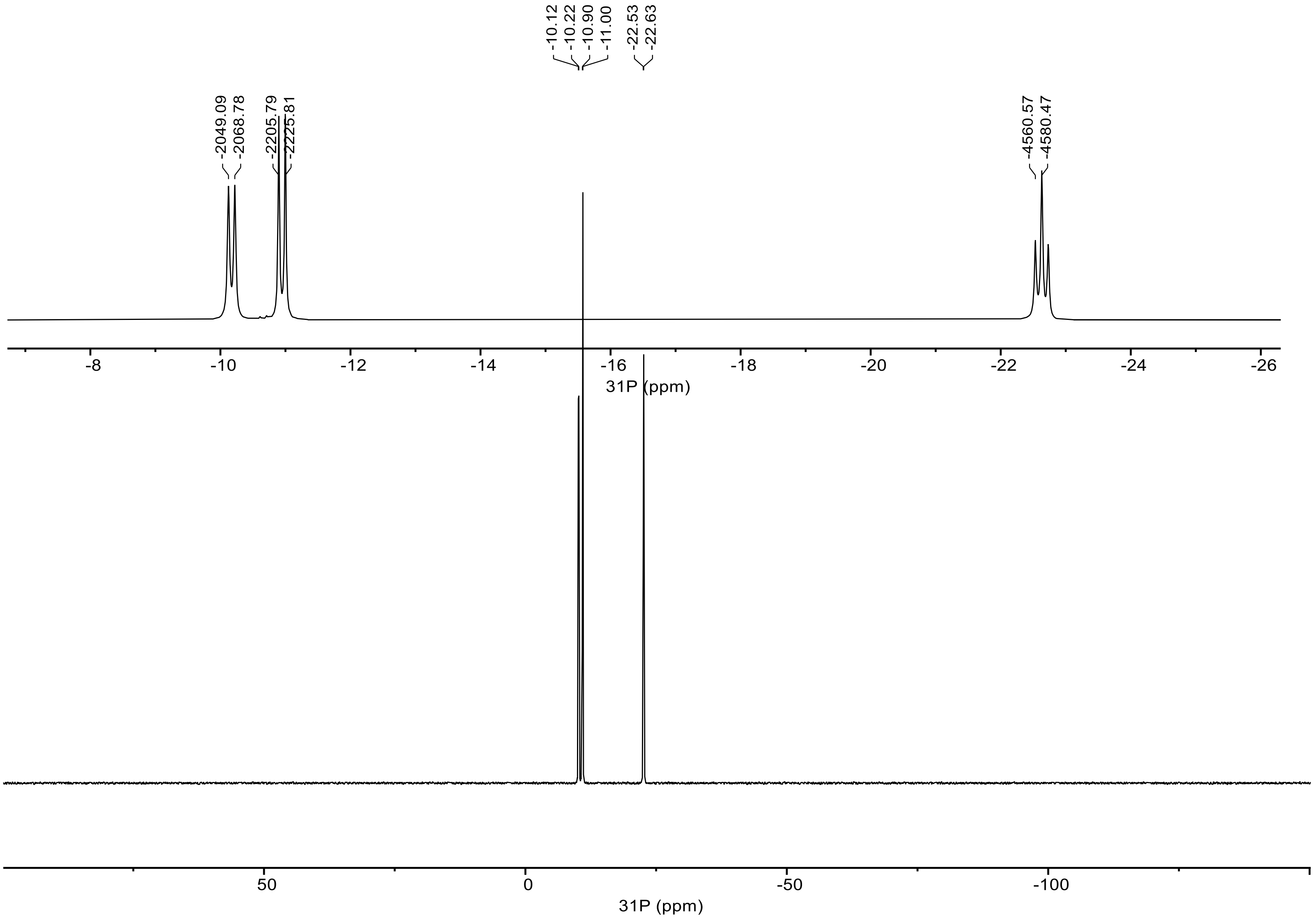

## $^1H$ NMR (500.0 MHz, $D_2O$), compound dU$^{sm}$TP

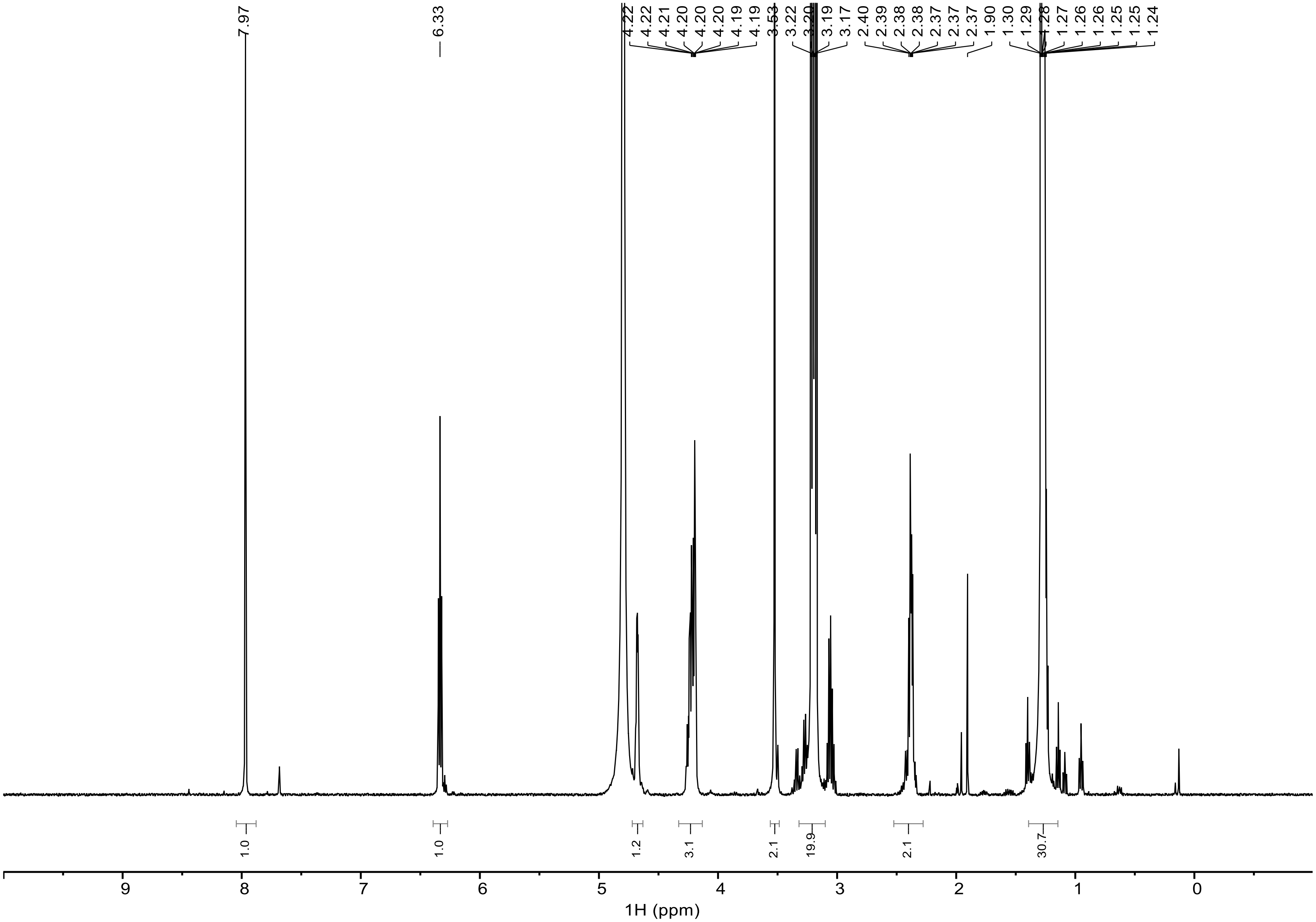

## $^{13}C$ APT NMR (125.7 MHz, $D_2O$), compound $dU^{sm}TP$

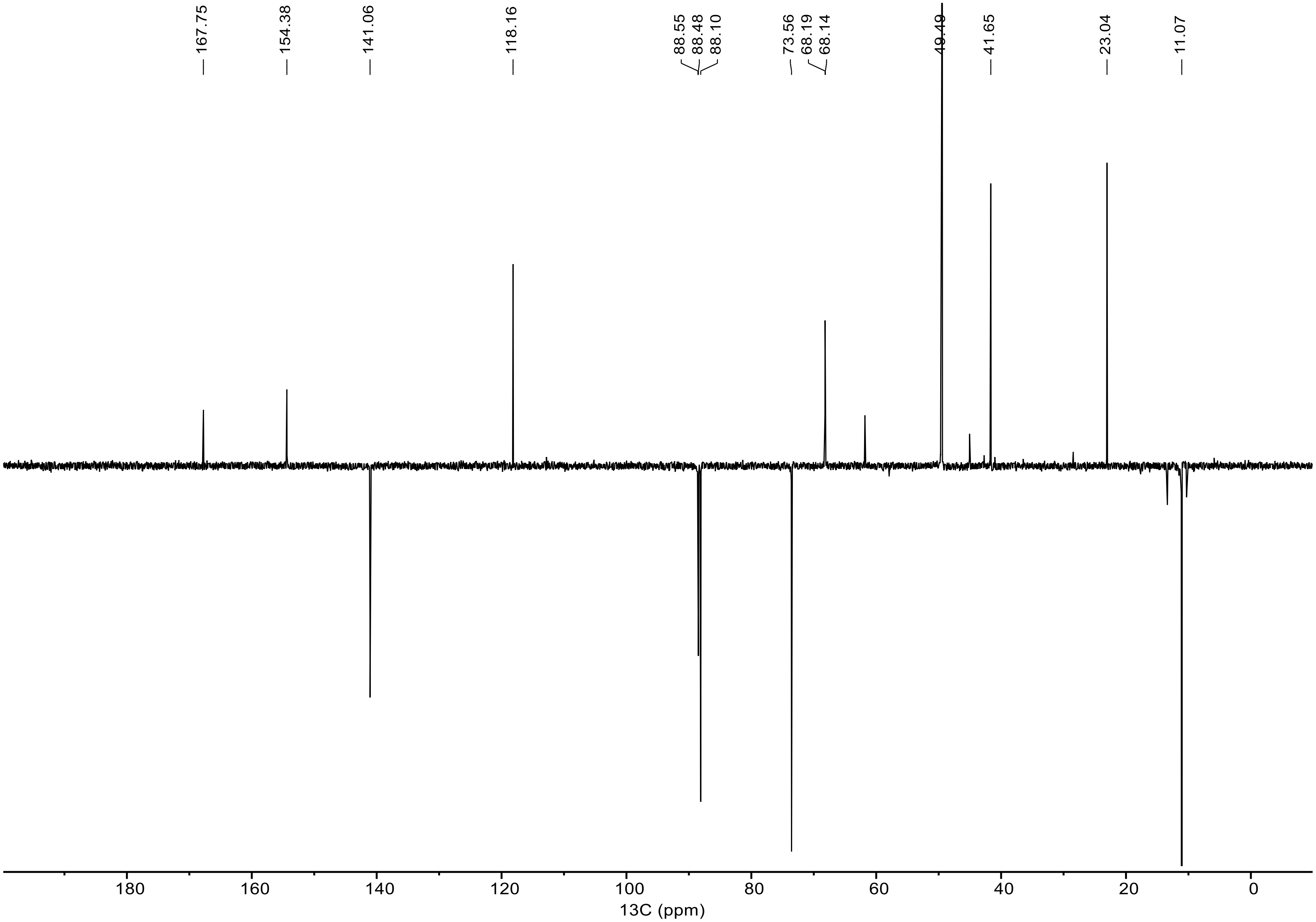

**$^{31}P\{^{1}H\}$ NMR (202.4 MHz, $D_2O$), compound dU^sm^TP**

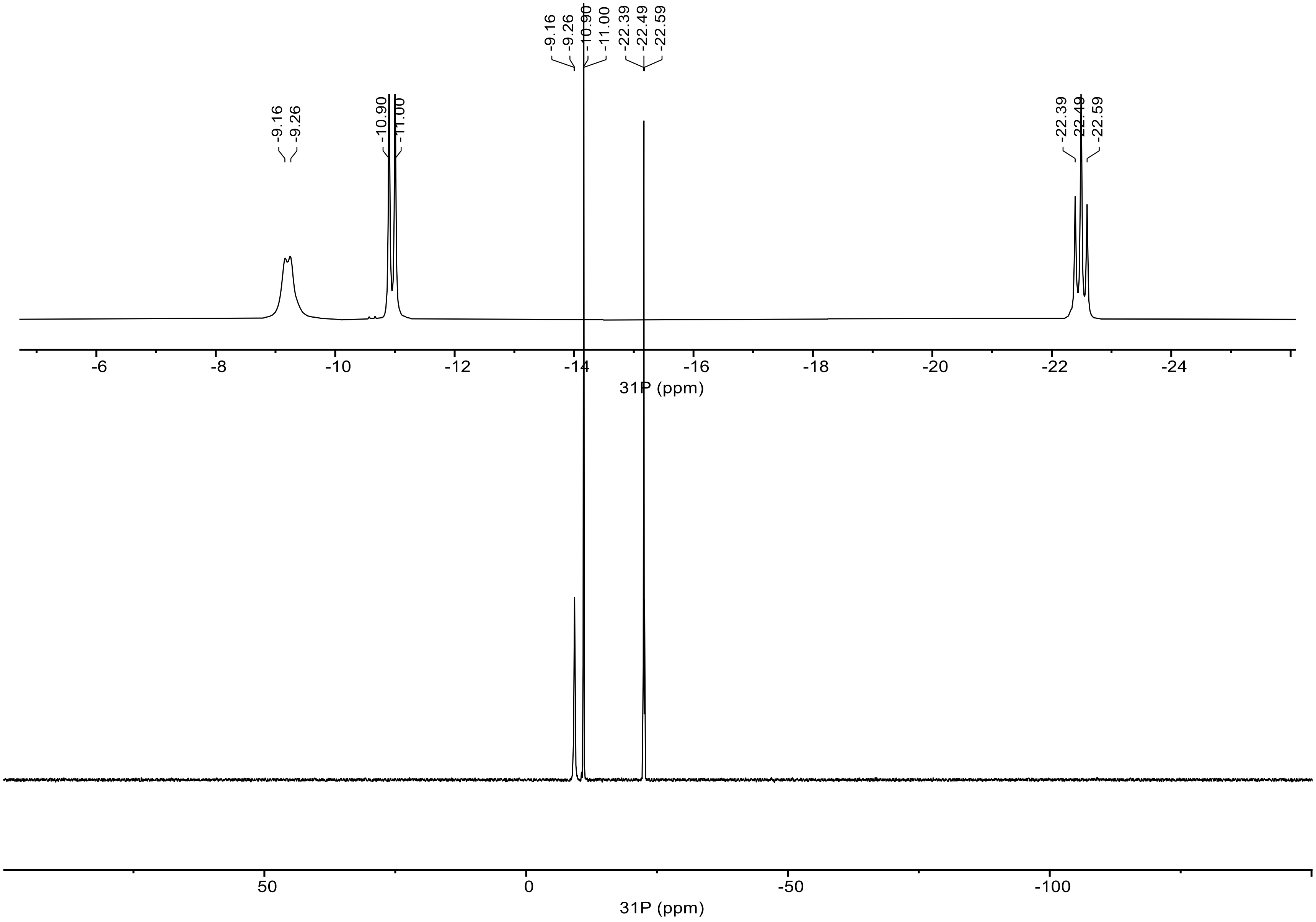

Supplement: Supplementary file 4 — Supplementary Data 1 [file 42004_2024_1354_MOESM4_ESM.pdf]
