# Supplementary material for: Bacteriophage-related epigenetic natural and non-natural pyrimidine nucleotides and their influence on transcription with T7 RNA polymerase
Source: Commun Chem. 2024 Nov 9;7:256. doi: 10.1038/s42004-024-01354-5 (PMC11550810; doi:10.1038/s42004-024-01354-5)
Supplement: Supplementary file 5 — reporting-summary [file 42004_2024_1354_MOESM5_ESM.pdf]

nature portfolio

Corresponding author(s): Michal Hocek

Last updated by author(s): Oct 15, 2024

Reporting Summary

Nature Portfolio wishes to improve the reproducibility of the work that we publish. This form provides structure for consistency and transparency in reporting. For further information on Nature Portfolio policies, see our Editorial Policies and the Editorial Policy Checklist.

Statistics

For all statistical analyses, confirm that the following items are present in the figure legend, table legend, main text, or Methods section.

n/a	Confirmed	
☐	☒	The exact sample size (n) for each experimental group/condition, given as a discrete number and unit of measurement
☐	☒	A statement on whether measurements were taken from distinct samples or whether the same sample was measured repeatedly
☒	☐	The statistical test(s) used AND whether they are one- or two-sided<br>*Only common tests should be described solely by name; describe more complex techniques in the Methods section.*
☒	☐	A description of all covariates tested
☒	☐	A description of any assumptions or corrections, such as tests of normality and adjustment for multiple comparisons
☐	☒	A full description of the statistical parameters including central tendency (e.g. means) or other basic estimates (e.g. regression coefficient) AND variation (e.g. standard deviation) or associated estimates of uncertainty (e.g. confidence intervals)
☒	☐	For null hypothesis testing, the test statistic (e.g. F, t, r) with confidence intervals, effect sizes, degrees of freedom and P value noted<br>*Give P values as exact values whenever suitable.*
☒	☐	For Bayesian analysis, information on the choice of priors and Markov chain Monte Carlo settings
☒	☐	For hierarchical and complex designs, identification of the appropriate level for tests and full reporting of outcomes
☒	☐	Estimates of effect sizes (e.g. Cohen's d, Pearson's r), indicating how they were calculated

Our web collection on statistics for biologists contains articles on many of the points above.

Software and code

Policy information about availability of computer code

Data collection	No software used beyond standart instrument operating software that was enclosed to following instruments: gel scans were acquired by Typhoon FLA 9500 (GE Healthcare) or Amersham Typhoon (Cytiva). LC-MS data were acquired at Agilent 1290 Infinity II BIO system. NMR samples were measured on Bruker Avance 400 III HD, Avance 500 III HD and Avance 600 III HD. High resolution MS spectra were collected on LTQ Orbitrap XL spectrometer (Thermo Fisher Scientific). IR spectra were measured on Bruker ALPHA spectrometer
Data analysis	Microsoft Excel was used to generate graphs and to analyze data from in vitro transcriptions and sequencing. NMR spectra were processed by MestReNova program. ImageJ (1.54f) was employed for gel densitometric analyses. UniDec (v 6.0.3) was used for biomolecule MS raw spectra deconvolution. Standard bioinformatics tools and custom in-house scripts were used to process NGS data.

For manuscripts utilizing custom algorithms or software that are central to the research but not yet described in published literature, software must be made available to editors and reviewers. We strongly encourage code deposition in a community repository (e.g. GitHub). See the Nature Portfolio guidelines for submitting code & software for further information.

Data

Policy information about availability of data

All manuscripts must include a data availability statement. This statement should provide the following information, where applicable:

- Accession codes, unique identifiers, or web links for publicly available datasets
- A description of any restrictions on data availability
- For clinical datasets or third party data, please ensure that the statement adheres to our policy

All data generated or analyzed during this study are included in this published article and its supplementary information files. Source data are provided with this paper as a link to repository.

Research involving human participants, their data, or biological material

Policy information about studies with human participants or human data. See also policy information about sex, gender (identity/presentation), and sexual orientation and race, ethnicity and racism.

Reporting on sex and gender	Not relevant, since no experiments on humans were performed in this study.
Reporting on race, ethnicity, or other socially relevant groupings	Not relevant, since no experiments on humans were performed in this study.
Population characteristics	Not relevant, since no experiments on humans were performed in this study.
Recruitment	Not relevant, since no experiments on humans were performed in this study.
Ethics oversight	Not relevant, since no experiments on humans were performed in this study.

Note that full information on the approval of the study protocol must also be provided in the manuscript.

Field-specific reporting

Please select the one below that is the best fit for your research. If you are not sure, read the appropriate sections before making your selection.

☒ Life sciences ☐ Behavioural & social sciences ☐ Ecological, evolutionary & environmental sciences

For a reference copy of the document with all sections, see nature.com/documents/nr-reporting-summary-flat.pdf

Life sciences study design

All studies must disclose on these points even when the disclosure is negative.

Sample size	No sample size calculations were performed. Initial triplicate provided consistent data and therefore no additional replications were performed.
Data exclusions	Initial in vitro testing of first batch of templates with sense-modified promoter provided consistent results, but later the Cx-modified precursors (37DNA_Cx) were analyzed on LC-MS and it showed inconsistent mass across all modifications, suggesting that prepared templates containing C-modifications were not of good quality. These templates and results were therefore discarded and a new batch of templates was prepared, using newly prepared C-modified precursors. These templates have shown different results for C-modified templates (higher efficiency of transcriptions).
Replication	Quantifications of templates were done at least in duplicate, using agarose gel electrophoresis. If the results were consistent, no more replicates were performed. In case of one or more inconsistencies, additional quantification replicate was performed.
Randomization	No randomization was performed. All used molecules and biomolecules were properly purified and characterized.
Blinding	Blinding was not relevant to this research because no animals or humans were used. All quantitative measurements were carried out unbiasedly, such as the use of ImageJ software.

Reporting for specific materials, systems and methods

We require information from authors about some types of materials, experimental systems and methods used in many studies. Here, indicate whether each material, system or method listed is relevant to your study. If you are not sure if a list item applies to your research, read the appropriate section before selecting a response.

Materials & experimental systems

n/a	Involved in the study	
☒	☐	Antibodies
☒	☐	Eukaryotic cell lines
☒	☐	Palaeontology and archaeology
☒	☐	Animals and other organisms
☒	☐	Clinical data
☒	☐	Dual use research of concern
☒	☐	Plants

Methods

n/a	Involved in the study	
☒	☐	ChIP-seq
☒	☐	Flow cytometry
☒	☐	MRI-based neuroimaging

Plants

Seed stocks	Not relevant, since no experiments on plants were performed in this study.
Novel plant genotypes	Not relevant, since no experiments on plants were performed in this study.
Authentication	Not relevant, since no experiments on plants were performed in this study.
